# Introducing DInaMo: A Package for Calculating Protein Circular Dichroism Using Classical Electromagnetic Theory

**DOI:** 10.3390/ijms160921237

**Published:** 2015-09-07

**Authors:** Igor V. Uporov, Neville Y. Forlemu, Rahul Nori, Tsvetan Aleksandrov, Boris A. Sango, Yvonne E. Bongfen Mbote, Sandeep Pothuganti, Kathryn A. Thomasson

**Affiliations:** 1 Chemistry Department, University of North Dakota, 151 Cornell St. Stop 9024, Grand Forks, ND 58202, USA; E-Mails: iuporov@gmail.com (I.V.U.); nforlemu@ggc.edu (N.Y.F.); rahul.nori@my.und.edu (R.N.); tsvetan.aleksandrov@gmail.com (T.A.); boris.sango@my.und.edu (B.A.S.); yvonne.mbote@okbu.edu (Y.E.B.M.); sandeeppothuganti@gmail.com (S.P.); 2 Faculty of Chemistry, M. V. Lomonosov Moscow State University, GSP-1, 1-3 Leninskiye Gory, 119991 Moscow, Russia; 3 Georgia Gwinnett College, 1000 University Center Lane, Lawrenceville, GA 30043, USA; 4 James E. Hurley College of Science & Mathematics, Oklahoma Baptist University, OBU Box 61772, 500 W. University, Shawnee, OK 74804, USA

**Keywords:** dipole interaction model, far-UV circular dichroism, theoretical circular dichroism calculations, computer program, α-helical proteins, β-sheet proteins, α/β proteins, other secondary structures

## Abstract

The dipole interaction model is a classical electromagnetic theory for calculating circular dichroism (CD) resulting from the π-π* transitions of amides. The theoretical model, pioneered by J. Applequist, is assembled into a package, DInaMo, written in Fortran allowing for treatment of proteins. DInaMo reads Protein Data Bank formatted files of structures generated by molecular mechanics or reconstructed secondary structures. Crystal structures cannot be used directly with DInaMo; they either need to be rebuilt with idealized bond angles and lengths, or they need to be energy minimized to adjust bond lengths and bond angles because it is common for crystal structure geometries to have slightly short bond lengths, and DInaMo is sensitive to this. DInaMo reduces all the amide chromophores to points with anisotropic polarizability and all nonchromophoric aliphatic atoms including hydrogens to points with isotropic polarizability; all other atoms are ignored. By determining the interactions among the chromophoric and nonchromophoric parts of the molecule using empirically derived polarizabilities, the rotational and dipole strengths are determined leading to the calculation of CD. Furthermore, ignoring hydrogens bound to methyl groups is initially explored and proves to be a good approximation. Theoretical calculations on 24 proteins agree with experiment showing bands with similar morphology and maxima.

## 1. Introduction

Circular Dichroism (CD) is a powerful structural biology method, critical for examining and evaluating protein conformational changes, protein folding dynamics, and most importantly secondary structural elements in proteins and peptides [1]. CD spectroscopy offers some salient advantages, such as simplicity, nondestructive procedure, rapid performance and small amounts of materials in the determination of molecular shape; it functions well even for large multimeric proteins that can neither be crystallized nor measured with NMR [2]. CD, therefore, provides considerable information about protein structures quickly and easily. This makes it important to understand the theory behind this chiroptical spectroscopic technique and doing so is still a major challenge [3].

Theoretical circular dichroism can enhance the interpretation of experimental CD, rapidly assist in determination of favorable solution conformations important for biological function, and predict the CD spectra of peptides and proteins [3]. Theoretical calculation of CD spectra is based on the characterization of the chromophores involved [4]. Both classical electromagnetic and quantum mechanical theories are currently being used to predict protein and peptide CD spectra with knowledge of their structure. Quantum mechanical methods achieve spectra prediction by direct evaluation of the dipole and rotational strengths of a molecule through determination of wave functions for the chromophores, particularly the amide chromophore. Classical methods, on the other hand, do not require the determination of the wave functions, but use empirically derived atomic polarizabilities and transition dipoles to predict the dipole and rotational strengths needed to calculate CD. Both methods are useful for predicting far-UV CD for proteins, but each has its own advantages and disadvantages.

One major advantage to quantum CD predictions is its ability to treat multiple transitions, from the amide π-π* and n-π* to aromatic chromophores such as phenylalanine or tryptophan. The major disadvantage is the inability of including all the nonchromophoric atoms in the calculations, although some nonchromophoric atoms may be included [5]; this means some side chains (e.g., proline) may be neglected which could have consequences for non α-helical structures such as poly-l-proline II [6,7]. For example, the first quantum mechanical prediction of a wide variety of proteins including collagen and poly-l-proline II CD worked with models represented by the backbone atoms including the amide hydrogen [8]. Significant improvement with poly-l-proline structures were achieved quantum mechanically using a poly-alanine model in the poly-l-proline II conformation, but the structure was effectively truncated from proline to alanine [5]. Classical methods that included the full proline side chain, were sensitive enough to reproduce CD, and when comparing calculations to experiment, estimated how puckered the proline ring was [9]. A brief review of current quantum mechanical methods follows.

CD predictions for proteins applying quantum mechanics are currently being done with matrix methods using parameters derived from various quantum mechanical (QM) techniques. The semiempircal quantum matrix method derives from the π-π* transition dipole moment obtained from experiments with *N*-acetylglycine and propanamide [10,11] and the other parameters (n-π* and transitions connecting π-π* and n-π* excited states) calculated quantum mechanically using the intermediate neglect of differential overlap/spectroscopic (INDO/S) wave functions for *N*-methylacetamide [12]. These parameters then allow for treating whole peptides and proteins [13,14,15,16,17,18,19,20,21,22,23]. Furthermore, very high-level *ab initio* calculations on *N*-methylacetamide: CASSCF/SCRF (complete active space self-consistent-field method implemented within a self-consistent reaction field) combined with multiconfigurational second-order perturbation theory (CASPT2-RF) [6,24] yields other very useful matrix method parameters. This latter matrix method has even been extended to include the charge-transfer transitions between amides observed in the vacuum-ultraviolet region of the CD spectrum of proteins [25].

Recently, QM has been combined with molecular mechanics (MM) and molecular dynamics (MD) to include dynamic fluctuations of the protein structures [26,27,28,29]. The molecular mechanics provides MD snapshots of the protein structure and the QM parameters for the amide transitions are used with each snapshot. MD/CD predictions applying free energy profile principle component analysis have been applied to chicken villin headpiece [26]. QM and MM are combined to create charge population analysis for the MD samples (exciton Hamiltonian with electrostatic fluctuations: EHEF) [29]. This algorithm avoids repeated QM calculations by determining the fluctuating Hamiltonian for all MD snapshots and has been tested on several proteins [29]. CD is predicted using MD/semiempirical QM combined with time-dependent DFT for carbonic anhydrase II [30]. QM/MD parameterized with experimental data and semiempirical molecular orbitals using intermediate neglect of differential overlap successfully predicts CD for amyloid fibrils [27].

Classical physics approaches, such as the dipole interaction model, based on coupled oscillator models, also predict far-UV CD for proteins. The dipole interaction model developed by Jon Applequist [31,32] from DeVoe’s theory [33,34] relies on changes in dipole moment, and therefore utilizes atomic and molecular polarizabilities. In the dipole interaction model, the amide chromophores (NC′O) are characterized as a single point with anisotropic polarizability, centered at or near the midpoint of the N-C′ bond; while the rest of the molecule (non-chromophoric portion) including hydrogens, backbone and side chain atoms are characterized by isotropic polarizability [35,36,37]. The dipole interaction model is well parameterized to predict the far-UV electric dipole allowed peptide π-π* transitions, which are empirically derived from the anisotropies, molar Kerr constants, polarizabilities and polar angles of small amides including: formamide, acetamide, *N*-methylformamide, *N*-methylacetamide, *N*,*N*-dimethylformamide, *N*,*N*-dimethylacetamide, trifluoroacetamide, trichloroacetamide, tribromoacetamide, *N*-methyltrifluoroacetamide, *N*-methyltrichloroacetamide, and *N*-methyltribromoacetamide [36]. The atomic polarizabilities for nonchromophoric elements (C (aliphatic), O (alcohol), and H (aliphatic or alcohol or amide)) are obtained experimentally from least squares fitting to molecular polarizabilities of small organic molecules determined at the NaD line (589.3 nm) [31,32,35]. This model has been successful in predicting CD spectra for β-sheets [38], β-turns [39], α-helices [40], and β-peptides [41] that are in good agreement with experimentally published data. The dipole interaction model is also the only successful method in predicting π-π* CD for both forms of poly-l-proline [42] and a small model of collagen [43]. The dipole interaction model also succeeded in the calculation of the CD spectra of small proteins like erabutoxin, myoglobin, cytochrome c, prealbumin, papain and ribonuclease A [3].

Synchrotron radiation circular dichroism (SRCD) is a technique with new data in the vacuum UV region (150–190 nm) characterized by greater sensitivity that is being made available in the Protein Circular Dichroism Data Bank (PCDDB) [44]. Although it is not necessary to have SRCD for secondary structure analysis or comparing theoretical calculations of the π-π* of the amide chromophore, the great advantage of the PCDDB is that the spectra contained within are well refereed and standardized so that the research community can depend on the high quality of experimental CD just as the community can depend on the high quality of crystal structures found in the Protein Data Bank (PDB). Even the raw sample spectra, raw baseline spectra, average sample and averaged baseline, the net smoothed spectrum and the final processed spectrum are all made available in both digital and graphical formats. Furthermore, SRCD is sensitive to different kinds of protein folds [45]; SRCD is able to detect protein-protein interactions (*i.e.*, quaternary or quinary structures) [46], as well as significantly expanding secondary structure analysis [47]. Thus, SRCD data provides a new avenue to evaluate and test theoretical CD calculations, even for the π-π* transitions.

Herein, the dipole interaction model is assembled into a single program package (DInaMo) written in Fortran and then tested with several different proteins. Comparisons of theoretical calculations are made with SRCD data when available. A variety of different proteins exhibiting a variety of different secondary structures are considered. This is the first attempt to use molecular mechanics as a structure-generating technique to include the entire tertiary structure of the protein and not just rebuild the secondary structures as has been previously done [3]. Furthermore, it is also a first attempt at applying a united atom approach to the nonchromophoric parts of the protein.

### 1.1. Theory

The dipole interaction model consists of *N* units that interact with each other by way of the fields of their induced electric dipole moments in the presence of a light wave [35,48]. A unit may be an atom, a group of atoms, or a whole molecule. For peptides and proteins, it is the amide group NC′O that is a single unit chromophore, and the aliphatic atoms are either treated as individual units or as units in a united atom approach where hydrogens are collapsed onto the atom to which they are bound. Polarizabilities are largest for the chromophoric points and smaller for the nonchromophoric points, with hydrogens having the smallest polarizabilities, so that it is sometimes possible to ignore a hydrogen polarizability contribution in the calculation. Oscillator *s* on unit *i* is polarized along the unit vector **u_is_** [49]. The polarizability (**α*_i_***) of oscillator *is* is *a_is_***u_is_u_is_**, where *a_is_* is a complex function of frequency [49]. Unit *i*, located at position ***r_i_*** has induced dipole moment **μ*_i_*** [48]. ***E_i_*** is the electric field at ***r_i_*** due to the light wave [48].

#### 1.1.1. Dipole Interactions

The interaction among the dipoles is expressed by Equation (1), where ***T_ij_*** is the dipole field tensor, which is a function of the positions, ***r_i_*** and ***r_j_***, of the two dipoles [48].

(1) $$\mu_{i}=\alpha_{i}\left[E_{i}-\overset{N}{\underset{j=1}{\sum }}T_{ij}\mu_{j}\right]$$

The matrix form of the system of equations represented by Equation (1) becomes

(2) Aμ=E

where μ is a column vector of the moments μ_***i***_, ***E*** is a column vector of the fields ***E_i_***, and the square interaction matrix ***A*** contains the elements [49]:

(3) $$A_{is,jt}=\{\begin{array}{c} a_{is}^{-1}\delta_{st}\left(i=j\right)\\ u_{is,\alpha }T_{ij,\alpha \beta }u_{jt,\beta }\left(i\neq j\right) \end{array}$$

The solution to Equation (2) is

(4) μ=BE

where ***B*** = ***A^−1^*** [48]. Optical properties are determined by Equation (4) using the coefficients of the various field terms [48].

#### 1.1.2. Normal Modes

Optical absorption and dispersion phenomena are expressed most easily in terms of normal modes of the system of coupled dipole oscillators [48,50,51]. Unit *i* has a number of dipole oscillators that are indexed by *is* with polarizability **α_*is*_** along a unit vector ***u_is_*** [48,50,51]. Band shapes are assumed to be Lorentzian so that the dispersion of an isolated oscillator is represented by a Lorentzian function having wavenumber ${\bar{\nu }}_{is}$ with a half-peak bandwidth of Γ.

(5) $$\alpha_{is}=\frac{D_{is}u_{is}u_{is}}{{\bar{\nu }}_{is}^{2}-{\bar{\nu }}^{2}+i\Gamma \bar{\nu }}$$

*D_is_* represents a constant related to the dipole strength, and $\bar{\nu }$ is the vacuum wavenumber of the light [48]. Equation (2) reduces to an eigenvalue problem where the eigenvalues of ***A°*** (the ***A*** matrix at $\bar{\nu }$ = 0) are a set of squares of normal mode wavenumbers ${\bar{\nu }}_{k}^{2}$ and the normalized eigenvectors ***t*^(*k*)^** are column vectors whose components are the relative amplitudes of the dipole moments of the oscillators [48]. Relative amplitudes of the electric dipole moment **μ^(*k*)^** and magnetic dipole moment ***m*^(*k*)^** for the system in the *k*-th normal model are given by

(6) $$\mu^{\left(k\right)}=\underset{is}{\sum }t_{is}^{\left(k\right)}u_{is}$$

(7) $$m^{\left(k\right)}=\underset{is}{\sum }t_{is}^{\left(k\right)}r_{i}\times u_{is}$$

Dipole strength *D_k_* and rotational strength *R_k_* associated with the *k*-th normal mode are expressed as

(8) $$D_{k}=\mu^{\left(k\right)}•\mu^{\left(k\right)}$$

(9) $$R_{k}=\mu^{\left(k\right)}•m^{\left(k\right)}$$

#### 1.1.3. Partially Dispersive Approximation

If any of the natural wavenumbers ${\bar{\nu }}_{is}$ are far above the spectral region of interest, the corresponding oscillators are approximately nondispersive. The normal mode problem can be simplified by partitioning the ***A°*** matrix into blocks [48,50,51].

(10) $$A^{o}=\left(\begin{array}{cc} A_{11}^{o} & A_{12}\\ A_{21} & A_{22}^{o} \end{array}\right)$$

The $A_{11}^{o}$ block contains the coefficients relating the dispersive oscillators to each other (*i.e.*, the chromophoric part of the system), the $A_{22}^{o}$ block contains the nondispersive oscillators (*i.e.*, the nonchromophoric part of the system), and the ***A*_12_** and the ***A*_21_** blocks contain the interactions between the two subsystems [48,50,51]. The normal modes in the spectral region of interest (e.g., far-UV for proteins) are those of the matrix

(11) $$A_{11}^{o}-A_{12}{\left(A_{22}^{0}\right)}^{-1}A_{21}$$

This means the order of the eigenvalue problem is significantly smaller than the full matrix *A* [48]. The advantage in computational efficiency is substantial in systems with only a few dispersive oscillators and many nondispersive oscillators [48]. For example, a small protein such as lysozyme has 128 dispersive oscillators representing the amide groups in the backbone while all other atoms including the hydrogens are treated as nondispersive (1037 units). This problem can be further reduced by ignoring hydrogens attached to CH_3_ groups altogether or collapsing them onto the C to which they are bound. For lysozyme this reduces the number of nondispersive units to 696.

#### 1.1.4. Spectra

Absorption molar extinction coefficient ε and circular dichroism Δε at each wavenumber are calculated as sums over the Lorentzian bands for all normal modes [36].

(12) $$\varepsilon =\frac{8\pi^{2}{\bar{\nu }}^{2}N_{A}\Gamma }{6909p}\overset{q}{\underset{k}{\sum }}\frac{D_{k}}{{\left({\bar{\nu }}_{k}^{2}-{\bar{\nu }}^{2}\right)}^{2}+\Gamma^{2}{\bar{\nu }}^{2}}$$

(13) $$\Delta \varepsilon =\frac{32\pi^{3}{\bar{\nu }}^{3}N_{A}\Gamma }{6909p}\overset{q}{\underset{k}{\sum }}\frac{R_{k}}{{\left({\bar{\nu }}_{k}^{2}-{\bar{\nu }}^{2}\right)}^{2}+\Gamma^{2}{\bar{\nu }}^{2}}$$

where *N_A_* is Avogadro’s number and *p* is the number of peptide residues; *q* is equal to *p*-1 for a monomeric structure because there is only one dispersive oscillator for each amide π-π* transition [36]. It is possible to have more dispersive oscillators per peptide (e.g., for the n-π* transition), but more work needs to be done to parameterize the n-π* transition, which is beyond the scope of this paper.

## 2. Results and Discussion

A note to the reader: it may be very helpful to briefly look through Section 3. Computational Methods section before completely reading the Results and Discussion because the parameters used and program pieces are described thoroughly there.

Comparing SRCD data or conventional CD data, the location of the bands is essentially the same in both cases for the region between 180 and 250 nm because the transitions (π-π* and n-π* are the same), but the ability of conventional CD to clearly reach as low as 180 nm is often challenging (e.g., for insulin conventional CD for insulin was recorded in the region between 195 and 240 [52]). Furthermore, the data available in the PCDDB is fully refereed, available and downloadable, making it an excellent choice of experimental spectra for comparison to theoretical calculations.

### 2.1. Lysozyme as a Benchmark to Examine Computational Methods

**Figure 1:**
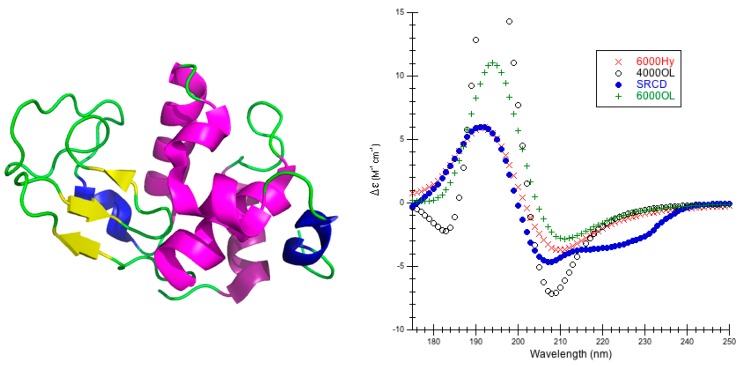
Lysozyme. (**Left**) Secondary structure of lysozyme (PDB code 2VB1 [56]) is shown: thick purple cartoons/coils correspond to α-helices (4–15, 24–37, 88–100, 108–115), the short blue cartoons/coils correspond to 3_10_-helices (80–85, 108–115) the yellow tapes are β-sheets, (43–45, 51–53, and 58–59) and the thin green ropes are turns and other structures; (**Right**) Predicted CD Using CDCALC and 2VB1 Minimized via NAMD/CHARMM22. Calculated spectra ignore all CH_3_ hydrogens. The 6000 and 4000 refer to bandwidths in cm^−1^. Calculated spectrum show the smallest RMSD 6000Hy (×), the largest RMSD 4000OL (o), and the most commonly successful for mainly alpha proteins, 6000OL (+). The blue dots (•) are the experimental SRCD (CD0000045000) [44,47]. The CATH fold classification [53] is mainly alpha/orthogonal bundle.

Lysozyme is a compact globular protein comprising a single polypeptide chain of 129 amino acids that CATH classifies as a mainly alpha type structure [53]. It is an enzyme that catalyzes the hydrolysis of 1,4-beta-linkages in peptidoglycans found in the cell walls of bacteria [54]. Lysozyme is actually a mixture of the major secondary structures, with four α-helices (30.2%), three β-sheets (6.2%), several turns (24%), three short 310-helices (10.1%), a β-bridge (4.7%), the rest is 9.3% bends and 15.5% irregular [44] (Figure 1).

The different minimizations of lysozyme result in structures that retained all α-helices, β-sheets, and turns, modifying the other more flexible structures the most. The root mean square deviation (RMSD) between experiment and calculated CD is smallest when α-helical parameters H (see Section 3.2 for more details about the parameters) and a bandwidth of 6000 cm^−1^ are used with any structure generation method (extensive minimization via Insight®II/Discover, moderate minimization with NAMD and ignoring hydrogens on methyl groups, or rebuilding with CAPPS) (Table 1, Table S1). The best RMSD is calculated for the structure where methyl hydrogens are ignored indicating this is a reasonable method to use. Both the CDCALC ignoring methyl hydrogens and the CAPPS results are as good as or better depending on the method than RMSDs determined from digitized data out of the literature [3,13,55]; the RMSD range in the literature calculations, however, is much smaller than the ranges across all parameters tested in DInaMo, suggesting that most matrix methods are not as sensitive to structure as the dipole interaction model. In all DInaMo calculations, the 6000 cm^−1^ bandwidth resembled experiment the most (Table S1, Figures S1–S3). Comparing the location and intensities of the peaks, CDCALC with the NAMD structure ignoring methyl hydrogens (Figure 1, Table S1, Figure S1) and CAPPS (Figure S2) reproduce both bands best using helical parameters, although the location of the chromophore impacts each prediction slightly. CDCALC with the Insight®II/Discover structure that included all hydrogens (Table S1 and Figure S3) does best with the helical parameters as well, but these predictions do not favor a single bandwidth; the 6000 cm^−1^ bandwidth reproduces the positive best band (peak), while the 4000 cm^−1^ bandwidth reproduced the negative peak best; this is a similar observation to previous dipole interaction model predictions [3]. The poly-l-proline II parameters consistently shift predicted CD to the red for both bands (Table S1, Figures S1 and S2). Based on the lysozyme results, the majority of the CDCALC predictions for other proteins are done with the NAMD minimized structures and ignore methyl hydrogens because these produced reasonable results with the least amount of computational effort.

### 2.2. α-Helical Proteins

All mainly α-helical proteins tested yield the general morphology of the CD spectrum in the π-π* region for both CDCALC and CAPPS. Predictions generally are slightly better for CDCALC than CAPPS based on RMSD values (Table 1, Tables S1–S9), but the difference is not large. RMSDs for the predicted spectra range from 0.756 M^−1^·cm^−1^ for cytochrome c to 10.337 M^−1^·cm^−1^ for bacteriorhodopsin using CDCALC with structure minimized using NAMD. CAPPS, on the other hand, ranges from 0.886 M^−1^·cm^−1^ for cytochrome c to 11.252 M^−1^·cm^−1^ for bacteriorhodopsin. The particular parameters that yield the best results varied from protein to protein and are not always the expected α-helical parameters (Tables S1–S9, Figures S1–S28). It is CAPPS that succeeds with helical parameters the most frequently; this is as expected since these parameters are designed to work with the rebuilt structure of CAPPS. CDCALC, on the other hand, uses energy minimized structures and does not remove turns or irregular loops; as a result, the original parameters most frequently yield the best comparison to experiment; e.g., for phospholipase A2 the RMSD is 0.994 M^−1^·cm^−1^. Generally, when the predicted CD does not locate a band precisely at the same place as an experiment, helical parameters slightly blue-shift CD (seen with CDCALC and CAPPS). The poly-l-proline II parameters, on the other hand, tend to yield red-shifted predictions. The CDCALC predictions in the π-π* region are typically as good as predictions in the literature; these include matrix method techniques using parameters that are semiempirical [13], *ab initio* [6,55,57], or exciton Hamiltonian with electrostatic fluctuations [29]; detailed RMSDs for reference calculations can be found in the Tables S1–S9. Herein, the newest protein, rhomboid peptidase, is presented as a representative example of α-helical proteins.

**Figure 2:**
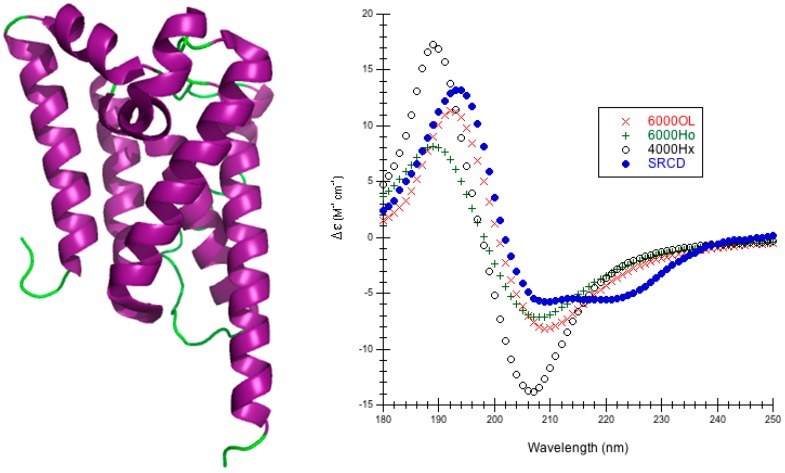
Rhomboid Peptidase. (**Left**) Secondary structure of rhomboid peptidase (PDB code 2NR9 [58]): thick purple cartoons/coils correspond to α-helices (9–28, 30–39, 43–50, 51–56, 57–59, 62–85, 85–109, 115–132, 152–157, 165–192) and the thin green ropes are turns and other structures; (**Right**) Predicted CD using CDCALC. The 2NR9 structure was minimized with 10,000 conjugate gradient steps using NAMD/CHARMM22. Calculated spectra ignore all CH_3_ group hydrogens. The 6000 and 4000 refer to bandwidths in cm^−1^. Calculated spectrum show the smallest RMSD 6000OL (×), the largest RMSD 4000Hx (o), and an example helical parameter result, 6000Ho (+). The blue dots (•) are the experimental SRCD (CD0000109000) [44,59].

Rhomboid Peptidase: PDB code 2NR9 is a moderate-size monomeric (196 amino acids) regulated intramembrane peptidase that cleaves transmembrane segments of integral membrane proteins (Figure 2) [58]. Rhomboid peptidase is 61.7% α-helix, 4.1% 3_10_-helix, 6.5% β-strand, 10.7% bonded turns, 7.7% bend, and 15.8% irregular [44]. CATH classifies rhomboid peptidase as a single domain that is mainly alpha/up-down bundle [53].

**Table 1:** CD Analysis of α-Helical Proteins. All RMSDs are calculated between 180 and 210 nm.

CD Method	Wavelength (nm)	Δε (M^−1^·cm^−1^)	Wavelength (nm)	Δε (M^−1^·cm^−1^)	RMSD (M^−1^·cm^−1^)	Range RMSDs † (M^−1^·cm^−1^)
**Lysozyme (Figure 1)**						
^a^ SRCD (CD0000045000) [47]	191	6.01	207	−4.68	0.000	
^b^ 6000Ho (PDB code 2VB1)	190	6.51	205	−1.83	1.620	1.620–5.783
^c^ 6000OL (PDB code 2VB1)	192	12.89	211	−2.81	3.585	0.935–7.477
^d^ 6000Ho (PDB code 2VB1)	190	6.49	208	−4.03	1.061	1.061–4.068
^e^ MM3 (PDB code 7LYZ)	192	5.37	210	−4.23	0.930	0.930–3.194
**Cytochrome c (Figure S4)**						
^a^ SRCD (CD0000021000) [47]	195	4.30	210	−4.29	0.000	
^c^ 6000OL (PDB code 1HRC)	192	5.04	210	−4.29	0.756	0.756–3.506
^d^ 6000Ho (PDB code 1HRC)	190	8.00	208	−6.52	3.036	0.886–7.617
^f^ BA98:2 (PDB code 1HRC)	184	8.17	206	−10.37	1.843	1.183–3.242
**Phospholipase A2 (Figure S7)**						
^a^ SRCD (CD0000059000) [47]	192	6.96	209	−4.63	0.000	
^c^ 6000OL (PDB code 1UNE)	191	8.54	210	−5.92	0.994	0.994–5.435
^d^ 6000Ho (PDB code 1UNE)	190	6.92	206	−5.53	1.821	1.821–5.313
^e^ MM3 (PDB code 1UNE)	191	9.37	209	−7.25	1.831	1.831–2.557
**Rhomboid Peptidase (Figure 2)**						
^a^ SRCD (CD0000109000) [59]	193	13.20	210	−5.77	0.000	
^c^ 6000OL (PDB code 2NR9)	192	11.33	209	−8.14	1.367	1.367–4.546
^d^ 6000Ho (PDB code 2NR9)	190	9.14	208	−7.47	4.526	3.704–7.959
**Calmodulin (Figure S12)**						
^a^ SRCD (CD0000013000) [47]	192	12.57	208	−6.58	0.000	
^c^ 6000OL (PDB code 1LIN)	192	9.30	209	−6.51	1.734	1.734–5.278
^d^ 6000Ho (PDB code 1LIN)	190	7.01	206	−4.24	3.453	3.082–4.755
^g^ MM2 (PDB code 1LIN)	192	11.93	210	−8.21	0.933	0.933–1.281
**Leptin (Figure S15)**						
^a^ SRCD (CD0000044000) [47]	191	13.20	207	−7.48	0.000	
^c^ 6000OL (PDB code 1AX8)	192	12.16	210	−7.17	2.071	2.071–8.142
^d^ 6000Ho (PDB code 1AX8)	190	10.92	208	−8.96	2.276	2.276–9.660
^h^ SI (PDB code 1AX8)	192	13.40	209	−10.85	2.437	2.437–8.328
**Bacteriorhodopsin (Figure S18)**						
^a^ SRCD (CD0000101000) [59]	195	15.67	214	−5.20	0.000	
^c^ 6000OL (PDB code 1QHJ)	192	14.27	210	−9.63	4.469	2.424–10.337
^d^ 6000Ho (PDB code 1QHJ)	190	10.45	208	−9.20	7.195	5.484–11.252
^i^ 6000Hy (PDB code 2BRD)	191	12.11	208	−9.61	5.985	5.985–9.952
**Horse Myoglobin (Figure S21)**						
^a^ SRCD (CD0000047000) [47]	192	16.75	209	−7.51	0.000	
^b^ 6000Ho (PDB code 3LR7)	189	15.49	205	−8.46	5.609	2.990–14.244
^c^ 6000OL (PDB code 2V1K)	192	11.65	210	−9.35	3.938	2.991–7.823
^d^ 6000Ho (PDB code 2V1K)	190	10.78	208	−8.29	4.946	4.946–8.261
^h^ MM1 (PDB code 1YMB)	192	16.80	211	−11.36	3.131	3.131–4.797
**Sperm Whale Myoglobin (Figure S25)**						
^a^ SRCD (CD0000048000) [47]	193	17.33	210	−7.77	0.000	
^b^ 6000Ho(PDB code 2JHO)	186	19.38	204	−6.07	8.344	2.392–12.070
^c^ 6000OL (PDB code 2JHO)	192	12.28	210	−9.29	3.988	3.169–8.131
^d^ 6000Ho (PDB code 2JHO)	188	10.88	208	−9.02	5.779	5.742–9.444
^j^ OH06:2 (PDB code unspecified)	191	16.86	209	−12.00	3.192	3.192–8.851

The DInaMo calculations are for the minimized or rebuilt structure using CDCALC or CAPPS. Example literature calculations are also listed when available. **†** The range of RMSDs of for all calculations including literature calculations is presented. For full RMSD information on all calculations including literature, please see the Supplementary Information for a full table of calculations with RMSDs for each protein. ^a^ SRCD from the PCDDB [44]; ^b^ CDCALC using PDB structure minimized via Insight^®^II/Discover/CVFF; ^c^ CDCALC using PDB structure minimized via NAMD/CHARMM22; ^d^ CAPPS with rebuilt secondary structures including hydrogens; ^e^ Matrix method using *ab initio* parameters including protein backbone, charge-transfer and side chain transitions [55]; ^f^ Dipole interaction model of rebuilt PDB structure with set Hy at 6000 cm^−1^ [3]; ^g^ Matrix method using *ab initio* parameters including protein backbone and charge-transfer transitions [55]; ^h^ Matrix method using *ab initio* parameters including only the protein backbone transitions [55]; ^i^ Dipole interaction model with rebuilt PDB structure with set Hy at 6000 cm^−1^ [3]; ^j^ Matrix method using unspecified myoglobin structure including local transitions and charge-transfer parameters [57].

This is the first attempt at a theoretical prediction of far-UV CD for rhomboid peptidase, most likely because it has been crystallized [58] fairly recently. RMSDs for predictions run as low as 1.367 M^−1^·cm^−1^ (6000OL, CDCALC) or as high as 7.959 M^−1^·cm^−1^ (4000 Hy, CAPPS) depending on the method and parameters (Table 1, and Table S4). For CDCALC, the original parameters (OL) with a bandwidth of 6000 cm^−1^ yielded the overall best RMSD, the best peak locations and the best intensities (Figure 2, Figure S10). The largest RMSD with CDCALC is also using the original parameters, but a bandwidth of 4000 cm^−1^. CDCALC and J parameters (poly-l-proline II) also appear to locate peaks well, but the peaks are slightly red-shifted; only the 4000 cm^−1^ bandwidth approaches the correct intensity around 193 nm, while the 6000 cm^−1^ bandwidth approaches the correct intensity at 210 nm. All H parameters (helical) yield slightly blue-shifted predicted spectra with CDCALC.

CAPPS for rhomboid peptidase similarly blue-shifts predictions using the helical (H) parameters and locates the peaks better with the poly-l-proline II (J) parameters (Supplementary Information Figure S11). Again, with the J parameter predicted intensities match best at 193 nm with the 4000 cm^−1^ bandwidth, and the 210 nm peak using the 6000 cm^−1^ bandwidth. This is similar to what was seen for α-helical proteins previously treated with the dipole interaction model (e.g., lysozyme, myoglobin) [3].

### 2.3. β-Sheet Proteins

DInaMo succeeds most frequently using the CDCALC method of simulating the CD spectrum for mainly beta type proteins (Table 2, Tables S10–S17, Figures S29–S46). RMSDs for CDCALC range from 1.408 M^−1^·cm^−1^ for jacalin (4000 Jo) to 4.798 M^−1^·cm^−1^ for outer membrane protein G (4000 Hx). Typically with CDCALC, the helical parameters locate peaks better than poly-l-proline II parameters, which most often are red-shifted; this pattern is observed for concanavalin A, outer membrane protein OPCA, rubredoxin, lentil lectin, pea lectin, avidin and outer membrane protein G. The exception is jacalin; CDCALC succeeds best with 4000 Jo parameters (RMSD 1.408 M^−1^·cm^−1^), but even this is red-shifted and weak compared to experiment. The original parameters with CDCALC are less predictable. Predictions sometimes resemble the helical parameter predictions (rubredoxin). Often predictions are very weak compared to the other parameter predictions (concanavalin A, outer membrane protein OPCA, the lentil and pea lectins, and outer membrane protein G). Sometimes predictions yield an incorrect sign for the peaks (jacalin), or predictions are simply red-shifted (avidin).

CAPPS has a tendency to fail for the larger mainly beta proteins (outer membrane protein OPCA, jacalin, pea lectin, and outer membrane protein G). When it does succeed, CAPPS typically yields a smaller RMSD than CDCALC (Table 2, Tables S10, S13, S14, and S16). The range of RMSDs for CAPPS is 0.681 M^−1^ cm^−1^ for concanavalin A (6000Hy) to 3.506 M^−1^·cm^−1^ for rubredoxin (4000 Jy). The poly-l-proline II parameters with CAPPS predictions are consistently weak and often red-shifted, and like CDCALC, the helical parameters perform better with CAPPS for mainly beta proteins.

**Table 2:** CD Analysis of β-Sheet Proteins. All RMSDs are calculated between 180 and 210 nm.

CD Method	Wavelength (nm)	Δε (M^−1^·cm^−1^)	Wavelength (nm)	Δε (M^−1^·cm^−1^)	RMSD (M^−1^·cm^−1^)	Range RMSDs † (M^−1^·cm^−1^)
**Concanavalin A (Figure S29)**						
^a^ SRCD (CD 0000020000) [47]	196	4.64	223	−2.25	0.000	
^b^ 4000 Hy (PDB code 1NLS)	199	3.09	211	−1.19	1.574	1.574–3.253
^c^ 6000 Hy (PDB code 1NLS)	198	4.53	216	−0.14	0.681	0.681–2.669
^d^ MM1 (PDB code 1NLS)	194	4.98	214	−1.44	1.518	1.518–3.375
**Outer Membrane Protein OPCA (Figure 3)**						
^a^ SRCD (CD0000119000) [59]	199	4.72	218	−1.56	0.000	
^b^ 4000Hy (PDB code 2VDF)	198	3.00	214	−0.322	1.625	1.526–2.959
**Jacalin (Figure S33)**						
^a^ SRCD (CD0000119000) [47]	192	−3.87	202	3.33	0.000	
^b^ 4000 Hy (PDB code 1KU8)	185	−1.56	199	1.72	2.001	1.408–2.558
^e^ MM3 (PDB code 1KU8)	183	−4.24	203	3.81	2.284	2.284–3.672
**Rubredoxin (Figure S35)**						
^a^SRCD (CD0000064000) [47]	191	1.47	202	−6.23	0.000	
^b^ 4000Hy (PDB code 1R0I)	189	3.21	206	−3.52	2.144	1.900–3.924
^c^ 6000Hy (PDB code 1R0I)	188	2.76	202	−2.70	1.886	1.472–3.506
^f^ BA98:1 (PDB code 8RXN)	192	4.30	210	−0.78	3.916	3.916–5.662
**Lentil Lectin (Figure S38)**						
^a^ SRCD (CD0000043000) [47]	195	5.43	226	−1.33	0.000	
^b^ 4000Hy (PDB code 1LES)	197	3.81	210	−1.29	1.887	1.887–3.571
^c^ 6000 Hy (1LES)	196	4.12	not observed	-	1.232	1.232–3.160
^g^ MM2 (1LES)	197	4.97	220	−1.32	0.415	0.415–2.997
**Pea Lectin (Figure S41)**						
^a^ SRCD (CD0000053000) [47]	196	5.05	226	−1.58	0.000	
^b^ 4000Hy (PDB code 1OFS)	198	3.12	210	−1.48	1.975	1.975–3.362
^d^ MM1 (PDB code 1OFS)	197	5.17	220	−1.35	0.373	0.373–2.084
**Avidin (Figure S43)**						
^a^ SRCD (CD0000008000) [47]	197	2.03	214	−0.04	0.000	
^b^ 4000 Hy (PDB code 2A8G)	200	5.04	211	−1.42	2.462	2.238–3.699
^c^ 6000 Hy (PDB code 2A8G)	200	3.36	not observed	-	2.435	2.092–3.421
^g^ MM2 (PDB code 1RAV)	197	5.31	218	−0.91	2.410	2.410–4.115
**Outer Membrane Protein G (Figure 4)**						
^a^ SRCD (CD0000118000) [59]	190	7.07	216	−3.13	0.000	
^b^ 4000 Hy (PDB code 2IWV)	203	2.51	213	−0.59	4.301	3.973–4.798

The DInaMo calculations are for the minimized or rebuilt structure using CDCALC or CAPPS. Example literature calculations are also listed when available. **†** The range of RMSDs is for all calculations including literature calculations is presented. For full RMSD information on all calculations including literature, please see the Supplementary Information for a full table of calculations with RMSDs for each protein. ^a^ SRCD from the PCDDB [44]; ^b^ CDCALC using PDB structure minimized via NAMD/CHARMM22; ^c^ CAPPS with rebuilt secondary structures including hydrogens; ^d^ Matrix method with *ab initio* parameters including only the protein backbone transitions [55]; ^e^ Matrix method using *ab initio* parameters including protein backbone, charge-transfer and side chain transitions [55]; ^f^ Dipole interaction model of rebuilt PDB structure [60] including residues 4–6, 8–12, 14–18, 20–22, 24–28, 30–32, 34–37, 39–44, 46–51 with set Hy at 4000 cm^−1^ [3]; ^g^ Matrix method using *ab initio* parameters including protein backbone and charge-transfer transitions [55].

Matrix method [55] and exciton Hamiltonian with electrostatic fluctuations [29] calculations for mainly beta proteins often yield RMSDs similar to those for CDCALC or CAPPS (Table 2, Supplementary Information Tables S10, S12–S16). Both the matrix method [55] and the exciton Hamiltonian with electrostatic fluctuations [29] yield better predictions for the lectins than DInaMo, but for jacalin, rubredoxin and avidin, the smallest DInaMo RMSDs are less than those for the matrix method [55]. Curiously, even the matrix method that includes all side chains fails to predict the negative band for rubredoxin at 225 nm or the positive band at 230 for avidin [55]. DInaMo also makes no prediction here, but this is to be expected since only the π-π* transition of the amide is being treated. Herein, details are presented for the two proteins for which there is very little theoretical CD currently presented in the literature, the two outer membrane proteins: OPCA and G.

**Figure 3:**
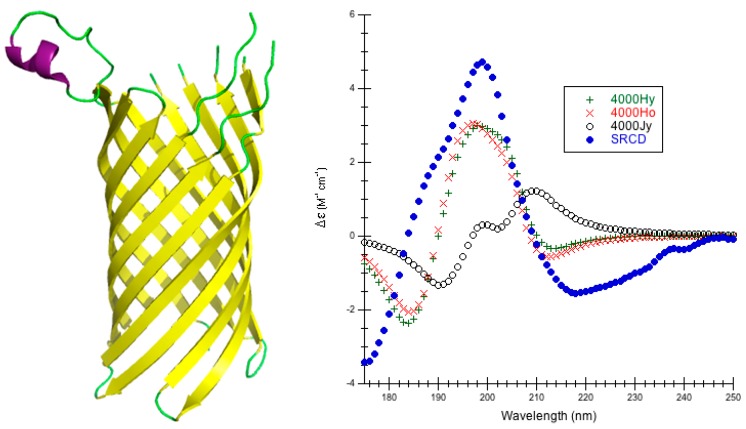
Outer Membrane Protein OPCA. (**Left**) Secondary structure of outer membrane protein OPCA (PDB code 2VDF [61]): thick purple cartoons/coils are α-helices (68–73), the yellow tapes are β-sheets (9–23, 26–43, 48–65, 85–103, 106–122, 131–150, 153–171, 182–185, 188–200, 240–253), and the thin green ropes are turns and other structures; (**Right**) Predicted CD Using CDCALC. The 2VDF [61] structure was minimized via 10,000 conjugate gradient steps with NAMD/CHARMM22. Calculated spectra ignore all CH_3_ group hydrogens. The blue dots (•) are the experimental SRCD (CD0000119000) [44,59]. The 6000 and 4000 refer to bandwidths in cm^−1^. Calculated spectrum show the smallest RMSD 4000Ho (×), the largest RMSD 4000Jy (o), and an example helical parameter result, 4000Hy (+). The CATH fold classification [53] is a single domain that is mainly beta/beta barrel.

Outer Membrane Protein OPCA: The integral outer membrane adhesin protein (PDB code 2VDF [61], outer membrane protein OPCA (OPCA)) is found in *Neisseria meningitidis*, which is the causative agent of meningococcal meningitis and septicemia. It binds sialic acid-containing polysaccharides on the surface of epithelial cells [61]. OPCA is a monomeric protein of 253 amino acids with 11 β-sheets and one α-helix (Figure 3) [61]. The PCDDB classifies the secondary structure as 1.6% α-helix, 66.8% β-strand, 0.8% β-bridge, 2.8% bonded turn, 2.8% bend, and 25.3% irregular [44].

This is a first attempt at predicting the far-UV CD spectrum for outer membrane protein OPCA. CDCALC produces a reasonably low RMSD with the helical parameters using a bandwidth of 4000 cm^−1^, the best being 4000 Ho, 1.526 M^−1^·cm^−1^ (Table 2, Figure 3, Table S11, Figure S32). The highest RMSD occurs for the 4000 Jy parameters. In general the poly-l-proline II parameters (Js) yield predictions that are weak in intensity and red-shifted. The original parameters also produce weak intensities, but are not as red-shifted as the Js. The helical parameters do a much better job of locating the peaks correctly and approximating intensity (Figure S32), particularly with a bandwidth of 4000 cm^−1^. CAPPS, on the other hand, completely fails to provide any predictions for the 2VDF structure.

**Figure 4:**
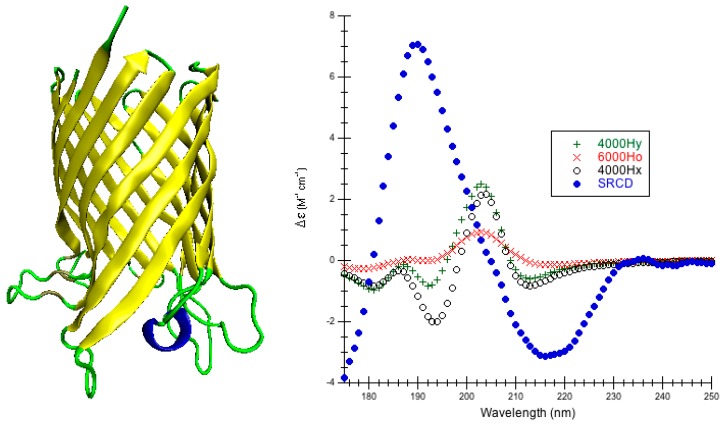
Outer Membrane Protein G. (**Left**) Secondary structure of outer membrane protein G (PDB code 2IWV [62]): the blue cartoons/coils correspond to α-helices (140–145), the yellow tapes are β-sheets (7–19, 43–56, 61–62, 70–79, 83–97, 204–218, 229–243, 248–261, 267–289), and the thin green ropes are turns and other structures; (**Right**) Predicted CD using CDCALC. The 2IWV structure was minimized via NAMD/CHARMM22/10,000 conjugate gradient steps. All CH_3_ group hydrogens are ignored. The 6000 and 4000 refer to bandwidths in cm^−1^. Calculated spectrum show the smallest RMSD 6000Ho (×), the largest RMSD 4000Hx (o), and an example helical parameter result, 4000Hy (+). The blue dots (•) are the experimental SRCD (CD0000119000) [44,59]. The CATH fold classification [53] is mainly beta/beta barrel.

Outer Membrane Protein G: 2IWV is a monomeric pore-forming protein found in *E. coli* outer membranes [62] that has 281 amino acids (Figure 4). The crystal structure is in the open state that occurs at pH 7 [62] as opposed to 2IWW that occurs at pH 5.6 that is a closed state where the pore is blocked by loop 6. CATH classifies the monomer of 2IWV as a single domain that is mainly beta/beta barrel [53]. The PCDDB classifies the secondary structure of 2IWV as 1.4% α-helix, 67.6% β-strand, 0.7% β-bridge, 7.7% bonded turn, 9.3% bend, and 13.3% irregular [44], and the experimental SRCD is measured at pH 8 [59].

DInaMo simulations of the far-UV CD of outer membrane protein G succeed for CDCALC, but not for CAPPS. All CDCALC predictions are weak and red-shifted compared to experiment, but the best predictions with the least shifting are for the helical parameters (Figure 4, Figure S46). The best RMSD occurs for the helical 6000 Ho (3.973 M^−1^·cm^−1^) (Table 2, Table S17). The worst RMSD also occurs with helical parameters (4000 Hx, 4.798 M^−1^·cm^−1^), but a different bandwidth. Long wavelength normal modes appear for the Jo and Jx parameters, explaining the greater red-shifting of the predictions and potentially suggesting more minimization is needed. When comparing the outer membrane proteins, CDCALC performs better with OPCA than G. This difference may be because the crystal structure of outer membrane protein G is not as well resolved (2.30 Å [62]) as the crystal structure for outer membrane protein OPCA (1.95 Å [61]). Furthermore, outer membrane protein G is larger (281 residues compared to the 253 residues of OPCA) making it more challenging a prediction.

### 2.4. α/β Proteins

When the DInaMo method succeeds, the general morphology of the predicted CD spectra agrees with experiment in the π-π* region (Figure 5, Figures S47–S55). CDCALC succeeds with all four proteins, but CAPPS only succeeds with two.

The smallest and largest RMSDs (Table 3, Tables S18–S21) for CDCALC predictions in this category occur for crambin: 4000 OL, 0.776 M^−1^·cm^−1^ and 6000 Jo, 7.515 M^−1^·cm^−1^. All other RMSDs for all four proteins fall within this range. The original parameters seem to produce the lowest RMSDs the most frequently (monellin 4000 OL, triose phosphate isomerase 6000 OL, and crambin 4000 OL), but helical 4000 Hx perform best with ferredoxin. Thus, when working with CDCALC, the original parameters (as they did for the α-helical proteins), seem to be the best first choice when working with energy minimized proteins. The only difference is a bandwidth of 4000 cm^−1^ might be a better choice than the 6000 cm^−1^, the choice recommended for purely α-helical proteins. As seen with all previous categories, the poly-l-proline parameters red-shift the predicted spectra. Helical parameters occasionally blue-shift predicted spectra (monellin and ferredoxin).

**Figure 5:**
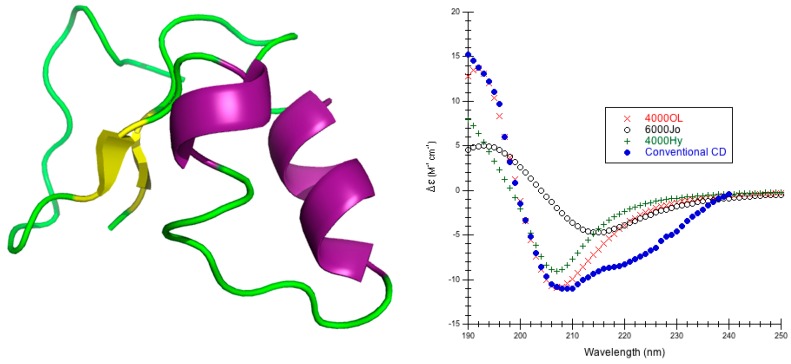
Crambin. (**Left**) Secondary structure of crambin (PDB code 1AB1 structure [65]): thick purple cartoons/coils correspond to α-helices (12–18, 27–30), the yellow tapes are β-sheets, (2–3, 33–34), and the thin green ropes are turns and other structures; (**Right**) Predicted CD Using CDCALC. The 6000 and 4000 refer to bandwidths in cm^−1^. Calculated spectrum show the smallest RMSD 4000OL (×), the largest RMSD 6000Jo (o), and an example helical parameter result, 4000Hy (+). The blue dots (•) are the experimental SRCD (CD0000046000) [44,47]. CATH classifies the secondary structure as alpha-beta/2-layer sandwich [53].

CAPPS fails for 50% of the α/β protein tested (monellin and ferredoxin). It succeeds in predicting CD for triose phosphate isomerase and crambin (Tables S20–S21). Helical parameters perform better with CAPPS since poly-l-proline II parameters red-shift predicted spectra (Table 2, Figure 5, Tables S20–S21, Figures S53, S55). Although the lowest RMSD for triose phosphate isomerase with CAPPS is 6000 Ho, 2.073 M^−1^·cm^−1^, it is the helical parameters with a bandwidth of 4000 cm^−1^ that reproduce the peak at 190 nm the best, but the bandwidth of 6000 cm^−1^ better resembles the slope as the CD crosses zero into the negative peak. For crambin, CAPPS helical predictions are similar to CDCALC, but are just a little less intense, and the poly-l-proline II parameters do not red-shift spectra as much as seen with CDCALC. Herein, one representative protein, crambin, is detailed.

**Table 3:** CD Analysis of α/β proteins. The DInaMo calculations are for the minimized or rebuilt structure using CDCALC or CAPPS. All RMSDs are calculated between 180 and 210 nm.

CD Method	Wavelength (nm)	Δε (M^−1^·cm^−1^)	Wavelength (nm)	Δε (M^−1^·cm^−1^)	RMSD (M^−1^·cm^−1^)	Range RMSDs † (M^−1^·cm^−1^)
**Monellin (Figure S47)**						
^a^ SRCD (CD0000046000) [47]	190	3.75	213	−3.32	0.000	
^b^ 4000OL (PDB code 1MOL)	191	4.37	212	−2.08	0.876	0.876–2.234
^c^ SII (PDB code 1MOL)	189	3.73	217	−0.96	1.501	1.501–3.938
**Ferredoxin (Figure S49)**						
^a^ SRCD (CD0000032000) [47]	185	1.03	201	−6.37	0.000	
^b^ 4000OL (PDB code 2FDN)	189	6.66	205	−5.19	4.627	1.388–5.076
^d^ MM2 (PDB code 2FDN)	194	3.99	214	−1.45	5.539	5.539–6.791
**Triose Phosphate Isomerase (Figure S51)**						
^a^ SRCD (CD0000070000) [47]	190	7.85	217	−5.06	0.000	
^b^ 4000OL (PDB code 7TIM)	192	10.70	207	−8.80	3.037	1.840–3.768
^e^ 4000Hx (PDB code 7TIM)	190	8.66	204	−6.14	2.522	2.073–3.437
^f^ MM3 (PDB code 7TIM)	192	7.54	211	−4.90	1.230	1.230–2.193
**Crambin (Figure 5)**						
^g^ Conventional CD [63]	191	15.26	209	−10.98	0.000	
^b^ 4000OL (PDB code 1AB1)	192	13.66	207	−10.93	0.776	0.776–7.515
^e^ 4000Hx (PDB code 1AB1)	192	8.14	206	−7.94	3.897	3.897–7.876

The DInaMo calculations are for the minimized or rebuilt structure using CDCALC or CAPPS. Example literature calculations are also listed when available. **†** The range of RMSDs if for all calculations including literature calculations is presented. For full RMSD information on all calculations including literature, please see the Supplementary Information for a full table of calculations with RMSDs for each protein. ^a^ SRCD from the PCDDB [44]; ^b^ CDCALC using PDB structure minimized via NAMD/CHARMM22; ^c^ Exciton Hamiltonian with electrostatic fluctuations based on 2000 MD snapshots that consider the electrostatic potential from all surroundings [29]; ^d^ Matrix method using including protein backbone and charge-transfer transitions [55]; ^e^ CAPPS using rebuilt secondary structures of PDB structure including hydrogens; ^f^ Matrix method on 7TIM [64] using *ab intio* parameters including protein backbone, charge-transfer and side chain transitions [55]; ^g^ Conventional CD for crambin in 60% ethanol [63].

Crambin: PDB code 1AB1 [65] (Figure 5) is a small hydrophobic plant seed protein that exhibits sequence homology to membrane-active plant toxins, but its function is unknown [63]. Crambin has only 46 amino acids and has been crystallized to very high resolution (e.g., 1AB1 has a resolution of 0.89 Å) [65]. The conventional CD spectrum in 60% ethanol shows secondary structure very similar to that of crystals: 36% helix, 23% sheet, 18% turn and 23% irregular [63]. The conventional CD spectrum in various environments: ethanol, methanol, trifluoroethanol and in small unilamellar DMPC vesicles yield similar secondary structures: 31%–38% α-helix, 29%–37% and β-sheet plus β-turn [66]. CATH classifies the secondary structure as alpha-beta/2-layer sandwich [53].

This is a first attempt to predict the far-UV CD for crambin. Both DInaMo methods CDCALC and CAPPS succeed in simulating spectra (Table 3, Supplementary Information Table S21, Figures S54 and S55). The best predictions occur with CDCALC and the same kinds of parameters (4000 OL). In general, the 4000 cm^−1^ bandwidth does a better job with intensities than the 6000 cm^−1^ bandwidth in all cases. Helical parameters locate peaks better while poly-l-proline II parameters red-shift predicted spectra. The original parameters (4000 OL) yield the smallest RMSD of 0.776 M^−1^·cm^−1^ using CDCALC (Figure 5). The largest RMSD occurs with CAPPS 6000 Jo 7.515 M^−1^·cm^−1^. Comparing CDCALC with CAPPS, CDCALC generally does better; *i.e.*, the best CAPPS prediction yields a larger RMSD (4000 Hx, 3.897 M^−1^·cm^−1^) than the best for CDCALC.

### 2.5. Other

This category includes proteins that either CATH [53] did not classify (e.g., insulin) or CATH classified as irregular (e.g., bovine pancreatic trypsin inhibitor and chain A of the light harvesting complex II). No single set of parameters work well for all the proteins in this group.

Insulin is the only protein studied where the poly-l-proline II parameters yield the best predictions with both CDCALC and CAPPS (Table 4, Table S22, Figures S56–S59). This is in spite of the secondary structure including three short α-helices and two even shorter 3_10_ helices (Figure S56). Curiously, the helical parameters consistently blue-shift spectra for insulin and the poly-l-proline parameters locate the peaks well (*i.e.*, not red-shifted as seen for all other proteins). Literature calculations using the matrix method including peptide, side chain and charge-transfer transitions predict RMSDs in the π-π* region nearly as low as the best of the DInaMo calculations (2.072 M^−1^·cm^−1^ for MM3 [55], 0.945 M^−1^·cm^−1^ for CDCALC 6000 Jy and 1.061 M^−1^·cm^−1^ for CAPPS 6000 Jy) (Table 4).

The helical parameters in DInaMo perform best for bovine pancreatic trypsin inhibitor (aka aprotinin) (Table 4, Supplementary Information Table S23, Figures S60–S62). There is only one short α-helix and one even shorter 3_10_ helix in aprotinin (Supplementary Information Figure S60). CDCALC does the better job of reproducing the CD spectrum than CAPPS because the helical parameters locate the peaks best with CDCALC. CAPPS helical parameters yield red-shifted spectra that are weaker than CDCALC predictions. Both CDCALC and CAPPS have the poly-l-proline II parameters predicting red-shifted spectra as commonly observed for many other proteins. The original parameters with CDCALC yield spectra that are similar to the helical parameters, but the spectra are more red-shifted. The details of light harvest protein complex II follow as the last example in this category.

**Figure 6:**
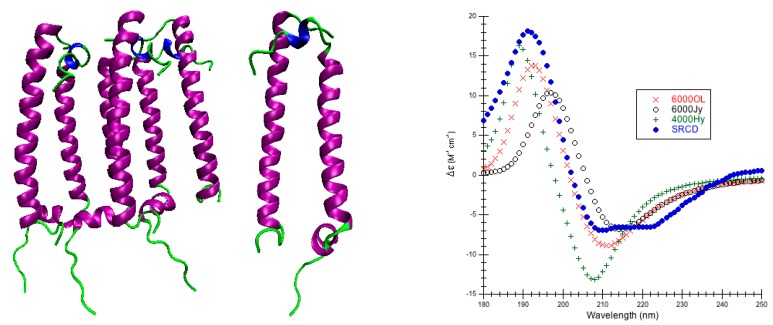
Light-Harvesting Protein Complex II. (**Left** & **Center**) Secondary structure of light-harvesting protein complex II (PDB code 1NKZ [67]). The purple coils are helices (12–37, 40–46); the 3_10_-helices are blue (6–8). The green coils are other structures. (**Left**) Asymmetric unit (A_3_B_3_); (**Center**) Heterodimer (AB). (**Right**) Predicted CD Using CDCALC. The 1NKZ AB dimer is minimized with 5000 conjugate gradient steps using NAMD/CHARMM22. Calculated spectra ignore all CH_3_ group hydrogens. The 6000 and 4000 refer to bandwidths in cm^−1^. Calculated spectrum show the smallest RMSD 6000 OL (×), the largest RMSD 4000Jy (o), and an example helical parameter result, 4000 Hy (+). The blue dots (•) are the experimental SRCD (CD0000114000) [44,59]. The CATH fold classification [53] is a combination of few secondary structures/irregular for chain A and mainly alpha/up-down bundle for chain B. Note: the complete hexameric asymmetric unit of the protein was not treated and neither were the any of the ligands (bacteriochlorophyll A, benzamidine, β-octylgucoside, rhodopin glucoside).

Light-Harvesting Protein Complex II: PDB code 1NKZ [67], an integral membrane protein from *Rhodopseudomonas acidophila* that participates in the first stages of photosynthesis, is a multimer of 18 subunits or nonamer of a dimer with an α- and a β-chain (Figure 6). The α-chain contains 53 residues and is classified by CATH as having few secondary structures and irregular architecture [53]. The β-chain contains 41 residues and is classified by CATH as mainly alpha/up-down bundle [53]. The PCDDB classifies 1NKZ as 69.1% α-helix, 3.2% 3_10_-helix, 5.3% bonded turn, 4.3% bend, and 18.1% irregular [44].

Herein, DInaMo makes a first attempt to simulate the far-UV CD of light-harvesting protein complex II using the heterodimer. Both CDCALC and CAPPS succeed in making predictions (78, Table 4, Table S24, Figures S63 and S64). Although RMSDs are fairly large, CDCALC yields the smallest RMSD with the original parameters and a bandwidth of 6000 cm^−1^ (6000 OL, 4.503 M^−1^·cm^−1^). CAPPS smallest RMSD is using the helical parameters and a bandwidth of 6000 cm^−1^ (6000 Ho, 6.349 M^−1^·cm^−1^). With CDCALC the helical parameters slightly blue-shift predicted CD, and the poly-l-proline II parameters red-shift predicted CD. 

**Table 4:** CD Analysis of Other Proteins. The DInaMo calculations are for the minimized or rebuilt structure using CDCALC or CAPPS. All RMSDs are calculated between 180 and 210 nm.

CD Method	Wavelength (nm)	Δε (M^−1^·cm^−1^)	Wavelength (nm)	Δε (M^−1^·cm^−1^)	RMSD (M^−1^·cm^−1^)	Range RMSDs† (M^−1^·cm^−1^)
**Insulin (Figure S56)**						
^a^ SRCD (CD0000040000) [47]	192	16.75	221	−8.08	0.000	
^b^ 6000OL (PDB code 3INC)	192	11.08	210	−4.68	3.253	0.945–7.731
^c^ 6000Jy (PDB code 3INC)	195	8.59	2.10	−5.85	1.129	1.129–9.930
^d^ 6000Jy (PDB code 3INC)	196	7.08	212	−4.46	1.061	1.061–9.018
^e^ MM3 (PDB code 1TRZ)	192	7.59	210	−4.45	2.072	2.072–3.639
**Bovine Pancreatic Trypsin Inhibitor (Figure S60)**						
^a^ SRCD (CD0000007000) [47]	187	4.52	202	−7.67	0.000	
^b^ 6000OL (PDB code 5PTI)	189	3.86	207	−3.42	3.056	1.669–4.954
^d^ 6000Jy (PDB code 5PTI)	196	1.14	210	−2.24	4.352	3.634–4.687
^f^ RH04:3 (PDB code 5PTI)	187	6.72	205	−6.48	1.629	1.629–7.100
**Light-Harvesting Protein Complex II (Figure 6)**						
^a^ SRCD (CD0000114000) [59]	191	18.12	210	−6.97	0.000	
^b^ 6000OL (PDB code 1NKZ)	192	13.81	211	−8.90	4.503	4.503–10.390
^d^ 6000Jy (PDB code 1NKZ)	196	9.98	214	−13.83	7.054	6.349–10.537

The DInaMo calculations are for the minimized or rebuilt structure using CDCALC or CAPPS. Example literature calculations are also listed when available. **†** The range of RMSDs if for all calculations including literature calculations is presented. For full RMSD information on all calculations including literature, please see the Supplementary Information for a full table of calculations with RMSDs for each protein. ^a^ SRCD from the PCDDB [44]; ^b^ CDCALC using PDB structure minimized via NAMD/CHARMM22; ^c^ CDALC using PDB structure minimized via Insight^®^II/Discover/CVFF; ^d^ CAPPS with rebuilt secondary structures of the PDB structure including all hydrogens; ^e^ Matrix method including *ab initio* protein backbone, charge-transfer and side chain transitions [55]; ^f^ Matrix method on including *ab initio* protein backbone and *ab initio* side chain parameters [68].

CDCALC best approximates the intensity at 191 nm with a bandwidth of 4000 cm^−1^ and the intensity at 210 nm with a bandwidth of 6000 cm^−1^. The original parameters locate both peaks best with CDCALC, but the bandwidth of 4000 cm^−1^ yields band peaks that are too intense. CAPPS on the other hand, locates peaks best using the helical parameters, but again the poly-l-proline II parameters yield red-shifted CD predictions. With CAPPS, the bandwidth of 6000 cm^−1^ does a better job of approximating intensity, but the positive peak prediction is too weak while the negative peak prediction is too strong. Considering that only the dimer of this complex multimeric membrane protein is considered (including the energy minimization in vacuum), DInaMo has made a reasonable first approximation for the far-UV CD spectrum.

### 2.6. Spearman Rank Correlation Coefficient

**Figure 7:**
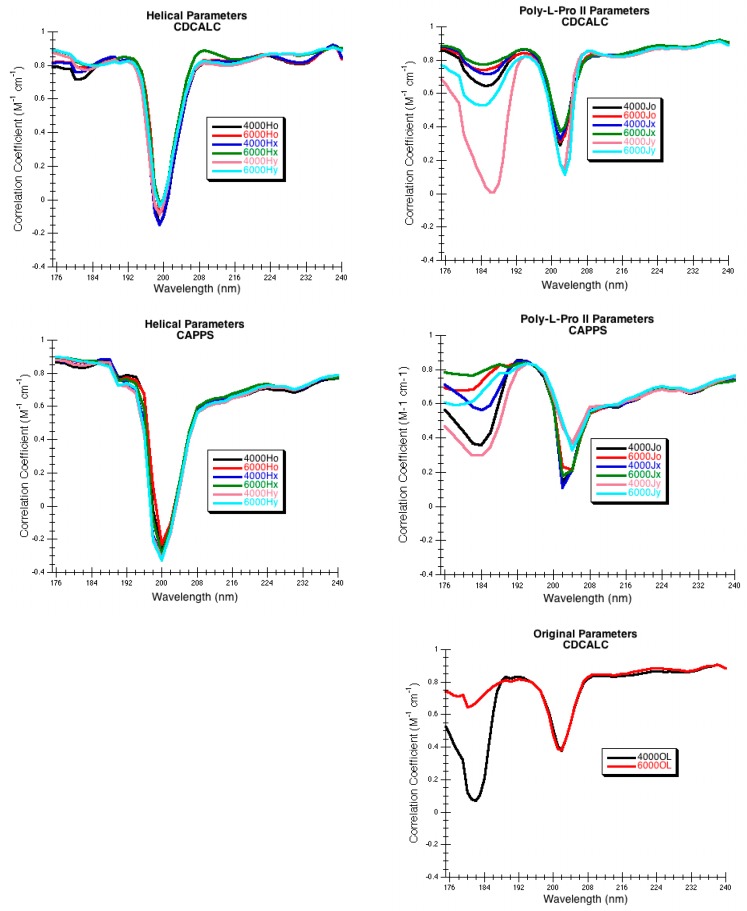
Spearman Rank Correlation Coefficients for DInaMo Calculations. CDCALC on 24 proteins. CAPPS on 17 proteins.

DInaMo can reproduce the general morphology of the far-UV CD of a variety of proteins. It also reproduces the majority of the maxima and minima in the π-π* region of the spectrum. When examining the Spearman rank correlation, the greatest errors in predictions occur when CD spectra cross zero (around 200 nm) (Figure 7). The helical parameters have the greatest error in the zero-crossing, while the original parameters have the least error in the zero-crossing. The poly-l-proline II and original parameters also show significant errors in the region below 190 nm, particularly for the narrower bandwidth of 4000 cm^−1^. The helical parameters perform much better in this region. With CAPPS the zero-crossing error is greater with the helical parameters, but these parameters do better in the region below 190 nm. Greater errors are seen in this region using the poly-l-proline II parameters with CAPPS as was seen with CDCALC.

CDCALC appears more dependable than CAPPS because CAPPS has a tendency to fail for larger proteins with extensive β-sheet structures. The problem with CAPPS occurs in the rebuilding process; multiple atomic collisions occur during the rebuild so that more than 50% of the protein would need to be ignored before the calculation will run. Furthermore, the Spearman rank correlation also shows CDCALC to be more dependable, particularly in the regions around 190 nm and above 208 nm. Using molecular mechanics to energy minimize the protein instead of rebuilding it, does prove successful when using CDCALC. Ignoring hydrogens on CH_3_ groups using CDCALC is a reasonable first approximation that eliminates excessive sensitivity to structure and the issue of close contacts found in CAPPS.

Considering the individual parameters sets used, no one set appears to be superior consistently, and all have proved to be useful at least once. Examining the Spearman rank correlation at a handful of wavelengths suggests that the Hx parameters at a bandwidth of 6000 cm^−1^ might be the best choice (Table 5), but other parameters do better in the region where the spectra cross zero (Figure 7). Guidelines for parameter use are better chosen based on the fold class of the protein, which will be provided in the Conclusions section of this paper.

**Table 5:** Spearman Rank Correlation Coefficients for Calculated Far-UV CD.

		Correlation Coefficient			
Method/# Proteins	Parameters	175 nm	190 nm	208 nm	220 nm
DInaMo/CDCALC	4000 Ho	0.79	0.82	0.80	0.84
24 proteins	6000 Ho	0.82	0.82	0.81	0.85
	4000 Hx	0.81	0.83	0.81	0.85
	6000 Hx	0.89	0.84	0.88	0.85
	4000 Hy	0.88	0.82	0.80	0.83
	6000 Hy	0.89	0.81	0.82	0.84
	4000 Jo	0.86	0.78	0.82	0.85
	6000 Jo	0.87	0.80	0.82	0.86
	4000 Jx	0.88	0.81	0.81	0.86
	6000 Jx	0.89	0.82	0.81	0.87
	4000 Jy	0.68	0.46	0.85	0.84
	6000 Jy	0.77	0.73	0.86	0.85
	4000 OL	0.52	0.82	0.82	0.85
	6000 OL	0.74	0.80	0.83	0.87
DInaMo/CAPPS	4000 Ho	0.87 ^a^	0.77	0.56	0.70
17 proteins	6000 Ho	0.89 ^a^	0.76	0.59	0.71
	4000 Hx	0.88 ^a^	0.77	0.59	0.70
	6000 Hx	0.90 ^a^	0.76	0.60	0.71
	4000 Hy	0.88 ^a^	0.73	0.56	0.68
	6000 Hy	0.90 ^a^	0.72	0.57	0.69
	4000 Jo	0.57 ^a^	0.80	0.56	0.65
	6000 Jo	0.69 ^a^	0.82	0.54	0.66
	4000 Jx	0.71 ^a^	0.80	0.56	0.65
	6000 Jx	0.78 ^a^	0.81	0.55	0.66
DInaMo/CAPPS	4000 Jy	0.47 ^a^	0.68	0.58	0.65
17 proteins	6000 Jy	0.61 ^a^	0.78	0.56	0.67
Matrix Method [25] 71 proteins	peptide backbone + side chain + charge-transfer	0.79	0.75	NA	0.88 ^b^
Dipole Interaction Model [3,6] 15 proteins	6000 Hy	NA	0.89	0.75	0.74
Matrix Method [6,12] 23 proteins	semiempirical	NA	0.69	0.72	0.86
Matrix Method [6,12] 47 proteins	semiempirical	NA	0.68	0.67	0.93
Matrix Method [6,69]15 proteins	ab initio	NA	0.87	0.71	0.96
Matrix Method [6,69] 23 proteins	ab initio	NA	0.81	0.73	0.89
Matrix Method [6,69] 29 proteins	ab initio	NA	0.84	0.73	0.90
Matrix Method [6,69] 47 proteins	ab initio	NA	0.86	0.80	0.94

^a^ At 176 nm; ^b^ At 222 nm. Grey highlight represents the best Spearman rank correlation for a set of calculations.

### 2.7. Comparison of DInaMo to the Matrix Method

The dipole interaction model, and DInaMo/CDCALC in particular, does a good job of approximating the π-π* transition region of the far-UV CD spectrum particularly when considering the Spearman rank correlation (Table 5, Figure 7). DInaMo does better in this region than a variety of matrix method calculations [6,12,25,69]. Specifically, only one matrix method simulation yields a greater Spearman rank correlation at 190 nm than CDCALC and that one used ab initio parameters of the amide π-π* and n-π* transitions [69]; furthermore, the difference between this matrix method calculation and CDCALC in Spearman rank correlation is small (0.02). The only literature method that yields a better Spearman rank correlation better than CDCALC at 190 nm is the original work of Bode and Applequist [3] that also uses the dipole interaction model and the difference with the CDCALC results may not be statistically significant (0.01). At 208 nm, CDCALC consistently yields the best Spearman rank correlations. Of course, DInaMo (both CDCALC and CAPPS) do not compete with the matrix method in the region of the n-π* transition (around 220 nm) because this transition is not included in DInaMo. The matrix methods do better because they include the n-π* transition [6,12,25,69]. What is surprising is that using energy-minimized structures seems to improve the DInaMo predictions in this region of the spectrum compared to rebuilding as done with CAPPS and the literature dipole interaction model calculations [3].

## 3. Experimental Section

**Figure 8:**
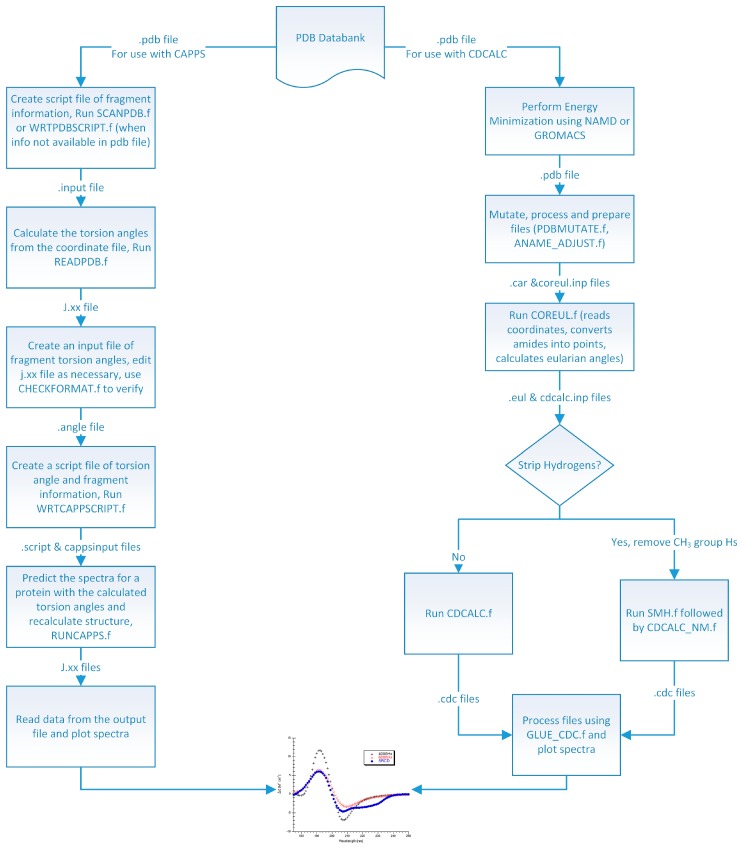
Flow Diagram of the DInaMo Package. Note, the CD spectrum, like the one pictured at the bottom of this diagram, can only be displayed using a common graphing program such as Origin or Kaleidagraph.

High quality structures were needed to predict circular dichroism for each protein so considerable effort was spent in preparing the model structures used (Figure 8). In the DInaMo package the user has a choice to either use molecular mechanics to add hydrogens and minimize the structure or extract the internal coordinates and rebuild the protein’s secondary structural components (including hydrogens) using idealized bond lengths and angles. Currently, DInaMo treats only aliphatic amino acids (alanine, valine, proline, glycine, leucine, and isoleucine) in their entirety; all other amino acids are mutated. Typically, alanine is chosen because it can be initially approximated from the current side chain and will not introduce strain into the backbone. Alternatively, the protein structure can also be rebuilt to account for only the secondary structure fragments using the CAPPS route (Figure 8). This automatically mutates any amino acid residues that are not currently treated to alanine before optimizing and reconstructing the structure. The molecular mechanics route (CDCALC, Figure 8) requires significant energy minimization to adjust bond lengths, bond angles, and to average the positions of the hydrogen atoms that needed to be added; it is common for crystal structure geometries to have slightly short bond lengths (e.g., see Carlson *et al.* 2005 as an example [70]) so that they cannot be used directly with the dipole interaction model. Furthermore, the dipole interaction model is sensitive to small changes in structure [9,70,71,72]. Energy minimization is followed by mutation of the nonaliphatic residues and another brief minimization to relax any atomic clashes, when minimized with Insight^®^II; these minimizations do not lead to changes in secondary structures, but impact highly flexible regions. It is the initial minimization that changes the flexible regions the most and not the post mutation minimization. When performed in NAMD, only one minimization was necessary.

Protein databank (PDB) [73] files of the protein structures used (Table 6) provide initial structures for the calculations. Hydrogen atoms were added to each protein structure as needed because they are required for the CD calculation. The particular PDB files were chosen for two reasons: (1) Each was a high-resolution structure with a R factor of less than 2.50 Å; (2) The structures chosen were the same species for which synchrotron radiation circular dichroism (SRCD) was available in the Protein Circular Dichroism Data Bank (PCDDB) [44]. The only exception was crambin, for which only conventional CD was available [63], but very high resolution crystal structures were available [65]

**Table 6:** PDB Structures and Literature CD Used.

Protein Name	PDB Code	Resolution (Å)	CATH Fold [57]	PCDDB Code
Avidin	2A8G [74]	1.99	mainly β	CD0000008000 [47]
Bacteriorhodopsin	1QHJ [75]	1.90	mainly α	CD0000101000 [59]
Bovine pancreatic trypsin inhibitor	5PTI [76]	1.00	irregular	CD0000007000 [47]
Calmodulin	1LIN [77]	2.00	mainly α	CD0000013000 [47]
Crambin	1AB1 [65]	0.89	α/β	Not applicable/[63]
Concanavalin A	1NLS [78]	0.94	mainly β	CD0000020000 [47]
Cytochrome c	1HRC [79]	1.90	mainly α	CD0000021000 [47]
Ferredoxin	2FDN [80]	0.94	α/β	CD0000032000 [47]
Insulin	3INC [81]	1.85	not classified	CD0000040000 [47]
Jacalin	1KU8 [82]	1.75	mainly β	CD0000041000 [47]
Lectin (lentil)	1LES [83]	1.90	mainly β	CD0000043000 [47]
Lectin (pea)	1OFS [73]	1.80	mainly β	CD0000053000 [47]
Leptin	1AX8 [84]	2.40	mainly α	CD0000044000 [47]
Light Harvesting Complex II	1NKZ [67]	2.00	irregular/mainly α	CD0000114000 [59]
Lysozyme	2VB1 [56]	0.65	mainly α	CD0000045000 [47]
Myoglobin (horse)	3LR7 [85] 2V1K [86]	1.25	mainly α	CD0000047000 [47]
Myoglobin (sperm whale)	2JHO [87]	1.40	mainly α	CD0000048000 [47]
Monellin	1MOL [88]	1.70	α/β	CD0000046000 [47]
Outer Membrane Protein G	2IWV [62]	2.30	mainly β	CD0000118000 [59]
Outer Membrane Protein OPCA	2VDF [61]	1.95	mainly β	CD0000119000 [59]
Phospholipase A2	1UNE [89]	1.50	mainly α	CD0000059000 [47]
Rhomboid peptidase	2NR9 [58]	2.20	mainly α	CD0000109000 [59]
Rubredoxin	1R0I [90]	1.50	mainlyβ	CD0000064000 [47]
Triose phosphate isomerase	7TIM [64]	1.90	α/β	CD0000070000 [47]

### 3.1. Energy Minimization (for use with CDCALC)

Each protein structure was minimized either with the Discover module of Insight^®^II (San Diego, CA, USA) or with NAMD [91]. Minimization was necessary to tweak the internal coordinates so that the structures could be used with the dipole interaction model. No major secondary structure elements were changed during the minimizations.

#### 3.1.1. NAMD

Each protein was minimized in vacuum via the conjugate gradient method. The minimization was performed using the CHARMM22 [92,93] force field in NAMD [91] for either 5000 or 10,000 steps. Larger proteins or lower resolution structures needed the larger number of steps for minimization. The structure at the last step of the minimization was used for CD predictions since no convergence criterion was required.

#### 3.1.2. Insight^®^II/Discover

Using the force field CVFF (Consistent-Valence Force Field) [94] within the Discover module of Insight^®^II (San Diego, CA, USA) has proven highly successful with small peptide structures used with the dipole interaction model [39,70,72] so that it was also applied to the proteins insulin, lysozyme, two species of myoglobin. Because solvent effects were not the object of this study, it was not deemed necessary to explicitly include the solvent in the Discover minimizations. A strategy of steepest descents followed by conjugate gradients was performed for the different proteins where the number of minimization steps varied for each protein. A large number of steps of steepest descents were chosen first to stay in a local minimum, followed by a short number of steps using conjugate gradients to just tweak the local minimum. For example, 900,000 steps of steepest descents and 100,000 steps of conjugate gradient were performed for lysozyme. The two myoglobins were minimized with 110,000 steps of steepest descents and 21,000 steps of conjugate gradients. Insulin only needed 1000 steps of steepest descents and 100 steps of conjugate gradients. These many iterations were needed to fine-tune the structure enough for use in circular dichroism (CD) calculations that included hydrogens. A final maximum derivative convergence criterion 0.001 was met for all minimizations upon completion of the conjugate gradients minimization.

### 3.2. CD Calculation

#### 3.2.1. CDCALC

Cartesian coordinates generated within Insight^®^II or NAMD were used to calculate the π-π* amide transitions of the protein using the dipole interaction model (DInaMo) [31,32,35]. With this method, coordinates for the nonchromophoric atoms of the protein were treated directly, while the chromophoric amides were reduced to a single point located along the C-N bond of the amide. For the structures generated via NAMD, the hydrogens on CH_3_ groups were deleted; the minimizations with Insight^®^II included these hydrogens as isotropic polarizabilities. All secondary structure types including α-helices, β-sheets, turns, poly-l-proline, and irregular structures are included in the calculation, so that no secondary structure is ignored. This is a major difference between CDCALC and CAPPS (see 3.3.2 CAPPS). The amide point position for the anisotropic chromophore was either the center of the N-C bond (o), shifted along the N–C bond 0.1 Å towards the carbonyl carbon (x), or shifted 0.1 Å normal to the C–N bond from the center into the NCO plane toward the carbonyl O (y). The Eulerian angles between the first amide chromophore and successive ones were calculated (COR_EUL in Figure 8). The CDCALC portion of the program generated the normal modes and spectrum for each protein. Three different dispersive parameters were tested: the original parameters created for the dipole interaction model (OL) [95], the α-helical parameters created for proteins (H) [36], and the poly-l-proline II parameters (J) [36]. The CD spectrum using CDCALC of each protein was computed between 175 and 250 nm with a step size of 1 nm with bandwidths of either 4,000 or 6000 cm^−1^. CDCALC for each protein was run on a Linux server (Fedora Core Linux 6, 64-bit) and was compiled using PGI FORTRAN 77 compiler.

#### 3.2.2. CAPPS

CAPPS functioned by breaking the PDB structure into secondary structural elements of α-helices and β-sheets and rebuilding them using idealized bond lengths and bond angles; torsion angles were retained from the PDB structure. Other parts of the protein structure were ignored. For example, lysozyme had two partial turn structures that were ignored (Table 7). For sperm whale myoglobin, one undefined secondary structure with residues 1–2 (VL), and one kink in a helix with residues 35–37 (GHP) were ignored (Table 7). If more than 50% of the protein needed to be ignored because of close contacts occurring during the rebuild, then CAPPS was considered a failure for that protein. Like CDCALC, once CAPPS identified the secondary structures and rebuilt them, coordinates for the nonchromophoric atoms including all hydrogens were treated directly, while the chromophoric amides were reduced to a single point located along the C–N bond of the amide. The amide point position was either the center of the N–C bond (o), shifted 0.1 Å towards the carbonyl carbon (x), or shifted 0.1 Å normal to the C–N bond, toward the carbonyl O (y). The Eulerian angles between the first amide chromophore and successive ones were calculated, normal modes were generated, and the spectrum predicted. Only the helical (H) and poly-l-proline II (J) parameters were tested as recommended by Bode and Applequist [3]. The CD was computed between 176 and 250 nm with a step size of 2 nm with bandwidths of either 4000 or 6000 cm^−1^. CAPPS for each protein was run on a Linux cluster that has 28 compute nodes, each of which has a dual 64-bit, 4-core Opteron processor and 16 GB of RAM.

### 3.3. CD Analysis

The results from the CD calculations were analyzed using Excel (Microsoft, Santa Rosa, CA, USA) and plotted with either Excel, OriginPro™ 7.5 (OriginLab Corporations, Northampton, MA, USA), or KaleidaGraph (Synergy Software, Reading, PA, USA). Published CD spectra were compared with the calculated values for each molecule. Further quantitative analysis was done by evaluating the normalized root mean square deviation (RMSD) between experiments and calculated at each wavelength for the total number of wavelengths *n*_λ_ computed.

(14) $$RMSD=\sqrt{\frac{{\left(Experimental\;CD\left(\lambda_{i}\right)-Calculated\left(\lambda_{i}\right)\right)}^{2}}{n_{\lambda }}}$$

## 4. Conclusions

Because the dipole interaction model is very sensitive to molecular geometry, it is crucial to optimize any protein structure either by energy minimization or rebuilding the secondary structure based on the torsions extracted from the PDB file. Current calculations suggest that energy minimization is an excellent choice for dealing with the geometric sensitivity and will less likely lead to failure than the rebuilding method, particularly if there are a significant number of β-sheets in the proteins.

The choice of parameters for use with DInaMo depends on the fold and algorithm used. The best choice of parameters for mainly alpha proteins using CDCALC is 6000 OL and using CAPPS is 6000 Ho. The best choice of parameters for mainly beta proteins is the 4000 Hy with both CDCALC and CAPPS. Alpha/beta proteins are best treated with 4000 OL using CDCALC and 4000 Hx using CAPPS. Other kinds of structures, especially irregular ones, are best treated with 6000 Jy. For any unusual or new folds, the user should continue to test all parameters sets when performing CD calculations, and this includes testing different bandwidths. Bandwidths around 4000 to 6000 cm^−1^ are recommended for calculations to approximate the experiment far-UV CD spectrum of proteins.

DInaMo/CDCALC is an excellent choice for simulating the far-UV CD in the π-π* region. Using energy-minimized structures ignoring the hydrogens on CH_3_ groups is the best current choice with DInaMo. More minimization is better than less, but 5000 conjugate gradient steps seems sufficient for small proteins with 150 amino acids or fewer, and 10,000 steps work better for 150–300 amino acids. For proteins larger than 300 amino acids, it is recommended to break the structure down into pieces 300 amino acids or fewer as long as no major secondary structures are disrupted [96] and then use CDCALC, but be sure to minimize the intact protein first.

Because the removal of the hydrogens on CH_3_ groups is successful, removing more Hs (e.g., from CH_2_ or CH groups) is being explored. Furthermore, creating new isotropic polarizability parameters for CH_3_, CH_2_ and CH groups that treat the points as mean polarizabilities is also being explored. Plans to add and optimize parameters for the n-π* transition are also beginning.

The code for DInaMo is available upon request from the corresponding author, Kathryn. A. Thomasson at University of North Dakota, Chemistry Department, 151 Cornell St. Stop 9024, Grand Forks, ND 58202, USA.

**Table 7:** PDB Structures Computed Using CAPPS and Fragments Ignored.

Protein Name	PDB Code	Fragments Ignored
Avidin	2A8G [74]	Turn (54A-54A), Turn (60A-62A), Turn (112A-112A)
Bacteriorhodopsin	1QHJ [75]	Turn (5A-5A), Turn (33A-36A), Turn (101A-104A), Turn (128A-130A), Turn (161A-164A)
Bovine pancreatic trypsin inhibitor	5PTI [76]	Turn (1A-1A), Turn (46A-46A), Turn (57A-58A), Sheet (45A-45A)
Calmodulin	1LIN [77]	Turn (3A-5A), Turn (27A-28A), Turn (100A-101A), Turn (146A-148A)
Crambin	1AB1 [65]	Turn (1A-2A), Sheet (32A-34A)
Cytochrome c	1HRC [79]	Turn (1A-1A), Turn (15A-48A), Turn (69A-69A), Helix (2A-14A)
Concanavalin A	1NLS [78]	Coil (1A-3A), Coil (11A-13A), Coil (79A), Coil (150A-152A), Coil (153A-155A)
Ferredoxin	2FDN [80]	CAPPS FAILED
Insulin	3INC [81]	C-terminus (21A), N-terminus (1B-7B), Turn (21B-23B), Helix (18A-20A), Sheet (24B-26B)
Jacalin	1KU8 [82]	CAPPS FAILED
Lentil Lectin	1LES [83]	Turn (1A-1A), Helix (98A-100A), Turn (62A-69A), Turn (180A-182A), Turn (190A-192A)
Pea Lectin	1OFS [73]	CAPPS FAILED
Leptin	1AX8 [84]	Turn (3A-3A), Turn (24A-50A), Turn (residues 68A-70A), Turn (144A-146A)
Light Harvesting Complex II	1NKZ [67]	Turn (2A-4A), Turn (10A-10A)
Lysozyme	2VB1 [56]	Turn (1A-3A), Turn (116A-118A), Sheet (43A-45A), Sheet (51A-53A)
Myoglobin (horse)	3LR7 [85] 2V1K [86]	Turn (1A-2A), Turn (21A-19A), Turn (59A-57A), Turn (97A-99A), Turn (151A-153A)
Myoglobin (sperm whale)	2JHO [87]	Turn (1A-2A), Turn (19A-19A), Turn (37A-35A), Turn (97A-99A)
Monellin	1MOL [88]	CAPPS FAILED
Outer Membrane Protein G	2IWV [62]	CAPPS FAILED
Outer Membrane Protein OPCA	2VDF [61]	CAPPS FAILED
Phospholipase A2	1UNE [89]	Turn (1A-1A), Turn (58A-58A), Helix (18A-21A), Helix (113A-115A)
Rhomboid peptidase	2NR9 [58]	Turn (29A-29A), Turn (40A-42A), Turn (86A-84A), Turn (193A-195A)
Rubredoxin	1R0I [90]	Turn (1A-3A), Turn (48A-48A), Sheet (4A-6A), Helix (45A-47A)
Triose phosphate isomerase	7TIM [64]	Turn (2A-4A), Turn (87A-89A), Turn (119A-121A), Turn (128A-130A), Turn (136A-138A), Turn (237A-237A)

## Supplementary Materials

Supplementary materials can be found at http://www.mdpi.com/1422-0067/16/09/21237/s1.
