# Supplementary material for: Introducing DInaMo: A Package for Calculating Protein Circular Dichroism Using Classical Electromagnetic Theory
Source: Int J Mol Sci. 2015 Sep 7;16(9):21237–76. doi: 10.3390/ijms160921237 (PMC4613251; doi:10.3390/ijms160921237)
Supplement: Supplementary file 1 [file ijms-16-21237-s001.pdf]

# Supplementary Information

**Table S1.** CD Analysis of Lysozyme. The DInaMo calculations are for the minimized or rebuilt structure using CDCALC or CAPPS. All RMSDs are calculated between 180 and 210 nm.

| **CD Method** | **Peak Wavelength (nm)** | **Δε ($M^{-1}\cdot cm^{-1}$)** | **Peak Wavelength (nm)** | **Δε ($M^{-1}\cdot cm^{-1}$)** | **RMSD ($M^{-1}\cdot cm^{-1}$)** |
|---|---|---|---|---|---|
| [a] SRCD | 191 | 6.01 | 207 | −4.68 | 0.000 |
| [b] 4000 Ho | 190 | 12.79 | 202 | −4.75 | 3.079 |
| [b] 6000 Ho | 190 | 6.51 | 205 | −1.83 | 1.620 |
| [b] 4000 Hx | 190 | 13.30 | 202 | −5.08 | 3.433 |
| [b] 6000 Hx | 189 | 6.87 | 205 | −2.00 | 1.768 |
| [b] 4000 Hy | 190 | 12.64 | 202 | −5.83 | 3.836 |
| [b] 6000 Hy | 188 | 6.70 | 204 | −2.54 | 2.060 |
| [b] 4000 Jo | 196 | 12.09 | 209 | −4.45 | 3.755 |
| [b] 6000 Jo | 195 | 6.10 | 212 | −1.71 | 2.595 |
| [b] 4000 Jx | 197 | 12.06 | 209 | −4.78 | 3.957 |
| [b] 6000 Jx | 196 | 6.30 | 212 | −1.90 | 2.693 |
| [b] 4000 Jy | 196 | 11.15 | 208 | −3.11 | 3.860 |
| [b] 6000 Jy | 196 | 5.45 | 212 | −0.93 | 2.919 |
| [b] 4000 OL | 194 | 17.94 | 207 | −1.17 | 5.783 |
| [b] 6000 OL | 194 | 9.63 | not observed | − | 3.798 |
| [c] 4000 Ho | 192 | 12.89 | 208 | −7.08 | 3.390 |
| [c] 6000 Ho | 192 | 6.87 | 210 | −3.38 | 1.175 |
| [c] 4000 Hx | 192 | 11.75 | 208 | −7.18 | 2.852 |
| [c] 6000 Hx | 192 | 6.375 | 210 | −3.38 | 0.984 |
| [c] 4000 Hy | 192 | 10.61 | 208 | −7.40 | 2.497 |
| [c] 6000 Hy | 192 | 5.95 | 210 | −3.70 | 0.934 |
| [c] 4000 Jo | 198 | 12.12 | 214 | −6.66 | 6.022 |
| [c] 6000 Jo | 198 | 6.44 | 217 | −3.17 | 3.974 |
| [c] 4000 Jx | 198 | 10.32 | 215 | −6.22 | 5.538 |
| [c] 6000 Jx | 198 | 5.69 | 217 | −3.03 | 2.859 |
| [c] 4000 Jy | 198 | 13.95 | 212 | −8.53 | 6.211 |
| [c] 6000 Jy | 198 | 6.46 | 214 | −4.02 | 3.609 |
| [c] 4000 OL | 194 | 11.02 | 208 | −7.18 | 7.478 |
| [c] 6000 OL | 192 | 12.89 | 211 | −2.81 | 3.585 |
| [d] 4000 Ho | 190 | 11.36 | 206 | −8.11 | 3.117 |
| [d] 6000 Ho | 190 | 6.49 | 208 | −4.03 | 1.061 |
| [d] 4000 Hx | 190 | 11.44 | 206 | −8.07 | 3.194 |
| [d] 6000 Hx | 190 | 6.40 | 208 | −4.04 | 1.129 |
| [d] 4000 Hy | 190 | 10.87 | 206 | −9.52 | 3.722 |
| [d] 6000 Hy | 188 | 6.41 | 208 | −5.01 | 1.628 |
| [d] 4000 Jo | 196 | 9.47 | 208 | −5.85 | 2.870 |
| [d] 6000 Jo | 196 | 4.19 | 210 | −2.60 | 2.134 |
| [d] 4000 Jx | 196 | 8.92 | 208 | −5.82 | 2.500 |

**Table S1.** *Cont.*

| CD Method | Peak Wavelength (nm) | Δε ($M^{-1}·cm^{-1}$) | Peak Wavelength (nm) | Δε ($M^{-1}·cm^{-1}$) | RMSD ($M^{-1}·cm^{-1}$) |
|---|---|---|---|---|---|
| [d] 6000 Jx | 194 | 3.96 | 210 | −2.66 | 1.991 |
| [d] 4000 Jy | 198 | 10.49 | 210 | −9.14 | 4.068 |
| [d] 6000 Jy | 196 | 4.88 | 212 | −4.48 | 2.188 |
| [e] 4000 Hy | 189 | 11.15 | 203 | −6.98 | 3.194 |
| [f] 6000 Hy | 189 | 6.20 | 206 | −3.41 | 1.712 |
| [g] SrWo1 | 190 | 6.39 | 207 | −2.05 | 0.876 |
| [h] SrWo2 | 190 | 6.29 | 210 | −2.46 | 0.940 |
| [i] MM1 | 192 | 7.40 | 211 | −4.43 | 1.380 |
| [j] MM2 | 192 | 7.20 | 210 | −4.82 | 1.444 |
| [k] MM3 | 192 | 5.37 | 210 | −4.23 | 0.930 |

[a] SRCD from the Protein Circular Dichroism Data Bank (CD0000045000) [44,47]; [b] CDALC using PDB structure 2VB1 that was minimized via Insight®II/Discover/CVFF. All hydrogens are included; [c] CDCALC, NAMD/CHARMM22 5000 steps. Hs attached to $-CH_3$s are ignored; [d] CAPPS, Rebuilt PDB code 2VB1 [56] secondary structures only. The following residues were ignored: 1–3, 43–45, 51–53, and 116–118. All hydrogens included; [e] CAPPS, Rebuilt PDB code 1LSE [97] published by Bode and Applequist [3] with a bandwidth of 4000 $cm^{-1}$; [f] CAPPS, Rebuilt PDB code 1LSE [97] published by Bode and Applequist [3] with a bandwidth of 6000 $cm^{-1}$; [g] Matrix method with semi empirical parameters on peptide groups only using PDB structure 7LYZ [13,98]; [h] Matrix method with semi empirical on peptide groups and aromatic side chains using PDB structure 7LYZ [13,98]; [i] Matrix method with *ab initio* parameters including only the protein backbone transitions using PDB structure 193L [55,99]; [j] Matrix method with *ab initio* parameters including protein backbone and charge-transfer transitions using PDB structure 193L [55,99]; [k] Matrix method with *ab initio* parameters including protein backbone, charge-transfer and side chain transitions using PDB structure 193L [55,99]; Grey highlight represents the smallest RMSD for a set of calculations.

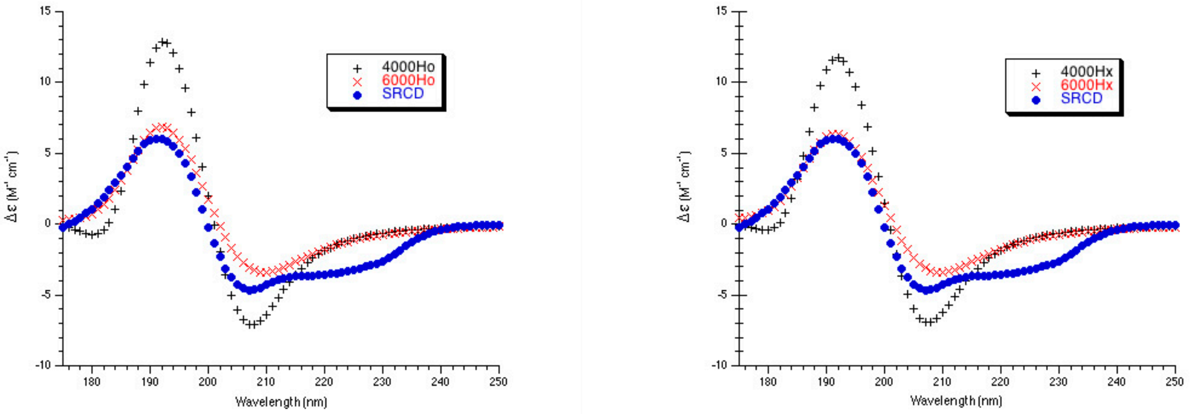


**Figure S1.** *Cont.*

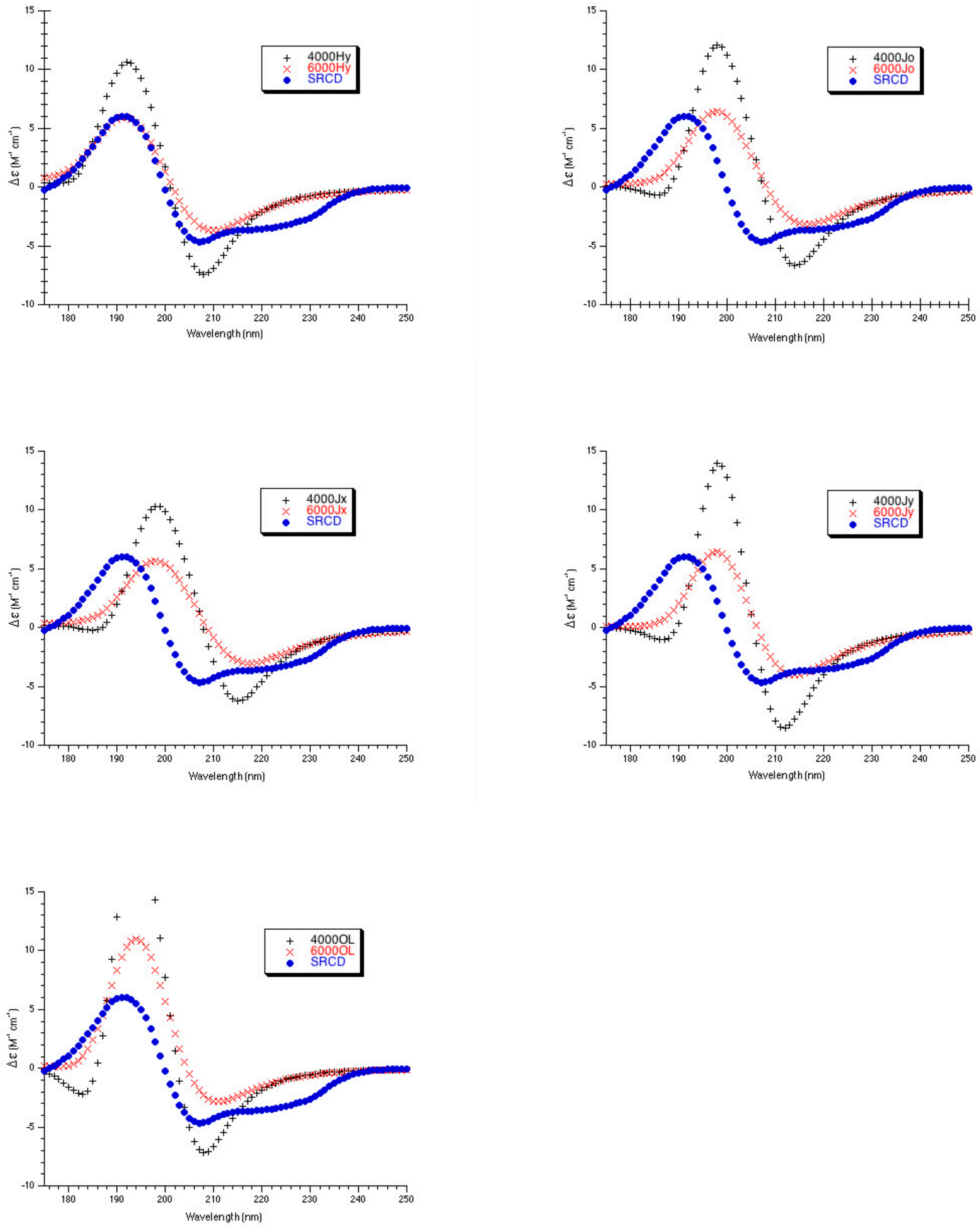


**Figure S1.** Lysozyme Predicted CD Using CDCALC and 2VB1 Minimized via NAMD/CHARMM22. Calculated spectra ignore all −$CH_3$ group hydrogens. Bandwidths are 4000 (+) and 6000 (×) $cm^{-1}$. The blue dots (•) are the experimental SRCD (CD0000045000) [44,47]. The CATH fold classification [53] is mainly alpha/orthogonal bundle.

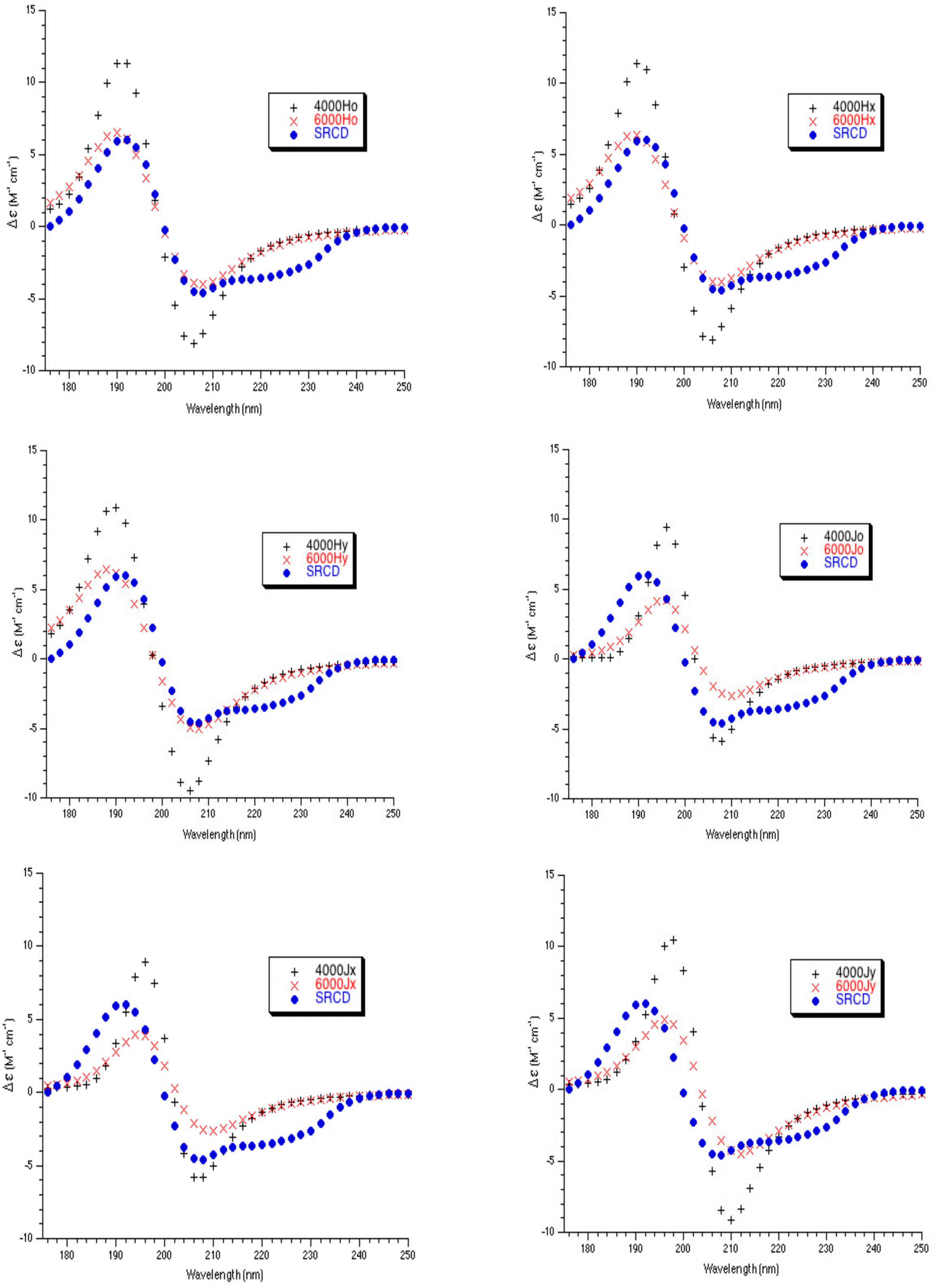


**Figure S2.** Lysozyme Predicted CD Using CAPPS (2VB1). The rebuilt structure ignores residues 1–3, 43–45, 51–53, and 116–118. All other structures are included with hydrogens. Bandwidths are 4000 (+) and 6000 (×) $cm^{-1}$. The blue dots (•) are the experimental SRCD (CD0000045000) [44,47]. The CATH fold classification [53] is mainly alpha/orthogonal bundle.

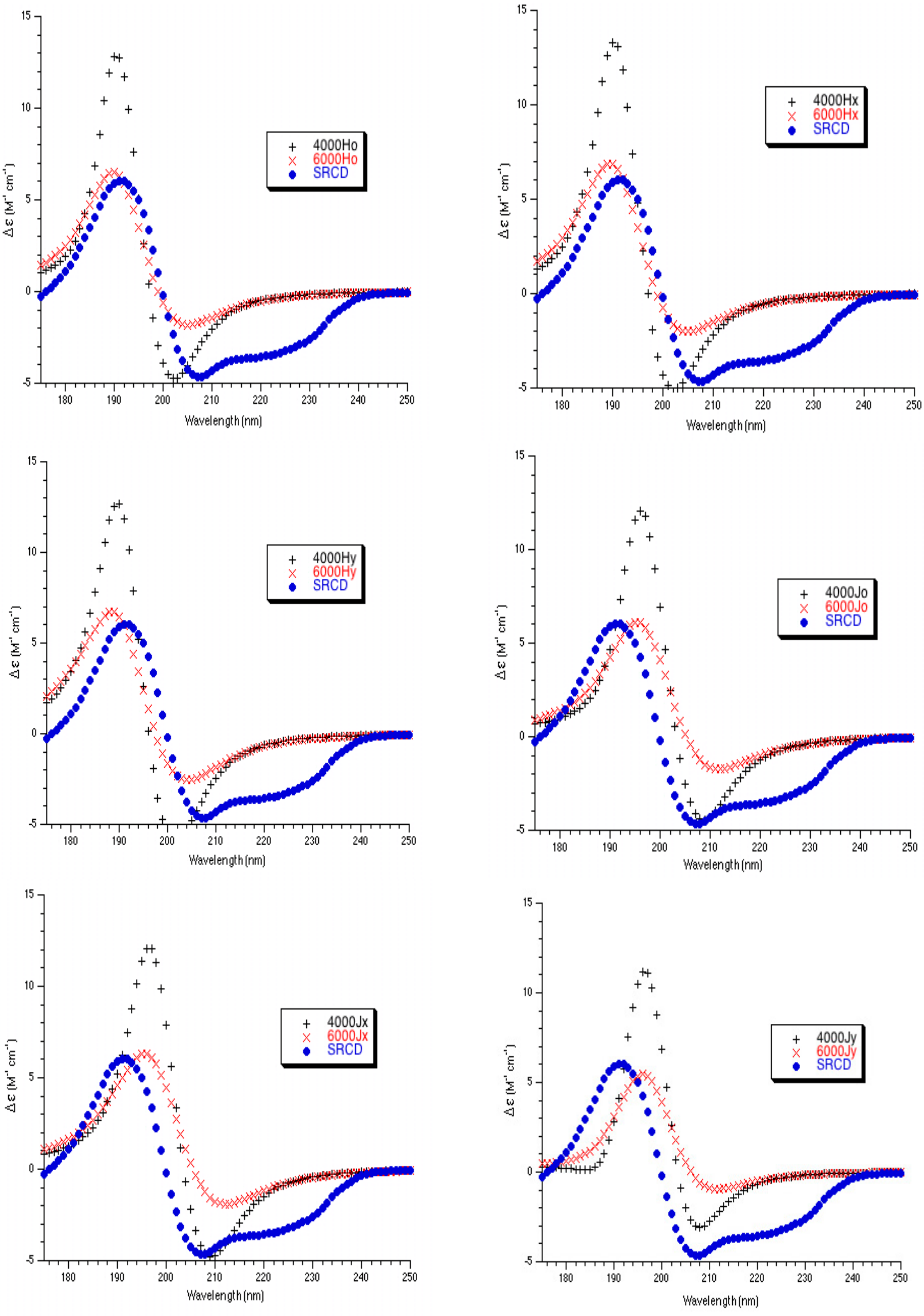


**Figure S3.** *Cont.*

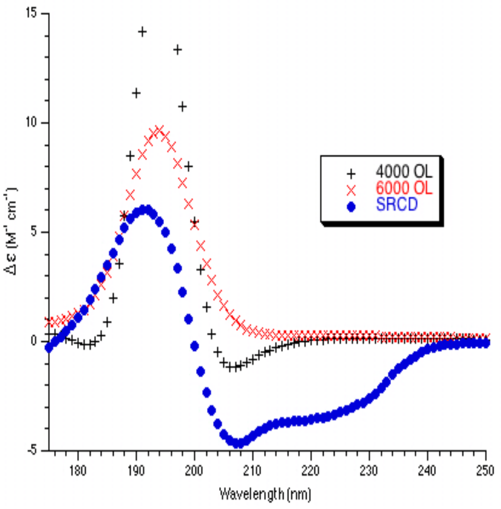


**Figure S3.** Lysozyme Predicted CD Using CDCALC & 2VB1 Minimized via Insight®II/Discover. Calculated spectra include all hydrogens. Bandwidths are 4000 (+) and 6000 (×) $cm^{-1}$. The blue dots (•) are the experimental SRCD (CD0000045000) [44,47]. The CATH fold classification [53] is mainly alpha/orthogonal bundle.

Cytochrome c: The small heme-containing electron transport protein cytochrome c (Figure S4) is a globular monomeric protein with 129 amino acids that CATH classifies as a mainly alpha type structure [53]. It is stable under a variety of conditions, and the change in oxidation state of the iron in the heme does not impact the cytochrome c's experimental far-UV CD spectrum [100]. Cytochrome c has five α-helices (48.1%), one β-bridge (1.9%), several turns (7.7%), and 32.7% of the molecule is irregular [44] (Figure S4).

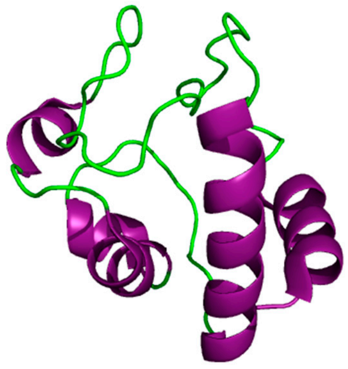

**Figure S4.** Cytochrome c Secondary Structure. PDB code 1HRC [79] structure showing secondary structure elements: thick purple cartoons/coils correspond to α-helices (1–14, 49–55, 60–68, 70–75, 87–104), and the thin green ropes are turns and other structures. The CATH fold classification [53] is mainly alpha/orthogonal bundle.

**Table S2.** CD Analysis of Cytochrome c. The DInaMo calculations are for the minimized or rebuilt structure using CDCALC or CAPPS. All RMSDs are calculated between 180 and 210 nm.

| CD Method | Peak Wavelength (nm) | Δε ($M^{-1}\cdot cm^{-1}$) | Peak Wavelength (nm) | Δε ($M^{-1}\cdot cm^{-1}$) | RMSD ($M^{-1}\cdot cm^{-1}$) |
|---|---|---|---|---|---|
| [a] SRCD | 195 | 4.30 | 210 | –4.29 | 0.000 |
| [b] 4000Ho | 191 | 6.92 | 207 | −6.66 | 2.689 |
| [b] 6000Ho | 189 | 3.58 | 208 | −3.75 | 1.496 |
| [b] 4000Hx | 190 | 7.45 | 207 | −7.03 | 3.078 |
| [b] 6000Hx | 189 | 4.10 | 208 | −3.94 | 1.587 |
| [b] 4000Hy | 190 | 6.52 | 207 | −6.49 | 2.565 |
| [b] 6000Hy | 189 | 3.65 | 208 | −3.64 | 1.480 |
| [b] 4000Jo | 196 | 6.49 | 214 | −6.23 | 1.662 |
| [b] 6000Jo | 195 | 3.37 | 214 | −3.49 | 1.095 |
| [b] 4000Jx | 196 | 6.48 | 214 | −6.40 | 1.770 |
| [b] 6000Jx | 195 | 3.63 | 216 | −3.61 | 1.085 |
| [b] 4000Jy | 197 | 8.58 | 211 | −6.58 | 2.817 |
| [b] 6000Jy | 196 | 4.04 | 213 | −3.38 | 1.384 |
| [b] 4000OL | 193 | 10.30 | 208 | −8.02 | 3.506 |
| [b] 6000OL | 192 | 5.04 | 210 | −4.29 | 0.756 |
| [c] 4000Ho | 192 | 15.19 | 206 | −12.59 | 7.293 |
| [c] 6000Ho | 190 | 8.00 | 208 | −6.52 | 3.036 |
| [c] 4000Hx | 190 | 14.74 | 206 | −12.12 | 7.161 |
| [c] 6000Hx | 190 | 7.99 | 208 | −6.28 | 3.062 |
| [c] 4000Hy | 190 | 14.58 | 206 | −13.26 | 7.617 |
| [c] 6000Hy | 190 | 7.93 | 208 | −7.10 | 3.483 |
| [c] 4000Jo | 196 | 12.04 | 208 | −8.91 | 4.297 |
| [c] 6000Jo | 196 | 4.97 | 210 | −4.26 | 1.037 |
| [c] 4000Jx | 196 | 11.57 | 208 | −8.49 | 3.979 |
| [c] 6000Jx | 196 | 4.84 | 210 | −4.09 | 0.886 |
| [c] 4000Jy | 198 | 14.19 | 210 | −12.07 | 5.784 |
| [c] 6000Jy | 196 | 6.11 | 212 | −6.13 | 1.751 |
| [d] SW04:1 | 191 | 8.40 | 221 | −2.42 | 2.348 |

**Table S2.** *Cont.*

| CD Method | Peak Wavelength (nm) | Δε ($M^{-1}\cdot cm^{-1}$) | Peak Wavelength (nm) | Δε ($M^{-1}\cdot cm^{-1}$) | RMSD ($M^{-1}\cdot cm^{-1}$) |
|---|---|---|---|---|---|
| [e] SW04:2 | 192 | 8.10 | 221 | −2.06 | 3.242 |
| [f] BA98:1 | 186 | 18.74 | 199 | −19.11 | 2.425 |
| [g] BA98:2 | 184 | 8.17 | 206 | −10.37 | 1.183 |
| [h] AB99:1 | 192 | 8.88 | 207 | −5.090 | 2.731 |
| [i] AB99:2 | 192 | 9.42 | 207 | −4.67 | 3.162 |
| [j] AB99:3 | 193 | 10.8 | 208 | −5.97 | 4.236 |
| [k] MM1 | 193 | 8.68 | 214 | −3.79 | 2.287 |
| [l] MM2 | 195 | 8.14 | 212 | −4.01 | 1.967 |
| [m] MM3 | 195 | 3.74 | 217 | −2.33 | 2.055 |

[a] SRCD CD0000021000 from the Protein Circular Dichroism Data Bank [44,47]; [b] CDCALC using PDB code 1HRC [79] with NAMD/CHARMM22 5000 conjugate gradient steps. Hs attached to $-CH_3$s are ignored; [c] CAPPS, Rebuilt PDB code 1HRC [79] secondary structures including hydrogens. The following residues were ignored: 1–48, and 69; [d] SW04:1 Matrix method with semi empirical parameters using PDB code 3CYT [101] including only peptide groups [13]; [e] SW04:2 Matrix method with semi empirical parameters using PDB code 3CYT [101] including all aromatic side chains and peptide groups [13]; [f] BA98:1 Dipole interaction model using PDB code 1HRC [79] including residues 3–12, 14–18, 20–24, 26–30, 32–36, 38–40, 50–53, 55–59, 61–67, 71–73, 75–76, 88–103 with set Hy at 4000 $cm^{-1}$ [3]; [g] BA98:2 Dipole interaction model of PDB code 1HRC [79] including residues 3–12, 14–18, 20–24, 26–30, 32–36, 38–40, 50–53, 55–59, 61–67, 71–73, 75–76, 88–103 with set Hy at 6000 $cm^{-1}$ [3]; [h] AB99:1 Dipole interaction model using PDB code 1HRC [79] including residues 3–12, 14–18, 20–24, 26–30, 32–36, 38–40, 50–53, 55–59, 61–67, 71–73, 75–76, 88–103 with set Hy at 4000 $cm^{-1}$ of the protein only [102]; [i] AB99:2 Dipole interaction model using PDB code 1HRC [79] including residues 3–12, 14–18, 20–24, 26–30, 32–36, 38–40, 50–53, 55–59, 61–67, 71–73, 75–76, 88–103 at 4000 $cm^{-1}$ of the protein and bound waters [102]; [j] AB99:3 Dipole interaction model using PDB code 1HRC [79] including residues 3–12, 14–18, 20–24, 26–30, 32–36, 38–40, 50–53, 55–59, 61–67, 71–73, 75–76, 88–103 at 4000 $cm^{-1}$ of the protein and bound waters and lattice with continuum [102]; [k] MM1 Matrix method with *ab initio* parameters including only the protein backbone transitions using PDB structure 1HRC [55,79]; [l] MM2 Matrix method with *ab initio* parameters including protein backbone and charge-transfer transitions using PDB structure 1HRC [55,79]; [m] MM3 Matrix method with *ab initio* parameters including protein backbone, charge-transfer and side chain transitions using PDB structure 1HRC [55,79]; Grey highlight represents the smallest RMSD for a set of calculations.

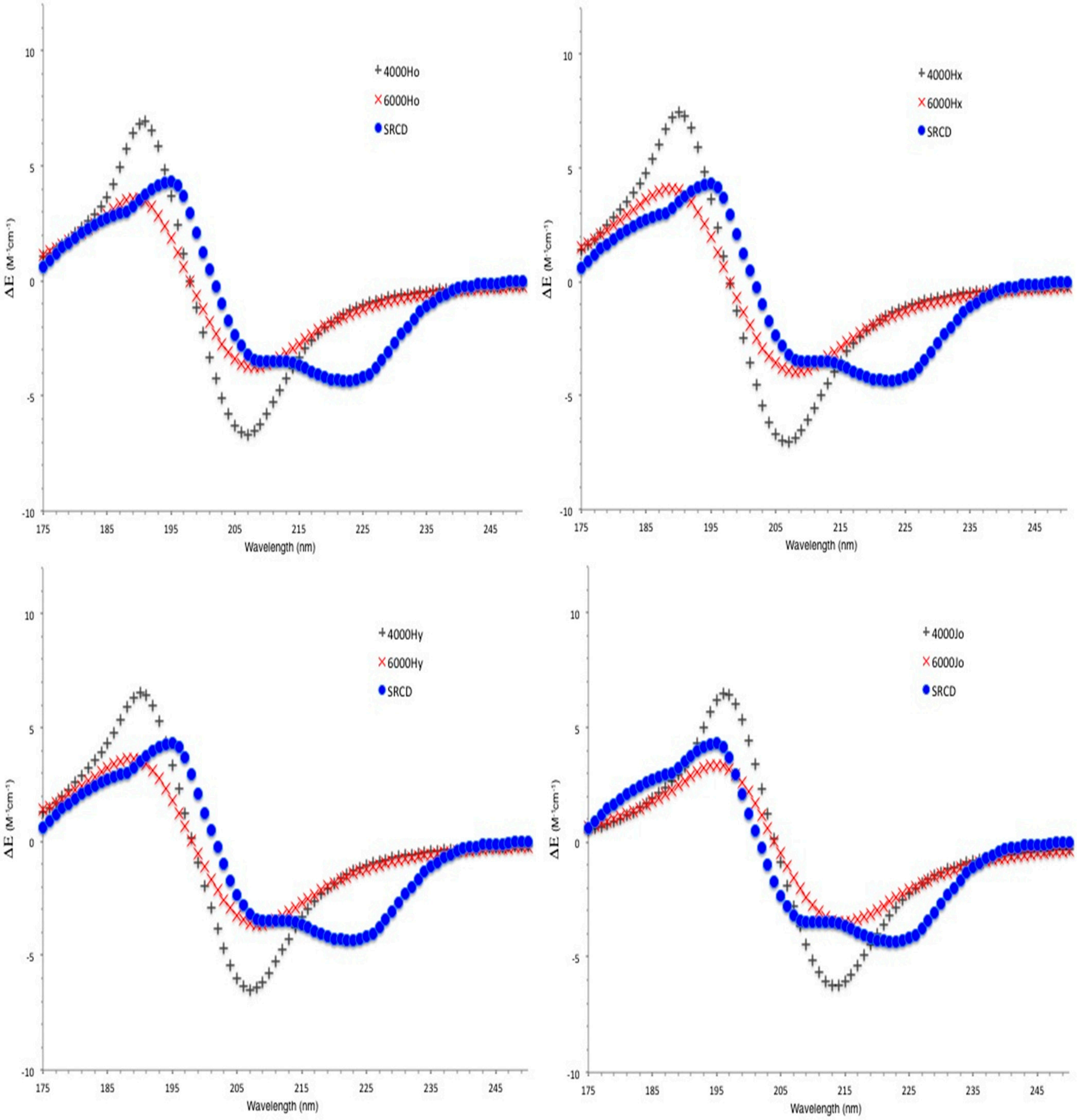


**Figure S5.** *Cont.*

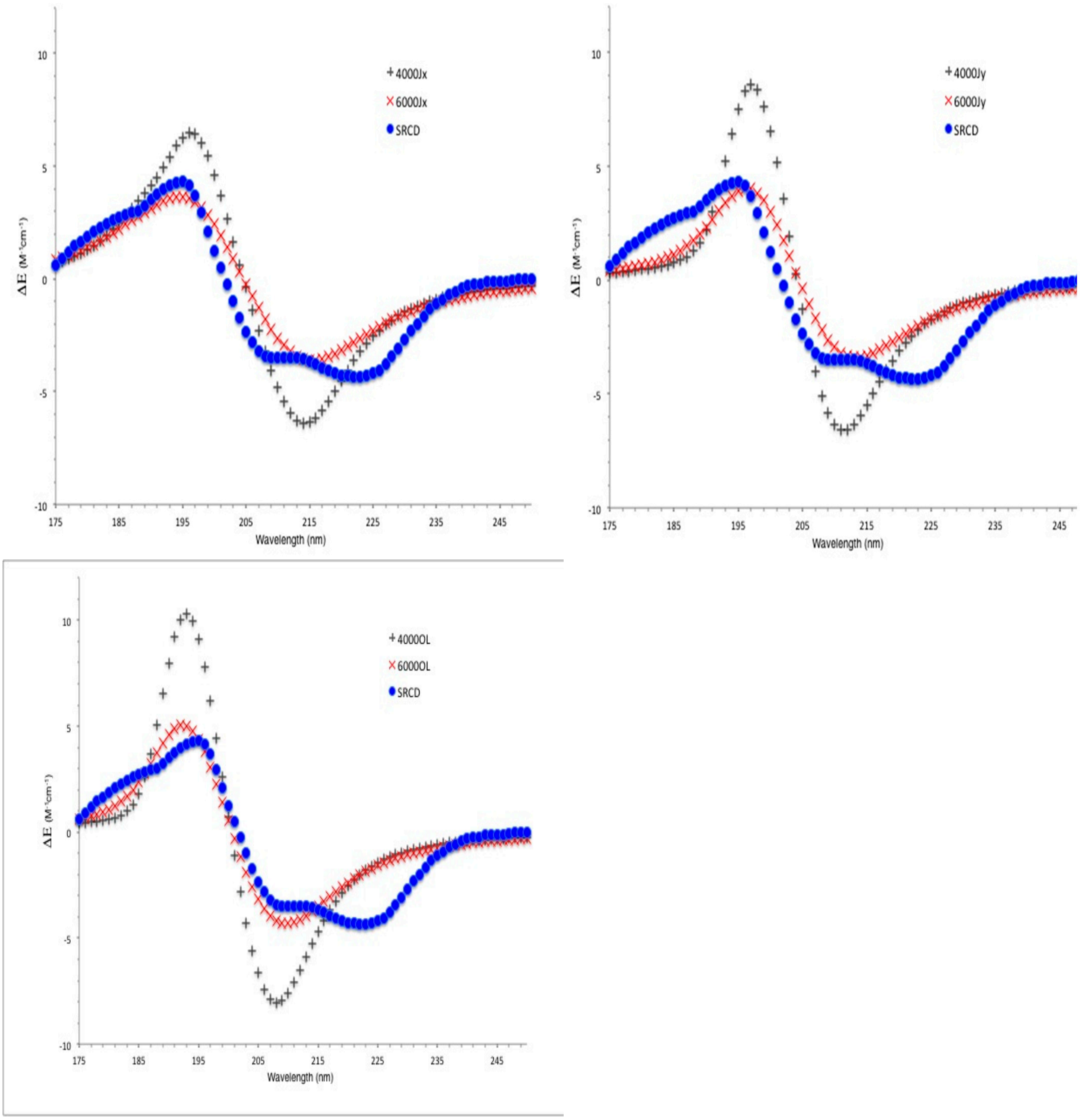


**Figure S5.** Cytochrome c Predicted CD Using CDCALC. The PDB 1HRC structure was minimized via NAMD/CHARMM22 using 5000 conjugate gradient steps. Calculated spectra ignore all $-CH_3$ group hydrogens. Bandwidths are 4000 (+) and 6000 (×) $cm^{-1}$. The blue dots (•) are the experimental SRCD (CD0000021000) [44,47]. The CATH fold classification [53] is mainly alpha/orthogonal bundle.

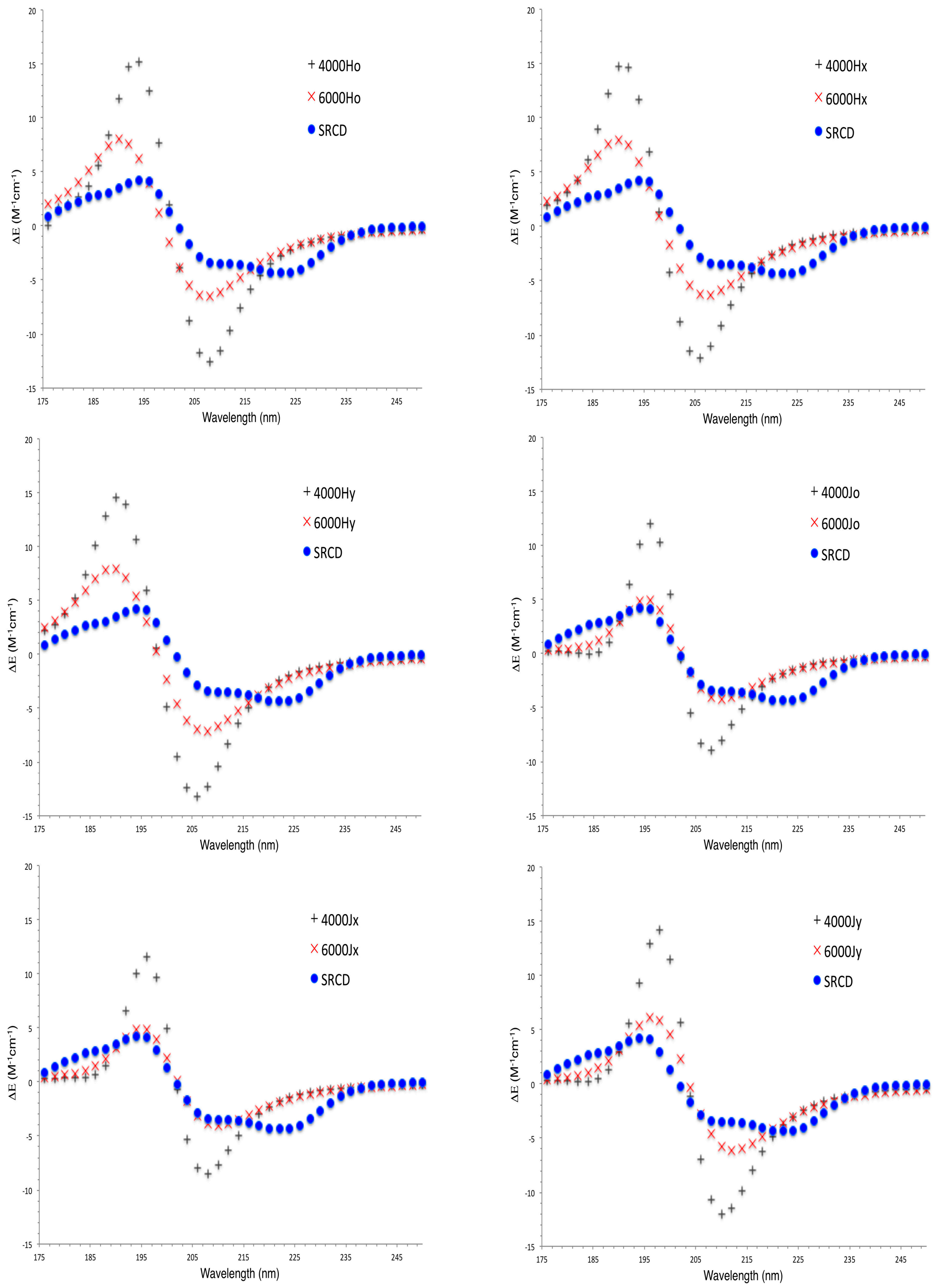


**Figure S6.** Horse Heart Cytochrome c Predicted CD Using CAPPS. The PDB 1HRC structure was rebuilt ignoring residues 1–48 and 69. Bandwidths are 4000 (+) and 6000 (×) $cm^{-1}$. The blue dots (•) are the experimental SRCD (CD0000021000) [44,47]. The CATH fold classification [53] is mainly alpha/orthogonal bundle.

Phospholipase A2: The PDB code 1UNE (Figure S7) [89] is a small monomeric membrane protein with 123 amino acids (Figure 2). It catalyzes the hydrolysis reaction of the sn-2 ester bond of phospholipids [103,104]. The α-helix is the dominant structure (44.7%), but there are small amounts of other secondary structures including some $3_{10}$-helix 4.9%, β-strand 6.5%, β-bridge 2.4%, bonded turns 16.3%, bend 5.7% and 19.5% irregular [44].

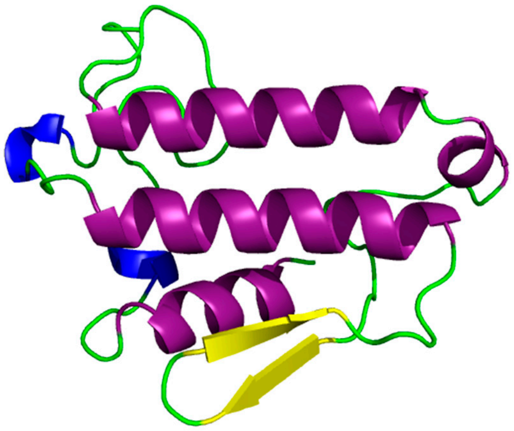

**Figure S7.** Phospholipase A2 Secondary Structure. PDB code 1UNE [89] structure showing secondary structure elements: thick purple cartoons/coils correspond to α-helices (2–12, 40–57, 59–63, 90–108), the short blue cartoons/coils correspond to $3_{10}$-helices (18–21, 113–115) the yellow tapes are β-sheets, (75–78, 81–84) and the thin green ropes are turns and other structures. The CATH fold classification [53] is mainly alpha/up-down bundle.

**Table S3.** CD Analysis of Phospholipase A2. The DInaMo calculations are for the minimized or rebuilt structure using CDCALC or CAPPS. All RMSDs are calculated between 180 and 210 nm.

| CD Method | Peak Wavelength (nm) | Δε ($M^{-1}\cdot cm^{-1}$) | Peak Wavelength (nm) | Δε ($M^{-1}\cdot cm^{-1}$) | RMSD ($M^{-1}\cdot cm^{-1}$) |
|---|---|---|---|---|---|
| [a] SRCD | 192 | 6.96 | 209 | −4.63 | 0.000 |
| [b] 4000Ho | 189 | 11.00 | 207 | −9.39 | 3.967 |
| [b] 6000Ho | 188 | 5.90 | 208 | −5.34 | 2.102 |
| [b] 4000Hx | 188 | 11.64 | 206 | −10.41 | 4.793 |
| [b] 6000Hx | 188 | 6.40 | 207 | −5.96 | 2.513 |
| [b] 4000Hy | 188 | 10.15 | 207 | −8.88 | 3.782 |
| [b] 6000Hy | 187 | 5.80 | 208 | −5.10 | 2.209 |
| [b] 4000Jo | 195 | 10.30 | 213 | −8.83 | 2.727 |
| [b] 6000Jo | 194 | 5.55 | 214 | −5.00 | 1.615 |
| [b] 4000Jx | 194 | 10.59 | 214 | −9.44 | 2.857 |
| [b] 6000Jx | 194 | 5.91 | 215 | −5.41 | 1.509 |
| [b] 4000Jy | 196 | 13.33 | 212 | −9.08 | 4.544 |
| [b] 6000Jy | 196 | 6.51 | 213 | −4.74 | 2.281 |
| [b] 4000OL | 192 | 16.88 | 208 | −10.94 | 5.435 |
| [b] 6000OL | 191 | 8.54 | 210 | −5.92 | 0.994 |

**Table S3.** *Cont.*

| CD Method | Peak Wavelength (nm) | Δε ($M^{-1}\cdot cm^{-1}$) | Peak Wavelength (nm) | Δε ($M^{-1}\cdot cm^{-1}$) | RMSD ($M^{-1}\cdot cm^{-1}$) |
|---|---|---|---|---|---|
| [c] 4000Ho | 192 | 12.93 | 206 | −10.42 | 4.846 |
| [c] 6000Ho | 190 | 6.92 | 206 | −5.53 | 1.821 |
| [c] 4000Hx | 190 | 12.68 | 204 | −10.32 | 4.842 |
| [c] 6000Hx | 190 | 6.94 | 206 | −5.42 | 1.900 |
| [c] 4000Hy | 190 | 12.78 | 206 | −11.32 | 5.313 |
| [c] 6000Hy | 190 | 6.90 | 206 | −6.12 | 2.246 |
| [c] 4000Jo | 196 | 8.76 | 206 | −7.57 | 2.425 |
| [c] 6000Jo | 194 | 3.80 | 210 | −3.69 | 1.934 |
| [c] 4000Jx | 196 | 8.59 | 206 | −7.42 | 2.273 |
| [c] 6000Jx | 194 | 3.89 | 208 | −3.61 | 1.860 |
| [c] 4000Jy | 198 | 10.42 | 210 | −10.48 | 3.796 |
| [c] 6000Jy | 196 | 4.52 | 212 | −5.40 | 1.830 |
| [d] B09:1 | 191 | 11.10 | 210 | −6.93 | 2.557 |
| [e] B09:2 | 192 | 10.77 | 209 | −7.51 | 2.455 |
| [f] B09:3 | 191 | 9.37 | 209 | −7.25 | 1.831 |

[a] SRCD from the Protein Circular Dichroism Data Bank (CD0000059000) [44,47]. [b] CDALC using PDB structure 1UNE [89] that was minimized via NAMD/CHARMM22 5000 steps; Hs attached to $-CH_3$s are ignored; [c] CAPPS, Rebuilt PDB code 1UNE [89] secondary structures only. The following residues were ignored: 1, 18-21, 58, and 113-115. All hydrogens included; [d] Matrix method with *ab initio* parameters on PDB code 1UNE [89] using *ab initio* parameters including only the protein backbone transitions [55]; [e] Matrix method with *ab initio* parameters on PDB code 1UNE [89] using *ab initio* parameters including protein backbone and charge-transfer transitions [55]; [f] Matrix method with *ab initio* parameters on PDB code 1UNE [89] using *ab initio* parameters including protein backbone, charge-transfer and side chain transition [55]. Grey highlight represents the smallest RMSD for a set of calculations.

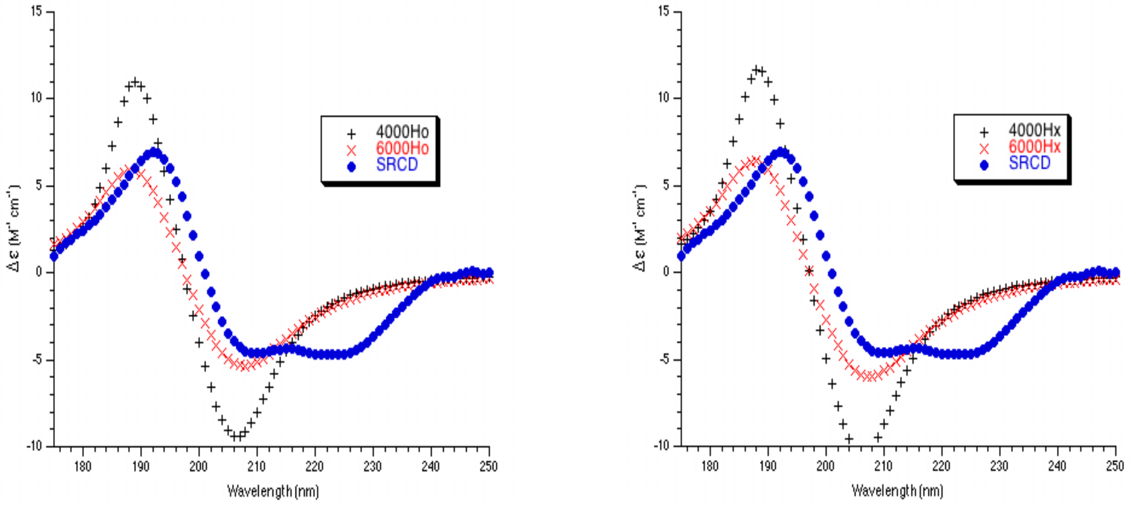


**Figure S8.** *Cont.*

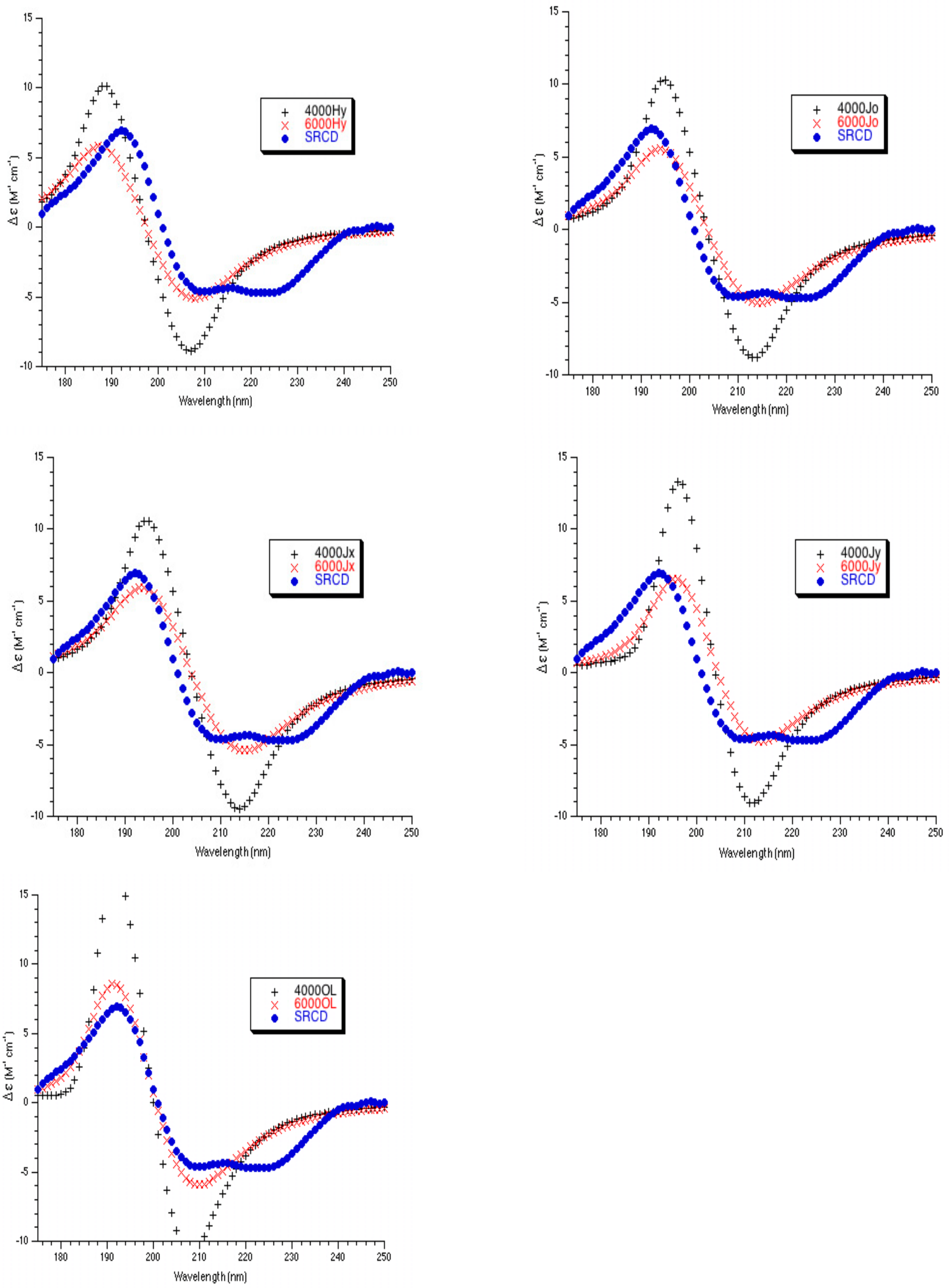


**Figure S8.** Phospholipase A2 Predicted CD Using CDCALC. The PDB 1UNE structure was minimized via NAMD/CHARMM22 for 5000 conjugate gradient steps. Calculated spectra ignore all −$CH_3$ group hydrogens. Bandwidths are 4000 (+) and 6000 (×) $cm^{-1}$. The blue dots (•) are the experimental SRCD (CD0000045000) [44,47]. The CATH fold classification [53] is mainly alpha/up−down bundle.

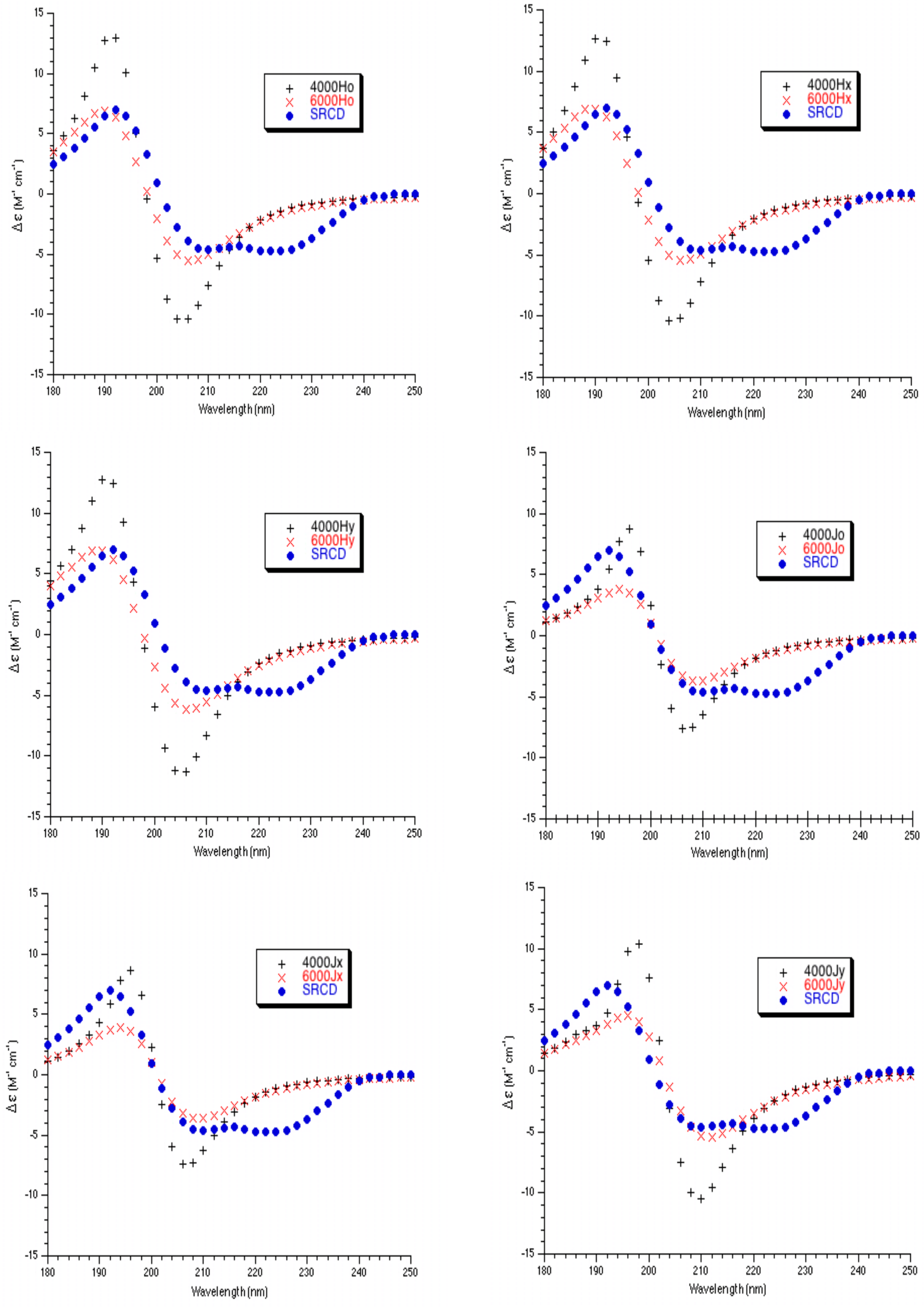


**Figure S9.** Phospholipase A2 Predicted CD Using CAPPS. The PDB 1UNE [89] structure was rebuilt with idealized bond lengths and angles. Residues 1, 18–21, 58, and 113–115 were ignored. Bandwidths are 4000 (+) and 6000 (×) $cm^{-1}$. The blue dots (•) are the experimental SRCD (CD0000045000) [44,47]. The CATH fold classification is mainly alpha/up-down bundle [53].

**Table S4.** CD Analysis of Rhomboid Peptidase. The DInaMo calculations are for the minimized or rebuilt structure using CDCALC or CAPPS. All RMSDs are calculated between 180 and 210 nm.

| CD Method | Peak Wavelength (nm) | Δε ($M^{-1}{\cdot}cm^{-1}$) | Peak Wavelength (nm) | Δε ($M^{-1}{\cdot}cm^{-1}$) | RMSD ($M^{-1}{\cdot}cm^{-1}$) |
|---|---|---|---|---|---|
| [a] SRCD | 193 | 13.20 | 210 | −5.77 | 0.000 |
| [b] 4000 Ho | 190 | 15.67 | 207 | −12.85 | 3.916 |
| [b] 6000 Ho | 189 | 8.17 | 208 | −7.15 | 3.074 |
| [b] 4000 Hx | 189 | 17.27 | 207 | −13.81 | 4.546 |
| [b] 6000 Hx | 188 | 9.26 | 208 | −7.71 | 3.181 |
| [b] 4000 Hy | 189 | 13.78 | 207 | −12.30 | 3.786 |
| [b] 6000 Hy | 188 | 7.42 | 208 | −6.97 | 3.268 |
| [b] 4000 Jo | 195 | 14.74 | 213 | −12.06 | 1.997 |
| [b] 6000 Jo | 195 | 7.64 | 215 | −6.70 | 2.224 |
| [b] 4000 Jx | 195 | 15.01 | 214 | −12.64 | 1.871 |
| [b] 6000 Jx | 194 | 8.10 | 215 | −7.10 | 2.029 |
| [b] 4000 Jy | 198 | 18.23 | 211 | −12.38 | 4.140 |
| [b] 6000 Jy | 197 | 8.56 | 213 | −6.25 | 2.814 |
| [b] 4000 OL | 193 | 24.00 | 208 | −15.65 | 4.505 |
| [b] 6000 OL | 192 | 11.33 | 209 | −8.14 | 1.367 |
| [c] 4000 Ho | 190 | 17.86 | 206 | −13.86 | 6.766 |
| [c] 6000 Ho | 190 | 9.14 | 208 | −7.47 | 4.526 |
| [c] 4000 Hx | 190 | 17.81 | 206 | −13.63 | 7.226 |
| [c] 6000 Hx | 188 | 9.20 | 206 | −7.44 | 4.868 |
| [c] 4000 Hy | 190 | 17.26 | 206 | −15.25 | 7.959 |
| [c] 6000 Hy | 188 | 8.95 | 206 | −8.55 | 5.444 |
| [c] 4000 Jo | 196 | 13.15 | 208 | −10.31 | 3.889 |
| [c] 6000 Jo | 194 | 5.71 | 210 | −5.09 | 4.366 |
| [c] 4000 Jx | 196 | 12.56 | 206 | −10.07 | 3.704 |
| [c] 6000 Jx | 194 | 5.57 | 210 | −5.00 | 4.372 |
| [c] 4000 Jy | 198 | 14.65 | 210 | −14.49 | 5.284 |
| [c] 6000 Jy | 196 | 6.53 | 212 | −7.53 | 4.130 |

[a] SRCD from the PCDDB code CD0000109000 [44,59]; [b] CDCALC using PDB structure 2NR9 [58] minimized via NAMD/CHARMM22 and 10,000 conjugate gradient steps. The hydrogens on all $-CH_3$ groups are ignored; [c] CAPPS results using PDB structure 2NR9 [58] that contained rebuilt secondary structures including hydrogens; the following residues were ignored: 29, 40–42, 84–86, 193–195. Grey highlight represents the smallest RMSD for a set of calculations.

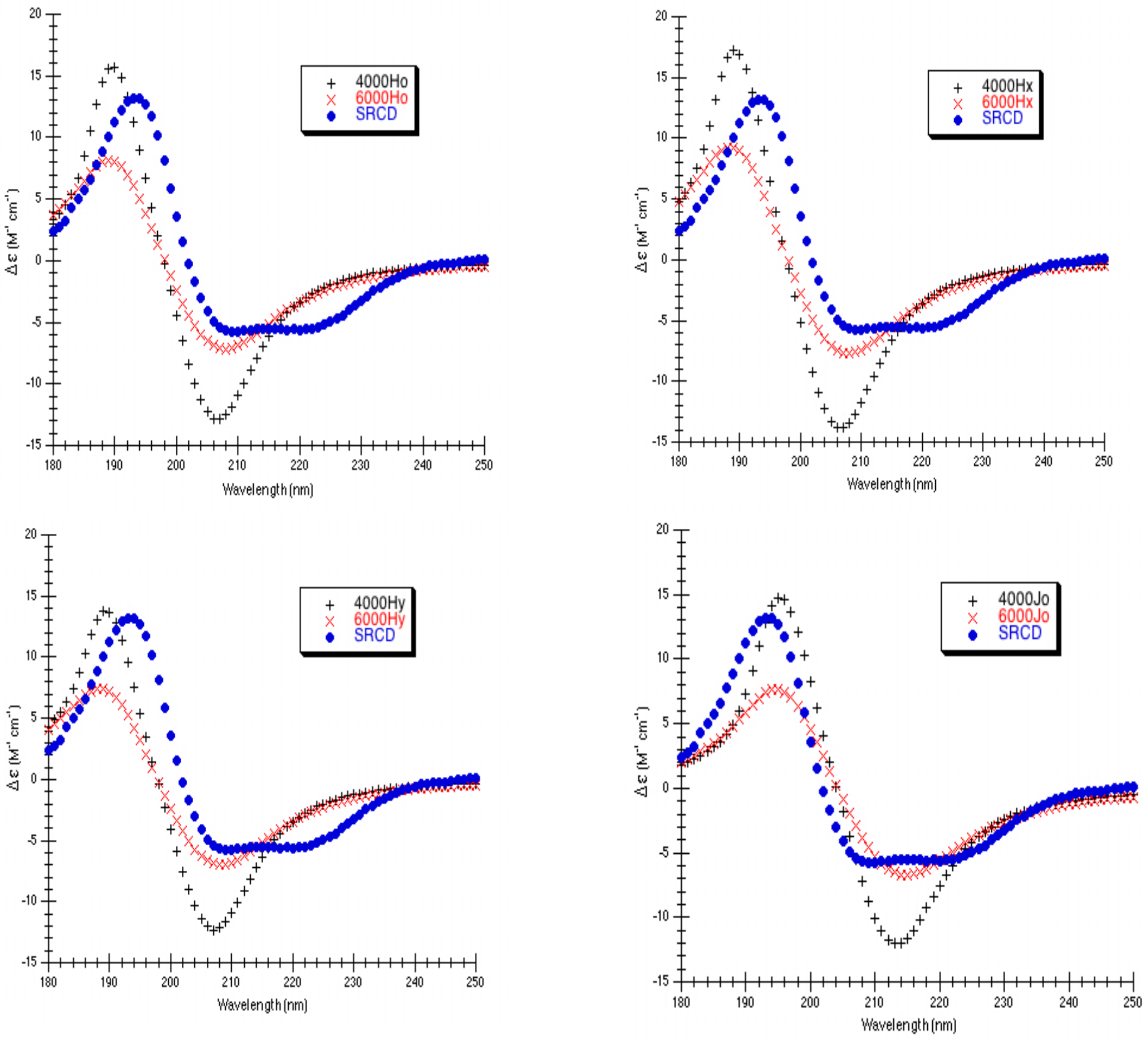


**Figure S10.** *Cont.*

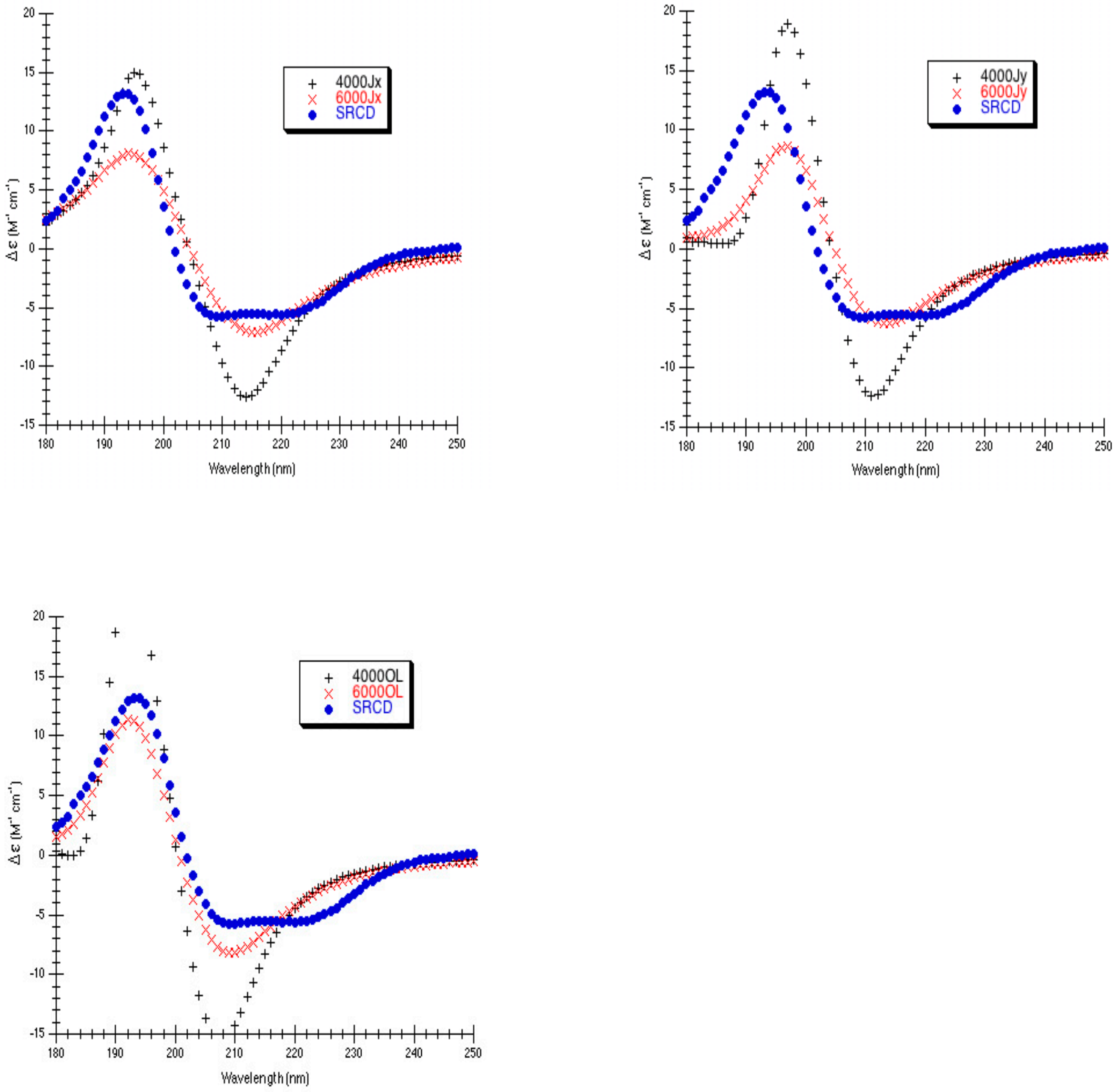


**Figure S10.** Rhomboid Peptidase Predicted CD Using CDCALC. The PDB 2NR9 structure was minimized with 10,000 conjugate gradient steps using NAMD/CHARMM22. Calculated spectra ignore all $-CH_3$ group hydrogens. Bandwidths are 4000 (+) and 6000 (×) $cm^{-1}$. The blue dots (•) are the experimental SRCD (CD0000109000) [44,59]. The CATH fold classification is mainly alpha/up-down bundle [53].

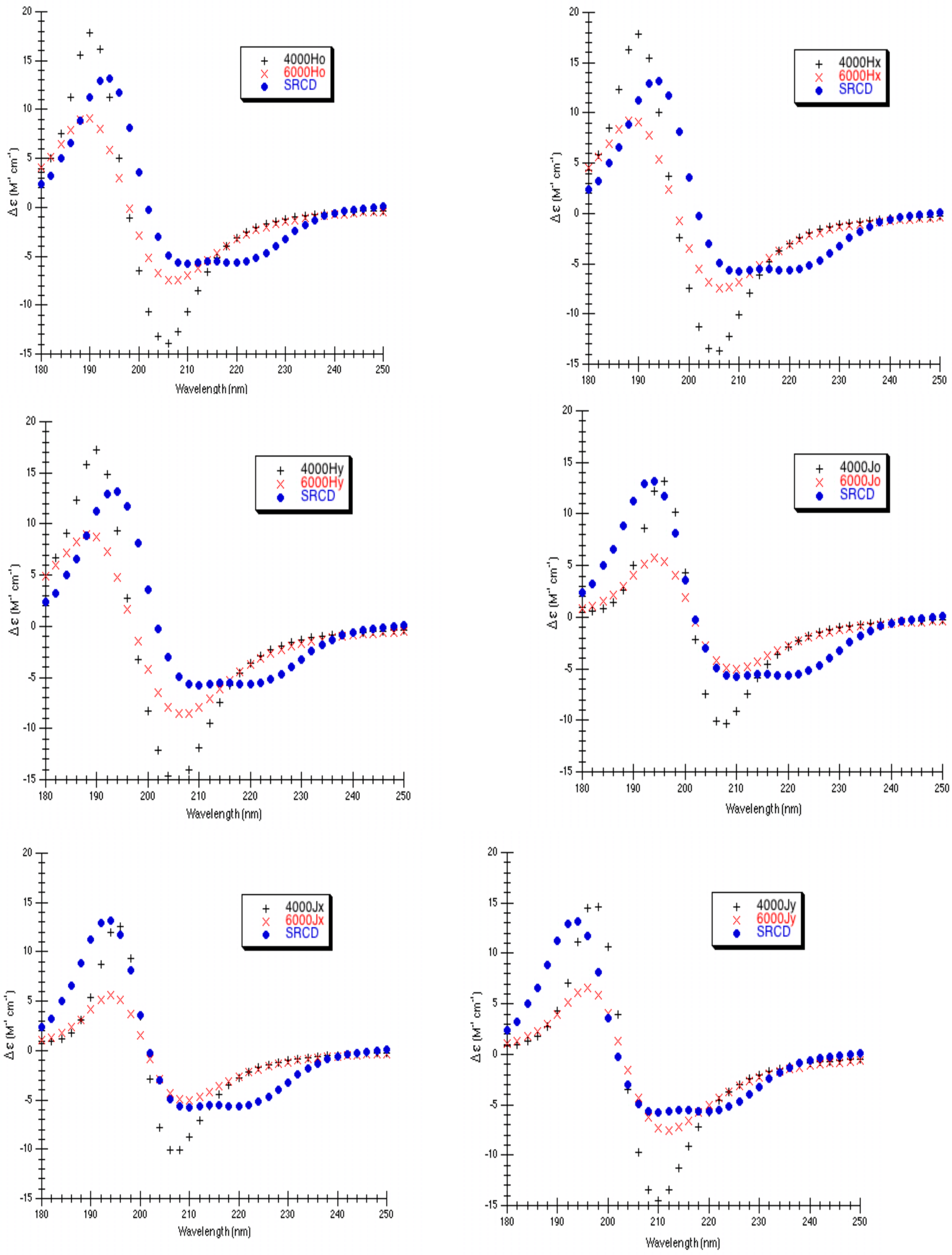


**Figure S11.** Rhomboid Peptidase Predicted CD Using CAPPS. The PDB 2NR9 [58] structure was rebuilt ignoring residues 29, 40–42, 84–86, and 193–195. Bandwidths are 4000 (+) and 6000 (×) $cm^{-1}$. The blue dots (•) are the experimental SRCD (CD000008000) [44,59]. The CATH fold classification is mainly alpha/up-down bundle [53].

Calmodulin: The smallish 148 amino acid calmodulin (PDB code 1LIN [77]) (Figure S12) is a multifunctional monomeric calcium binding protein that participates in signaling pathways that

regulate many crucial processes such as growth, proliferation, and movement [105]. The calmodulin structure is 56.8% α-helix, 5.4% β-strand, 8.1% bonded turns, 10.8% bend, and 18.9% irregular [44] (Figure S12).

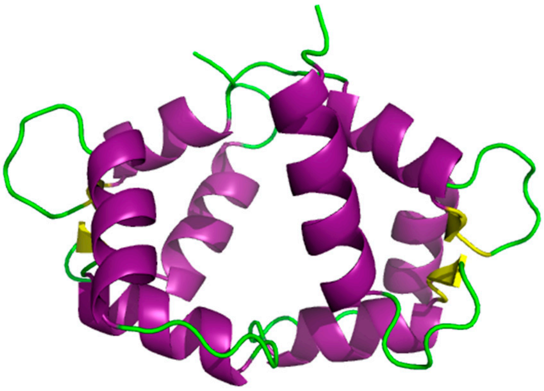

**Figure S12.** Calmodulin Secondary Structure. PDB code 1LIN [77] showing secondary structure elements: thick purple cartoons/coils correspond to α-helices (6–19, 29–38, 45–55, 65–73, 82–92, 102–111, 118–128, 138–145) the yellow tapes are β-sheets, (26–27, 63–64, 99–100, 136–137) and the thin green ropes are turns and other structures. Right: Predicted CD using CDCALC with the OL parameters. The CATH fold classification [53] is mainly alpha/orthogonal bundle.

**Table S5.** CD Analysis of Calmodulin. The DInaMo calculations are for the minimized or rebuilt structure using CDCALC or CAPPS. All RMSDs are calculated between 180 and 210 nm.

| **CD Method** | **Peak Wavelength (nm)** | **Δε ($M^{-1}·cm^{-1}$)** | **Peak Wavelength (nm)** | **Δε ($M^{-1}·cm^{-1}$)** | **RMSD ($M^{-1}·cm^{-1}$)** |
|---|---|---|---|---|---|
| [a] SRCD | 192 | 12.57 | 208 | −6.58 | 0.000 |
| [b] 4000 Ho | 190 | 11.65 | 206 | −9.23 | 3.500 |
| [b] 6000 Ho | 189 | 6.17 | 207 | −5.42 | 3.997 |
| [b] 4000 Hx | 189 | 12.41 | 206 | −10.84 | 4.379 |
| [b] 6000 Hx | 188 | 6.69 | 207 | −6.06 | 4.164 |
| [b] 4000 Hy | 189 | 10.21 | 207 | −9.50 | 3.844 |
| [b] 6000 Hy | 188 | 5.36 | 208 | −5.50 | 4.400 |
| [b] 4000 Jo | 195 | 11.01 | 213 | −9.23 | 3.546 |
| [b] 6000 Jo | 194 | 5.78 | 214 | −5.09 | 4.171 |
| [b] 4000 Jx | 195 | 11.04 | 214 | −9.85 | 3.189 |
| [b] 6000 Jx | 194 | 6.00 | 215 | −5.56 | 3.926 |
| [b] 4000 Jy | 197 | 14.43 | 211 | −9.98 | 5.278 |
| [b] 6000 Jy | 196 | 6.89 | 213 | −5.00 | 4.710 |
| [b] 4000 OL | 192 | 18.67 | 207 | −12.54 | 4.208 |
| [b] 6000 OL | 192 | 9.30 | 209 | −6.51 | 1.734 |

**Table S5.** *Cont.*

| CD Method | Peak Wavelength (nm) | Δε ($M^{-1}\cdot cm^{-1}$) | Peak Wavelength (nm) | Δε ($M^{-1}\cdot cm^{-1}$) | RMSD ($M^{-1}\cdot cm^{-1}$) |
|---|---|---|---|---|---|
| [c] 4000 Ho | 190 | 13.67 | 204 | −8.21 | 3.082 |
| [c] 6000 Ho | 190 | 7.01 | 206 | −4.24 | 3.453 |
| [c] 4000 Hx | 190 | 13.36 | 204 | −8.16 | 3.351 |
| [c] 6000 Hx | 190 | 6.81 | 206 | −4.24 | 3.661 |
| [c] 4000 Hy | 190 | 13.11 | 204 | −9.04 | 3.590 |
| [c] 6000 Hy | 190 | 6.68 | 206 | −4.74 | 3.740 |
| [c] 4000 Jo | 196 | 9.24 | 206 | −7.24 | 3.463 |
| [c] 6000 Jo | 194 | 4.14 | 208 | −3.55 | 4.698 |
| [c] 4000 Jx | 196 | 8.73 | 206 | −7.19 | 3.456 |
| [c] 6000 Jx | 194 | 3.92 | 208 | −3.60 | 4.755 |
| [c] 4000 Jy | 196 | 10.10 | 208 | −10.21 | 4.295 |
| [c] 6000 Jy | 196 | 4.43 | 210 | −4.86 | 4.515 |
| [d] B09:1 | 192 | 12.80 | 210 | −7.47 | 1.205 |
| [e] B09:2 | 192 | 11.93 | 210 | −8.21 | 0.933 |
| [f] B09:3 | 191 | 11.44 | 209 | −8.88 | 1.281 |

[a] SRCD spectrum from PCDDB (CD0000013000) [44,47]; [b] CDALC using PDB structure 1LIN minimized via NAMD/CHARMM22 for 5000 steps. Hydrogens attached to $-CH_3$ groups are ignored; [c] CAPPS using PDB structure 1LIN ignoring residues 3−5, 27−28, 100−101, and 146−148; [d] Matrix method using *ab initio* parameters including only the protein backbone transitions on PDB code 1LIN [55]; [e] Matrix method using *ab initio* parameters including protein backbone and charge-transfer transitions on PDB code 1LIN [55]; [f] Matrix method using *ab initio* parameters including protein backbone, charge-transfer and side chain transitions on PDB code 1LIN [55]; Grey highlight represents the lowest RMSD for each category.

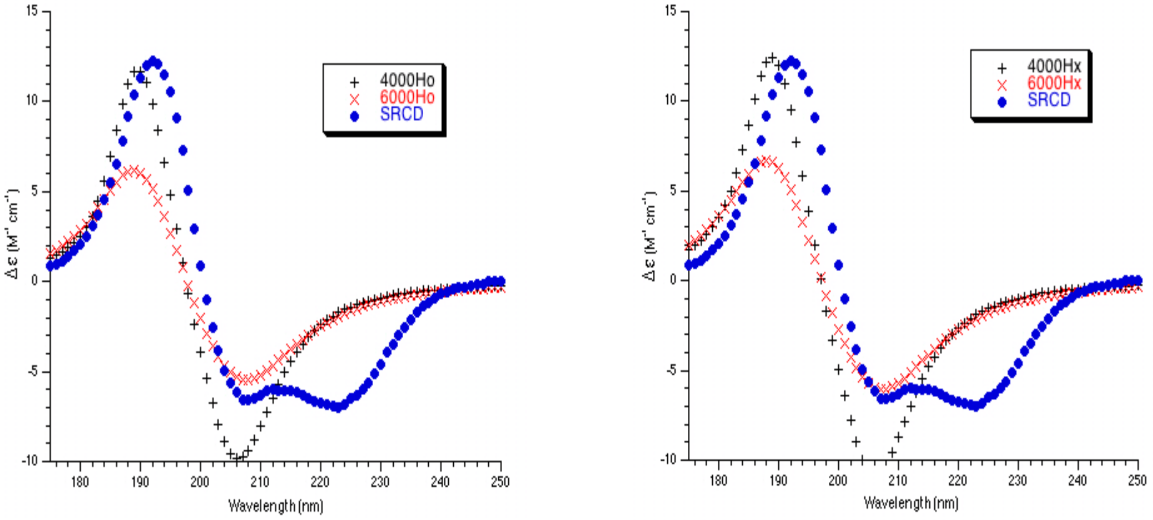


**Figure S13.** *Cont.*

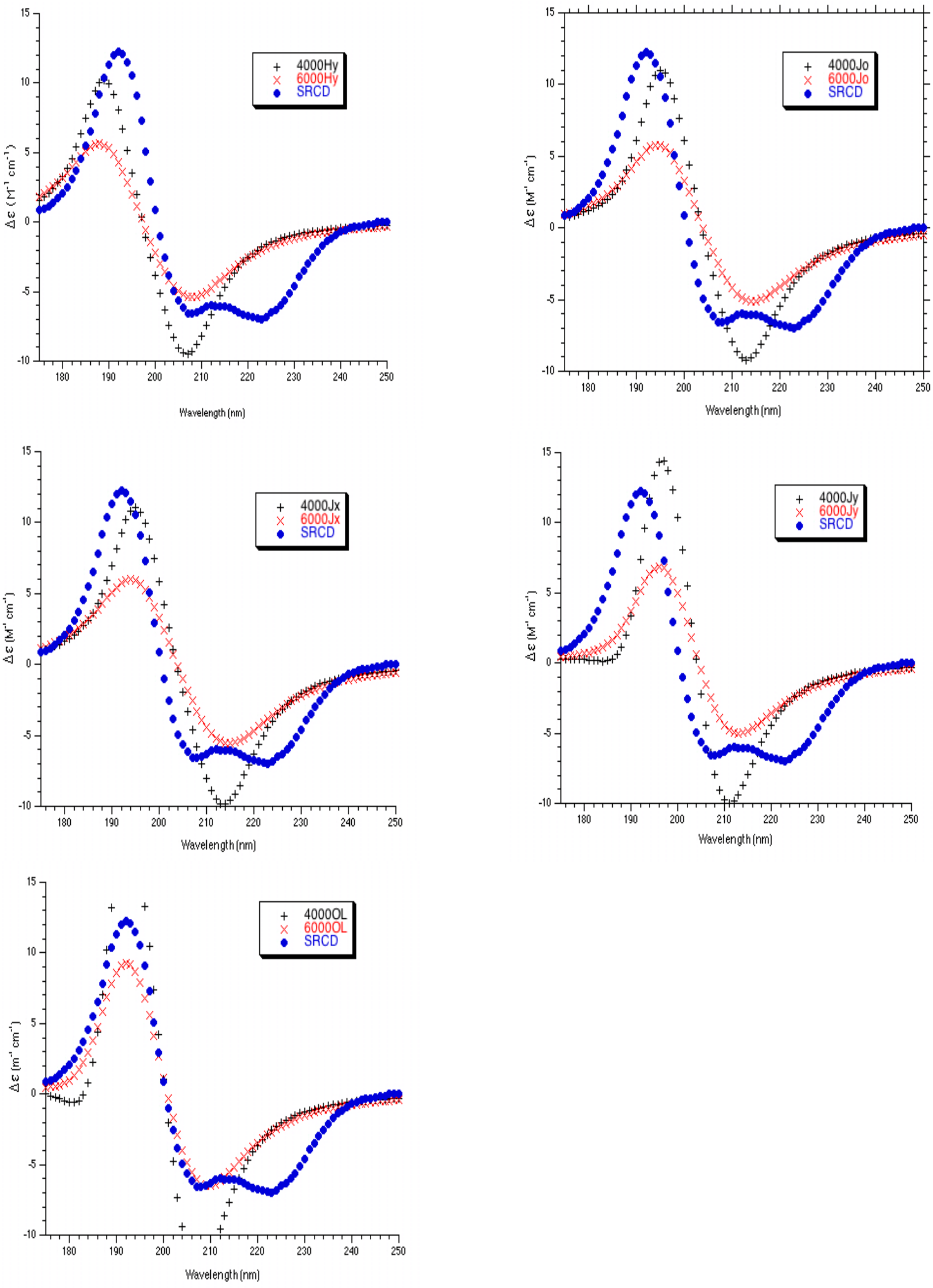


**Figure S13.** Calmodulin Predicted CD Using CDCALC. The PDB 1LIN [77] structure was minimized via 5000 conjugate gradients steps with NAMD/CHARMM22. Calculated spectra ignore all $-CH_3$ group hydrogens. Bandwidths are 4000 (+) and 6000 (×) $cm^{-1}$. The blue dots (•) are the experimental SRCD (CD0000013000) [44,47]. The CATH fold classification [53] is mainly alpha/orthogonal bundle.

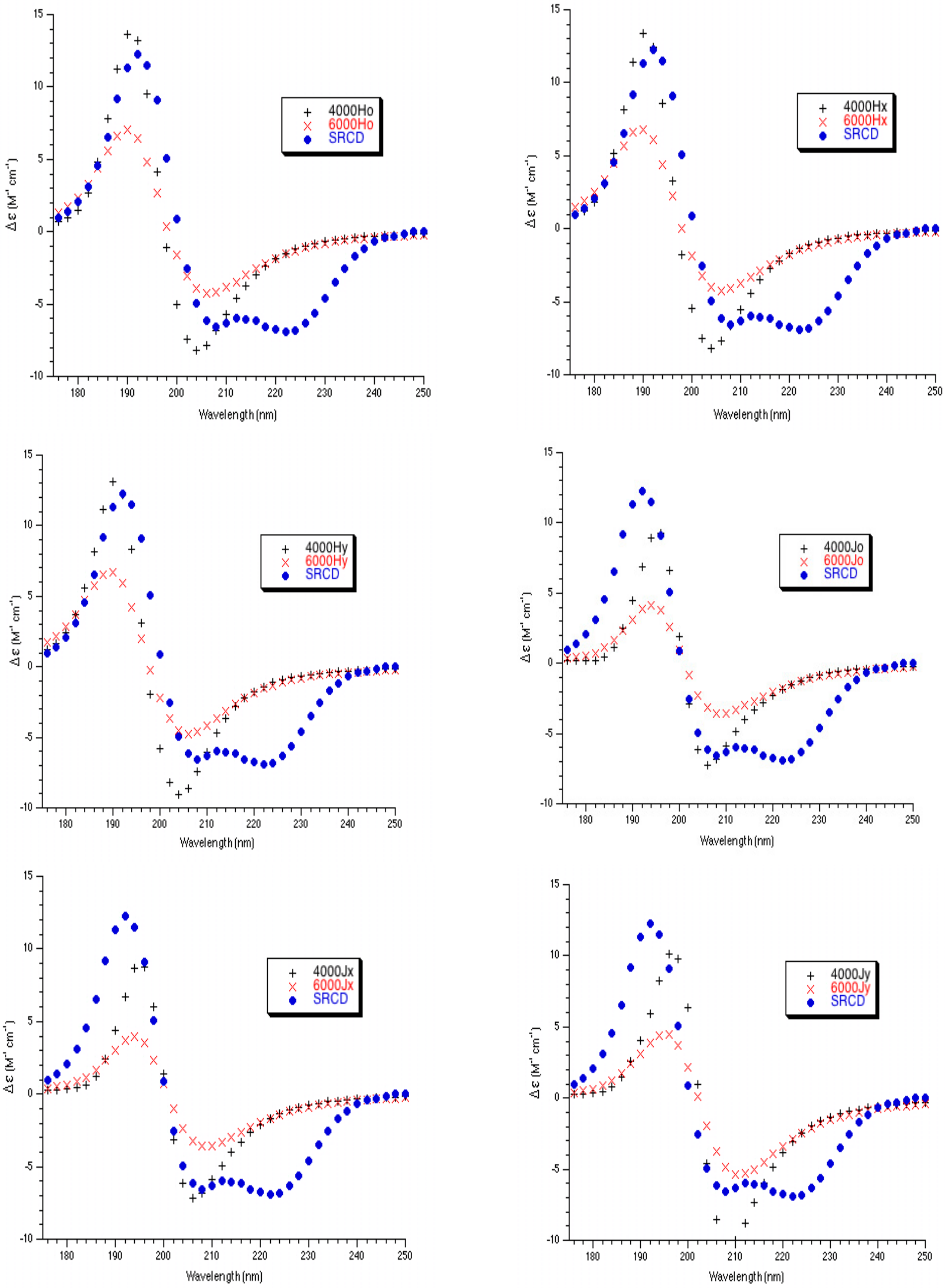


**Figure S14.** Calmodulin Predicted CD Using CAPPS. Calculated spectra of rebuilt PDB code 1LIN included full secondary structures including all hydrogen atoms; only the following residues were ignored: 3–5, 27–28, 100–101, and 146–148. Bandwidths are 4000 (+) and 6000 (×) $cm^{-1}$. The blue dots (•) are the experimental SRCD (CD0000013000) [44,47]. The CATH fold classification [53] is mainly alpha/ orthogonal bundle.

Leptin: The human obesity protein leptin (PDB code 1AX8 [84]) is a fairly small monomeric protein of 146 amino acids (Figure S15). Leptin needs to be present in the central nervous system in appropriate concentrations to act as a weight-lowering hormone [84]. The leptin 1AX8 structure is classified in the PCDDB as 56.2% α-helix, 4.8% $3_{10}$-helix, 1.4% β-bridge, 3.4% bonded turn, 4.8% bend, and 29.5% irregular [44] (Figure S15).

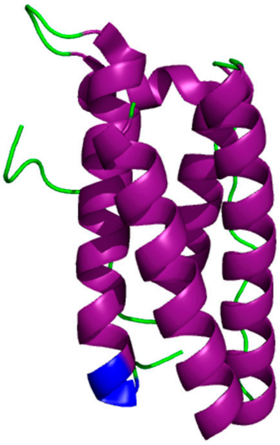

**Figure S15.** Leptin Secondary Structure. The PDB 1AX8 [84] structure showing secondary structure elements: thick purple cartoons/coils correspond to α-helices (4–23, 51–67, 71–94, 120–140, 106–115), the short blue cartoons/coil correspond to $3_{10}$–helices (141–143) and the thin green ropes are turns and other structures. The CATH fold classification [53] is mainly alpha/up-down bundle.

**Table S6.** CD Analysis of Leptin. The DInaMo calculations are for the minimized or rebuilt structure using CDCALC or CAPPS. All RMSDs are calculated between 180 and 210 nm.

| CD Method | Peak Wavelength (nm) | Δε ($M^{-1}\cdot cm^{-1}$) | Peak Wavelength (nm) | Δε ($M^{-1}\cdot cm^{-1}$) | RMSD ($M^{-1}\cdot cm^{-1}$) |
|---|---|---|---|---|---|
| [a] SRCD | 191 | 13.20 | 207 | −7.48 | 0.000 |
| [b] 4000 Ho | 190 | 15.00 | 207 | −10.79 | 3.296 |
| [b] 6000 Ho | 190 | 7.52 | 208 | −5.91 | 3.972 |
| [b] 4000 Hx | 190 | 15.26 | 207 | −11.60 | 3.615 |
| [b] 6000 Hx | 189 | 7.99 | 208 | −6.40 | 3.819 |
| [b] 4000 Hy | 190 | 12.20 | 207 | −10.16 | 3.211 |
| [b] 6000 Hy | 189 | 6.31 | 208 | −5.70 | 4.531 |
| [b] 4000 Jo | 196 | 14.02 | 214 | −10.09 | 5.860 |
| [b] 6000 Jo | 195 | 7.06 | 215 | −5.54 | 5.531 |
| [b] 4000 Jx | 196 | 13.56 | 214 | −10.48 | 5.580 |
| [b] 6000 Jx | 195 | 7.13 | 216 | −5.79 | 5.340 |
| [b] 4000 Jy | 197 | 18.22 | 212 | −11.26 | 8.142 |
| [b] 6000 Jy | 197 | 8.45 | 214 | −5.61 | 6.155 |
| [b] 4000 OL | 192 | 24.33 | 208 | −14.19 | 6.289 |
| [b] 6000 OL | 192 | 12.16 | 210 | −7.17 | 2.071 |

**Table S6.** *Cont.*

| CD Method | Peak Wavelength (nm) | Δε ($M^{-1}\cdot cm^{-1}$) | Peak Wavelength (nm) | Δε ($M^{-1}\cdot cm^{-1}$) | RMSD ($M^{-1}\cdot cm^{-1}$) |
|---|---|---|---|---|---|
| [c] 4000 Ho | 192 | 22.90 | 206 | −17.32 | 6.600 |
| [c] 6000 Ho | 190 | 10.92 | 208 | −8.96 | 2.276 |
| [c] 4000 Hx | 192 | 21.80 | 206 | −17.21 | 6.532 |
| [c] 6000 Hx | 190 | 10.69 | 208 | −8.88 | 2.602 |
| [c] 4000 Hy | 192 | 21.21 | 206 | −18.99 | 6.993 |
| [c] 6000 Hy | 190 | 10.58 | 208 | −10.21 | 2.938 |
| [c] 4000 Jo | 196 | 17.75 | 208 | −14.76 | 6.963 |
| [c] 6000 Jo | 196 | 6.72 | 210 | −7.20 | 5.174 |
| [c] 4000 Jx | 196 | 16.56 | 208 | −14.23 | 6.493 |
| [c] 6000 Jx | 194 | 6.19 | 210 | −7.02 | 5.161 |
| [c] 4000 Jy | 198 | 20.99 | 210 | −20.76 | 9.660 |
| [c] 6000 Jy | 196 | 8.25 | 212 | −10.53 | 5.552 |
| [d] SI | 192 | 13.40 | 209 | −10.85 | 2.437 |
| [e] SII | 192 | 6.82 | 208 | −3.52 | 5.908 |
| [f] SIII | 191 | 4.21 | 201 | −2.18 | 8.328 |

[a] SRCD (CD0000044000) from the Protein Circular Dichroism Data Bank [44,47]; [b] CDALC using PDB code 1AX8 [84] minimized by NAMD/CHARMM22 10,000 steps. Hs attached to $-CH_3$s are ignored; [c] CAPPS, Rebuilt PDB 1AX8 [84] structures ignoring residues 3, 24–50, 68–70, and 144–146. All hydrogens included; [d] Exciton Hamiltonian with Electrostatic Fluctuations (EHEF) method SI (a single conformation) using PDB code 1AX8 [29,84]; [e] EHEF method SII (based on 2000 MD snapshots) using PDB code 1AX8 [23]; [f] EHEF method SIII (uses only peptide groups) using PDB code 1AX8 [29,84];Grey highlight represents the smallest RMSD for a set of calculations.

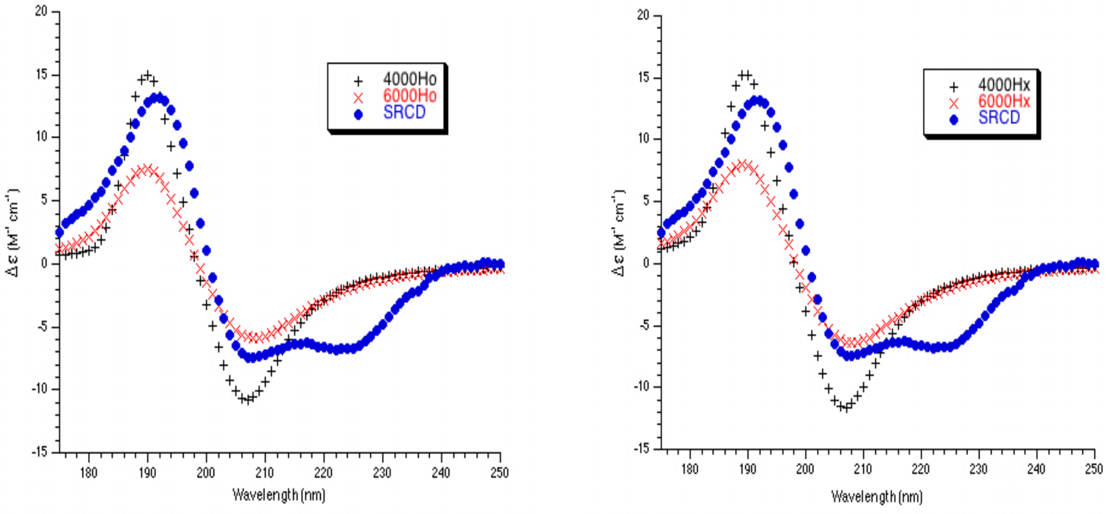


**Figure S16.** *Cont.*

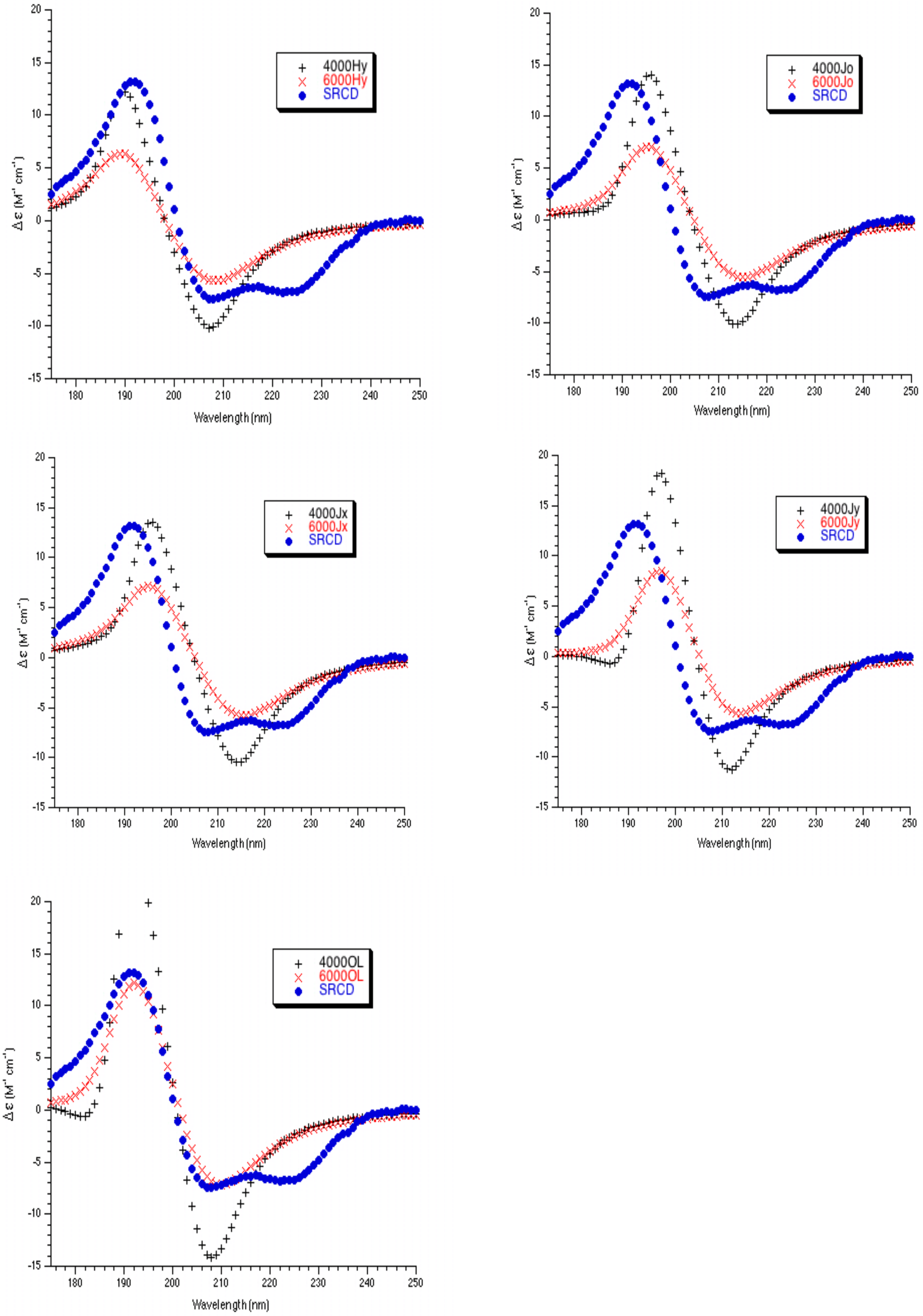


**Figure S16.** Leptin Predicted CD Using CDCALC. The PDB 1AX8 [84] structure was minimized via 10,000 conjugate gradient steps with NAMD/CHARMM22. Calculated spectra where all −$CH_3$ group hydrogens are ignored. Bandwidths are 4000 (+) and 6000 (×) $cm^{-1}$. The blue dots (•) are the experimental SRCD (CD0000044000) [1,2]. The CATH fold classification [53] is mainly alpha/up-down bundle.

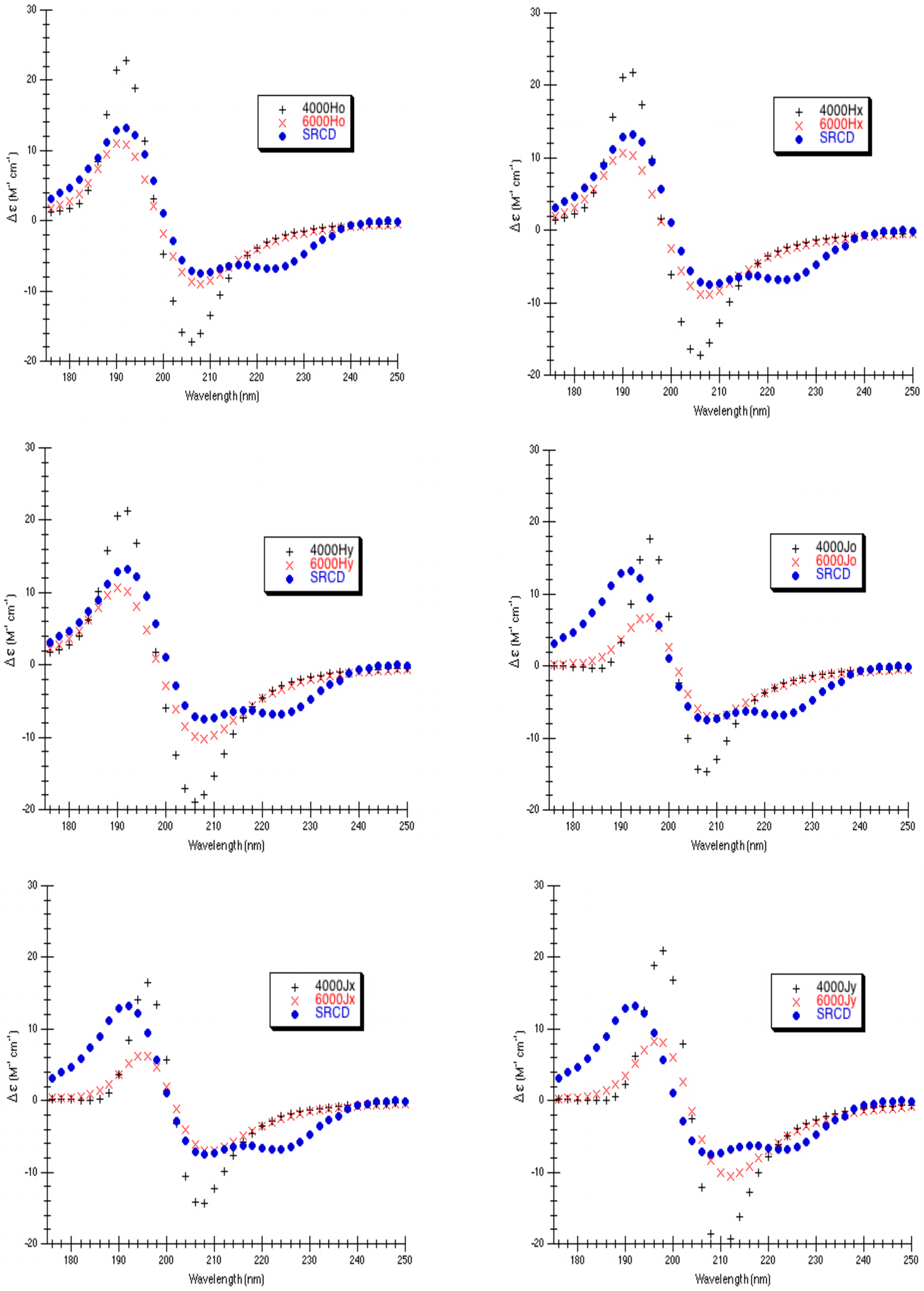


**Figure S17.** Leptin Predicted CD Using CAPPS. The PDB 1AX8 [84] structure was rebuilt with idealized bond lengths and angles. Residues 3, 24–50, 68–70, and 144–146 were ignored. Bandwidths are 4000 (+) and 6000 (×) $cm^{-1}$. The blue dots (•) are the experimental SRCD (CD0000044000) [44,47]. The CATH fold classification [53] is mainly alpha/up-down bundle.

Bacteriorhodopsin: The PDB structure 1QHJ [75] is a larger monomeric protein of 248 amino acids that acts as a proton pump converting light energy into a proton gradient driving ATP synthesis (Figure S18). The PCDDB describes the secondary structure of bacteriorhodopsin as 68.5% α-helix, 1.2% $3_{10}$-helix, 4.8% β-strand, 8.5% bonded turn, 3.2% bend, and 13.7% irregular [44]. CATH classifies it as a single domain that is mainly alpha/up-down bundle [53].

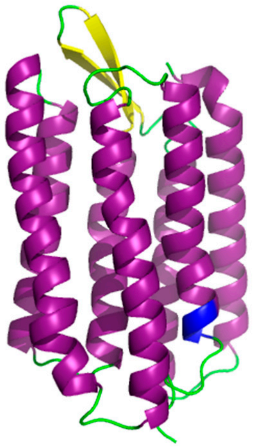

**Figure S18.** Bacteriorhodopsin Secondary Structure. The PDB 1QHJ [75] structure showing secondary structure elements: thick purple cartoons/coils correspond to α-helices (6–32, 37–58, 80–100, 105–127, 131–160, 165–191, 201–221), the short blue cartoons/coil correspond to $3_{10}$-helices (222–224) the yellow tapes are β-sheets, (66–71, 74–79) and the thin green ropes are turns and other structures. The CATH fold classification [53] is mainly alpha/up-down bundle.

**Table S7.** CD Analysis of Bacteriorhodopsin. The DInaMo calculations are for the minimized or rebuilt structure using CDCALC or CAPPS. All RMSDs are calculated between 180 and 210 nm.

| CD Method | Peak Wavelength (nm) | Δε ($M^{-1}\cdot cm^{-1}$) | Peak Wavelength (nm) | Δε ($M^{-1}\cdot cm^{-1}$) | RMSD ($M^{-1}\cdot cm^{-1}$) |
|---|---|---|---|---|---|
| [a] SRCD | 195 | 15.67 | 214 | −5.20 | 0.000 |
| [b] 4000Ho | 190 | 20.67 | 207 | −14.62 | 9.380 |
| [b] 6000Ho | 190 | 10.80 | 209 | −7.97 | 6.844 |
| [b] 4000Hx | 189 | 20.24 | 207 | −16.11 | 10.337 |
| [b] 6000Hx | 189 | 10.57 | 208 | −9.02 | 7.611 |
| [b] 4000Hy | 190 | 16.69 | 208 | −14.03 | 8.831 |
| [b] 6000Hy | 189 | 8.80 | 209 | −7.93 | 7.322 |
| [b] 4000Jo | 195 | 19.41 | 214 | −13.71 | 2.664 |
| [b] 6000Jo | 195 | 10.21 | 215 | −7.49 | 3.179 |
| [b] 4000Jx | 196 | 17.54 | 215 | −14.52 | 2.424 |
| [b] 6000Jx | 195 | 9.17 | 216 | −8.21 | 3.758 |
| [b] 4000Jy | 198 | 22.32 | 212 | −14.97 | 6.513 |
| [b] 6000Jy | 197 | 10.48 | 214 | −7.58 | 4.347 |
| [b] 4000OL | 192 | 29.59 | 208 | −17.90 | 9.902 |
| [b] 6000OL | 192 | 14.27 | 210 | −9.63 | 4.469 |

**Table S7.** *Cont.*

| CD Method | Peak Wavelength (nm) | Δε ($M^{-1}\cdot cm^{-1}$) | Peak Wavelength (nm) | Δε ($M^{-1}\cdot cm^{-1}$) | RMSD ($M^{-1}$ $cm^{-1}$) |
|---|---|---|---|---|---|
| [c] 4000Ho | 192 | 20.25 | 206 | −17.39 | 10.341 |
| [c] 6000Ho | 190 | 10.45 | 208 | −9.20 | 7.195 |
| [c] 4000Hx | 190 | 20.03 | 206 | −17.30 | 10.812 |
| [c] 6000Hx | 190 | 10.39 | 206 | −9.16 | 7.589 |
| [c] 4000Hy | 190 | 19.01 | 206 | −18.95 | 11.252 |
| [c] 6000Hy | 190 | 9.90 | 208 | −10.26 | 8.067 |
| [c] 4000Jo | 196 | 16.40 | 208 | −14.73 | 6.915 |
| [c] 6000Jo | 194 | 6.85 | 210 | −7.65 | 6.230 |
| [c] 4000Jx | 196 | 15.68 | 208 | −14.64 | 7.046 |
| [c]6000Jx | 194 | 6.58 | 210 | −7.56 | 6.404 |
| [c] 4000Jy | 196 | 19.23 | 210 | −20.74 | 7.586 |
| [c] 6000Jy | 196 | 8.21 | 212 | −10.99 | 5.484 |
| [d] 4000Hy | 192 | 24.95 | 207 | −18.42 | 9.952 |
| [d] 6000Hy | 191 | 12.11 | 208 | −9.61 | 5.985 |

[a] SRCD from the PCDDB Code CD0000101000 [44,59]; [b] CDCALC, PDB code 1QHJ [75] NAMD/CHARMM22 minimized for 10,000 conjugate gradient steps. The hydrogens on all −$CH_3$ groups are ignored; [c] CAPPS, Rebuilt PDB code 1QHJ [75] secondary structures only. The following residues were ignored: 5, 33–36, 101–104, 128–130, and 161–164. All hydrogens included; [d] CAPPS, Rebuilt PDB code 2BRD [106] published by Bode and Applequist [3]. Grey highlight represents the smallest RMSD for a set of calculations.

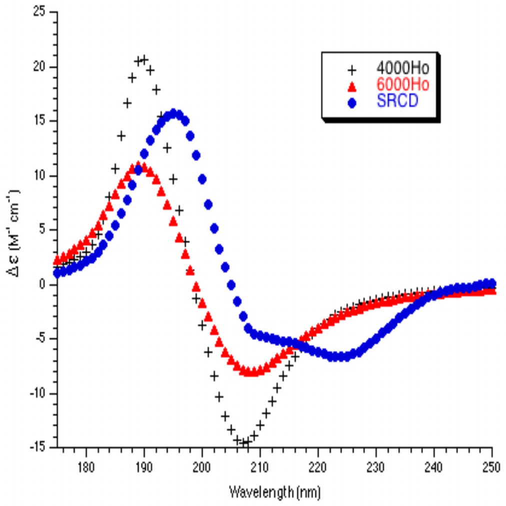


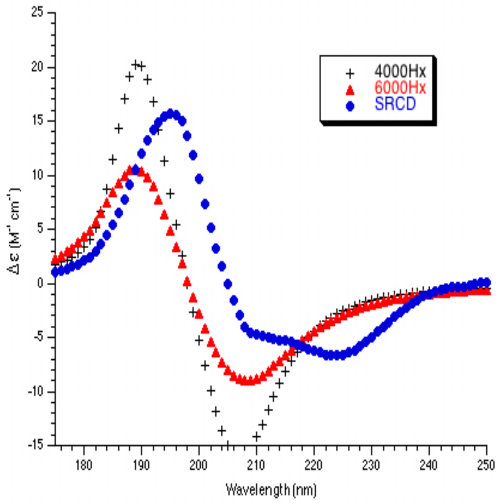


**Figure S19.** *Cont.*

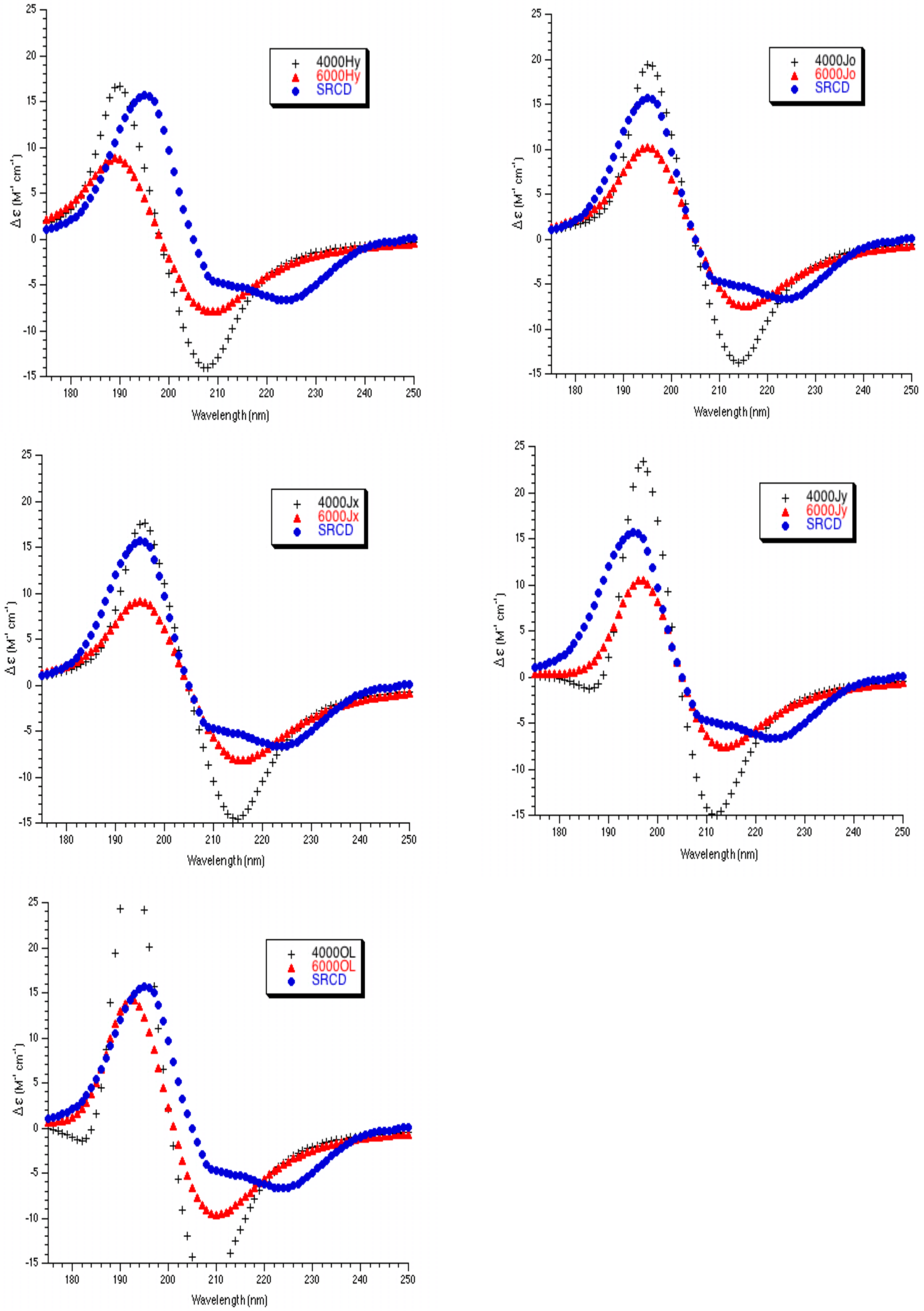


**Figure S19.** Bacteriorhodopsin Predicted CD Using CDCALC. The PDB 1QHJ [75] structure was minimized with 10,000 conjugate gradient steps using NAMD/CHARMM22. Calculated spectra ignore all $-CH_3$ group hydrogens. Bandwidths are 4000 (+) and 6000 (×) $cm^{-1}$. The blue dots (•) are the experimental SRCD (CD0000101000) [44,59]. The CATH fold classification [53] is mainly alpha/up-down bundle.

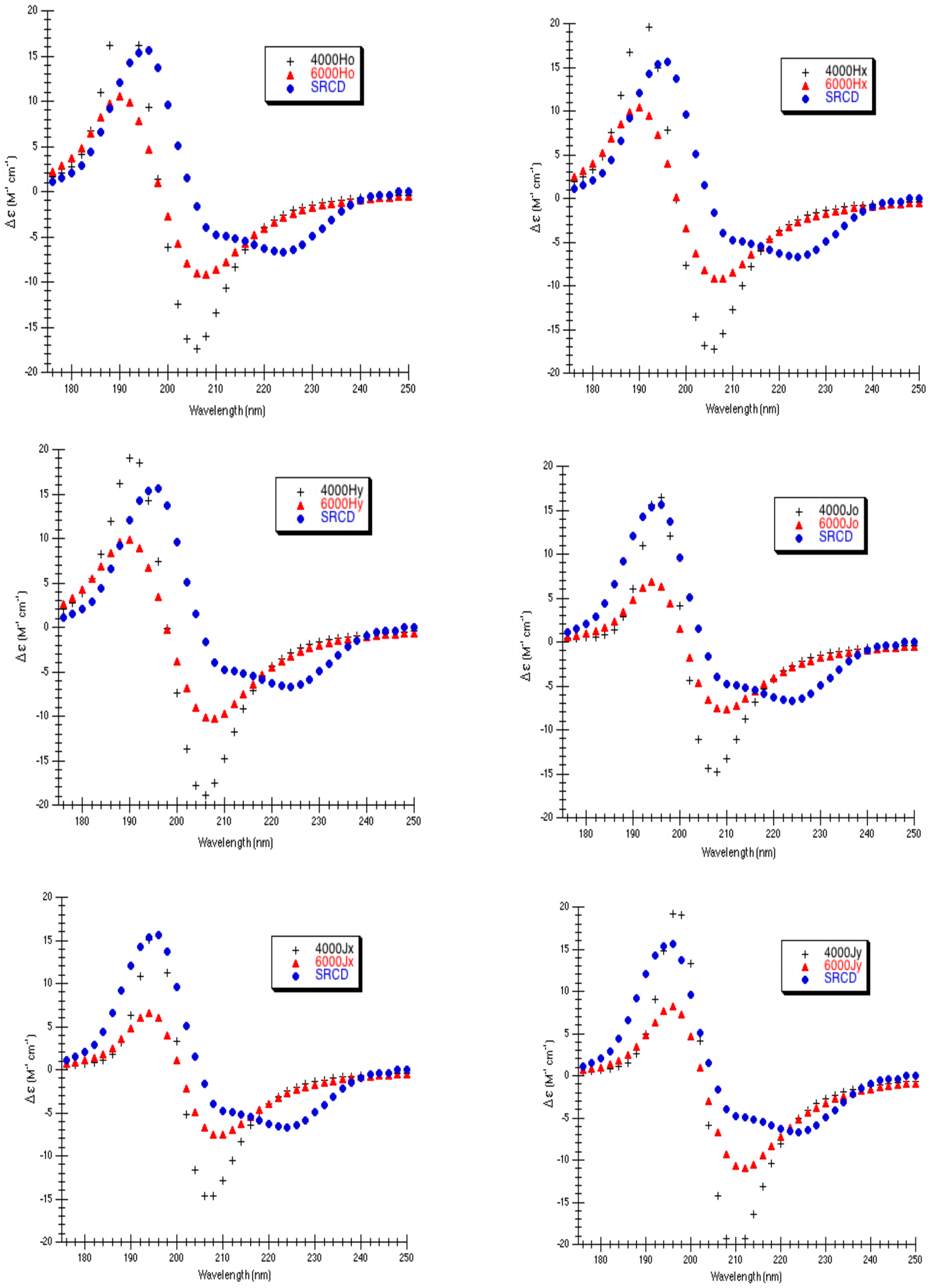


**Figure S20.** Bacteriorhodopsin Predicted CD Using CAPPS. The PDB 1QHJ [75] structure was rebuilt ignoring residues 5, 33–36, 101–104, 128–130, 161–164. Bandwidths are 4000 (+) and 6000 (×) $cm^{-1}$. The blue dots (•) are the experimental SRCD (CD0000101000) [44,59]. The CATH fold classification [53] is mainly alpha/up-down bundle.

Myoglobin: This monomeric oxygen transporter has multiple structures in the PDB that are high resolution. Furthermore, two different species have spectra in the PCDDB, so both species are treated with DInaMo. CATH classifies all the structures studied as mainly alpha/orthogonal bundle [53].

*Horse myoglobin*: Herein, both PDB code 3LR7 (Figure S21), which has nitrite bound to the ferric iron of the heme group [85], and 2V1K, which has a ferrous iron with nothing bound to the heme group [86], have extremely similar protein structures (eight α-helices and one $3_{10}$-helix). The secondary structures of horse myoglobin identified in the PCDDB are 73.9% α-helix, 13.1% bonded turn, 2.0% bend, and 11.1% irregular [44].

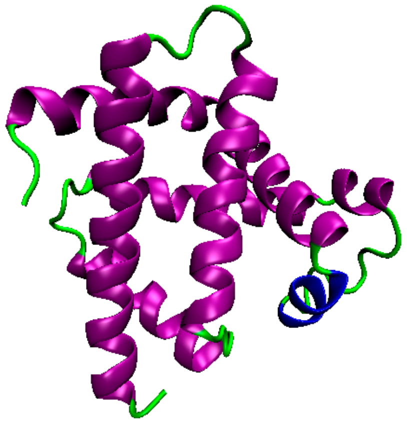

**Figure S21.** Horse Myoglobin Secondary Structure. PBD code 3LR7 [85] structure showing secondary structure elements: thick purple cartoons/coils correspond to α-helices (3–20, 20–36, 37–40, 51–58, 58–77, 82–96, 100–119, 124–150), the short blue cartoons/coil correspond to $3_{10}$−helices (41−43) the thin green ropes are turns and other structures. The CATH fold classification [53] is mainly alpha/orthogonal bundle.

**Table S8.** CD Analysis of Horse Myoglobin. The DInaMo calculations are for the minimized or rebuilt structure using CDCALC or CAPPS. All RMSDs are calculated between 180 and 210 nm.

| CD Method | Peak Wavelength (nm) | Δε ($M^{-1}\cdot cm^{-1}$) | Peak Wavelength (nm) | Δε ($M^{-1}\cdot cm^{-1}$) | RMSD ($M^{-1}\cdot cm^{-1}$) |
|---|---|---|---|---|---|
| [a] SRCD | 192 | 16.75 | 209 | −7.51 | 0.000 |
| [b] 4000 Ho | 189 | 30.16 | 203 | −17.40 | 10.913 |
| [b] 6000 Ho | 189 | 15.49 | 205 | −8.46 | 5.609 |
| [b] 4000 Hx | 189 | 30.51 | 203 | −17.82 | 11.345 |
| [b] 6000 Hx | 188 | 15.93 | 205 | −8.76 | 5.840 |
| [b] 4000 Hy | 188 | 26.00 | 203 | −18.22 | 11.530 |
| [b] 6000 Hy | 188 | 13.81 | 205 | −9.31 | 7.148 |
| [b] 4000 Jo | 195 | 28.36 | 210 | −16.34 | 7.312 |
| [b] 6000 Jo | 194 | 14.53 | 212 | −7.93 | 2.990 |
| [b] 4000 Jx | 195 | 27.10 | 211 | −16.09 | 6.899 |
| [b] 6000 Jx | 194 | 14.30 | 213 | −7.94 | 3.139 |
| [b] 4000 Jy | 195 | 31.65 | 208 | −18.76 | 9.788 |

**Table S8.** *Cont.*

| CD Method | Peak Wavelength (nm) | Δε ($M^{-1}·cm^{-1}$) | Peak Wavelength (nm) | Δε ($M^{-1}·cm^{-1}$) | RMSD ($M^{-1}·cm^{-1}$) |
|---|---|---|---|---|---|
| [b] 6000 Jy | 195 | 14.66 | 210 | −8.69 | 3.820 |
| [b] 4000 OL | 193 | 46.39 | 206 | −16.53 | 14.244 |
| [b] 6000 OL | 192 | 23.89 | 209 | −6.69 | 3.542 |
| [c] 4000 Ho | 192 | 11.65 | 208 | −13.65 | 6.839 |
| [c] 6000 Ho | 188 | 8.44 | 208 | −7.83 | 6.734 |
| [c] 4000 Hx | 187 | 15.49 | 207 | −14.98 | 7.823 |
| [c] 6000 Hx | 187 | 9.05 | 208 | −8.69 | 7.050 |
| [c] 4000 Hy | 188 | 13.40 | 208 | −12.86 | 7.206 |
| [c] 6000 Hy | 187 | 7.60 | 206 | −7.34 | 7.296 |
| [c] 4000 Jo | 195 | 14.65 | 213 | −12.75 | 3.521 |
| [c] 6000 Jo | 194 | 7.92 | 215 | −7.33 | 5.359 |
| [c] 4000 Jx | 194 | 14.58 | 214 | −13.65 | 2.991 |
| [c] 6000 Jx | 193 | 8.21 | 215 | −7.96 | 5.045 |
| [c] 4000 Jy | 197 | 18.45 | 212 | −13.26 | 6.616 |
| [c] 6000 Jy | 196 | 8.96 | 214 | −6.95 | 6.120 |
| [c] 4000 OL | 192 | 23.51 | 208 | −16.79 | 5.941 |
| [c] 6000 OL | 192 | 11.65 | 210 | −9.35 | 3.938 |
| [d] 4000 Ho | 190 | 20.28 | 206 | −15.75 | 6.324 |
| [d] 6000 Ho | 190 | 10.78 | 208 | −8.29 | 4.946 |
| [d] 4000 Hx | 190 | 20.32 | 206 | −15.84 | 6.839 |
| [d] 6000 Hx | 190 | 10.75 | 208 | −8.32 | 5.295 |
| [d] 4000 Hy | 190 | 19.20 | 206 | −17.55 | 7.486 |
| [d] 6000 Hy | 190 | 10.15 | 208 | −9.60 | 5.881 |
| [d] 4000 Jo | 196 | 17.60 | 208 | −12.65 | 6.414 |
| [d] 6000 Jo | 196 | 7.24 | 210 | −6.16 | 6.439 |
| [d] 4000 Jx | 194 | 16.95 | 208 | −12.63 | 6.112 |
| [d] 6000 Jx | 194 | 6.95 | 210 | −6.19 | 6.428 |
| [d] 4000 Jy | 198 | 20.32 | 210 | −18.01 | 8.261 |
| [d] 6000 Jy | 192 | 8.62 | 212 | −9.29 | 6.335 |
| [g] MM1 | 192 | 16.80 | 211 | −11.36 | 3.131 |
| [h] MM2 | 190 | 16.75 | 210 | −12.07 | 3.716 |
| [i] MM3 | 191 | 13.43 | 209 | −11.78 | 4.797 |

[a] SRCD from the Protein Circular Dichroism Data Bank for horse myoglobin CD0000047000 [44,47]; [b] CDALC using PDB code 3LR7 [85] minimized by Insight®II/Discover/CVFF 110,000 steps of steepest descents followed by 21,000 steps of conjugate gradients. All hydrogens are included; [c] CDCALC using PDB code 2V1K [86] minimized with NAMD/CHARMM22 5000 conjugate gradient steps. Hs attached to $-CH_3$s are ignored; [d] CAPPS, Rebuilt PDB code 2V1K [86] secondary structures including hydrogens. The following residues were ignored: 1–2, 19–21, 57–59, 97–99, and 151–153; [g] Matrix method with *ab initio* parameters using PDB code 1YMB [107] including only the protein backbone transitions [55]; [h] Matrix method with *ab initio* parameters using PDB code 1YMB [107] including protein backbone and charge-transfer transitions [55]; [i] Matrix method using with *ab initio* parameters PDB code 1YMB [107] including protein backbone, charge-transfer and side chain transitions [55]; Grey highlight represents the smallest RMSD for a set of calculations.

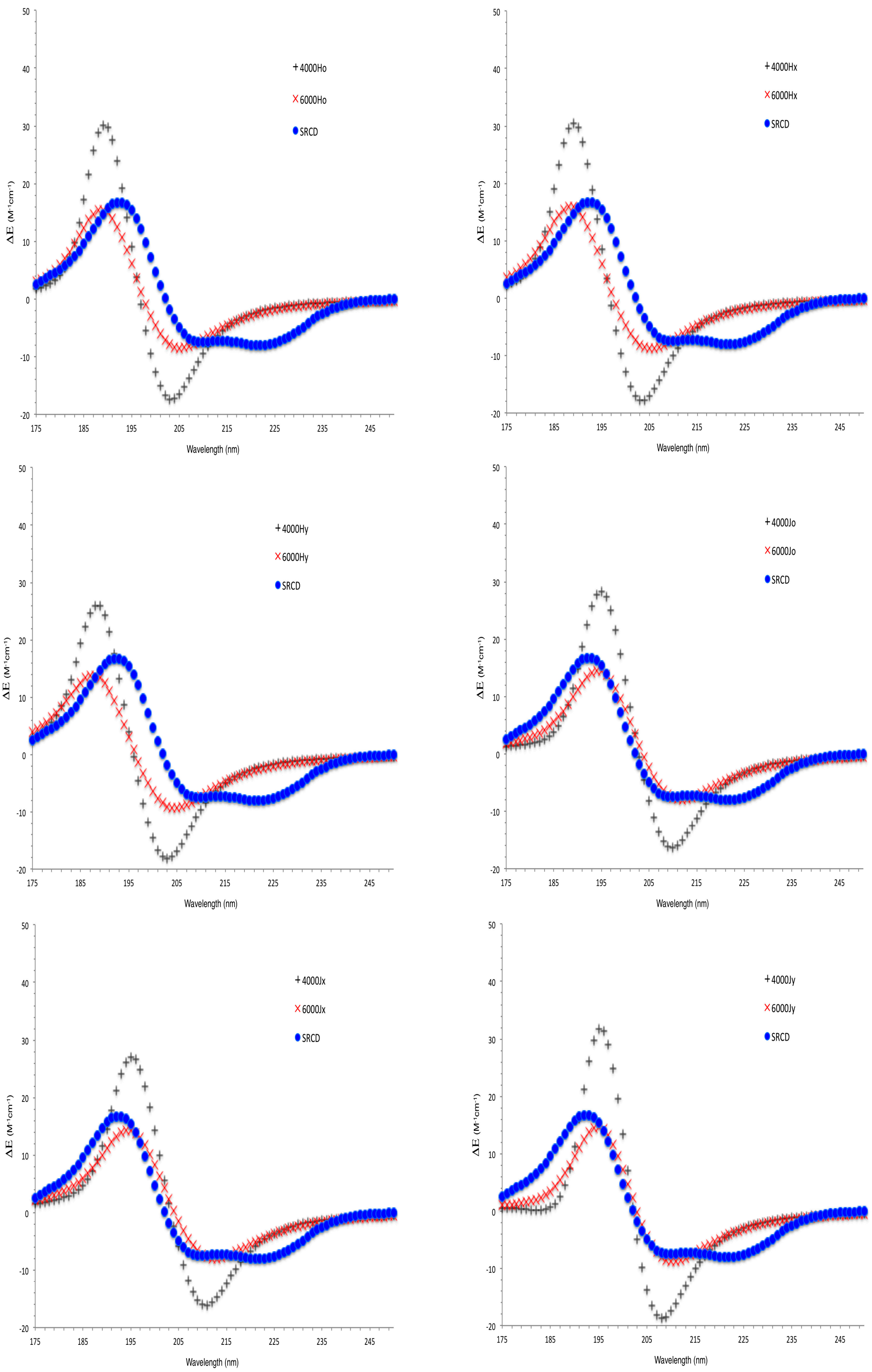


**Figure S22.** *Cont.*

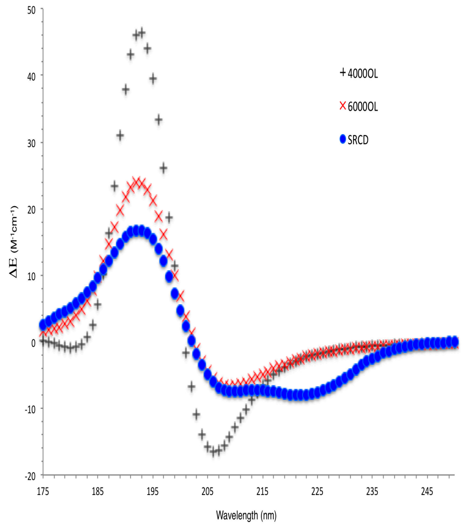


**Figure S22.** Horse Myoglobin Predicted CD Spectra Using CDCALC with Structure 3LRZ [86] Minimized by Insight®II/Discover. Calculated spectra include all hydrogens. Bandwidths are 4000 (+) and 6000 (×) $cm^{-1}$. The blue dots (•) are the experimental SRCD (CD0000047000) [44,47]. The CATH fold classification [53] is mainly alpha/orthogonal bundle.

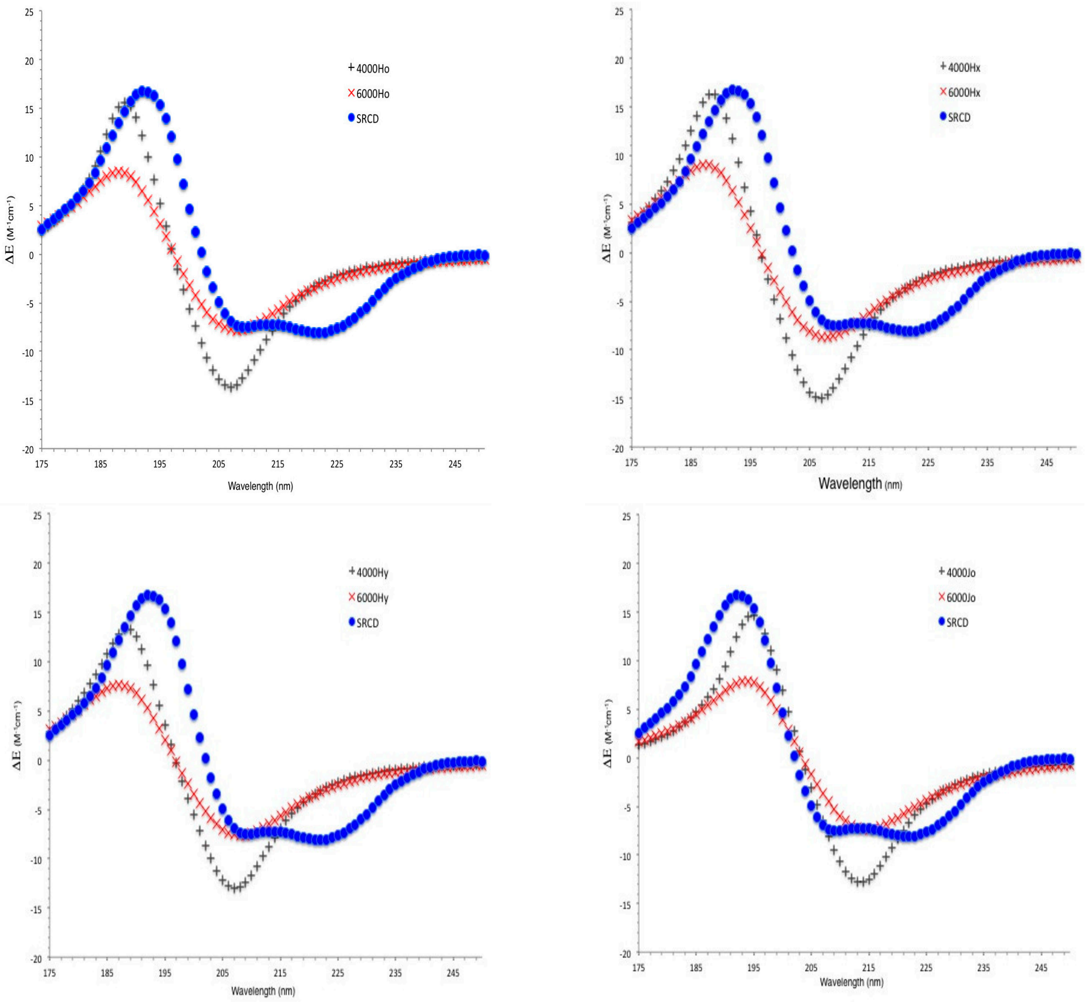


**Figure S23.** *Cont.*

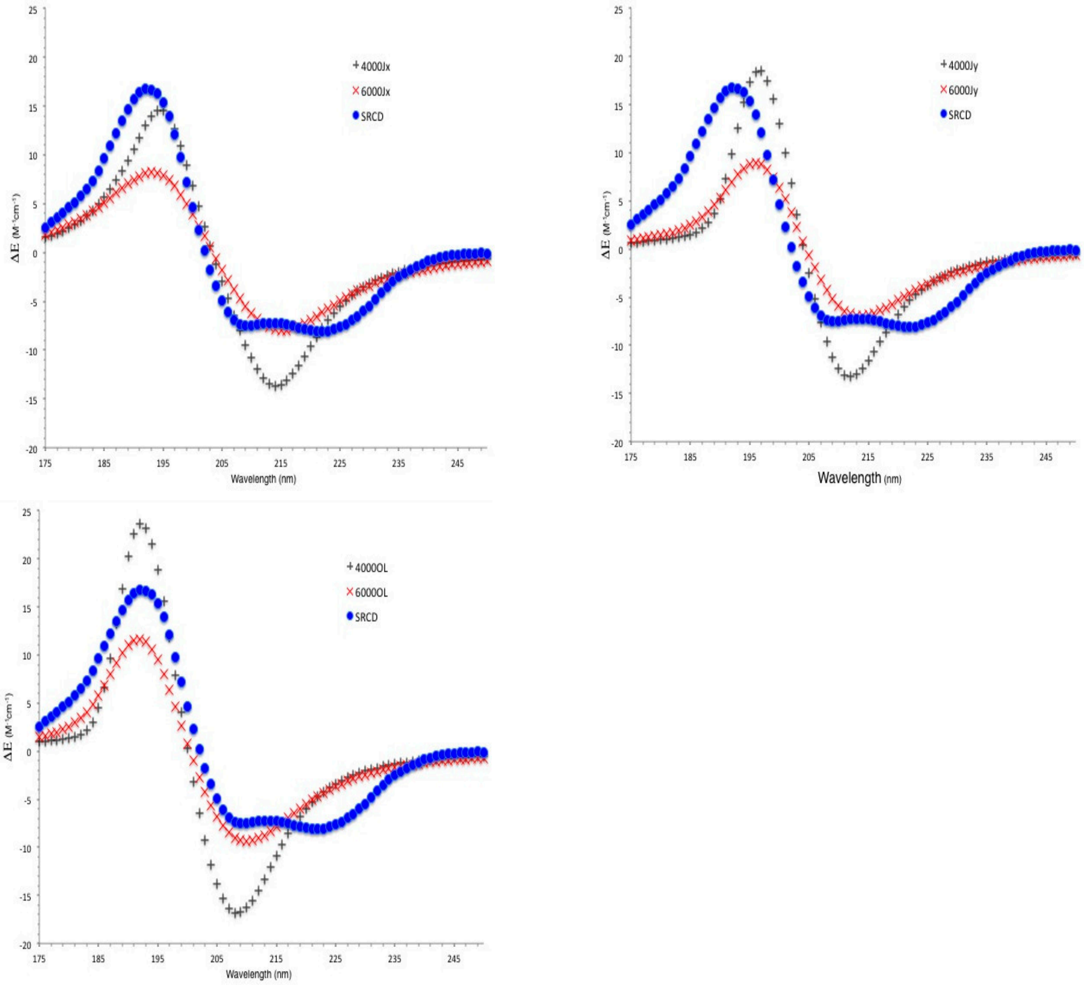


**Figure S23.** Horse Myoglobin Predicted CD Using CDCALC with Structure Minimized by NAMD/CHARMM22. PDB code 2V1K [86] was minimized by 5000 conjugate gradient steps. Calculated spectra ignore all $-CH_3$ group hydrogens. Bandwidths are 4000 (+) and 6000 (×) $cm^{-1}$. The blue dots (•) are the experimental SRCD (CD0000047000) [44,47]. The CATH fold classification [53] is mainly alpha/orthogonal bundle.

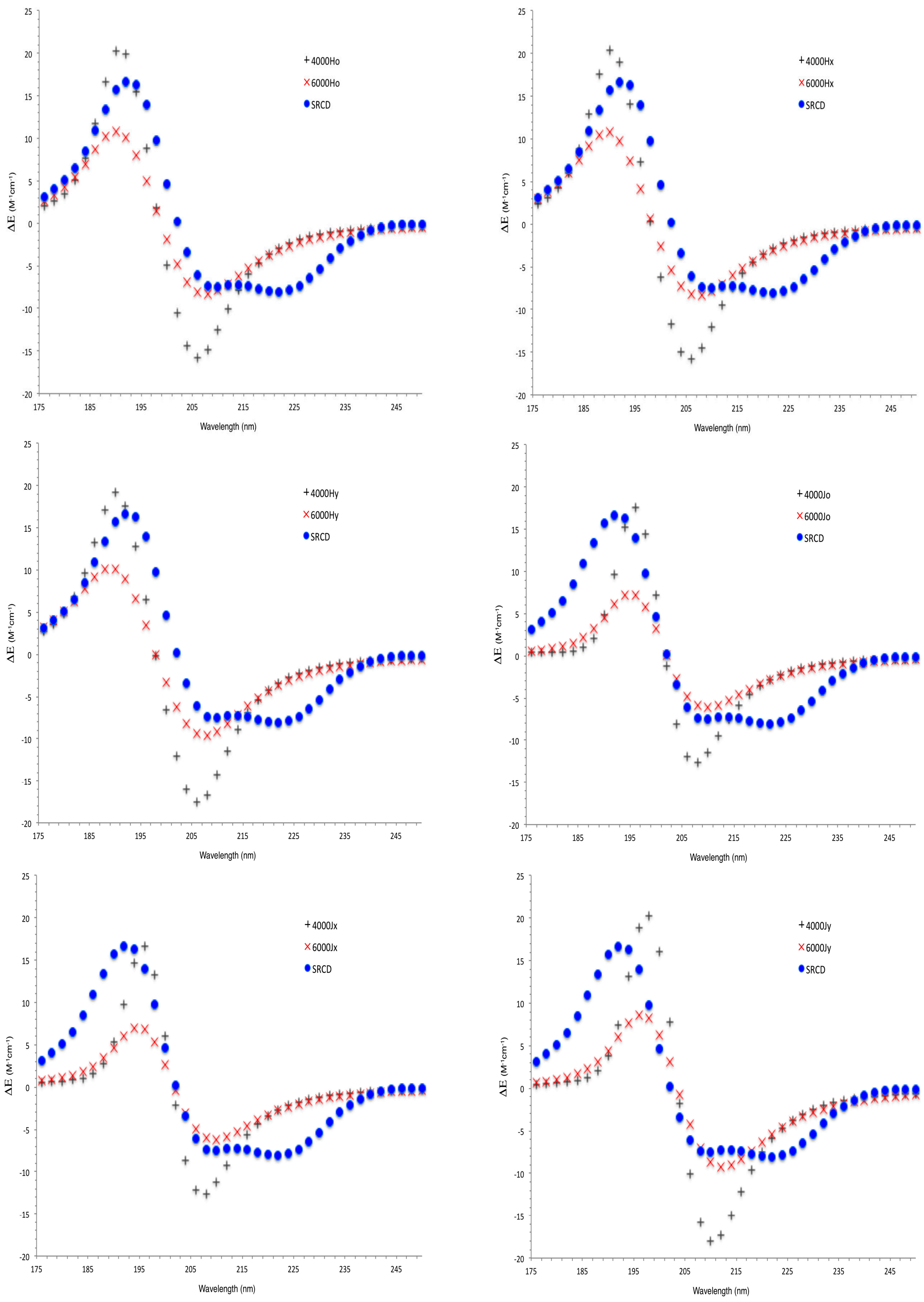


**Figure S24.** Horse Myoglobin Predicted CD Using CAPPS. 2V1K [86] is rebuilt structure ignoring residues 1–2, 19–21, 57–59, 97–99, and 151–153. All other structures are included with hydrogens. Bandwidths are 4000 (+) and 6000 (×) $cm^{-1}$. The blue dots (•) are the experimental SRCD (CD0000047000) [44,47]. The CATH fold classification [53] is mainly alpha/orthogonal bundle.

*Sperm whale myoglobin*: Sperm whale myoglobin (Figure S25) is very similar to horse myoglobin, but there are subtle differences. The PDB 2JHO structure has seven α-helices and one $3_{10}$-helix (Figure S25) [87], and the PCDDB describes the secondary structure as 74.2% α-helix, 4.0% $3_{10}$-helix, 9.3% bonded turn, 2.0% bend, and 10.6% irregular [44].

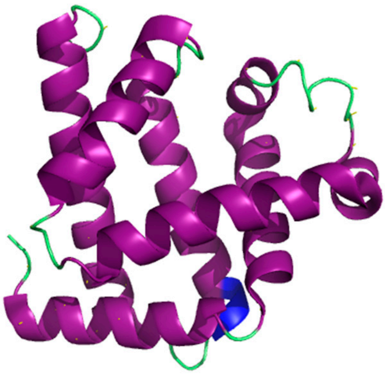

**Figure S25.** Sperm Whale Myoglobin Secondary Structure. PDB 2JHO [87] structure showing secondary structure elements: thick purple cartoons/coils correspond to α-helices (3–18, 20–36, 36–42, 51–57, 58–77, 82–96, 100–116, 124–150), the short blue cartoons/coil correspond to $3_{10}$-helices (117–119) and the thin green ropes are turns and other structures. The CATH fold classification [53] is mainly alpha/orthogonal bundle.

**Table S9.** CD Analysis of Sperm Whale Myoglobin. The DInaMo calculations are for the minimized or rebuilt structure using CDCALC or CAPPS. All RMSDs are calculated between 180 and 210 nm.

| CD Method | Peak Wavelength (nm) | Δε ($M^{-1}\cdot cm^{-1}$) | Peak Wavelength (nm) | Δε ($M^{-1}\cdot cm^{-1}$) | RMSD ($M^{-1}\cdot cm^{-1}$) |
|---|---|---|---|---|---|
| [a] SRCD | 193 | 17.33 | 210 | −7.77 | 0.000 |
| [b] 4000 Ho | 187 | 18.48 | 202 | −11.90 | 10.355 |
| [b] 6000 Ho | 186 | 19.38 | 204 | −6.07 | 8.344 |
| [b] 4000 Hx | 187 | 19.38 | 202 | −12.30 | 10.880 |
| [b] 6000 Hx | 186 | 10.94 | 204 | −6.34 | 8.513 |
| [b] 4000 Hy | 186 | 18.31 | 201 | −12.82 | 12.070 |
| [b] 6000 Hy | 185 | 10.30 | 203 | −7.00 | 9.577 |
| [b] 4000 Jo | 193 | 17.41 | 203 | −11.16 | 2.810 |
| [b] 6000 Jo | 192 | 9.65 | 210 | −5.71 | 4.681 |
| [b] 4000 Jx | 193 | 17.74 | 209 | −11.14 | 2.392 |
| [b] 6000 Jx | 192 | 10.06 | 211 | −5.80 | 4.386 |
| [b] 4000 Jy | 194 | 19.04 | 207 | −12.45 | 4.529 |
| [b] 6000 Jy | 193 | 9.11 | 209 | −5.93 | 5.282 |
| [b] 4000 OL | 191 | 31.24 | 205 | −10.97 | 7.133 |
| [b] 6000 OL | 191 | 16.49 | 208 | −4.60 | 2.456 |

**Table S9.** *Cont.*

| CD Method | Peak Wavelength (nm) | Δε ($M^{-1}·cm^{-1}$) | Peak Wavelength (nm) | Δε ($M^{-1}·cm^{-1}$) | RMSD ($M^{-1}·cm^{-1}$) |
|---|---|---|---|---|---|
| [c] 4000 Ho | 189 | 15.67 | 207 | −13.79 | 7.272 |
| [c] 6000 Ho | 188 | 8.54 | 208 | −7.82 | 7.178 |
| [c] 4000 Hx | 188 | 16.22 | 207 | −14.95 | 8.131 |
| [c] 6000 Hx | 188 | 9.02 | 208 | −8.56 | 7.469 |
| [c] 4000 Hy | 188 | 13.44 | 207 | −13.10 | 7.592 |
| [c] 6000 Hy | 187 | 7.66 | 208 | −7.58 | 7.715 |
| [c] 4000 Jo | 195 | 14.66 | 214 | −12.90 | 3.599 |
| [c] 6000 Jo | 193 | 8.00 | 215 | −7.32 | 5.672 |
| [c] 4000 Jx | 194 | 14.51 | 214 | −13.58 | 3.169 |
| [c] 6000 Jx | 193 | 8.17 | 215 | −7.81 | 5.449 |
| [c] 4000 Jy | 196 | 19.07 | 212 | −13.61 | 6.603 |
| [c] 6000 Jy | 196 | 9.35 | 210 | −5.95 | 6.232 |
| [c] 4000 OL | 192 | 24.22 | 208 | −17.21 | 6.122 |
| [c] 6000 OL | 192 | 12.28 | 210 | −9.29 | 3.988 |
| [d] 4000 Ho | 190 | 20.19 | 206 | −16.91 | 7.744 |
| [d] 6000 Ho | 188 | 10.88 | 208 | −9.02 | 5.779 |
| [d] 4000 Hx | 190 | 20.10 | 206 | −17.02 | 8.509 |
| [d] 6000 Hx | 188 | 11.11 | 206 | −9.27 | 6.266 |
| [d] 4000 Hy | 188 | 19.18 | 206 | −18.92 | 9.444 |
| [d] 6000 Hy | 188 | 10.74 | 206 | −10.62 | 7.053 |
| [d] 4000 Jo | 196 | 17.42 | 208 | −13.49 | 6.023 |
| [d] 6000 Jo | 194 | 7.54 | 210 | −6.61 | 6.096 |
| [d] 4000 Jx | 196 | 16.45 | 208 | −13.51 | 5.802 |
| [d] 6000 Jx | 194 | 7.28 | 210 | −6.68 | 6.097 |
| [d] 4000 Jy | 196 | 17.91 | 210 | −19.55 | 7.716 |
| [d] 6000 Jy | 196 | 8.87 | 212 | −10.26 | 5.742 |
| [e] B09:1 | 191 | 16.86 | 209 | −12.00 | 3.209 |
| [f] B09:2 | 191 | 16.13 | 209 | −13.17 | 4.091 |
| [g] B09:3 | 190 | 13.23 | 209 | −13.37 | 5.424 |

**Table S9.** *Cont.*

| CD Method | Peak Wavelength (nm) | Δε ($M^{-1}·cm^{-1}$) | Peak Wavelength (nm) | Δε ($M^{-1}·cm^{-1}$) | RMSD ($M^{-1}·cm^{-1}$) |
|---|---|---|---|---|---|
| [h] HCG03:1 | 190 | 9.48 | 214 | −7.73 | 4.415 |
| [i] HCG03:2 | 193 | 26.19 | 206 | −5.10 | 4.356 |
| [j] HCG03:3 | 191 | 12.42 | 206 | −8.39 | 5.437 |
| [k] HCG03:4 | 193 | 10.44 | 207 | −9.85 | 8.499 |
| [l] HCG03:5 | 190 | 12.36 | 208 | −7.32 | 3.857 |
| [m] HCG03:6 | 192 | 3.13 | 214 | −6.08 | 8.992 |
| [n] BA98:1 | 189 | 17.17 | 206 | −18.48 | 8.684 |
| [o] BA98:2 | 188 | 9.36 | 207 | −10.19 | 7.372 |
| [p] AB99:1 | 190 | 17.10 | 206 | −18.52 | 8.110 |
| [q] AB99:2 | 191 | 21.45 | 206 | −21.59 | 8.851 |
| [r] AB99:3 | 190 | 17.10 | 206 | −18.52 | 8.112 |
| [s] SW04:1 | 189 | 15.12 | 222 | 6.94 | 5.795 |
| [t] SW04:2 | 188 | 15.93 | 221 | 6.89 | 5.636 |
| [u] OH06:1 | 191 | 15.38 | 210 | −12.10 | 3.362 |
| [v] OH06:2 | 192 | 16.12 | 210 | −11.11 | 3.192 |

[a] SRCD from the Protein Circular Dichroism Data Bank for sperm whale myoglobin CD0000048000 [44,47]; [b] CDALC with PDB code 2 JHO [87] minimized using Insight®II/Discover/CVFF via110,000 steps of steepest descents followed by 21,000 steps of conjugate gradients. All hydrogens are included; [c] CDCALC with PDB code 2JHO minimized by NAMD/CHARMM22 5000 conjugate gradient steps. Hs attached to $-CH_3$s are ignored; [d] CAPPS with PDB code 2JHO including hydrogens. The following residues were ignored: 1−2, 19, 35−37, and 97−99; [e] Matrix method with *ab initio* parameters including only the protein backbone transitions on PDB code 1A6M [55]; [f] Matrix method, *ab initio* parameters including protein backbone & charge-transfer transitions (PDB code 1A6M) [55]; [g] Matrix method, *ab initio* parameters including protein backbone, charge-transfer & side chain transitions (PDB code 1A6M) [55]; [h] Matrix method, PDB code 4MBN [108] using *ab initio* parameters & a bandwidth of 15.5 nm [6]; [i] Matrix method, PDB code 4MBN [108] using *ab initio* parameters & a bandwidth of 9.5 nm [31]; [j] Matrix method, PDB code 4MBN [108] using *ab initio* parameters & a bandwidth of 10.5 nm & a dielectric constant of 1.5 [6]; [k] Matrix method, PDB code 4MBN [108] using *ab initio* parameters & bandwidth of 10.5 nm & a dielectric constant of 1.2 using four amide transitions [6]; [l] Matrix method, PDB code 4MBN [108] using *ab initio* parameters, a bandwidth of 10.5 nm, a dielectric constant of 1.2 using four amide transitions with rotations of −10° and −30° to the $\pi_{nb}$-π* and $\pi_b$-π * transitions respectively [6]; [m] Matrix method, PDB code 4MBN [108] using *ab initio* parameters, a bandwidth of 10.5 nm, a dielectric constant of 1.2 using four amide transition with no rotations of −30° to the $\pi_{nb}$-π* and $\pi_b$-π * transitions [6]; [n] Dipole interaction model on PDB code 1VXA [109] using Hy parameters & a bandwidth of 4000 $cm^{-1}$ [3]; [o] Dipole interaction model on PDB code 1VXA [109] using Hy parameters & a bandwidth of 6000 $cm^{-1}$ [3]; [p] Dipole interaction model on PDB code 1VXA [109] using Hy parameters & a bandwidth of 4000 $cm^{-1}$ & no solvent [102]; [q] Dipole interaction model on PDB code 1VXA [109] using Hy parameters & a bandwidth of 4000 $cm^{-1}$ & bound waters [102]; [r] Dipole interaction model on PDB code 1VXA [109] using Hy parameters & a bandwidth of 4000 $cm^{-1}$ & bound waters and lattice plus continuum [102]; [s] Matrix method on PDB code 1MBN [110] with INDO/S wavefunctions including the peptide groups only [13]; [t] Matrix method on PDB code 1MBN [110] with INDO/S wavefunction including the peptide groups and aromatic chromophores [13]; [u] Matrix method with *ab initio* parameters including local amide transitions and interpeptide charge-transfer transitions [57]; [v] Matrix method with *ab initio* parameters including only local amide transitions [57]. Grey highlight represents the smallest RMSD for a set of calculations.

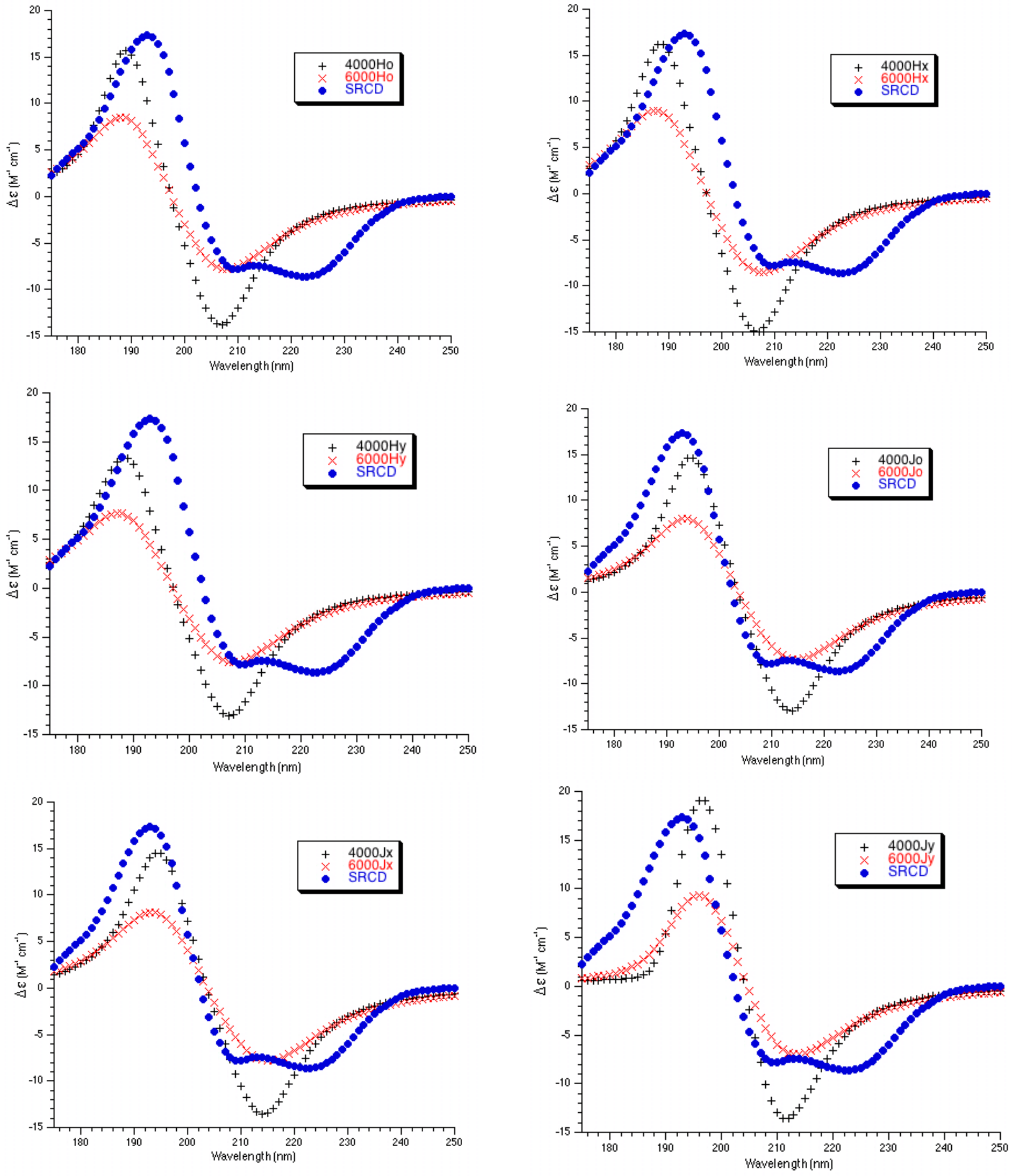


**Figure S26.** *Cont.*

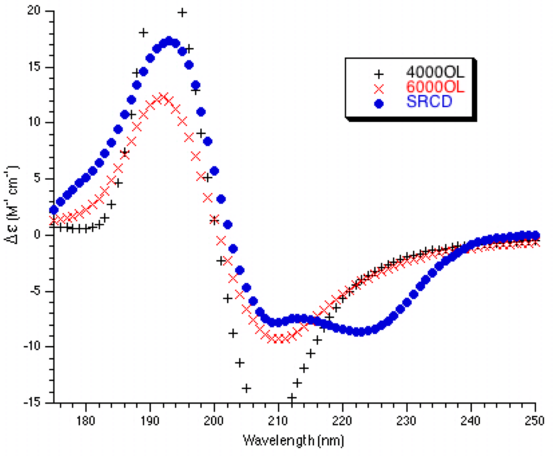


**Figure S26.** Sperm Whale Myoglobin Predicted CD Using CDCALC with Structure Minimized via NAMD/CHARMM22. The 2JHO [87] structure was minimized with 5000 conjugate gradient steps using NAMD/CHARMM22. Calculated spectra ignore all $-CH_3$ group hydrogens. Bandwidths are 4000 (+) and 6000 (×) $cm^{-1}$. The blue dots (•) are the experimental SRCD (CD000048000) [44,47]. The CATH fold classification [53] is mainly alpha/orthogonal bundle.

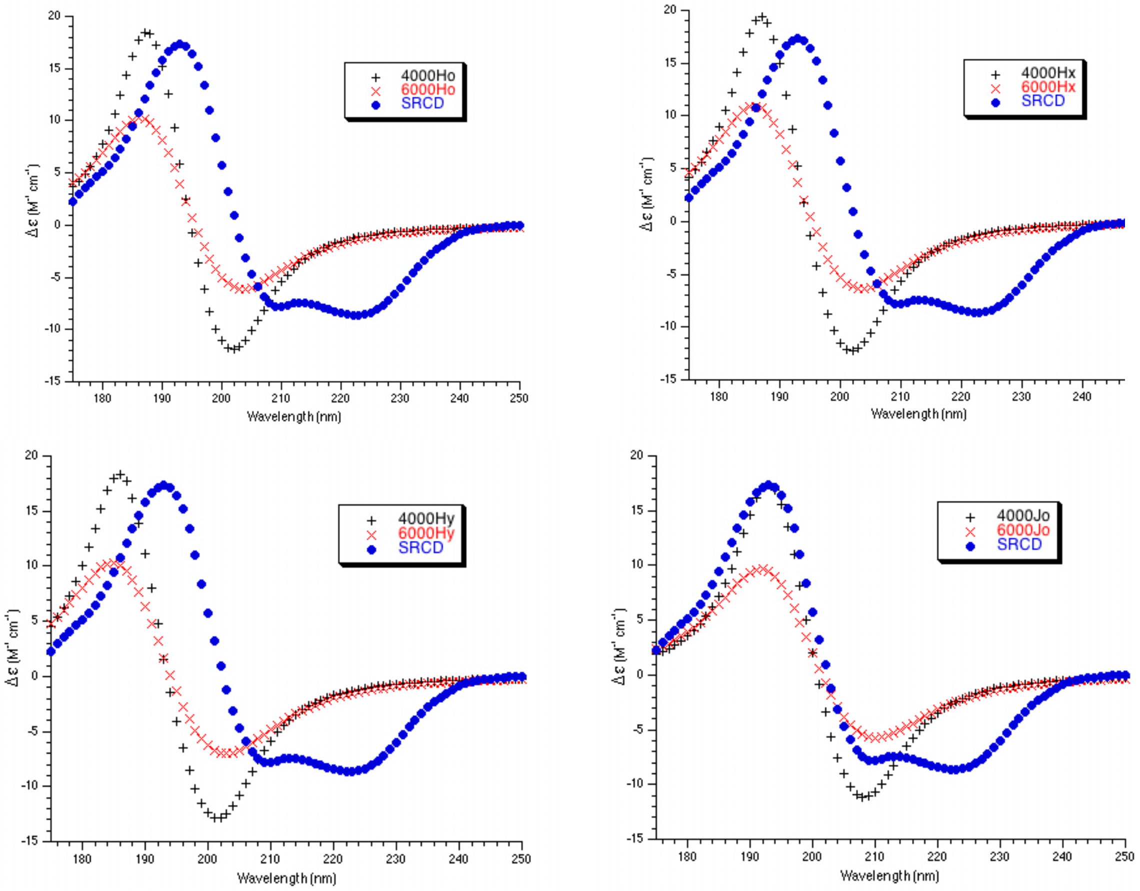


**Figure S27.** *Cont.*

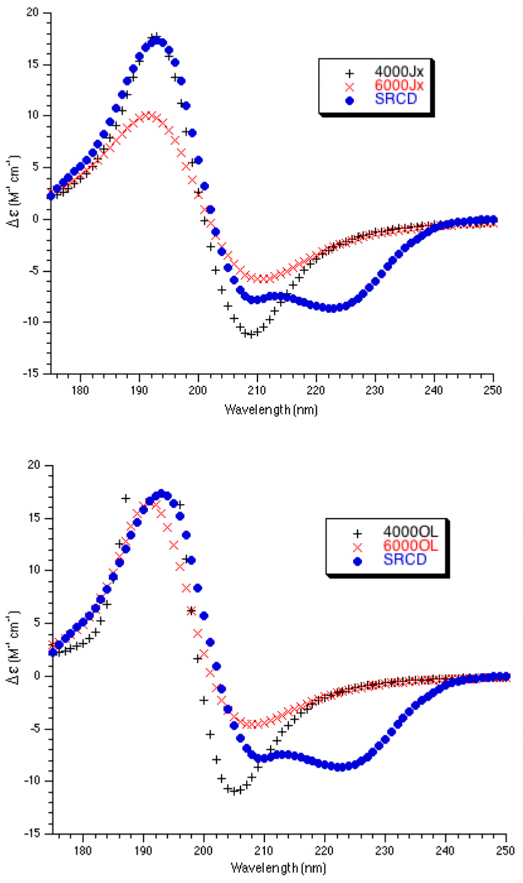


**Figure S27.** Sperm Whale Myoglobin Predicted CD Using CDCALC with Structure 2JHO [87] Minimized by Insight®II/Discover. Calculated spectra include all hydrogens. Bandwidths are 4000 (+) and 6000 (×) $cm^{-1}$. The blue dots (•) are the experimental SRCD (CD0000048000) [44,47]. The CATH fold classification [53] is mainly alpha/orthogonal bundle.

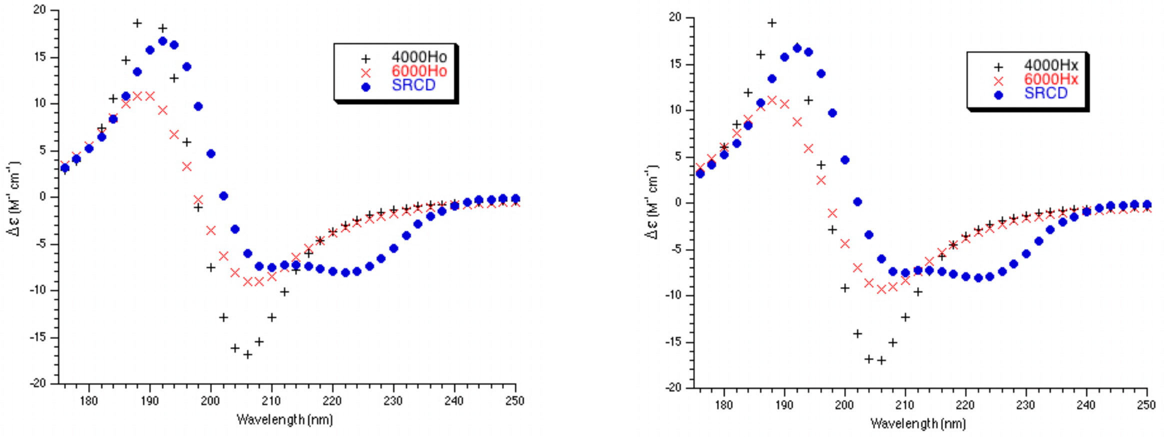


**Figure S28.** *Cont.*

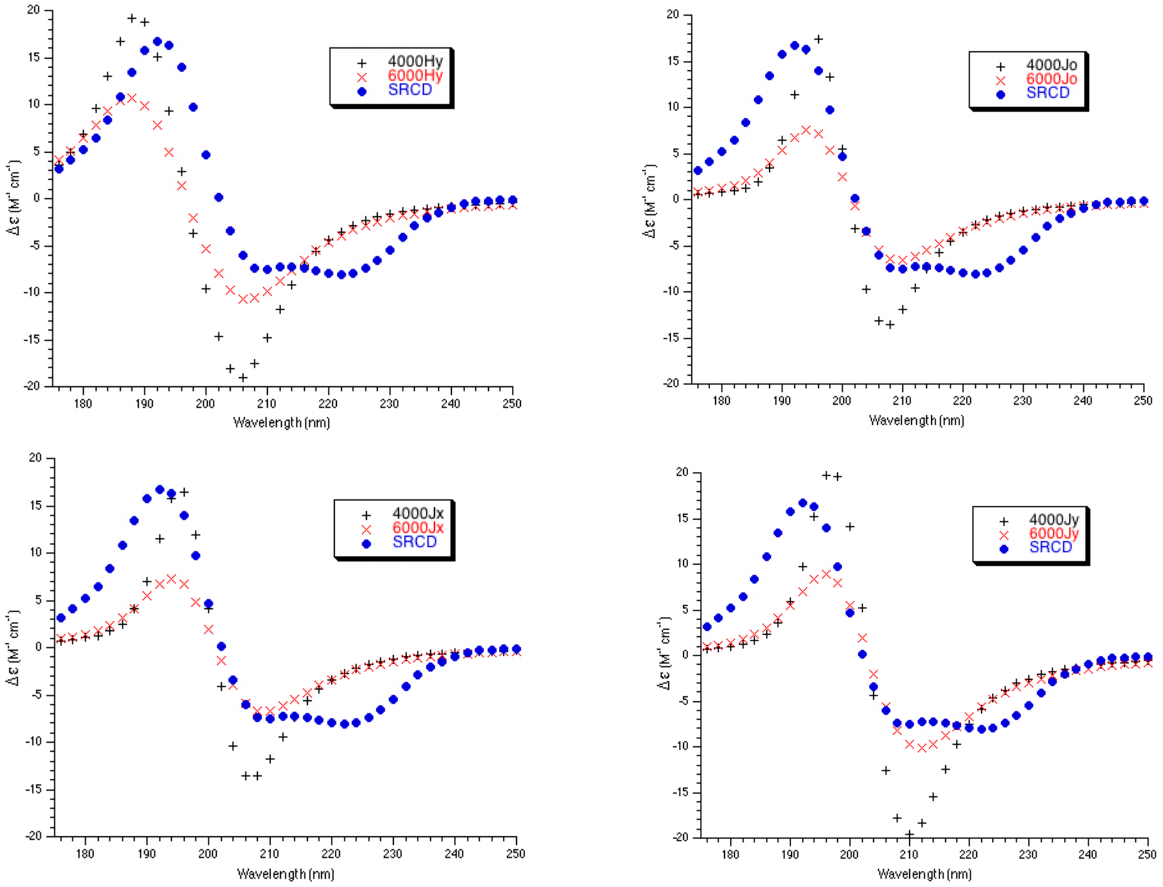


**Figure S28.** Sperm Whale Myoglobin Predicted CD Using CAPPS and 2JHO [87]. The rebuilt structure ignores residues 1–2, 19, 35–37, and 97–99. All other structures are included with hydrogens. Bandwidths are 4000 (+) and 6000 (×) $cm^{-1}$. The blue dots (•) are the experimental SRCD (CD0000048000) [44,47]. The CATH fold classification [53] is mainly alpha/orthogonal bundle.

Concanavalin A: PDB code 1NLS (Figure S29) is a saccharide-binding protein that has uncertain biological function, but structurally homologous animal lectins are believed to be important in cell-to-cell crosslinking and cell-attachment by viral proteins [78]. Concanavalin A is a homotetramer with each monomer containing 237 amino acids and 15 β-strands (Figure 10) [78]. The PCDDB [44] describes the secondary structure as 3.8% $3_{10}$-helix, 46.0% β-strand, 11.8% bonded turn, 13.5% bend, and 24.1% irregular.

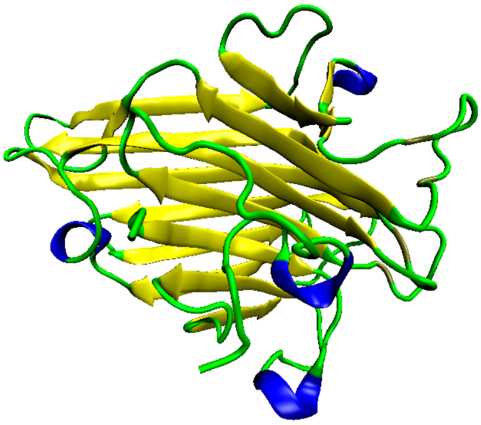

**Figure S29.** Concanavalin A Secondary Structure. Monomer of PDB code 1NLS [78] showing secondary structures. The yellow tapes are β-sheets (4–10, 24–29, 36–39, 47–55, 60–67, 73–78, 90–96, 105–116, 123−130, 140−144, 147−150, 170–175, 188–198, 209–215). The short blue cartoons/coil correspond to $3_{10}$-helices (14–18, 80–84, 150–152, 226–230). The green ropes are turns and other structures. CATH classifies the secondary structure as mainly beta/sandwich [53].

**Table S10.** CD Analysis of Concanavalin A. The DInaMo calculations are for the minimized or rebuilt structure using CDCALC or CAPPS. All RMSDs are calculated between 180 and 210 nm.

| CD Method | Peak Wavelength (nm) | Δε ($M^{-1}·cm^{-1}$) | Peak Wavelength (nm) | Δε ($M^{-1}·cm^{-1}$) | RMSD ($M^{-1}·cm^{-1}$) |
|---|---|---|---|---|---|
| [a] SRCD | 196 | 4.64 | 223 | −2.25 | 0.000 |
| [b] 4000 Ho | 200 | 3.22 | 211 | −1.15 | 1.815 |
| [b] 6000 Ho | 200 | 1.35 | 215 | −0.43 | 1.946 |
| [b] 4000 Hx | 200 | 3.63 | 212 | −1.01 | 1.965 |
| [b] 6000 Hx | 200 | 1.59 | 216 | −0.33 | 1.933 |
| [b] 4000 Hy | 199 | 3.09 | 211 | −1.19 | 1.574 |
| [b] 6000 Hy | 199 | 1.38 | 215 | −0.45 | 1.839 |
| [b] 4000 Jo | 206 | 3.03 | 218 | −1.08 | 3.170 |
| [b] 6000 Jo | 206 | 1.27 | 222 | −0.41 | 2.642 |
| [b] 4000 Jx | 207 | 3.46 | 220 | −0.96 | 3.253 |
| [b] 6000 Jx | 207 | 1.63 | 224 | −0.30 | 2.614 |
| [b] 4000 Jy | 207 | 1.46 | – | Not observed | 2.836 |
| [b] 6000 Jy | 209 | 0.69 | – | Not observed | 2.582 |
| [b] 4000 OL | 205 | 1.69 | – | Not observed | 2.945 |
| [b] 6000 OL | 207 | 0.81 | – | Not observed | 2.634 |
| [c] 4000 Ho | 198 | 8.51 | 208 | −2.01 | 2.295 |
| [c] 6000 Ho | 198 | 3.70 | 214 | −0.64 | 0.808 |
| [c] 4000 Hx | 198 | 8.75 | 210 | −1.88 | 2.669 |
| [c] 6000 Hx | 198 | 3.65 | 214 | −0.59 | 1.112 |
| [c] 4000 Hy | 198 | 9.70 | 210 | −1.21 | 2.600 |

**Table S10.** *Cont.*

| CD Method | Peak Wavelength (nm) | Δε ($M^{-1}·cm^{-1}$) | Peak Wavelength (nm) | Δε ($M^{-1}·cm^{-1}$) | RMSD ($M^{-1}·cm^{-1}$) |
|---|---|---|---|---|---|
| [c] 6000 Hy | 198 | 4.53 | 216 | −0.14 | 0.681 |
| [c] 4000 Jo | 198 | 2.18 | 210 | −0.65 | 2.133 |
| [c] 6000 Jo | 200 | 0.76 | 214 | −0.27 | 2.229 |
| [c] 4000 Jx | 200 | 2.05 | 210 | −0.54 | 2.343 |
| [c] 6000 Jx | 200 | 0.63 | 214 | −0.22 | 2.361 |
| [c] 4000 Jy | 200 | 3.22 | 214 | −0.23 | 2.209 |
| [c] 6000 Jy | 202 | 1.44 | – | Not observed | 2.076 |
| [d] BA98:1 | 192 | 4.30 | 210 | −0.78 | 3.303 |
| [e] BA98:2 | 193 | 7.84 | 206 | −2.89 | 2.794 |
| [f] MM1 | 194 | 4.98 | 214 | −1.44 | 1.518 |
| [g] MM2 | 194 | 5.13 | 213 | −1.47 | 1.874 |
| [h] MM3 | 194 | 7.54 | 211 | −1.76 | 3.375 |

[a] SRCD from the PCDDB code CD0000020000 [44,47]; [b] CDCALC using PDB structure 1NLS [78] minimized via NAMD/CHARMM22 and 5000 conjugate gradient steps. The hydrogens on all $-CH_3$ groups are ignored; [c] CAPPS results using PDB structure 1NLS [78] that contained rebuilt secondary structures including hydrogens; the following residues were ignored: 1–3, 11–13, 79, 150–155; [d] BA98:1 Dipole interaction model of PDB code 2CTV [111] including residues 4–6, 8–12, 14–18, 20–22, 24–28, 30–32, 34–37, 39–44, 46–51 with set Hy at 4000 $cm^{-1}$ [3]; [e] BA98:2 Dipole interaction model of PDB code 2CTV [111] including residues 4–6, 8–12, 14–18, 20–22, 24–28, 30–32, 34–37, 39–44, 46–51 with set Hy at 6000 $cm^{-1}$ [5]; [f] Matrix method using *ab initio* parameters on PDB code 1NLS including only the protein backbone transitions [55]; [g] Matrix method using *ab initio* parameters on PDB code 1NLS including protein backbone and charge-transfer transitions [55]; [h] Matrix method using *ab initio* parameters on PDB code 1NLS including protein backbone, charge-transfer and side chain transitions [55]. Grey highlight represents the smallest RMSD for a set of calculations.

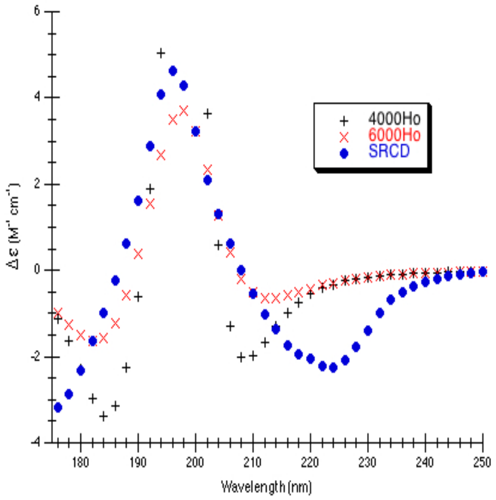


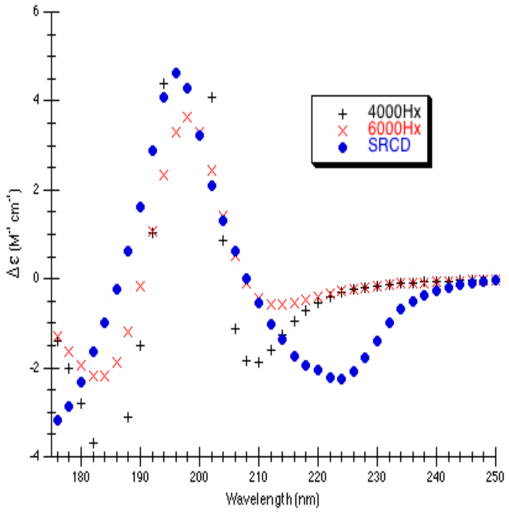


**Figure S30.** *Cont.*

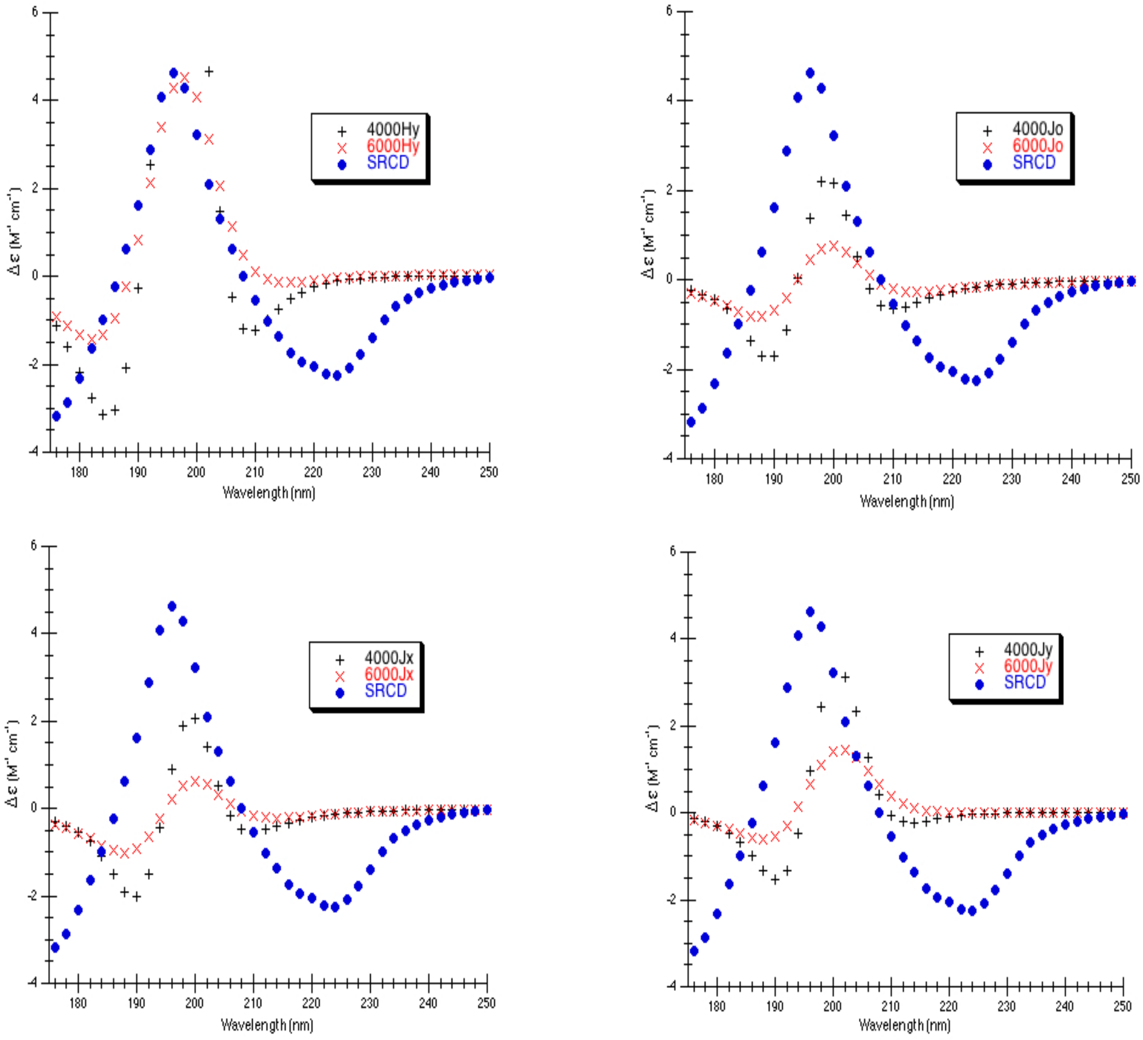


**Figure S30.** Concanavalin A Predicted CD Using CAPPS. The PDB 1NLS [78] structure is rebuilt ignoring residues 1–3, 11–13, 79, 150–152, 153–155. Bandwidths are 4000 (+) and 6000 (×) cm$^{-1}$. The blue dots (•) are the experimental SRCD (CD0000020000) [1,2]. CATH classifies the secondary structure as mainly beta/sandwich [53].

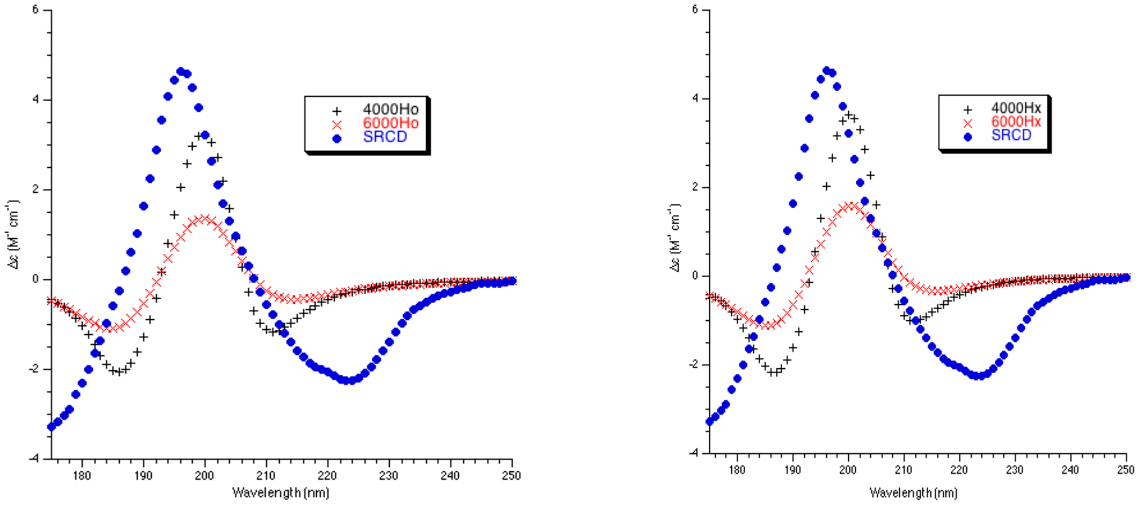


**Figure S31.** *Cont.*

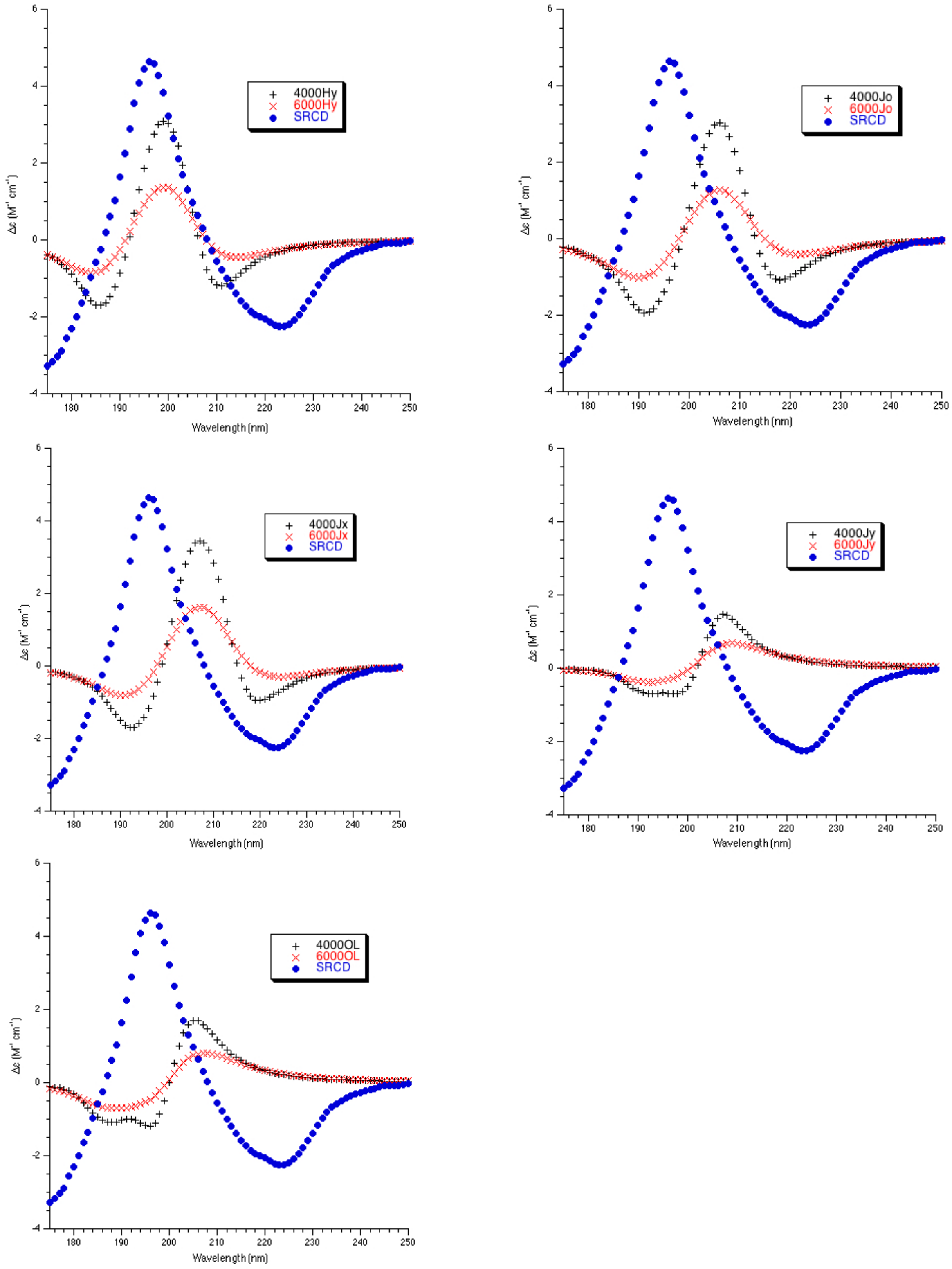


**Figure S31.** Concanavalin A Predicted CD Using CDCALC. The PDB 1NLS [78] structure was minimized with 5000 conjugate gradient steps using NAMD/CHARMM22. Calculated spectra ignore all $-CH_3$ group hydrogens. Bandwidths are 4000 (+) and 6000 (×) $cm^{-1}$. The blue dots (•) are the experimental SRCD (CD0000020000) [44,47]. CATH classifies the secondary structure as mainly beta/sandwich [53].

**Table S11.** CD Analysis of Outer Membrane Protein OPCA. The DInaMo calculations are for the minimized or rebuilt structure using CDCALC or CAPPS. All RMSDs are calculated between 180 and 210 nm.

| CD Method | Peak Wavelength (nm) | Δε ($M^{-1}\cdot cm^{-1}$) | Peak Wavelength (nm) | Δε ($M^{-1}\cdot cm^{-1}$) | RMSD ($M^{-1}\cdot cm^{-1}$) |
|---|---|---|---|---|---|
| [a] SRCD | 199 | 4.72 | 218 | −1.56 | 0.000 |
| [b] 4000 Ho | 197 | 3.06 | 213 | −0.559 | 1.526 |
| [b] 6000 Ho | 198 | 1.73 | 218 | −0.100 | 1.799 |
| [b] 4000 Hx | 198 | 3.12 | 213 | −0.641 | 1.574 |
| [b] 6000 Hx | 199 | 1.76 | 217 | −0.129 | 1.817 |
| [b] 4000 Hy | 198 | 3.00 | 214 | −0.322 | 1.625 |
| [b] 6000 Hy | 199 | 1.75 | not observed | – | 1.838 |
| [b] 4000 Jo | 203 | 2.88 | 220 | −0.525 | 2.664 |
| [b] 6000 Jo | 204 | 1.62 | 225 | −0.094 | 2.434 |
| [b] 4000 Jx | 205 | 2.91 | 221 | −0.687 | 2.962 |
| [b] 6000 Jx | 205 | 1.60 | 226 | −0.174 | 2.627 |
| [b] 4000 Jy | 209 | 1.24 | not observed | – | 2.959 |
| [b] 6000 Jy | 210 | 0.65 | not observed | – | 2.823 |
| [b] 4000 OL | 201 | 1.46 | not observed | – | 2.197 |
| [b] 6000 OL | 201 | 0.88 | not observed | – | 2.334 |

[a] SRCD from the PCDDB (CD0000119000) [44,59]; [b] CDCALC using PDB structure 2VDF [61] minimized via NAMD/CHARMM22 for 10,000 steps. Hydrogens attached to $-CH_3$ groups are ignored; Grey highlight represents the smallest RMSD for a set of calculations.

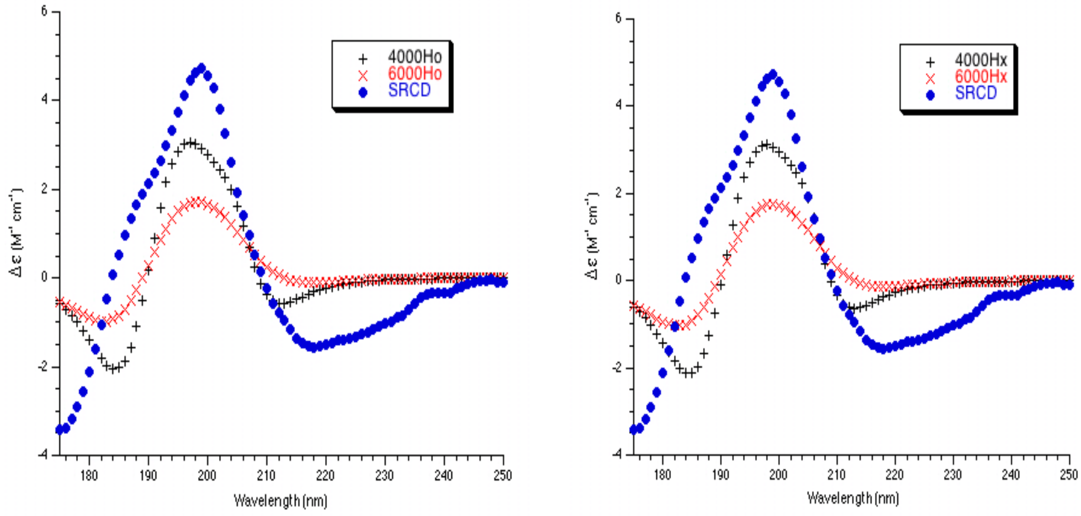


**Figure S32.** *Cont.*

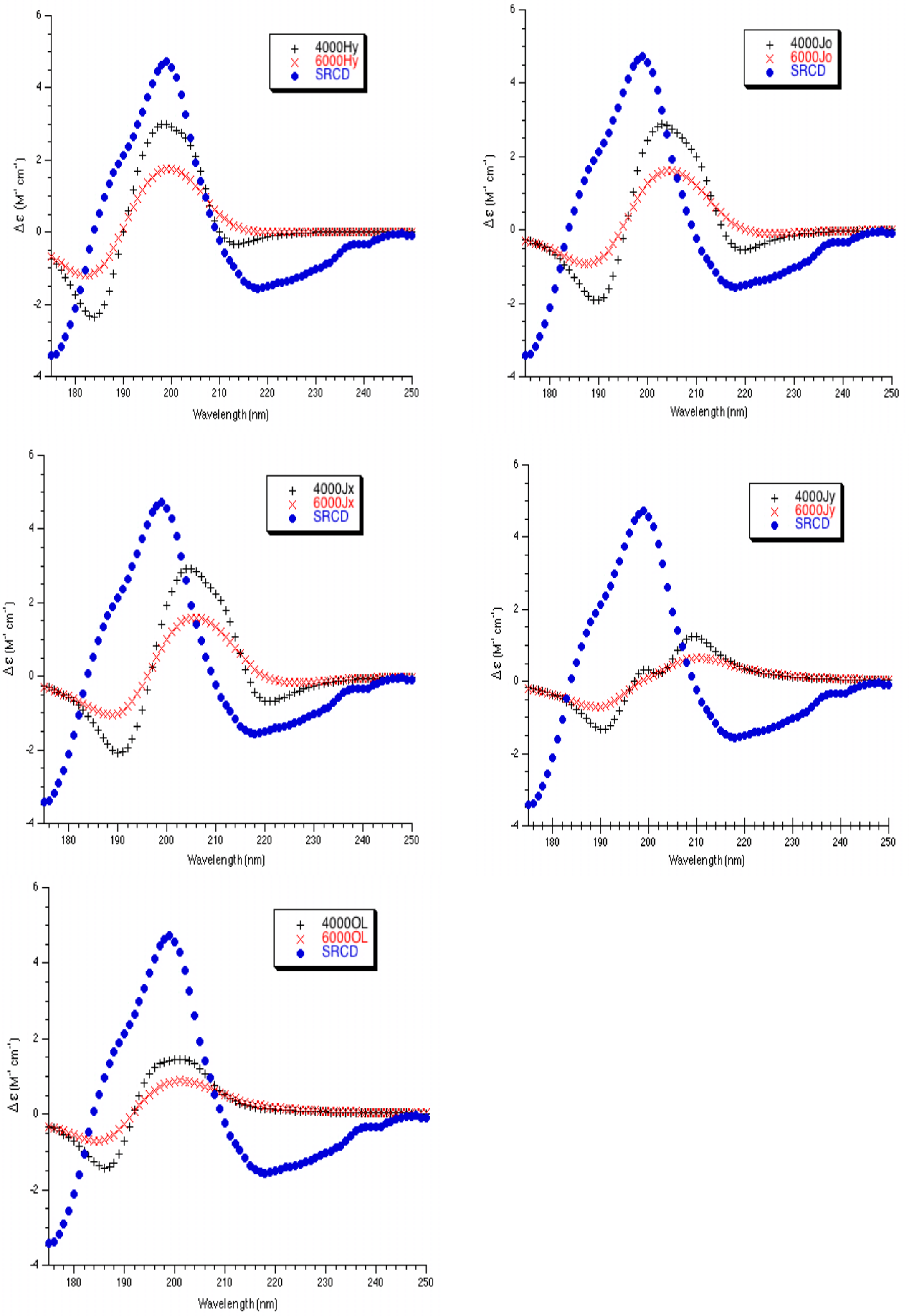


**Figure S32.** Outer Membrane Protein OPCA Predicted CD Using CDCALC. The 2VDF [61] structure was minimized via 10,000 conjugate gradient steps with NAMD/CHARMM22. Calculated spectra ignore all −$CH_3$ group hydrogens. Bandwidths are 4000 (+) and 6000 (×) $cm^{-1}$. The blue dots (•) are the experimental SRCD (CD0000119000) [44,59]. The CATH fold classification [53] is a single domain that is mainly beta/beta barrel.

Jacalin: PDB code 1KU8 [82] (Figure S33), a seed lectin from jack fruit that binds carbohydrates, is a tetramer of a heterodimer (*i.e.*, a heterooctomer), with the one chain of the dimer containing 133 amino acids and eight β-sheets each and the other chain of the dimer containing only 18 amino acids with one β-sheet [82] (Figure S33). CATH classifies each dimer as a single domain (four total) that is mainly beta/aligned-prism [53]. The PCDDB describes the secondary structure as 62.6% β-strand, 0.7% β-bridge, 5.6% bonded turn, 8.8% bend, and 22.4% irregular [44].

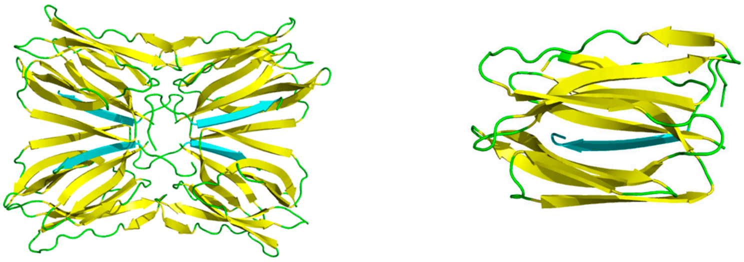

**Figure S33.** Jacalin Secondary Structure. 1KU8 [82] structure showing secondary structure elements; the yellow and cyan tapes are β-sheets (Chains A,C,E,G: 2–5, 11–19, 25–34, 37–40, 52–57, 65–75, 78–88, 92–97, 102–110, 112–120, 124–132; Chains B,D,F,H: 10–16), and the thin green ropes are turns and other structures. (**Left**) The full tetramer; (**Right**) The dimer used in CD calculations. The CATH fold classification [53] is mainly beta/aligned prism.

**Table S12.** CD Analysis of Jacalin. The DInaMo calculations are for the minimized or rebuilt structure using CDCALC or CAPPS. All RMSDs are calculated between 180 and 210 nm.

| CD Method | Trough (nm) | Δε ($M^{-1}\cdot cm^{-1}$) | Peak (nm) | Δε ($M^{-1}\cdot cm^{-1}$) | Trough (nm) | Δε ($M^{-1}\cdot cm^{-1}$) | RMSD ($M^{-1}\cdot cm^{-1}$) |
|---|---|---|---|---|---|---|---|
| [a] SRCD | 192 | −3.87 | 202 | 3.33 | 219 | −2.31 | 0.000 |
| [b] 4000Ho | 186 | −1.68 | 199 | 1.65 | 211 | −0.54 | 2.022 |
| [b] 6000Ho | 185 | −0.88 | 199 | 0.61 | 214 | −0.24 | 2.062 |
| [b] 4000Hx | 186 | −1.48 | 199 | 2.29 | 213 | −0.32 | 1.956 |
| [b] 6000Hx | 185 | −0.70 | 199 | 1.03 | 217 | −0.08 | 2.043 |
| [b] 4000Hy | 185 | −1.56 | 199 | 1.73 | 212 | −0.31 | 2.001 |
| [b] 6000Hy | 184 | −0.79 | 200 | 0.76 | 217 | −0.09 | 2.064 |
| [b] 4000Jo | 191 | −1.59 | 205 | 1.55 | 218 | −0.50 | 1.408 |
| [b] 6000Jo | 190 | −0.82 | 205 | 0.57 | 221 | −0.22 | 1.843 |
| [b] 4000Jx | 191 | −1.18 | 206 | 2.27 | 221 | −0.31 | 1.541 |
| [b] 6000Jx | 190 | −0.50 | 206 | 1.09 | 227 | −0.07 | 1.866 |
| [b] 4000Jy | 193 | −1.08 | 212 | 0.56 | not observed | – | 1.967 |
| [b] 6000Jy | 193 | −0.49 | 213 | 0.29 | not observed | – | 2.116 |
| [b] 4000OL | 195 | 0.51 | 201 | −1.55 | 211 | 0.73 | 2.558 |
| [b] 6000OL | 195 | −0.20 | 201 | −0.46 | 214 | 0.33 | 2.275 |

**Table S12.** *Cont.*

| CD Method | Trough (nm) | Δε ($M^{-1}\cdot cm^{-1}$) | Peak (nm) | Δε ($M^{-1}\cdot cm^{-1}$) | Trough (nm) | Δε ($M^{-1}\cdot cm^{-1}$) | RMSD ($M^{-1}\cdot cm^{-1}$) |
|---|---|---|---|---|---|---|---|
| [c] B09:1 | 178 | −2.62 | 197 | 4.69 | 220 | −1.30 | 3.672 |
| [d] B09:2 | 175 | −5.50 | 196 | 4.58 | 222 | −1.35 | 3.607 |
| [e] B09:3 | 183 | −4.24 | 203 | 3.81 | 224 | −1.16 | 2.284 |

[a] SRCD spectrum (CD0000041000) [44,47]; [b] CDALC using PDB structure 1KU8 [82] minimized via NAMD/CHARMM22 for 10,000 steps. Hydrogens attached to $-CH_3$ groups are ignored.; [c] Matrix method with *ab initio* parameters including only the protein backbone transitions on PDB code 1KU8 [55]; [d] Matrix method with *ab initio* parameters including protein backbone and charge-transfer transitions on PDB code 1KU8 [55]; [e] Matrix method with *ab initio* parameters including protein backbone, charge-transfer and side chain transitions on PDB code 1KU8 [55]; Grey highlight represents the lowest RMSD for each category.

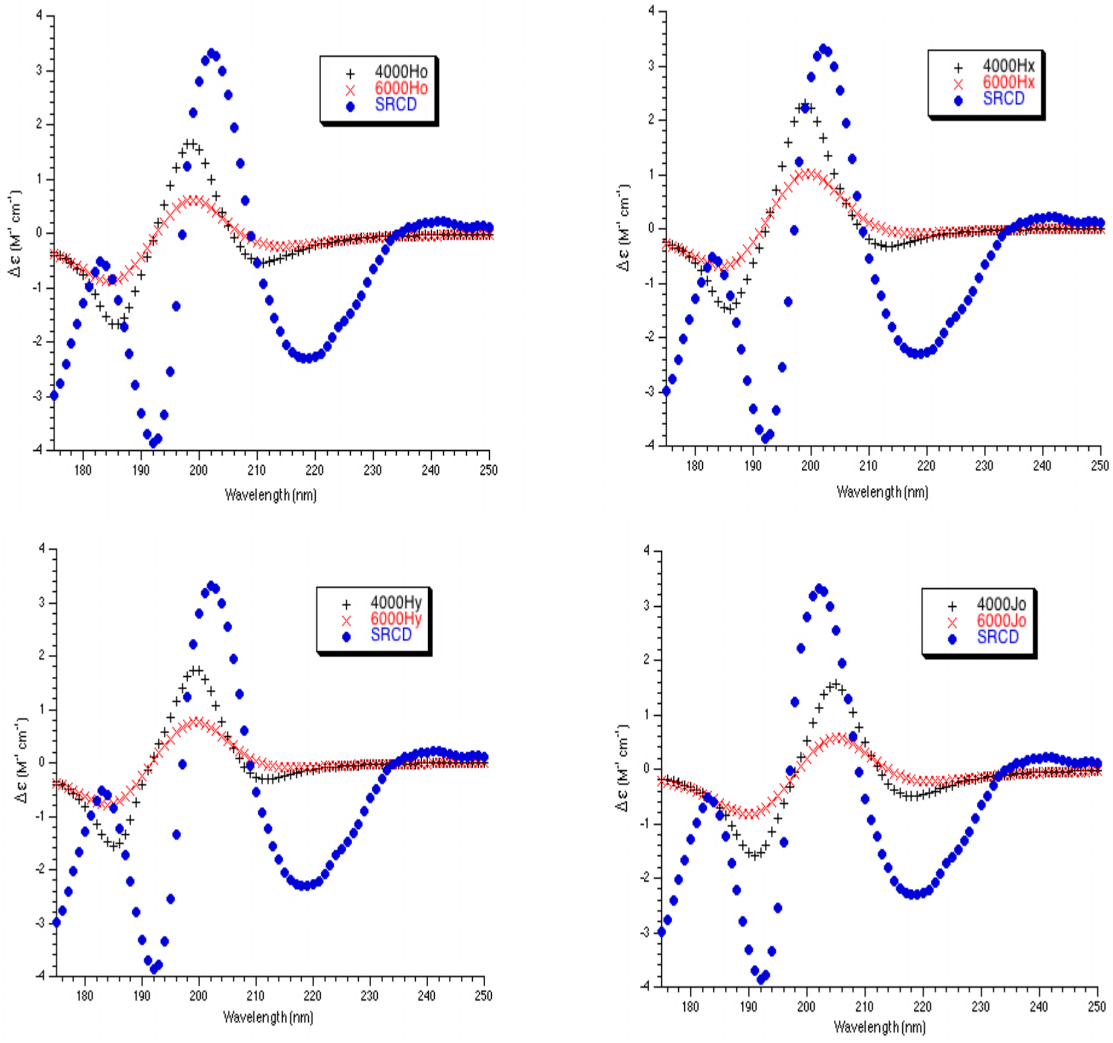


**Figure S34.** *Cont.*

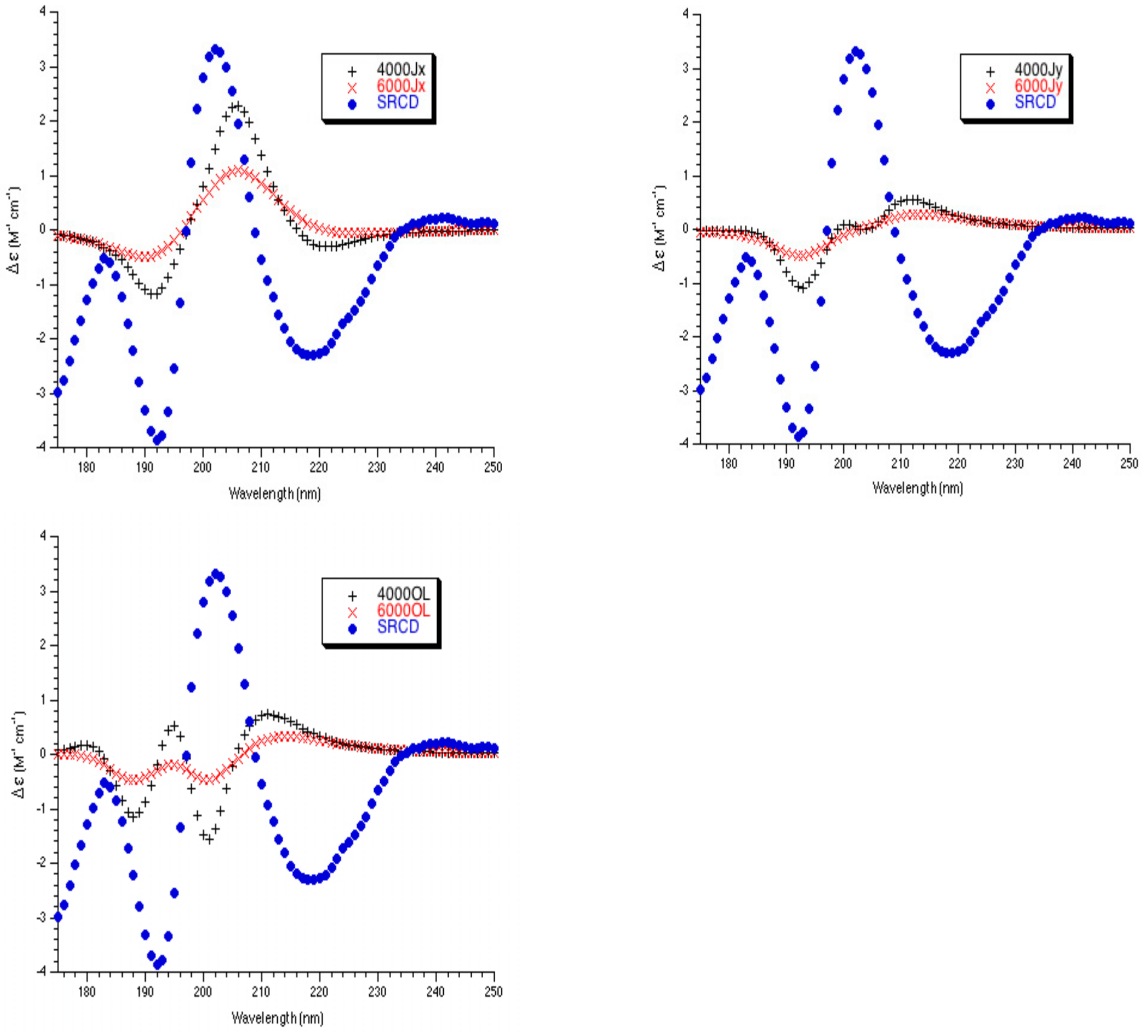


**Figure S34.** Jacalin Predicted CD Using CDCALC. The PDB 1KU8 [82] structure was minimized via 10,000 conjugate gradient steps with NAMD/CHARMM22. Calculated spectra ignore all $-CH_3$ group hydrogens. Bandwidths are 4000 (+) and 6000 (×) cm$^{-1}$. The blue dots (•) are the experimental SRCD (CD0000041000) [44,47]. The CATH fold classification [53] is mainly beta/aligned prism.

Rubredoxin: The PDB 1R0I [90] structure of rubredoxin (*Claustridum pasternarium*) is a monomer of 54 amino acids (Figure S35). The crystal structure replaces the native iron with cadmium to better understand the chelation geometry of this iron-containing protein; the Cd structures is very similar to the Fe, but the coordinating geometry is expanded. Natural rubredoxin has iron chelated to four Cys residues and acts as an electron acceptor for electron transport [112]. The CD spectra of other species of rubredoxin are similar in morphology (*Pseudomonas oleovorans* [113], *Desulfovibrio gigas* [114], *Pyrococcus furiosus* [115], and *Chlorobium tepidum* [116]), and replacing the Fe with Cd does not change the overall CD spectrum, but there is a small metal-dependent change in intensity [113]. In the minimization Zn is used since parameters for Cd are not available. CATH classifies 1R0I as a monomer with a single domain that is mainly beta/single sheet [53] (Figure 13). The PCDDB [44] classifies the secondary structure as 16.7% $3_{10}$-helix, 14.8% β-strand, 7.4% β-bridge, 25.9% bonded turn, 1.9% bend, and 33.3% irregular.

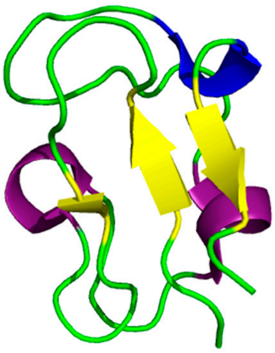

**Figure S35.** Rubredoxin Secondary Structure. PDB 1R0I [90] secondary structure elements: thick purple cartoons/coils correspond to α-helices (19–23, 29–33), the short blue cartoons/coil correspond to $3_{10}$-helices (45–47), the yellow tapes are β-sheets, (4–6, 12–13, 49–51), and the thin green ropes are turns and other structures. The CATH fold classification [53] is mainly beta/single sheet.

**Table S13.** CD Analysis of Rubredoxin. The DInaMo calculations are for the minimized or rebuilt structure using CDCALC or CAPPS. All RMSDs are calculated between 180 and 210 nm.

| CD Method | Peak Wavelength (nm) | Δε ($M^{-1}·cm^{-1}$) | Peak Wavelength (nm) | Δε ($M^{-1}·cm^{-1}$) | RMSD ($M^{-1}·cm^{-1}$) |
|---|---|---|---|---|---|
| [a] SRCD | 191 | 1.47 | 202 | −6.23 | 0.000 |
| [b] 4000Ho | 189 | 4.18 | 205 | −3.29 | 2.411 |
| [b] 6000Ho | 189 | 2.13 | 207 | −1.72 | 2.447 |
| [b] 4000Hx | 189 | 3.12 | 205 | −3.30 | 1.900 |
| [b] 6000Hx | 189 | 1.42 | 207 | −1.89 | 2.149 |
| [b] 4000Hy | 189 | 3.21 | 206 | −3.52 | 2.144 |
| [b] 6000Hy | 189 | 1.56 | 207 | −1.93 | 2.266 |
| [b] 4000Jo | 195 | 3.91 | 212 | −3.09 | 3.924 |
| [b] 6000Jo | 195 | 2.00 | 214 | −1.61 | 3.344 |
| [b] 4000Jx | 195 | 2.80 | 213 | −3.03 | 3.449 |
| [b] 6000Jx | 195 | 1.25 | 214 | −1.74 | 3.033 |
| [b] 4000Jy | 195 | 3.11 | 207 | −3.58 | 3.361 |
| [b] 6000Jy | 194 | 1.85 | 210 | −1.78 | 2.915 |
| [b] 4000OL | 191 | 5.45 | 202 | −4.19 | 2.333 |
| [b] 6000OL | 190 | 2.58 | 204 | −1.90 | 2.302 |
| [c] 4000Ho | 190 | 5.11 | 202 | −5.91 | 1.852 |
| [c] 6000Ho | 188 | 2.25 | 204 | −2.93 | 1.684 |
| [c] 4000Hx | 190 | 4.52 | 202 | −5.71 | 1.472 |
| [c] 6000Hx | 188 | 1.81 | 204 | −2.85 | 1.561 |
| [c] 4000Hy | 188 | 5.82 | 202 | −5.40 | 2.254 |

**Table S13.** *Cont.*

| CD Method | Peak Wavelength (nm) | Δε ($M^{-1}\cdot cm^{-1}$) | Peak Wavelength (nm) | Δε ($M^{-1}\cdot cm^{-1}$) | RMSD ($M^{-1}\cdot cm^{-1}$) |
|---|---|---|---|---|---|
| [c] 6000Hy | 188 | 2.76 | 202 | −2.70 | 1.886 |
| [c] 4000Jo | 192 | 2.44 | 206 | −5.60 | 2.488 |
| [c] 6000Jo | 190 | 1.29 | 208 | −3.11 | 2.086 |
| [c] 4000Jx | 192 | 2.55 | 206 | −5.62 | 2.289 |
| [c] 6000Jx | 190 | 1.26 | 208 | −3.10 | 1.979 |
| [c] 4000Jy | 190 | 3.45 | 208 | −7.48 | 3.506 |
| [c] 6000Jy | 188 | 1.98 | 208 | 4.12 | 2.363 |
| [d] BA98:1 | 192 | 4.30 | 210 | −0.78 | 3.916 |
| [e] BA98:2 | 193 | 7.84 | 206 | −2.89 | 4.657 |
| [f] B09:1 | 194 | 4.98 | 214 | −1.44 | 4.432 |
| [g] B09:2 | 194 | 5.13 | 213 | −1.47 | 4.568 |
| [h] B09:3 | 194 | 7.54 | 211 | −1.76 | 5.622 |

[a] SRCD from the PCDDB code CD0000064000 [44,47]; [b] CDCALC using PDB structure 1R0I [90] minimized via NAMD/CHARMM22 and 5,000 conjugate gradient steps. The hydrogens on all $-CH_3$ groups are ignored; [c] CAPPS results using PDB structure 1R0I [90] that contained rebuilt secondary structures including hydrogens; the following residues were ignored: 1–3, 4–6, 48, 45–47; [d] BA98:1 Dipole interaction model of PDB code 8RXN [60] including residues 4–6, 8–12, 14–18, 20–22, 24–28, 30–32, 34–37, 39–44, 46–51 with set Hy at 4000 $cm^{-1}$ [3]; [e] BA98:2 Dipole interaction model of PDB code 8RXN [60] including residues 4–6, 8–12, 14–18, 20–22, 24–28, 30–32, 34–37, 39–44, 46–51 with set Hy at 6000 $cm^{-1}$ [3]; [f] Matrix method on PDB code 1R0I [55] using *ab initio* parameters including only the protein backbone transitions; [g] Matrix method on PDB code 1R0I [55] using *ab initio* parameters including protein backbone and charge-transfer transitions; [h] Matrix method on PDB code 1R0I [55] using *ab initio* parameters including protein backbone, charge-transfer and side chain transitions. Grey highlight represents the lowest RMSD for each category.

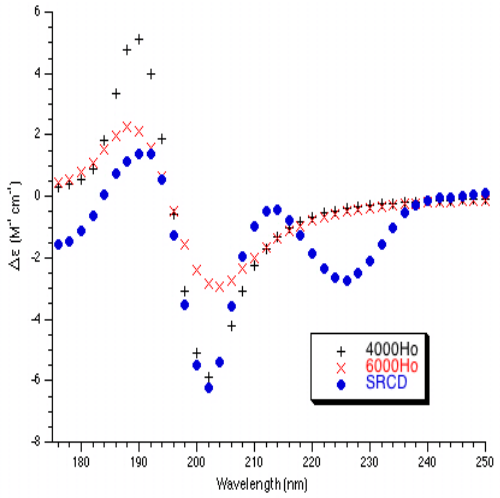


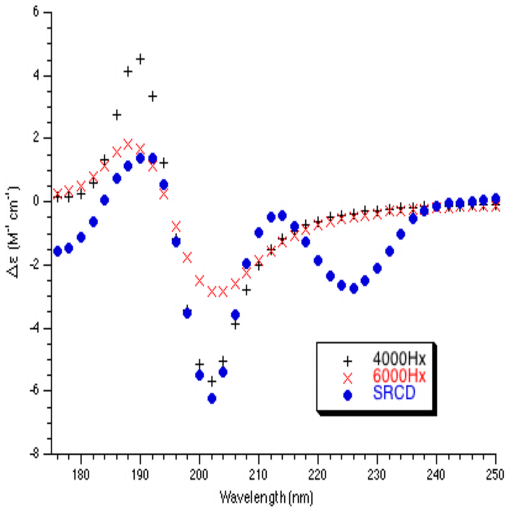


**Figure S36.** *Cont.*

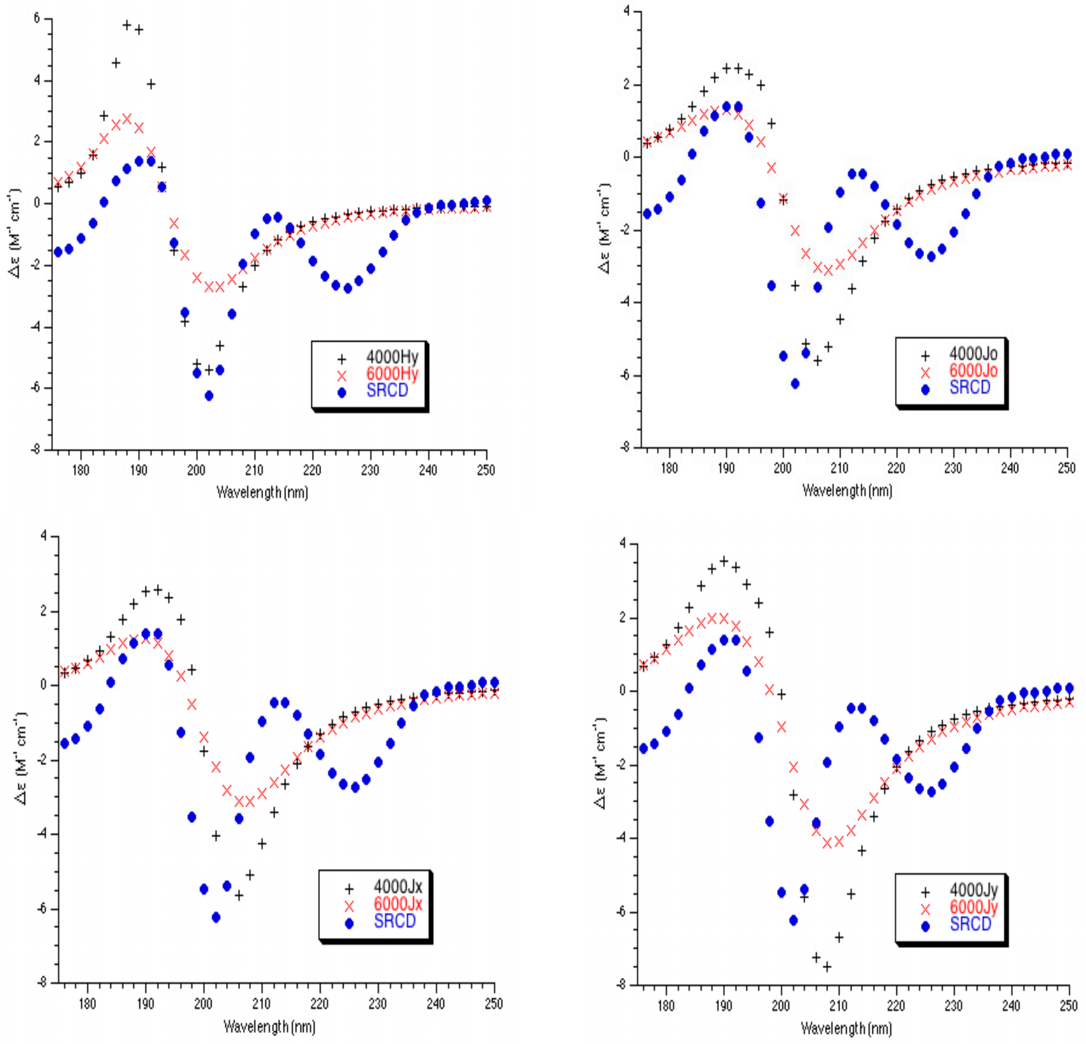


**Figure S36.** Rubredoxin Predicted CD Using CAPPS. The PDB 1R0I [90] structure was rebuilt ignoring residues 1–3, 4–6, 48, and 45–47. Bandwidths are 4000 (+) and 6000 (×) $cm^{-1}$. The blue dots (•) are the experimental SRCD (CD0000064000) [44,47]. The CATH fold classification [53] is mainly beta/single sheet.

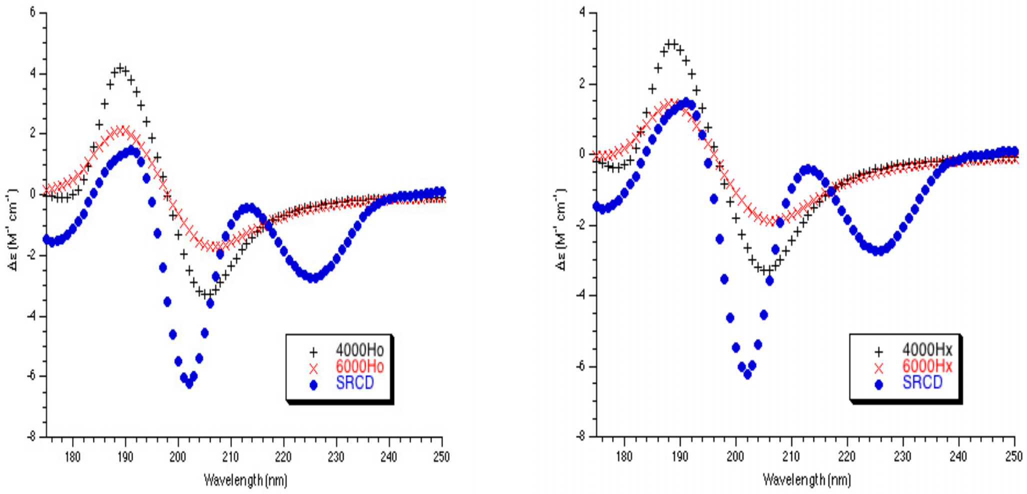


**Figure S37.** *Cont.*

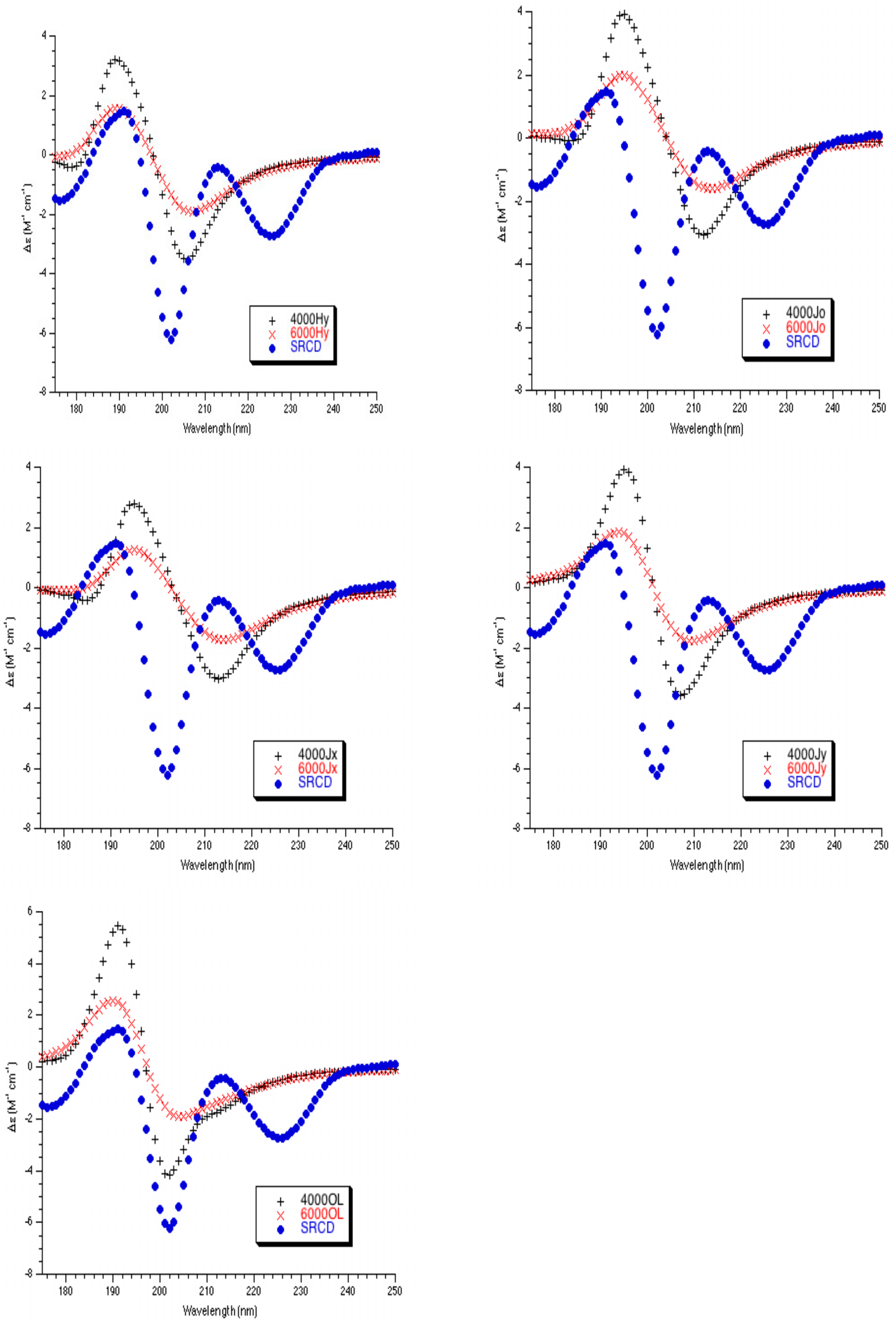


**Figure S37.** Rubredoxin Predicted CD Using CDCALC. The PDB 1R0I [90] structure was minimized with 5000 conjugate gradient steps using NAMD/CHARMM22. Calculated spectra ignore all –$CH_3$ group hydrogens. Bandwidths are 4000 (+) and 6000 (×) $cm^{-1}$. The blue dots (•) are the experimental SRCD (CD0000064000) [44,47]. The CATH fold classification [53] is mainly beta/single sheet.

Lentil Lectin: The PDB 1LES structure of lentil lectin (Figure S38) is a heterotetramer (a dimer of a heterodimer) that has 181 residues in one chain and 52 residues in the other chain [83]. CATH classifies each chain as a single domain, both of which are mainly beta/sandwich [53]. The PCDDB classifies the secondary structure as 1.7% α-helix, 2.6% $3_{10}$-helix, 47.0% β-strand, 1.3% β-bridge, 10.7% bonded turn, 11.6% bend, and 25.1% irregular [44].

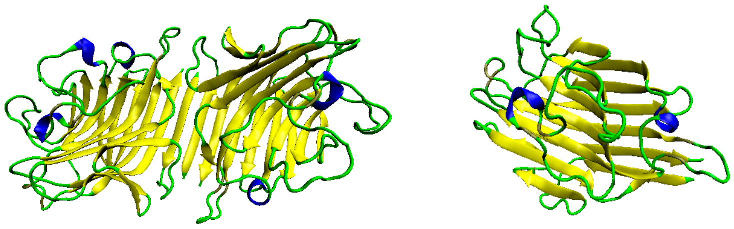

**Figure S38.** Lentil Lectin Secondary Structure. PDB code 1LES [83] secondary structure elements: thick blue cartoons/coils correspond to α-helices in chains A & C: 98–100, 111–113 and to $3_{10}$-helices (45−47), the yellow tapes are β-sheets (chains A & C: 2–8, 18–23, 41–46, 62–69, 82–87, 116–121, 136–141, 146–151, 158–166, A173–A179; chains B & D: 2–8, 22–27, 39–46), and the thin green ropes are turns and other structures. (**Left**) Full heterotetramer; (**Right**) Dimer used in CD calculations. The CATH fold classification [53] is mainly beta/sandwich.

**Table S14.** CD Analysis of Lentil Lectin. The DInaMo calculations are for the minimized or rebuilt structure using CDCALC. All RMSDs are calculated between 180 and 210 nm.

| CD Method | Peak Wavelength (nm) | Δε ($M^{-1}·cm^{-1}$) | Peak Wavelength (nm) | Δε ($M^{-1}·cm^{-1}$) | Peak Wavelength (nm) | Δε ($M^{-1}·cm^{-1}$) | RMSD ($M^{-1}·cm^{-1}$) |
|---|---|---|---|---|---|---|---|
| [a] SRCD | 177 | −4.28 | 195 | 5.43 | 226 | −1.33 | 0.000 |
| [b] 4000 Ho | 184 | −0.94 | 198 | 3.85 | 210 | −1.27 | 1.973 |
| [b] 6000 Ho | 182 | −0.40 | 198 | 1.82 | 213 | −0.43 | 2.488 |
| [b] 4000 Hx | 183 | −0.80 | 198 | 3.76 | 210 | −1.43 | 2.037 |
| [b] 6000 Hx | 181 | −0.32 | 198 | 1.78 | 214 | −0.52 | 2.485 |
| [b] 4000 Hy | 184 | −1.05 | 197 | 3.81 | 210 | −1.29 | 1.887 |
| [b] 6000 Hy | 182 | −0.43 | 197 | 1.83 | 213 | −0.46 | 2.422 |
| [b] 4000 Jo | 189 | −0.89 | 204 | 3.62 | 217 | −1.19 | 3.044 |
| [b] 6000 Jo | 187 | −0.37 | 204 | 1.71 | 221 | −0.41 | 2.894 |
| [b] 4000 Jx | 189 | −0.73 | 205 | 3.51 | 218 | −1.39 | 3.131 |
| [b] 6000 Jx | 187 | −0.28 | 205 | 1.68 | 222 | −0.52 | 2.939 |
| [b] 4000 Jy | 189 | −0.68 | 207 | 0.98 | not observed | – | 3.524 |
| [b] 6000 Jy | 189 | −0.43 | 209 | 0.39 | not observed | – | 3.419 |
| [b] 4000 OL | 185 | −0.93 | 204 | 0.82 | not observed | – | 3.571 |
| [b] 6000 OL | 185 | −0.64 | 207 | 0.25 | not observed | – | 3.472 |
| [c] 4000 Ho | 182 | −3.06 | 198 | 7.23 | 208 | −1.36 | 1.701 |
| [c] 6000 Ho | 182 | −1.60 | 198 | 3.21 | 214 | −0.40 | 1.653 |
| [c] 4000 Hx | 184 | −3.79 | 198 | 7.35 | 208 | −1.43 | 1.873 |
| [c] 6000 Hx | 182 | −2.05 | 198 | 3.18 | 212 | −0.40 | 1.654 |
| [c] 4000 Hy | 182 | −2.94 | 196 | 8.72 | 210 | −0.42 | 1.695 |

**Table S14.** *Cont.*

| CD Method | Peak Wavelength (nm) | Δε ($M^{-1}\cdot cm^{-1}$) | Peak Wavelength (nm) | Δε ($M^{-1}\cdot cm^{-1}$) | Peak Wavelength (nm) | Δε ($M^{-1}\cdot cm^{-1}$) | RMSD ($M^{-1}\cdot cm^{-1}$) |
|---|---|---|---|---|---|---|---|
| [c] 6000 Hy | 182 | −1.39 | 196 | 4.12 | not observed | – | 1.232 |
| [c] 4000 Jo | 188 | −2.11 | 202 | 2.37 | 214 | −0.15 | 3.065 |
| [c] 6000 Jo | 188 | −1.12 | 202 | 0.97 | 222 | −0.014 | 3.081 |
| [c] 4000 Jx | 188 | −2.19 | 202 | 2.29 | 214 | −0.16 | 3.099 |
| [c] 6000 Jx | 188 | −1.17 | 202 | 0.91 | 222 | −0.02 | 3.109 |
| [c] 4000 Jy | 190 | −2.04 | 202 | 3.66 | not observed | – | 3.160 |
| [c] 6000 Jy | 188 | −0.98 | 204 | 1.78 | not observed | – | 2.938 |
| [d] B09:1 | 179 | −2.60 | 197 | 4.71 | 220 | −1.29 | 0.690 |
| [e] B09:2 | 175 | −5.40 | 197 | 4.97 | 220 | −1.32 | 0.415 |
| [f] B09:3 | 178 | −6.48 | 197 | 7.04 | 222 | −1.28 | 1.570 |
| [g] SI | 178 | −8.69 | 196 | 15.40 | 219 | −4.17 | 0.888 |
| [h] SII | 181 | −14.99 | 197 | 15.15 | 224 | −3.12 | 0.932 |
| [i] SIII | 181 | −1.65 | 197 | 5.66 | 221 | −0.90 | 2.997 |

[a] SRCD from the PCDDB code CD0000043000 [44,47]; [b] CDCALC using PDB structure 1LES [83] chains A and B minimized via NAMD/CHARMM22 and 5,000 conjugate gradient steps. The hydrogens on all $-CH_3$ groups are ignored; [c] CAPPS results using PDB structure 1LES [83] that contained rebuilt secondary structures including hydrogens; the following residues were ignored: 1, 98–100, 62–69, 180–182, 190–192; [d] Matrix method using *ab initio* parameters including only the protein backbone transitions on PDB code 1LES [55]; [e] Matrix method using *ab initio* parameters including protein backbone and charge-transfer transitions PDB code 1LES [55]; [f] Matrix method using *ab initio* parameters including protein backbone, charge-transfer and side chain transitions PDB code 1LES [55]; [g] Exciton Hamiltonian with Electrostatic Fluctuations 1LES simulated spectrum based on a single conformation convoluted with a Gaussian envelop [29]; [h] Exciton Hamiltonian with Electrostatic Fluctuations 1LES simulated spectrum based on 2000 MD snapshots that considers the electrostatic potential from all surroundings [29]; [i] Exciton Hamiltonian with Electrostatic Fluctuations 1LES simulated spectrum that takes account of only peptide groups [29]. Grey highlight represents the lowest RMSD for each category.

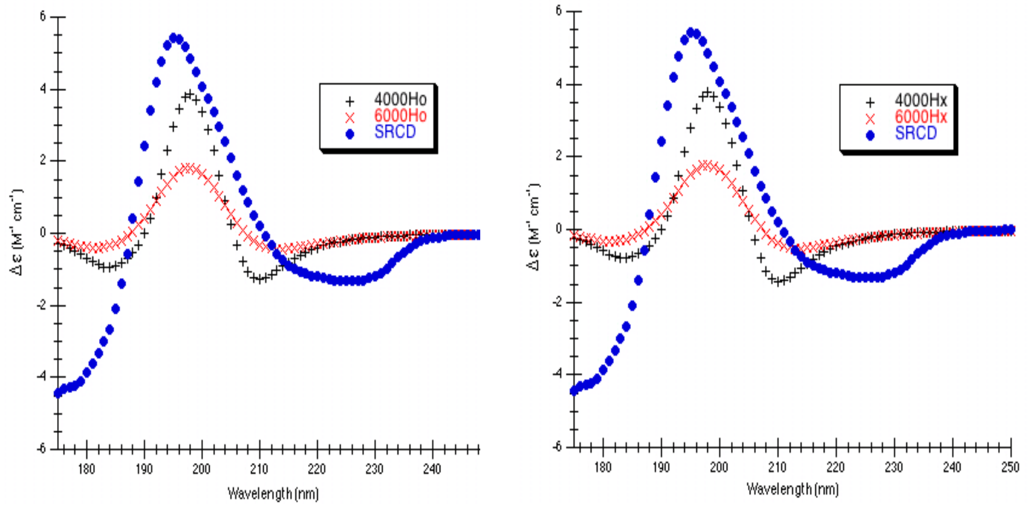


**Figure S39.** *Cont.*

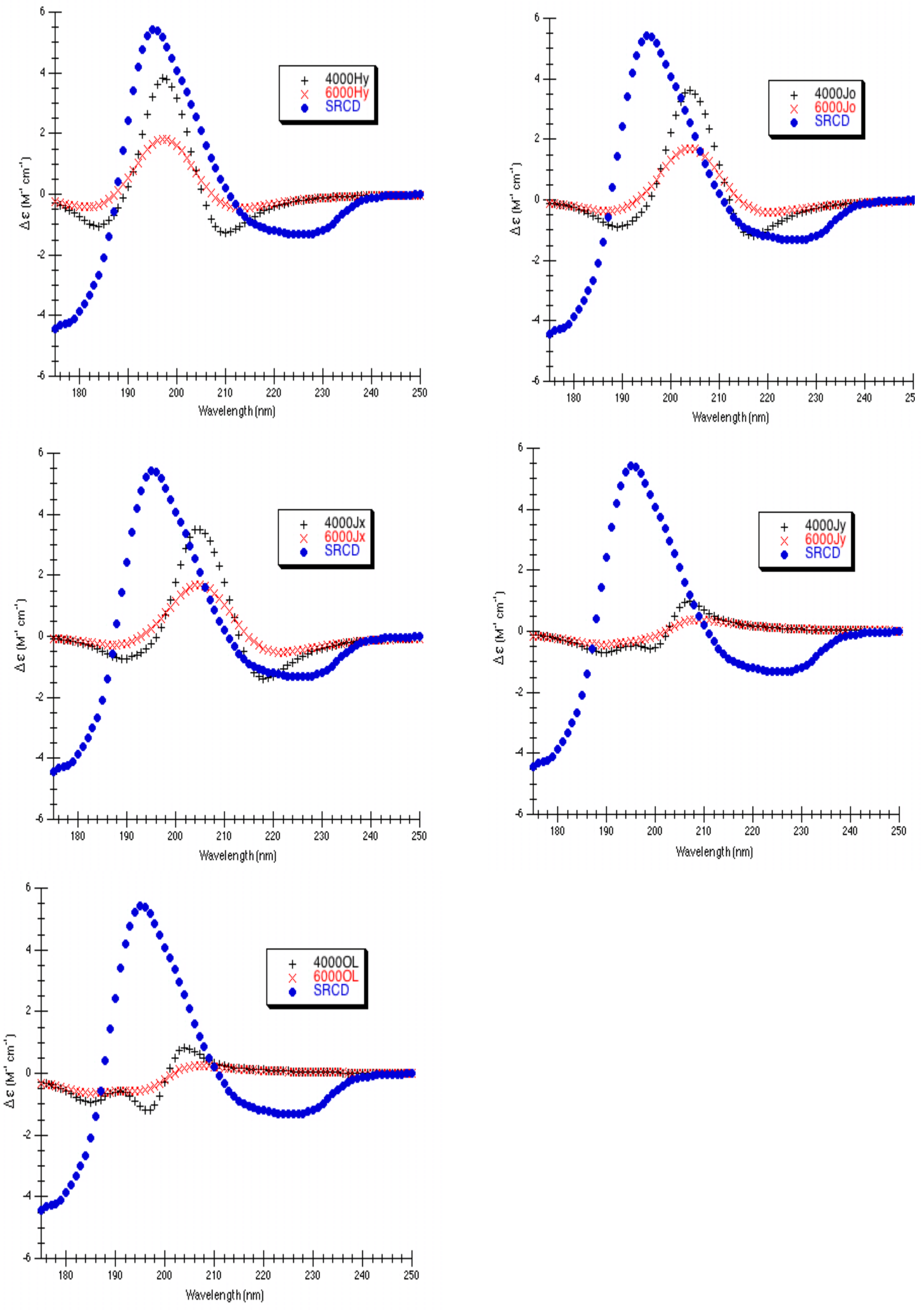


**Figure S39.** Lentil Lectin Predicted CD Using CDCALC. The PDB 1LES [83] A & B chains were minimized with 5000 conjugate gradient steps using NAMD/CHARMM22. Calculated spectra ignore all −$CH_3$ group hydrogens. Bandwidths are 4000 (+) and 6000 (×) $cm^{-1}$. The blue dots (•) are the experimental SRCD (CD0000043000) [44,47]. The CATH fold classification [53] is mainly beta/sandwich.

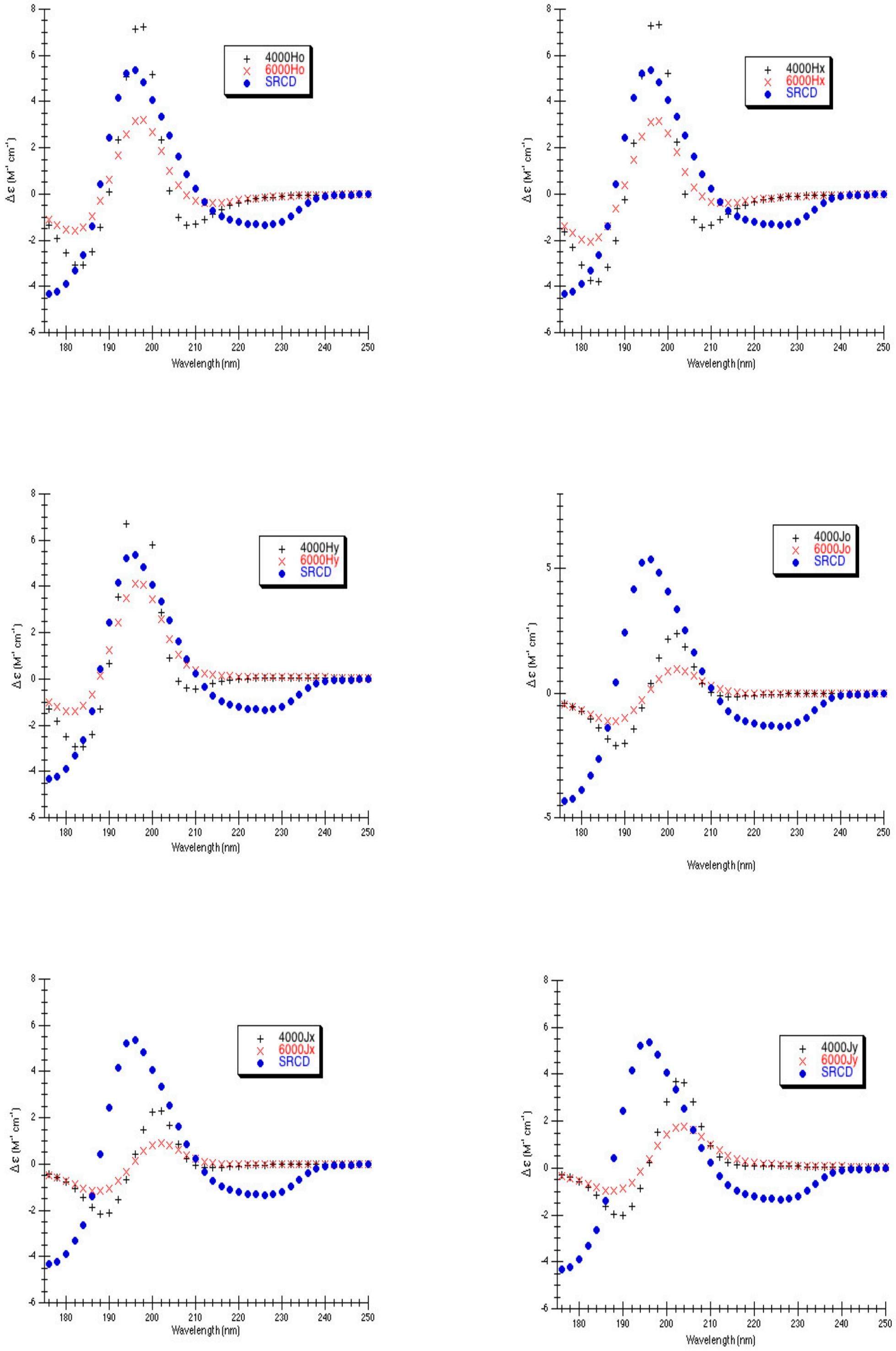


**Figure S40.** Lentil Lectin Predicted CD Using CAPPS. The rebuilt structure of 1LES [83] ignores residues 1, 98–100, 62–69, 180–182, 190–192. Calculated spectra ignore all $-CH_3$ group hydrogens. Bandwidths are 4000 (+) and 6000 (×) $cm^{-1}$. The blue dots (•) are the experimental SRCD (CD0000043000) [44,47]. The CATH fold classification [53] is mainly beta/sandwich.

Pea Lectin: Like the lentil lectin, PDB code 1OFS [73], is a sucrose-binding heterotetramer, a dimer of a heterodimer with an α-chain of 187 residues and a β-chain of 48 residues (Figure S41). CATH classifies each chain as mainly beta/sandwich [53]. The PCDDB [44] describes the secondary structure as 0.9% α-helix, 3.2% $3_{10}$-helix, 46.4% β-strand, 0.9% β-bridge, 11.7% bonded turn, 10.6% bend, and 26.4% irregular.

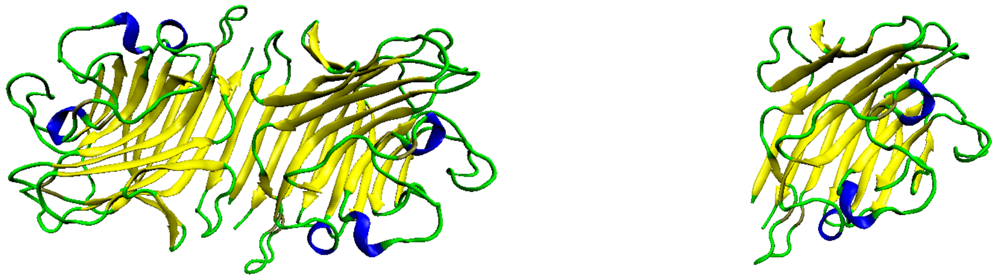

**Figure S41.** Pea Lectin Secondary Structure. PDB 1OFS [73] structure showing secondary structure elements: blue cartoons/coils correspond to $3_{10}$- and α-helices ($3_{10}$-helices chains A & C: 98–100, 111–113; α-helices B & D chains: 12–15), the yellow tapes are β-sheets: (chains A & C: 2–8, 18–22, 25–27, 30–34, 41–46, 50–51, 62–72, 82–89, 116–121, 136–141, 160–167, 172–179; B & D chains: 2–9, 19–27, 34–46), and the thin green ropes are turns and other structures. (**Left**) Full tetramer; (**Right**) Dimer used in CD calculations. The CATH fold classification [53] is mainly beta/sandwich.

**Table S15.** CD Analysis of Pea Lectin. The DInaMo calculations are for the minimized or rebuilt structure using CDCALC or CAPPS. All RMSDs are calculated between 180 and 210 nm.

| CD Method | Peak Wave-Length (nm) | Δε ($M^{-1}\cdot cm^{-1}$) | Peak Wave-Length (nm) | Δε ($M^{-1}\cdot cm^{-1}$) | Peak Wave-Length (nm) | Δε ($M^{-1}\cdot cm^{-1}$) | RMSD ($M^{-1}\cdot cm^{-1}$) |
|---|---|---|---|---|---|---|---|
| [a] SRCD | 175 | −4.47 | 196 | 5.05 | 226 | −1.58 | 0.000 |
| [b] 4000 Ho | 184 | −0.96 | 198 | 3.30 | 209 | −1.51 | 2.069 |
| [b] 6000 Ho | 182 | −0.48 | 198 | 1.45 | 213 | −0.61 | 2.478 |
| [b] 4000 Hx | 183 | −0.72 | 198 | 3.02 | 210 | −1.68 | 2.183 |
| [b] 6000 Hx | 181 | −0.36 | 198 | 1.31 | 213 | −0.70 | 2.535 |
| [b] 4000 Hy | 183 | −1.04 | 198 | 3.12 | 210 | −1.48 | 1.975 |
| [b] 6000 Hy | 181 | −0.49 | 197 | 1.45 | 213 | −0.60 | 2.417 |
| [b] 4000 Jo | 189 | −0.90 | 204 | 3.09 | 216 | −1.42 | 2.857 |
| [b] 6000 Jo | 187 | −0.45 | 204 | 1.36 | 220 | −0.57 | 2.799 |
| [b] 4000 Jx | 189 | −0.73 | 205 | 2.76 | 218 | −1.68 | 2.928 |
| [b] 6000 Jx | 187 | −0.36 | 205 | 1.20 | 221 | −0.73 | 2.857 |

**Table S15.** *Cont.*

| CD Method | Peak Wave-Length (nm) | Δε ($M^{-1}\cdot cm^{-1}$) | Peak Wave-Length (nm) | Δε ($M^{-1}\cdot cm^{-1}$) | Peak Wave-Length (nm) | Δε ($M^{-1}\cdot cm^{-1}$) | RMSD ($M^{-1}\cdot cm^{-1}$) |
|---|---|---|---|---|---|---|---|
| [b] 4000 Jy | 187 | −0.20 | 207 | 0.76 | not observed | – | 3.362 |
| [b] 6000 Jy | 196 | −0.30 | 209 | 0.31 | not observed | – | 3.243 |
| [b] 4000 OL | 182 | −0.25 | 205 | 0.67 | not observed | – | 3.228 |
| [b] 6000 OL | 193 | −0.24 | 207 | 0.30 | not observed | – | 3.168 |
| [c] B09:1 | 179 | −2.93 | 197 | 5.17 | 220 | −1.35 | 0.373 |
| [d] B09:2 | 175 | −5.56 | 197 | 5.23 | 220 | −1.44 | 0.741 |
| [e] B09:3 | 177 | −6.78 | 198 | 7.15 | 221 | −1.14 | 2.084 |

[a] SRCD from the PCDDB code CD0000053000 [44,47]; [b] CDCALC using PDB structure 1OFS [73] chains A and B minimized via NAMD/CHARMM22 and 5000 conjugate gradient steps. The hydrogens on all $-CH_3$ groups are ignored; [c] Matrix method using *ab initio* parameters including only the protein backbone transitions on PDB code 1OFS [55]; [d] Matrix method using *ab initio* parameters including protein backbone and charge-transfer transitions on PDB code 1OFS [55]; [e] Matrix method using *ab initio* parameters including protein backbone, charge-transfer and side chain transitions on PDB code 1OFS [55]. Grey highlight represents the lowest RMSD for each category.

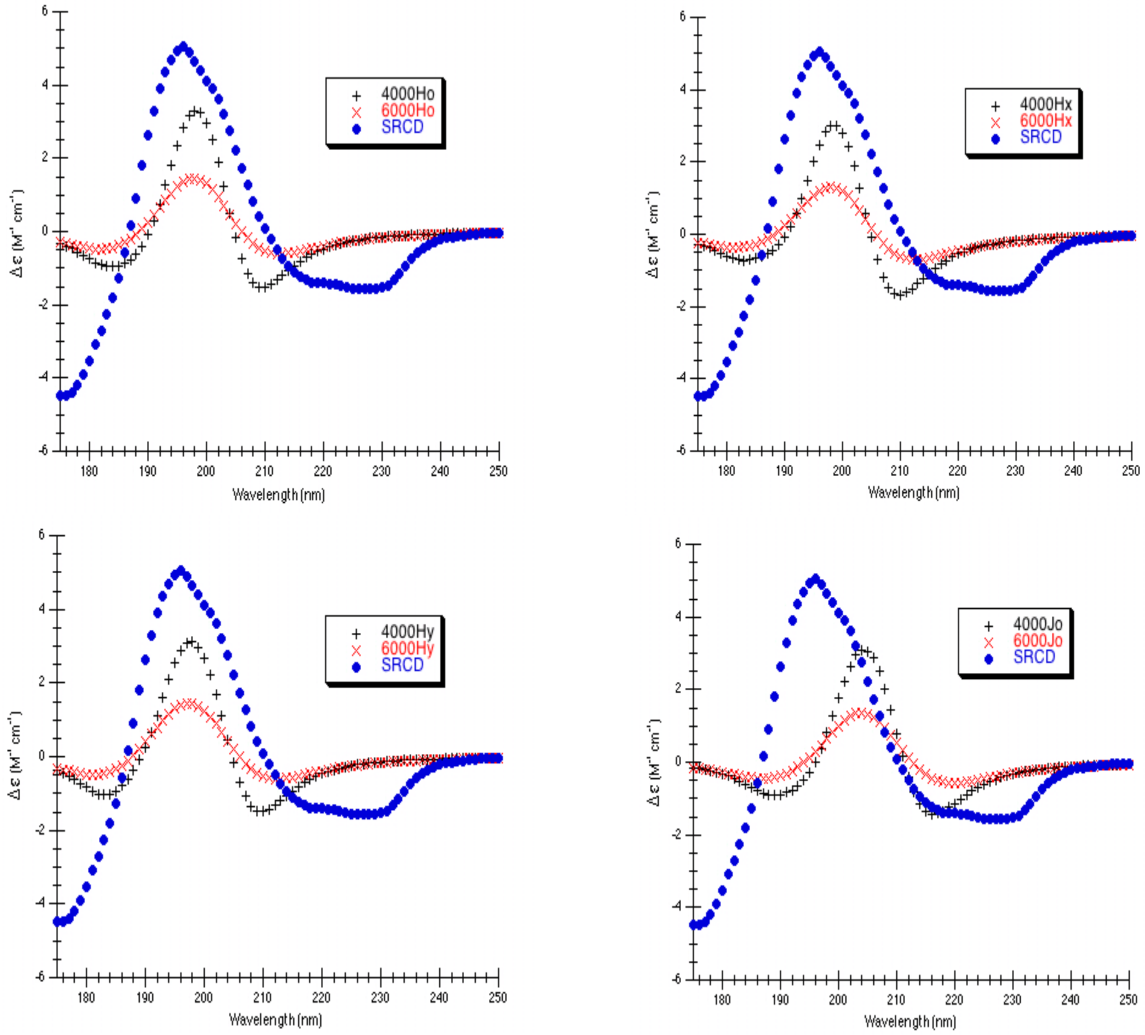


**Figure S42.** *Cont.*

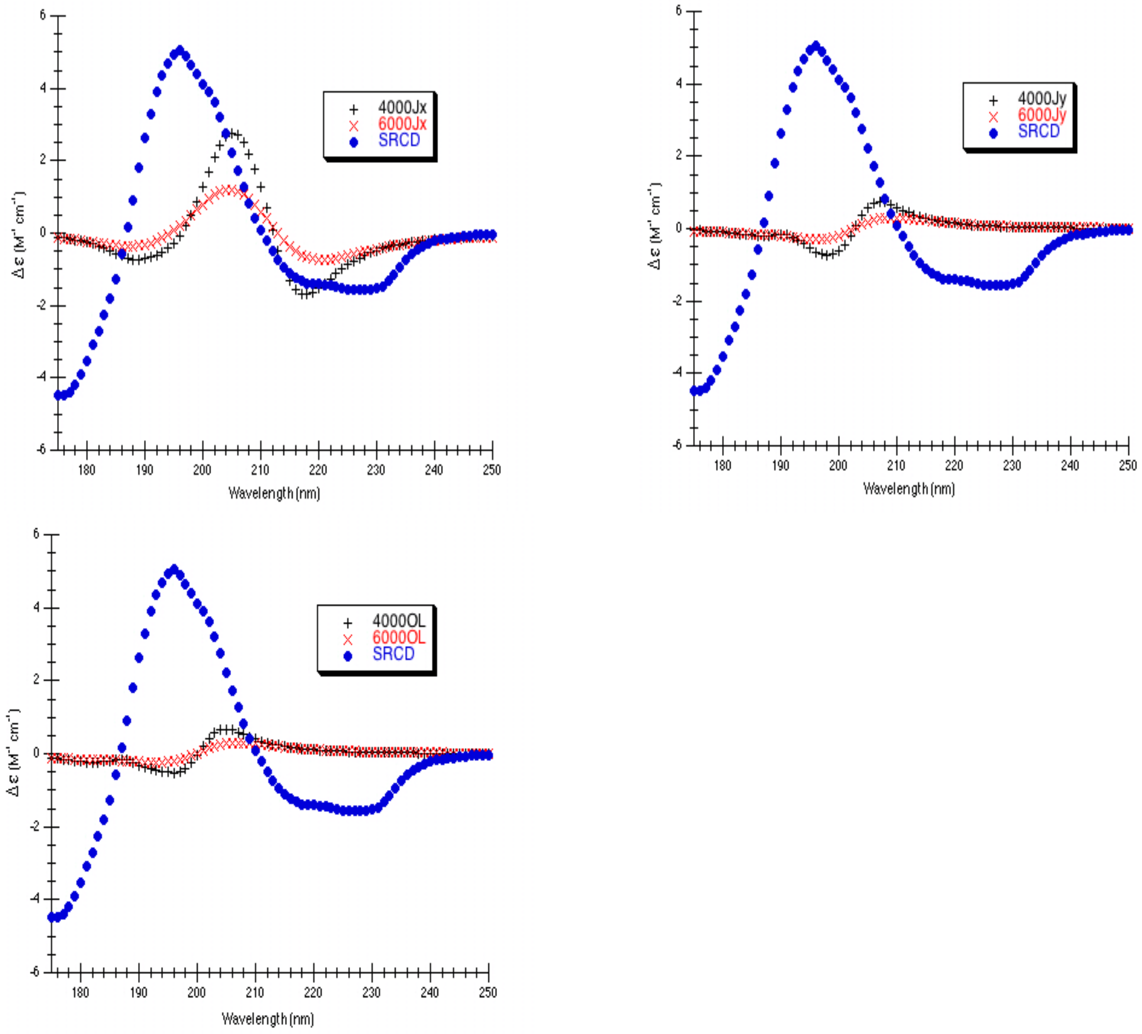


**Figure S42.** Pea Lectin Predicted CD Using CDCALC. The PDB 1OFS [73] A & B chains were minimized with 5000 conjugate gradient steps using NAMD/CHARMM22. Calculated spectra ignore all $-CH_3$ group hydrogens. Bandwidths are 4000 (+) and 6000 (×) $cm^{-1}$. The blue dots (•) are the experimental SRCD (CD0000053000) [1,2]. The CATH fold classification [53] is mainly beta/sandwich.

Avidin: PDB code 2A8G [74] is a dimer of the native homotetramer with each chain having 126 amino acids (Figure S43). Avidin has a high affinity for binding biotin and will also bind 8-oxodeoxyguanosin and related bases [74]. CATH classifies each monomer as a single domain that is mainly beta/beta barrel [53]. The PCDDB describes the secondary structure as 7.1% $3_{10}$-helix, 49.2% β-strand, 3.9% bonded turn, 13.4% bend, and 26.4% irregular [44].

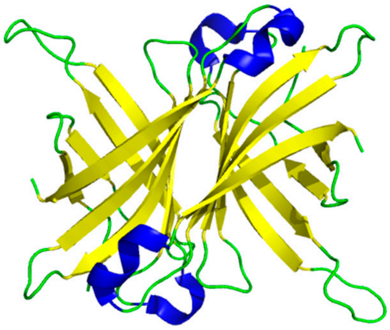

**Figure S43.** Avidin Secondary Structure. PDB code 2A8G [74] dimer showing secondary structure elements: the short blue cartoons/coil correspond to $3_{10}$-helices (55–59, 105–111 in each chain), the yellow tapes are β-sheets (8–12, 17–20, 28–35, 45–53, 63–69, 72−85, 91–100, 113–122, 8–12, 17–20, 28–35, 45–53, 63–69, 72–85, 91–100, 113–122 in each chain), and the thin green ropes are turns and other structures. The CATH fold classification [53] is mainly beta/beta barrel.

**Table S16.** CD Analysis of Avidin. The DInaMo calculations are for the minimized or rebuilt structure using CDCALC or CAPPS. All RMSDs are calculated between 180 and 210 nm.

| CD Method | Peak Wavelength (nm) | Δε ($M^{-1}\cdot cm^{-1}$) | Peak Wavelength (nm) | Δε ($M^{-1}\cdot cm^{-1}$) | RMSD ($M^{-1}\cdot cm^{-1}$) |
|---|---|---|---|---|---|
| [a] SRCD | 197 | 2.03 | 214 | −0.04 | 0.000 |
| [b] 4000 Ho | 200 | 5.17 | 211 | −1.00 | 2.573 |
| [b] 6000 Ho | 200 | 2.36 | 216 | −0.23 | 2.311 |
| [b] 4000 Hx | 200 | 5.80 | 212 | −0.92 | 2.836 |
| [b] 6000 Hx | 200 | 2.72 | 218 | −0.16 | 2.343 |
| [b] 4000 Hy | 200 | 5.04 | 211 | −1.42 | 2.462 |
| [b] 6000 Hy | 200 | 2.33 | 216 | −0.30 | 2.238 |
| [b] 4000 Jo | 206 | 4.85 | 218 | −0.94 | 3.418 |
| [b] 6000 Jo | 206 | 2.21 | 224 | −0.94 | 2.720 |
| [b] 4000 Jx | 207 | 5.34 | 220 | −0.94 | 3.699 |
| [b] 6000 Jx | 207 | 2.58 | 226 | −0.18 | 2.840 |
| [b] 4000 Jy | 205 | 1.90 | not observed | – | 2.914 |
| [b] 6000 Jy | 207 | 0.79 | not observed | – | 2.796 |
| [b] 4000 OL | 201 | 2.26 | not observed | – | 2.556 |
| [b] 6000 OL | 202 | 0.88 | not observed | – | 2.598 |
| [c] 4000 Ho | 200 | 5.65 | 210 | −0.72 | 2.628 |
| [c] 6000 Ho | 200 | 2.41 | 216 | −0.11 | 2.313 |
| [c] 4000 Hx | 200 | 6.44 | 210 | −0.59 | 2.701 |
| [c] 6000 Hx | 200 | 2.70 | 218 | −0.06 | 2.092 |
| [c] 4000 Hy | 198 | 7.27 | 212 | −0.03 | 3.025 |

**Table S16.** *Cont.*

| CD Method | Peak Wavelength (nm) | Δε ($M^{-1}\cdot cm^{-1}$) | Peak Wavelength (nm) | Δε ($M^{-1}\cdot cm^{-1}$) | RMSD ($M^{-1}\cdot cm^{-1}$) |
|---|---|---|---|---|---|
| [c] 6000 Hy | 200 | 3.36 | not observed | – | 2.435 |
| [c] 4000 Jo | 204 | −0.44 | 210 | −0.83 | 3.392 |
| [c] 6000 Jo | 206 | −0.56 | 210 | −0.57 | 3.205 |
| [c] 4000 Jx | 204 | −0.11 | 212 | −0.64 | 3.238 |
| [c] 6000 Jx | 206 | −0.37 | 212 | −0.42 | 3.102 |
| [c] 4000 Jy | 206 | 0.39 | 216 | −0.53 | 3.421 |
| [c] 6000 Jy | 204 | −0.10 | 216 | −0.29 | 3.242 |
| [d] B09:1 | 197 | 5.31 | 216 | −0.87 | 2.736 |
| [e] B09:2 | 197 | 5.31 | 218 | −0.91 | 2.410 |
| [f] B09:3 | 197 | 8.22 | 222 | −0.65 | 4.115 |

[a] SRCD from the PCDDB code CD00000800 [44,47]. Note: Peaks at 179 nm and 229 nm are not listed for clarity; [b] CDCALC using PDB structure 2A8G [74] minimized via NAMD/CHARMM22 and 5000 conjugate gradient steps. The hydrogens on all $-CH_3$ groups are ignored; [c] CAPPS results using PDB structure 2A8G [74] that contained rebuilt secondary structures including hydrogens; the following residues were ignored: 54, 60–62, 112; [d] Matrix method using *ab initio* parameters including only the protein backbone transitions on PDB code 1RAV [55,117]. [e] Matrix method using *ab initio* parameters including protein backbone and charge-transfer transitions on PDB code 1RAV [55,117]. [f] Matrix method using *ab initio* parameters including protein backbone, charge-transfer and side chain transitions on PDB code 1RAV [55,117]. Grey highlight represents the lowest RMSD for each category.

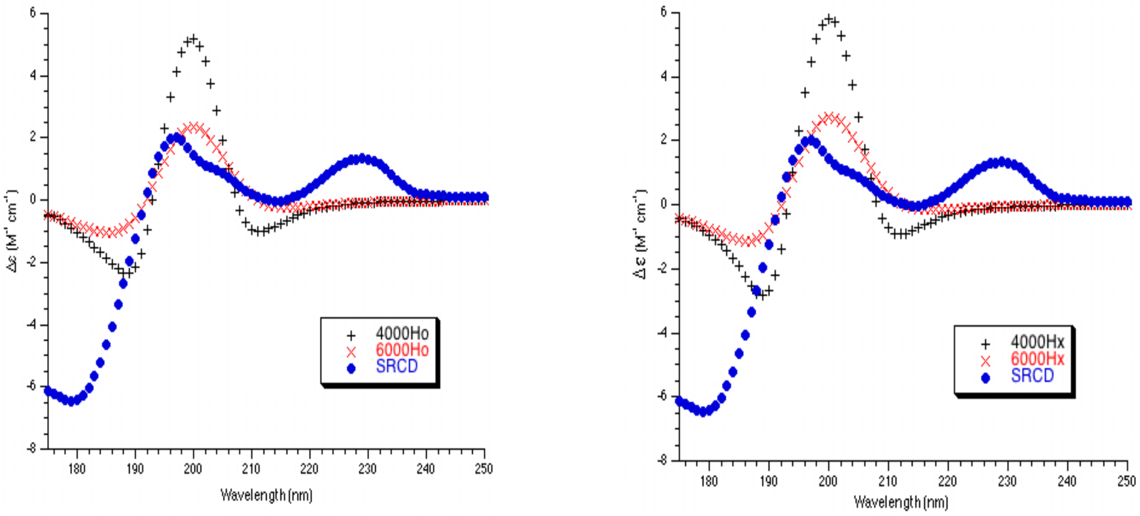


**Figure S44.** *Cont.*

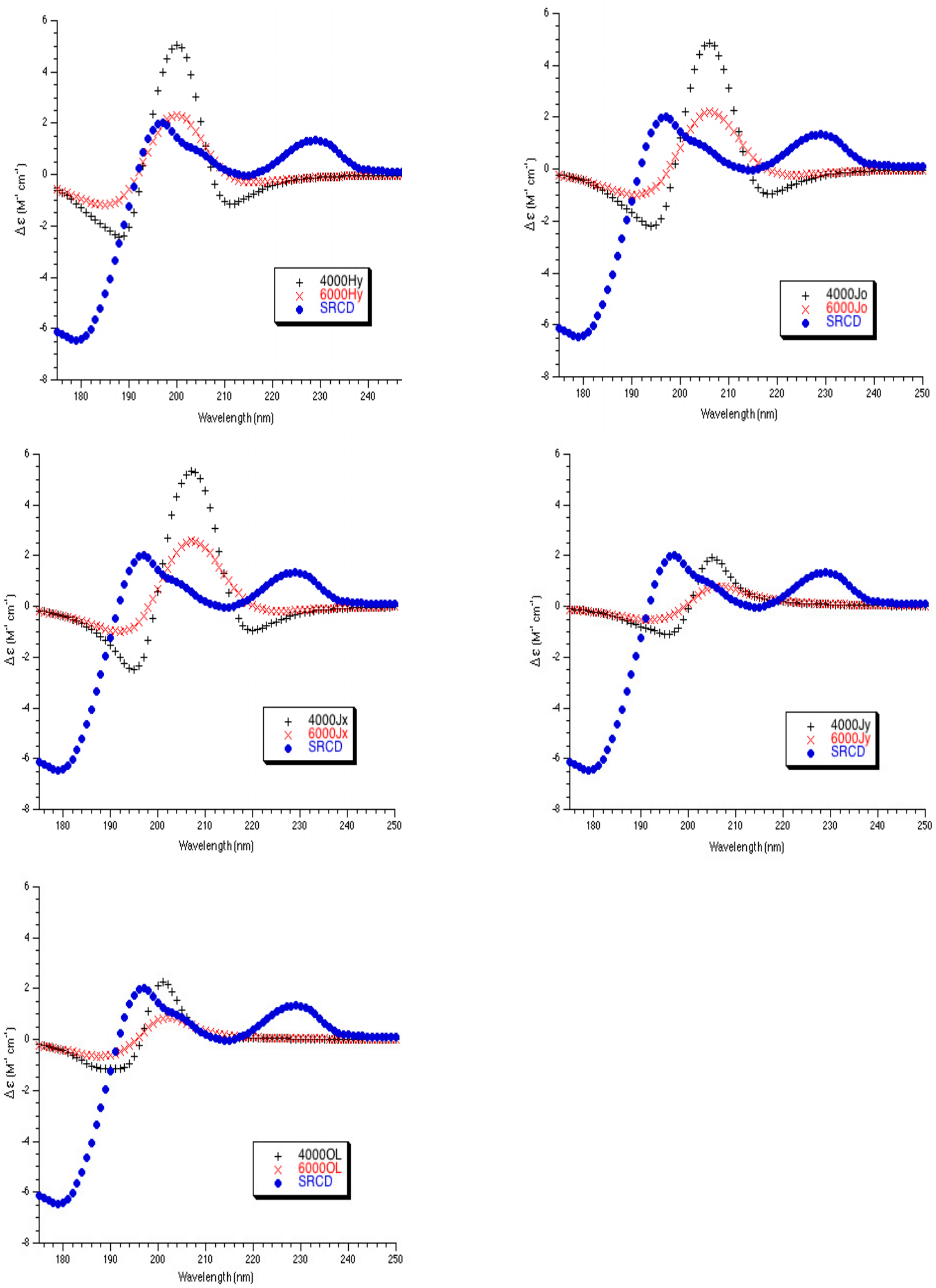


**Figure S44.** Avidin Predicted CD Using CDCALC. The PDB 2A8G [74] dimer was minimized with 5000 conjugate gradient steps using NAMD/CHARMM22. Calculated spectra ignore all $-CH_3$ group hydrogens. Bandwidths are 4000 (+) and 6,000 (×) $cm^{-1}$. The blue dots (•) are the experimental SRCD (CD000008000) [44,47]. The CATH fold classification [53] is mainly beta/beta barrel.

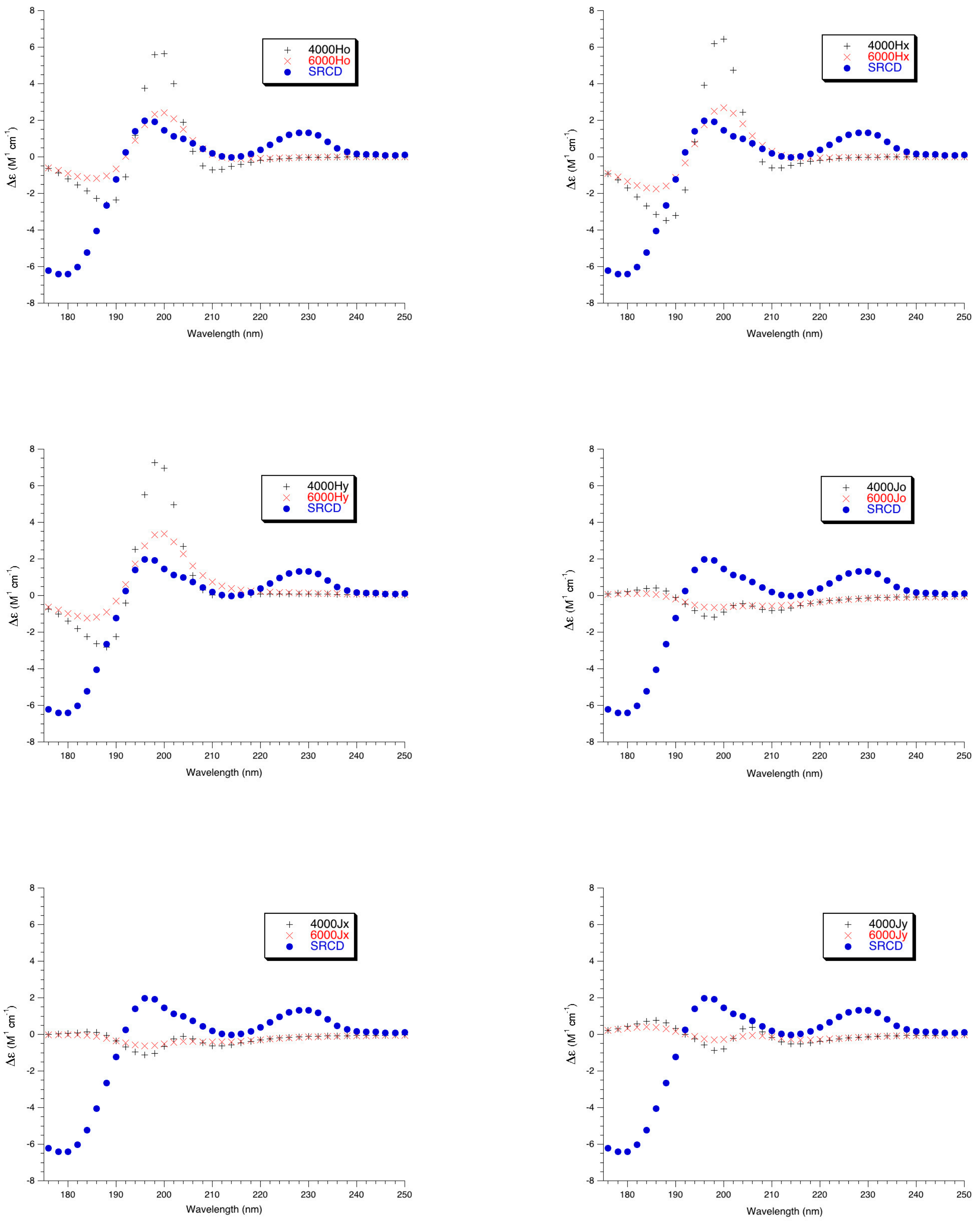


**Figure S45.** Avidin Predicted CD Using CAPPS. The PDB 2A8G [74] structure was rebuilt ignoring residues 54, 60–62, and 112. Bandwidths are 4000 (+) and 6000 (×) cm$^{-1}$. The blue dots (•) are the experimental SRCD (CD0000008000) [44,47]. The CATH fold classification [53] is mainly beta/beta barrel.

**Table S17.** CD Analysis of Outer Membrane Protein G. The DInaMo calculations are for the minimized or rebuilt structure using CDCALC.

| CD Method | Peak Wavelength (nm) | Δε ($M^{-1}\cdot cm^{-1}$) | Peak Wavelength (nm) | Δε ($M^{-1}\cdot cm^{-1}$) | RMSD ($M^{-1}\cdot cm^{-1}$) |
|---|---|---|---|---|---|
| [a] SRCD | 190 | 7.07 | 216 | −3.13 | 0.000 |
| [b] 4000 Ho | 203 | 2.40 | 213 | −0.55 | 4.104 |
| [b] 6000 Ho | 203 | 0.93 | 217 | −0.14 | 3.973 |
| [b] 4000 Hx | 204 | 2.16 | 213 | −0.82 | 4.798 |
| [b] 6000 Hx | 204 | 0.57 | 216 | −0.33 | 4.480 |
| [b] 4000 Hy | 203 | 2.51 | 213 | −0.59 | 4.301 |
| [b] 6000 Hy | 203 | 1.01 | 218 | −0.16 | 4.108 |
| [b] 4000 Jo | 209 | 2.22 | 220 | −0.52 | 4.326 |
| [b] 6000 Jo | 209 | 0.87 | 225 | −0.13 | 4.126 |
| [b] 4000 Jx | 211 | 2.29 | 221 | −0.78 | 4.493 |
| [b] 6000 Jx | 211 | 0.79 | 225 | −0.27 | 4.282 |
| [b] 4000 Jy | 207 | 1.41 | not observed | − | 4.176 |
| [b] 6000 Jy | 209 | 0.64 | not observed | − | 4.049 |
| [b] 4000 OL | 202 | 1.37 | 213 | −0.25 | 4.342 |
| [b] 6000 OL | 204 | 0.33 | 217 | −0.10 | 4.200 |

[a] SRCD from the Protein Circular Dichroism Data Bank CD0000118000 [44,59]; [b] CDALC using PDB structure 2IWV [62] that was minimized via NAMD/CHARMM22 10,000 steps. Hs attached to $-CH_3$s are ignored. Grey highlight represents the lowest RMSD for each category.

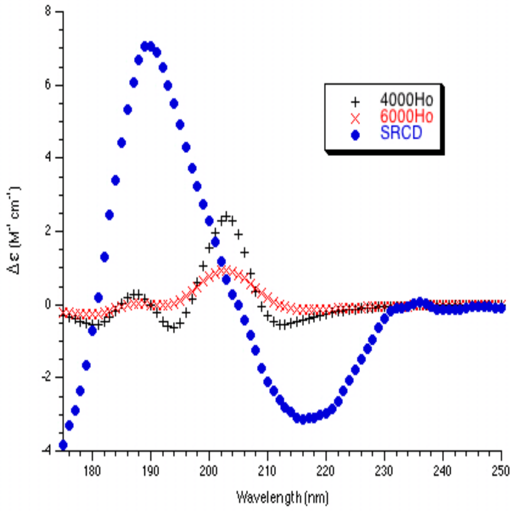


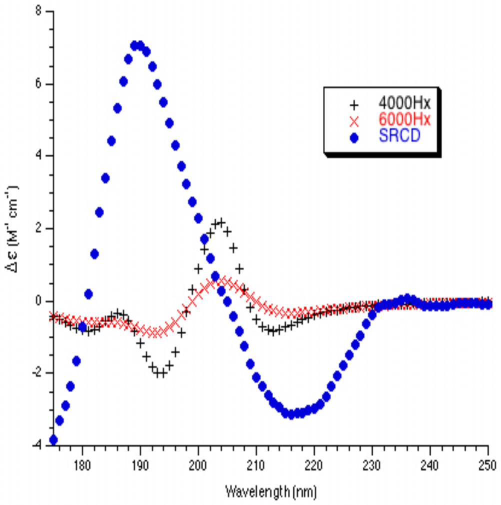


**Figure S46.** *Cont.*

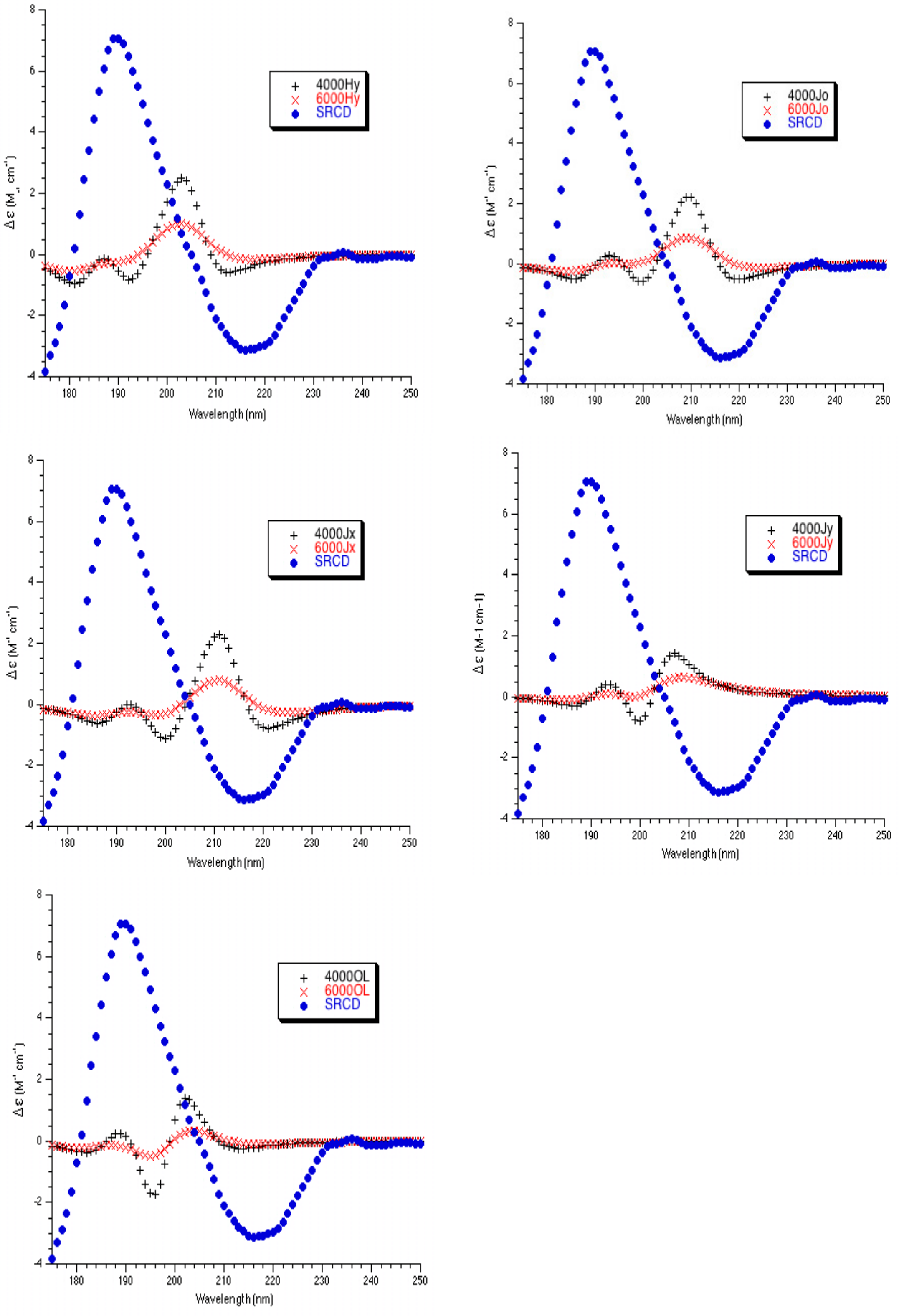


**Figure S46.** Outer Membrane Protein G Predicted CD Using CDCALC. The PDB 2IWV [62] structure was minimized via 10,000 conjugate gradient steps by NAMD/CHARMM22. All $-CH_3$ group hydrogens are ignored. Bandwidths are 4000 (+) and 6000 (×) $cm^{-1}$. The blue dots (•) are the experimental SRCD (CD0000119000) [44,59]. The CATH fold classification [53] is mainly beta/beta barrel.

Monellin: The very sweet-tasting protein monellin (PDB code 1MOL) (Figure S47) has 94 amino acids and has been fused to make a monomer (Figure 6), but has two distinct structures in the crystal [88]. In its natural state it is a heterodimer of two chains (one 44 amino acids and the other 50 amino acids) [118]. Herein the monomer structure 1MOL is used; the A chain is used for minimization (5000 steps of conjugate gradients) and simulation of the CD. CATH classified 1MOL as an alpha-beta/roll [53]. The PCDDB describes the 1MOL secondary structure as 17.0% α-helix, 52.1% β-strand, 10.1% β-turn, 3.7% bend, and 17.0% irregular [44].

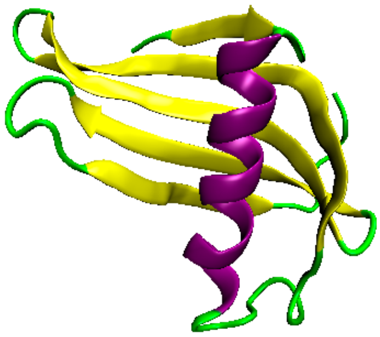

**Figure S47.** Monellin Secondary Structure. PDB code 1MOL [88] chain A showing secondary structure elements: thick purple cartoons/coils correspond to α-helices (10–26), the yellow tapes are β-sheets, (2–6, 34–48, 52–64, 67–76, 82–88), and the thin green ropes are turns and other structures. The CATH fold classification [53] is alpha beta/roll.

**Table S18.** CD Analysis of Monellin. The DInaMo calculations are for the minimized or rebuilt structure using CDCALC. All RMSDs are calculated between 180 and 210 nm.

| CD Method | Peak Wavelength (nm) | Δε ($M^{-1}\cdot cm^{-1}$) | Peak Wavelength (nm) | Δε ($M^{-1}\cdot cm^{-1}$) | RMSD ($M^{-1}\cdot cm^{-1}$) |
|---|---|---|---|---|---|
| [a] SRCD | 190 | 3.75 | 213 | −3.32 | 0.000 |
| [b] 4000 Ho | 188 | 2.18 | 209 | −2.75 | 1.503 |
| [b] 6000 Ho | 188 | 1.04 | 211 | −1.39 | 1.701 |
| [b] 4000 Hx | 188 | 2.88 | 209 | −2.74 | 1.507 |
| [b] 6000 Hx | 187 | 1.47 | 211 | −1.36 | 1.652 |
| [b] 4000 Hy | 189 | 2.50 | 209 | −2.86 | 1.404 |
| [b] 6000 Hy | 190 | 1.38 | 211 | −1.40 | 1.619 |
| [b] 4000 Jo | 194 | 2.03 | 216 | −2.58 | 2.234 |
| [b] 6000 Jo | 194 | 0.98 | 218 | −1.30 | 2.115 |
| [b] 4000 Jx | 193 | 2.18 | 217 | −2.66 | 1.996 |
| [b] 6000 Jx | 193 | 1.02 | 218 | −1.39 | 1.996 |
| [b] 4000 Jy | 195 | 3.45 | 213 | −1.84 | 2.122 |
| [b] 6000 Jy | 195 | 1.40 | 214 | −1.01 | 1.985 |
| [b] 4000 OL | 191 | 4.37 | 212 | −2.08 | 0.876 |
| [b] 6000 OL | 190 | 1.75 | 213 | −1.24 | 1.366 |

**Table S18.** *Cont.*

| CD Method | Peak Wavelength (nm) | Δε ($M^{-1}·cm^{-1}$) | Peak Wavelength (nm) | Δε ($M^{-1}·cm^{-1}$) | RMSD ($M^{-1}·cm^{-1}$) |
|---|---|---|---|---|---|
| [c] B09:1 | 190 | 3.49 | 214 | −3.10 | 3.938 |
| [d] B09:2 | 195 | 7.86 | 215 | −2.26 | 3.793 |
| [e] B09:3 | 195 | 6.88 | 214 | −2.47 | 4.966 |
| [f] SI | 191 | 8.63 | 211 | −3.19 | 3.362 |
| [g] SII | 189 | 3.73 | 217 | −0.96 | 1.501 |
| [h] SIII | 189 | 2.33 | 197 | −0.45 | 1.973 |

[a] SRCD from the PCDDB code CD0000046000 [44,47]; [b] CDCALC using PDB structure 1MOL [88] minimized via NAMD/CHARMM22 and 5000 conjugate gradient steps. The hydrogens on all $-CH_3$ groups are ignored; [c] Matrix method using *ab initio* parameters including only the protein backbone transitions on PDB code 1MOL [55,88]; [d] Matrix method using *ab initio* parameters including protein backbone and charge-transfer transitions on PDB code 1MOL [55,88]; [e] Matrix method using *ab initio* parameters including protein backbone, charge-transfer and side chain transitions on PDB code 1MOL [55,88]; [f] Exciton Hamiltonian with electrostatic fluctuations & PDB code 1MOL [88] based on a single conformation convoluted with a Gaussian envelop [29]; [g] Exciton Hamiltonian with electrostatic fluctuations & PDB code 1MOL [88] based on 2000 MD snapshots that consider the electrostatic potential from all surroundings [29]; [h] Exciton Hamiltonian with electrostatic fluctuations & PDB code 1MOL [88] simulated spectrum that take account of only peptide groups [29]. Grey highlight represents the lowest RMSD for each category.

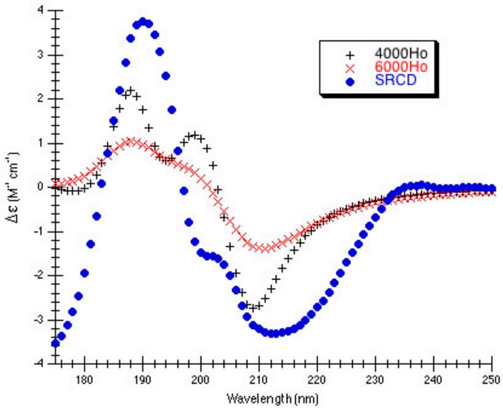


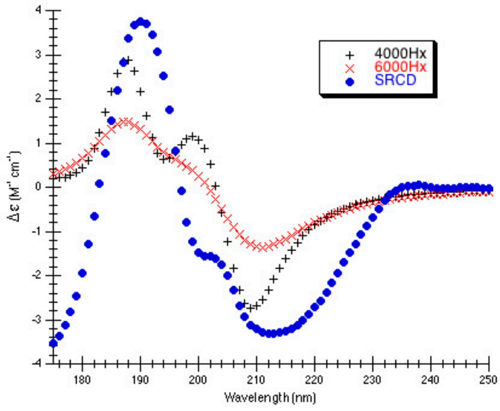


**Figure S48.** *Cont.*

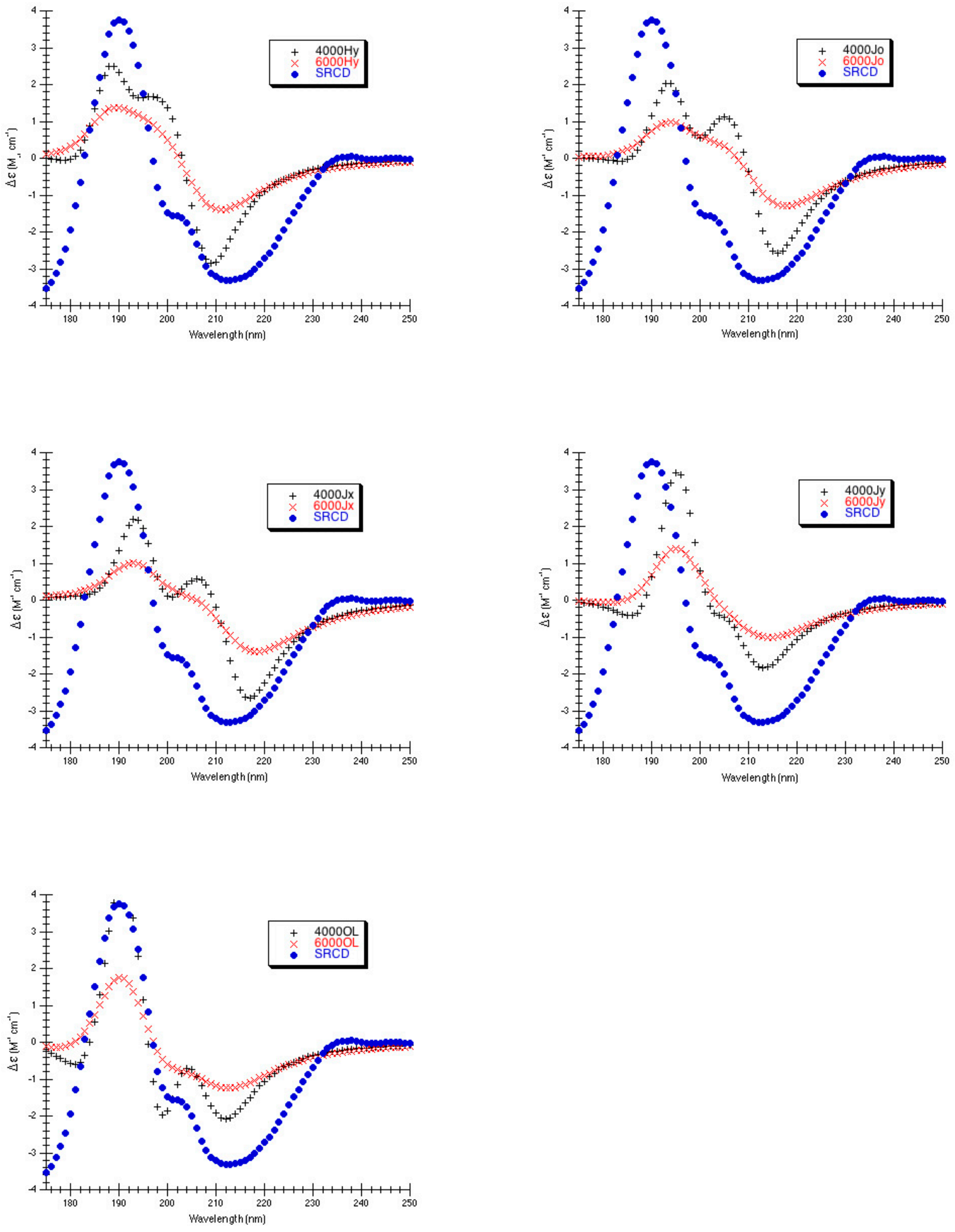


**Figure S48.** Monellin Predicted CD Using CDCALC. The PDB 1MOL [88] structure was minimized with 5000 conjugate gradient steps using NAMD/CHARMM22. Calculated spectra ignore all $-CH_3$ group hydrogens. Bandwidths are 4000 (+) and 6000 (×) $cm^{-1}$. The blue dots (•) are the experimental SRCD (CD0000046000) [44,47]. The CATH fold classification [53] is alpha beta/roll.

Ferredoxin: PDB code 2FDN [80] (Figure S49) is an electron-transfer protein from *Clostridium pasteurianum* that links hydrogenase with a variety of electron donors or acceptors [119]. It is an iron-containing protein that functions as an electron-mediating catalyst for the biological production or utilization of hydrogen gas by bacteria [120]. Ferredoxin has two iron-sulfur clusters within the small monomeric protein of 55 amino acids. The A chain of the monomeric structure is used for minimization, without considering the iron-sulfur cluster. CATH classifies ferredoxin as a single domain with alpha-beta/2-layer sandwich architecture [53]. The PCDDB describes the secondary structure as 7.3% α-helix, 5.5% $3_{10}$-helix, 18.2% β-strand, 21.8% bonded turn, 5.5% bend, and 41.8% irregular [44].

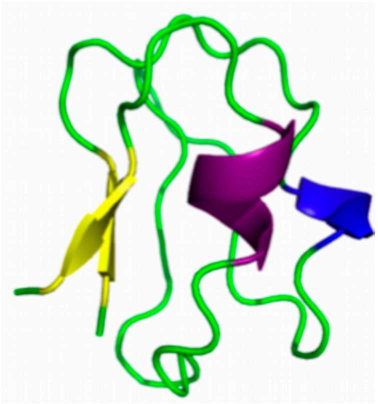

**Figure S49.** Ferredoxin Secondary Structure. PDB 2FDN [80] structure showing secondary structure elements: thick purple cartoons/coils correspond to α-helices (42–46), the short blue cartoons/coil correspond to $3_{10}$-helices (15–17) and the yellow tapes are β-sheets, (2–4, 52–54), and the thin green ropes are turns and other structures. The iron sulfur clusters are not shown because they are not included in the CD calculation. The CATH fold classification [53] is alpha beta/2-layer sandwich.

**Table S19.** CD Analysis of Ferredoxin. The DInaMo calculations are for the minimized or rebuilt structure using CDCALC. All RMSDs are calculated between 180 and 210 nm.

| CD Method | Peak Wavelength (nm) | Δε ($M^{-1}·cm^{-1}$) | Peak Wavelength (nm) | Δε ($M^{-1}·cm^{-1}$) | RMSD ($M^{-1}·cm^{-1}$) |
|---|---|---|---|---|---|
| [a] SRCD | 185 | 1.03 | 201 | −6.37 | 0.000 |
| [b] 4000 Ho | 185 | 3.42 | 207 | −3.84 | 2.509 |
| [b] 6000 Ho | 183 | 2.06 | 207 | −2.54 | 2.654 |
| [b] 4000 Hx | 182 | 2.48 | 206 | −4.52 | 1.338 |
| [b] 6000 Hx | 180 | 1.26 | 205 | −3.27 | 1.743 |
| [b] 4000 Hy | 182 | 2.39 | 206 | −4.14 | 1.643 |
| [b] 6000 Hy | 180 | 1.22 | 206 | −2.90 | 4.241 |
| [b] 4000 Jo | 190 | 3.22 | 214 | −3.59 | 3.901 |
| [b] 6000 Jo | 188 | 1.94 | 214 | −2.38 | 3.641 |
| [b] 4000 Jx | 187 | 2.53 | 214 | −4.11 | 2.610 |
| [b] 6000 Jx | 186 | 1.12 | 212 | −2.98 | 2.652 |

**Table S19.** *Cont.*

| CD Method | Peak Wavelength (nm) | Δε ($M^{-1}$·$cm^{-1}$) | Peak Wavelength (nm) | Δε ($M^{-1}$·$cm^{-1}$) | RMSD ($M^{-1}$·$cm^{-1}$) |
|---|---|---|---|---|---|
| [b] 4000 Jy | 192 | 5.46 | 209 | −4.66 | 5.076 |
| [b] 6000 Jy | 191 | 3.14 | 211 | −2.89 | 4.241 |
| [b] 4000 OL | 189 | 6.66 | 205 | −5.19 | 4.627 |
| [b] 6000 OL | 188 | 3.86 | 209 | −3.29 | 3.782 |
| [c] B09:1 | 195 | 4.22 | 214 | −1.25 | 5.685 |
| [d] B09:2 | 194 | 3.99 | 214 | −1.45 | 5.539 |
| [e] B09:3 | 194 | 6.88 | 211 | −2.19 | 6.791 |

[a] SRCD from the PCDDB code CD0000032000 [44,47]; [b] CDCALC using PDB structure 2FDN [80] minimized via NAMD/CHARMM22 1000 conjugate gradient steps. The hydrogens on all $-CH_3$ groups are ignored; [c] Matrix method using *ab initio* parameters including only the protein backbone transitions on PDB code 2FDN [55,80]; [d] Matrix method using *ab initio* parameters including protein backbone and charge-transfer transitions on PDB code 2FDN [55,80]; [e] Matrix method using *ab initio* parameters including protein backbone, charge-transfer and side chain transitions on PDB code 2FDN [55,80]. Grey highlight represents the lowest RMSD for each category.

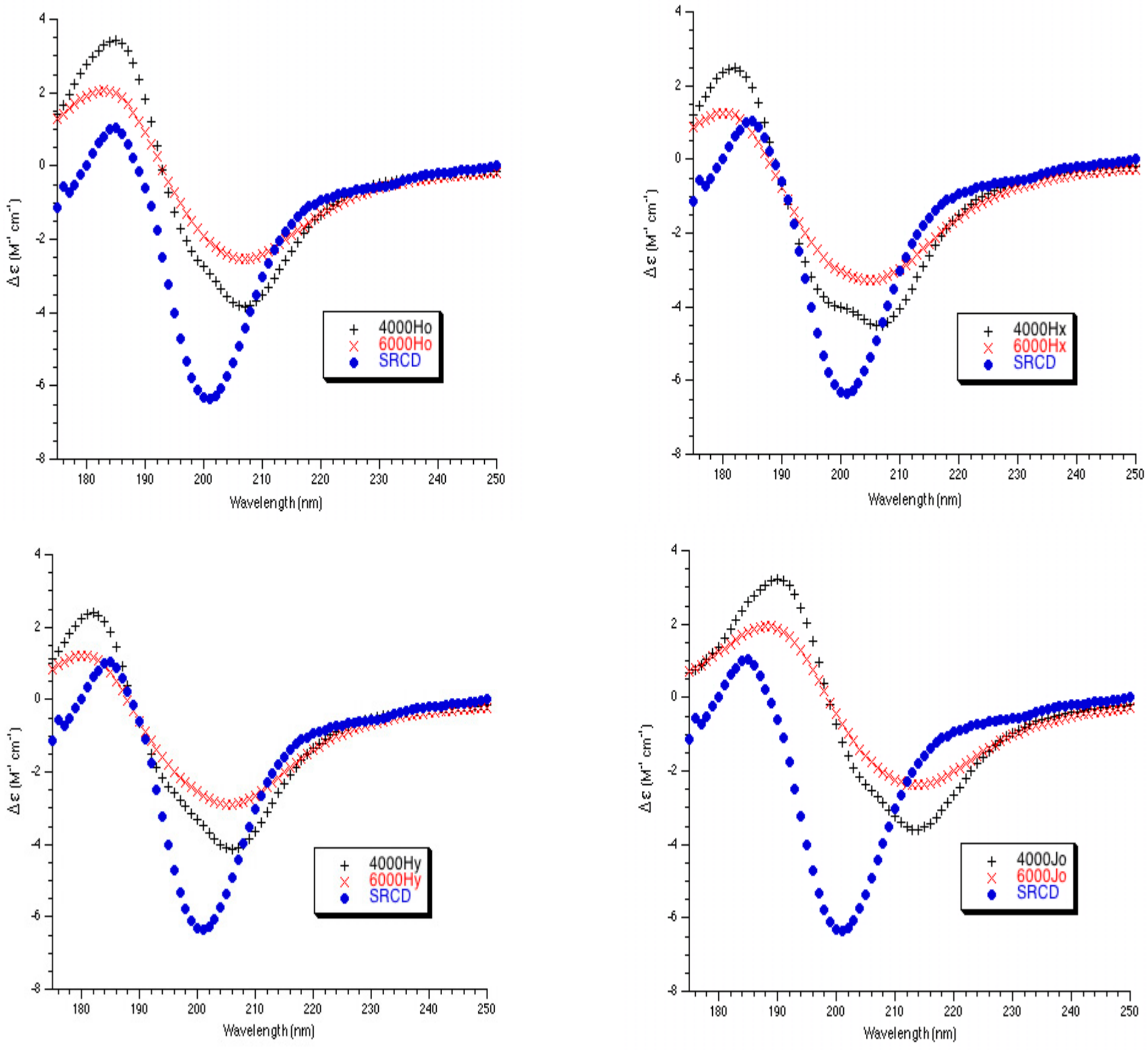


**Figure S50.** *Cont.*

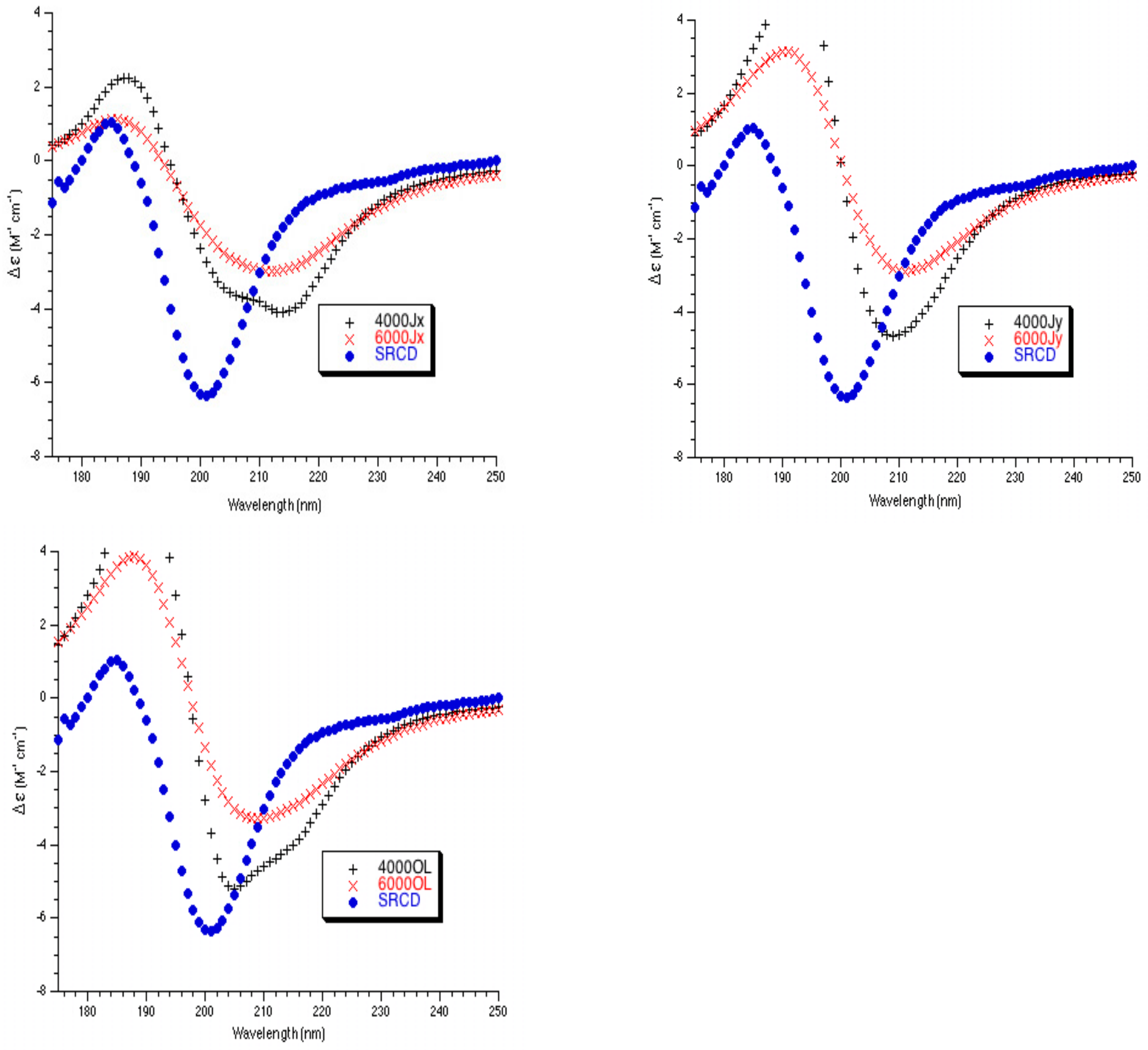


**Figure S50.** Ferredoxin Predicted CD Using CDCALC. The PDB 2FDN [80] structure was minimized with 1000 conjugate gradient steps using NAMD/CHARMM22. Calculated spectra ignore all $-CH_3$ group hydrogens. Bandwidths are 4000 (+) and 6000 (×) $cm^{-1}$. The blue dots (•) are the experimental SRCD (CD0000032000) [44,47]. The CATH fold classification [53] is alpha-beta/2-layer sandwich.

Triose Phosphate Isomerase: PDB code 7TIM from *Saccharomyces cerevisiae* (Figure S51) is a homodimer glycolytic enzyme that catalyzes the interconversion of dihydroxyacetone phosphate to glyceraldehyde-3-phosphate [64]. Each monomer of triose phosphate isomerase has 247 residues that CATH classifies as a single domain that is alpha−beta with architecture of alpha-beta-barrel [53]. The PCCDB describes the secondary structure as 38.9% α-helix, 5.7% $3_{10}$-helix, 16.2% β-strand, 0.8% β-bridge, 10.7% bonded turn, 6.3% bend, and 21.5% irregular [44].

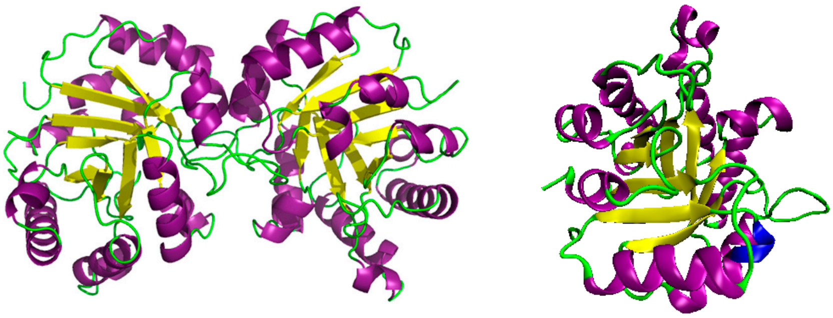

**Figure S51.** Triose Phosphate Isomerase Secondary Structure. PDB 7TIM structure [64] showing secondary structure elements: thick purple cartoons/coils correspond to α-helices (17–29, 44–53, 80–86, 96–101, 106–118, 131–135, 139–150, 178–195, 198–203, 214–219, 232–236, 238–244), the short blue cartoons/coil correspond to $3_{10}$−helices (15−17), the yellow tapes are β-sheets (5–10, 36–41, 59–63, 90–93, 122–127, 160–164, 206–209, 228–231), and the thin green ropes are turns and other structures. (**Left**) Homodimer; (**Right**) Monomer used in CD calculation. The CATH fold classification [53] for triose phosphate isomerase is alpha beta/alpha-beta barrel.

**Table S20.** CD Analysis of Triose Phosphate Isomerase. The DInaMo calculations are for the minimized or rebuilt structure using CDCALC or CAPPS. All RMSDs are calculated between 180 and 210 nm.

| CD Method | Peak Wavelength (nm) | Δε ($M^{-1}\cdot cm^{-1}$) | Peak Wavelength (nm) | Δε ($M^{-1}\cdot cm^{-1}$) | RMSD ($M^{-1}\cdot cm^{-1}$) |
|---|---|---|---|---|---|
| [a] SRCD | 190 | 7.85 | 217 | −5.06 | 0.000 |
| [b] 4000 Ho | 190 | 7.51 | 206 | −7.62 | 3.116 |
| [b] 6000 Ho | 188 | 4.19 | 207 | −4.20 | 2.924 |
| [b] 4000 Hx | 189 | 7.63 | 206 | −8.54 | 3.768 |
| [b] 6000 Hx | 187 | 4.41 | 207 | −4.80 | 3.187 |
| [b] 4000 Hy | 189 | 6.82 | 206 | −6.98 | 3.049 |
| [b] 6000 Hy | 187 | 4.06 | 207 | −3.92 | 3.053 |
| [b] 4000 Jo | 195 | 7.03 | 212 | −7.14 | 2.202 |
| [b] 6000 Jo | 194 | 3.93 | 214 | −3.941 | 2.575 |
| [b] 4000 Jx | 195 | 7.02 | 213 | −7.79 | 1.899 |
| [b] 6000 Jx | 193 | 4.10 | 214 | −4.39 | 2.360 |
| [b] 4000 Jy | 196 | 8.08 | 211 | −7.29 | 3.115 |
| [b] 6000 Jy | 195 | 3.96 | 212 | −3.79 | 2.893 |
| [b] 4000 OL | 192 | 10.70 | 207 | −8.80 | 3.037 |
| [b] 6000 OL | 191 | 5.45 | 209 | −4.72 | 1.840 |
| [c] 4000 Ho | 192 | 9.32 | 204 | −6.32 | 2.442 |
| [c] 6000 Ho | 190 | 5.14 | 206 | −3.13 | 2.073 |
| [c] 4000 Hx | 190 | 8.66 | 204 | −6.14 | 2.522 |
| [c] 6000 Hx | 190 | 4.90 | 206 | −3.01 | 2.283 |
| [c] 4000 Hy | 190 | 9.39 | 204 | −6.79 | 2.827 |

**Table S20.** *Cont.*

| CD Method | Peak Wavelength (nm) | Δε ($M^{-1}\cdot cm^{-1}$) | Peak Wavelength (nm) | Δε ($M^{-1}\cdot cm^{-1}$) | RMSD ($M^{-1}\cdot cm^{-1}$) |
|---|---|---|---|---|---|
| [c] 6000 Hy | 188 | 5.33 | 206 | −3.56 | 2.175 |
| [c] 4000 Jo | 196 | 6.08 | 208 | −4.06 | 2.995 |
| [c] 6000 Jo | 194 | 2.85 | 210 | −1.82 | 3.437 |
| [c] 4000 Jx | 196 | 5.68 | 206 | −3.99 | 2.793 |
| [c] 6000 Jx | 194 | 2.79 | 210 | −1.77 | 3.371 |
| [c]4000 Jy | 198 | 6.99 | 210 | −5.97 | 3.096 |
| [c] 6000 Jy | 196 | 3.35 | 212 | −2.96 | 3.140 |
| [d] B09:1 | 192 | 8.45 | 211 | −4.82 | 1.410 |
| [e] B09:2 | 192 | 8.30 | 211 | −5.23 | 1.562 |
| [f] B09:3 | 192 | 7.54 | 211 | −4.90 | 1.230 |
| [g] WS99:1 | 222 | 6.24 | 189 | 10.97 | 1.423 |
| [h] WS99:2 | 222 | 6.02 | 188 | 12.52 | 2.193 |

[a] SRCD from the PCDDB code CD0000070000 [44,47]; [b] CDCALC using PDB structure 7TIM [64] minimized via NAMD/CHARMM22 and 5,000 conjugate gradient steps. The hydrogens on all −$CH_3$ groups are ignored; [c] CAPPS results using PDB structure 7TIM that contained rebuilt secondary structures including hydrogens; the following residues were ignored: 2–4, 87–89, 119–121, 128–130, 136–138, 237; [d] Matrix method using ab intio parameters including only the protein backbone transitions on PDB code 7TIM [55,64]; [e] Matrix method using *ab initio* parameters including protein backbone and charge-transfer transitions on PDB code 7TIM [55,64]; [f] Matrix method using ab intio parameters including protein backbone, charge-transfer and side chain transitions on PDB code 7TIM [55,64]; [g] Matrix method on PDB code 1TIM [121] (according to [122]) using semi-empirical parameters for the π-π* and n-π* transitions excluding aromatic side chains [13]; [h] Matrix method on PDB code 1TIM [121] (according to [122]) using semi-empirical parameters for the π-π* and n-π * transitions including aromatic side chains [13]. Grey highlight represents the lowest RMSD for each category.

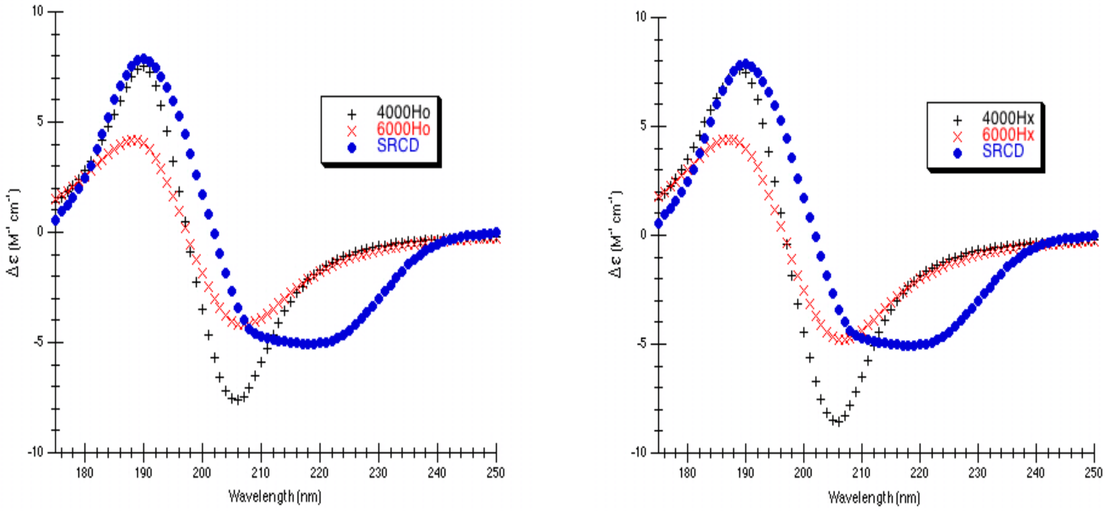


**Figure S52.** *Cont.*

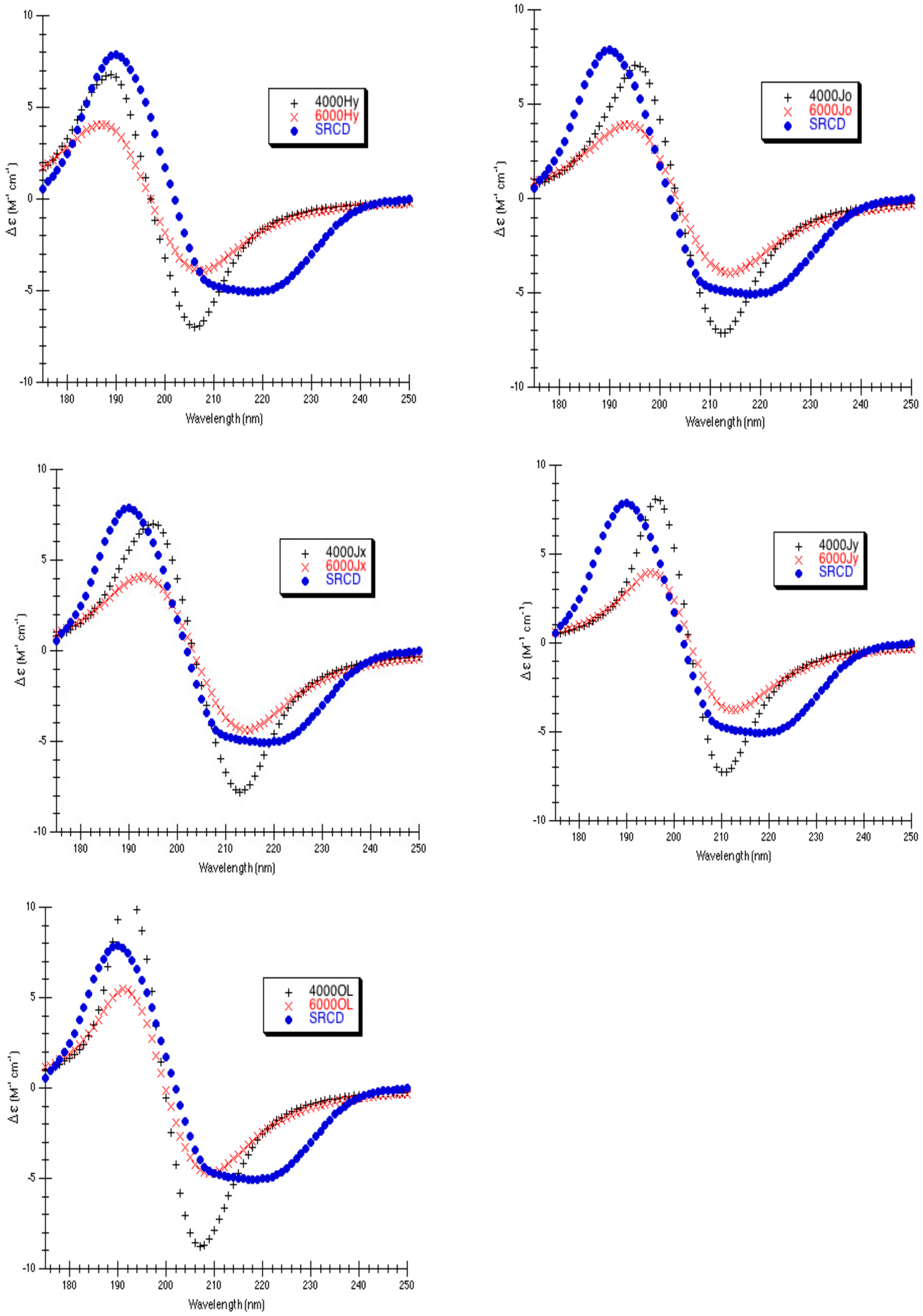


**Figure S52.** Triose Phosphate Isomerase Predicted CD Using CDCALC. The PDB 7TIM [64] structure was minimized with 5000 conjugate gradient steps using NAMD/CHARMM22. Calculated spectra ignore all $-CH_3$ group hydrogens. Bandwidths are 4000 (+) and 6000 (×) cm$^{-1}$. The blue dots (•) are the experimental SRCD (CD0000070000) [44,47]. The CATH fold classification [53] is alpha beta/ alpha-beta barrel.

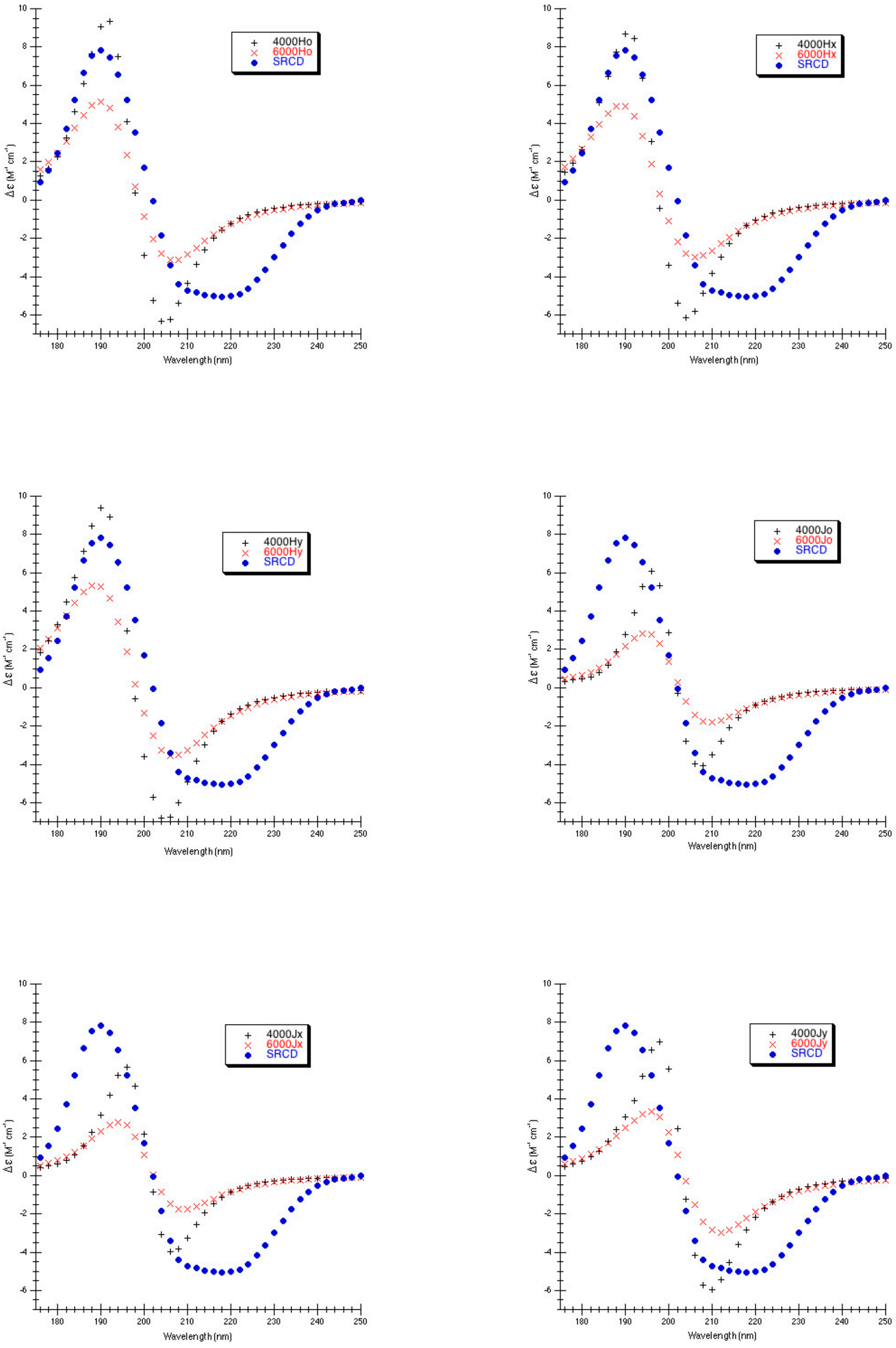


**Figure S53.** Triose Phosphate Isomerase Predicted CD Using CAPPS. The PDB 7TIM [64] structure was rebuilt ignoring residues 1–2 and 32–34. Bandwidths are 4000 (+) and 6000 (×) cm$^{-1}$. The blue dots (•) are the experimental SRCD (CD0000070000) [44,47]. The CATH fold classification [53] is alpha beta/ alpha-beta barrel.

**Table S21.** CD Analysis of Crambin. The DInaMo calculations are for the minimized or rebuilt structure using CDCALC or CAPPS. All RMSDs are calculated between 180 and 210 nm.

| CD Method | Peak Wavelength (nm) | Δε ($M^{-1}·cm^{-1}$) | Peak Wavelength (nm) | Δε ($M^{-1}·cm^{-1}$) | RMSD ($M^{-1}·cm^{-1}$) |
|---|---|---|---|---|---|
| [a] Conventional CD | 191 | 15.26 | 209 | −10.98 | 0.000 |
| [b] 4000 Ho | 193 | 9.13 | 207 | −9.26 | 4.470 |
| [b] 6000 Ho | 188 | 5.31 | 208 | −5.03 | 6.741 |
| [b] 4000 Hx | 188 | 9.55 | 207 | −10.09 | 4.830 |
| [b] 6000 Hx | 187 | 5.65 | 208 | −5.59 | 6.750 |
| [b] 4000 Hy | 188 | 8.35 | 207 | −9.05 | 4.817 |
| [b] 6000 Hy | 187 | 5.09 | 208 | −4.95 | 6.923 |
| [b] 4000 Jo | 194 | 8.58 | 213 | −8.68 | 6.433 |
| [b] 6000 Jo | 193 | 5.00 | 215 | −4.71 | 7.515 |
| [b] 4000 Jx | 194 | 8.68 | 214 | −9.11 | 6.419 |
| [b] 6000 Jx | 193 | 5.20 | 216 | −5.05 | 7.455 |
| [b] 4000Jy | 196 | 11.34 | 210 | −9.15 | 6.188 |
| [b] 6000 Jy | 195 | 5.70 | 212 | −4.73 | 7.163 |
| [b] 4000 OL | 192 | 13.66 | 207 | −10.93 | 0.776 |
| [b] 6000 OL | 190 | 7.17 | 209 | −6.08 | 5.035 |
| [c] 4000 Ho | 192 | 8.04 | 206 | −7.94 | 3.936 |
| [c] 6000 Ho | 190 | 4.55 | 208 | −9.95 | 6.788 |
| [c] 4000 Hx | 192 | 8.14 | 206 | −7.94 | 3.897 |
| [c] 6000 Hx | 190 | 4.62 | 208 | −3.97 | 6.778 |
| [c] 4000 Hy | 194 | 7.47 | 206 | −7.07 | 4.609 |
| [c] 6000 Hy | 192 | 4.23 | 208 | −3.35 | 7.147 |
| [c] 4000 Jo | 196 | 6.53 | 206 | −5.59 | 5.798 |
| [c] 6000 Jo | 194 | 3.16 | 208 | −2.76 | 7.833 |
| [c] 4000 Jx | 194 | 6.85 | 206 | −5.85 | 5.666 |
| [c] 6000 Jx | 194 | 3.28 | 208 | −2.80 | 7.775 |
| [c] 4000 Jy | 198 | 7.55 | 208 | −6.74 | 6.597 |
| [c] 6000 Jy | 196 | 3.57 | 210 | −3.29 | 7.876 |

[a] Conventional CD for crambin in 60% ethanol [63]; [b] CDCALC, PDB code 1AB1 [65] minimized by NAMD/CHARMM22 5,000 steps. Hs attached to $-CH_3$s are ignored; [c] CAPPS, Rebuilt PDB code 1AB1 [65] secondary structures including hydrogens. The following residues were ignored: 1–2 and 32–34. Grey highlight represents the lowest RMSD for each category.

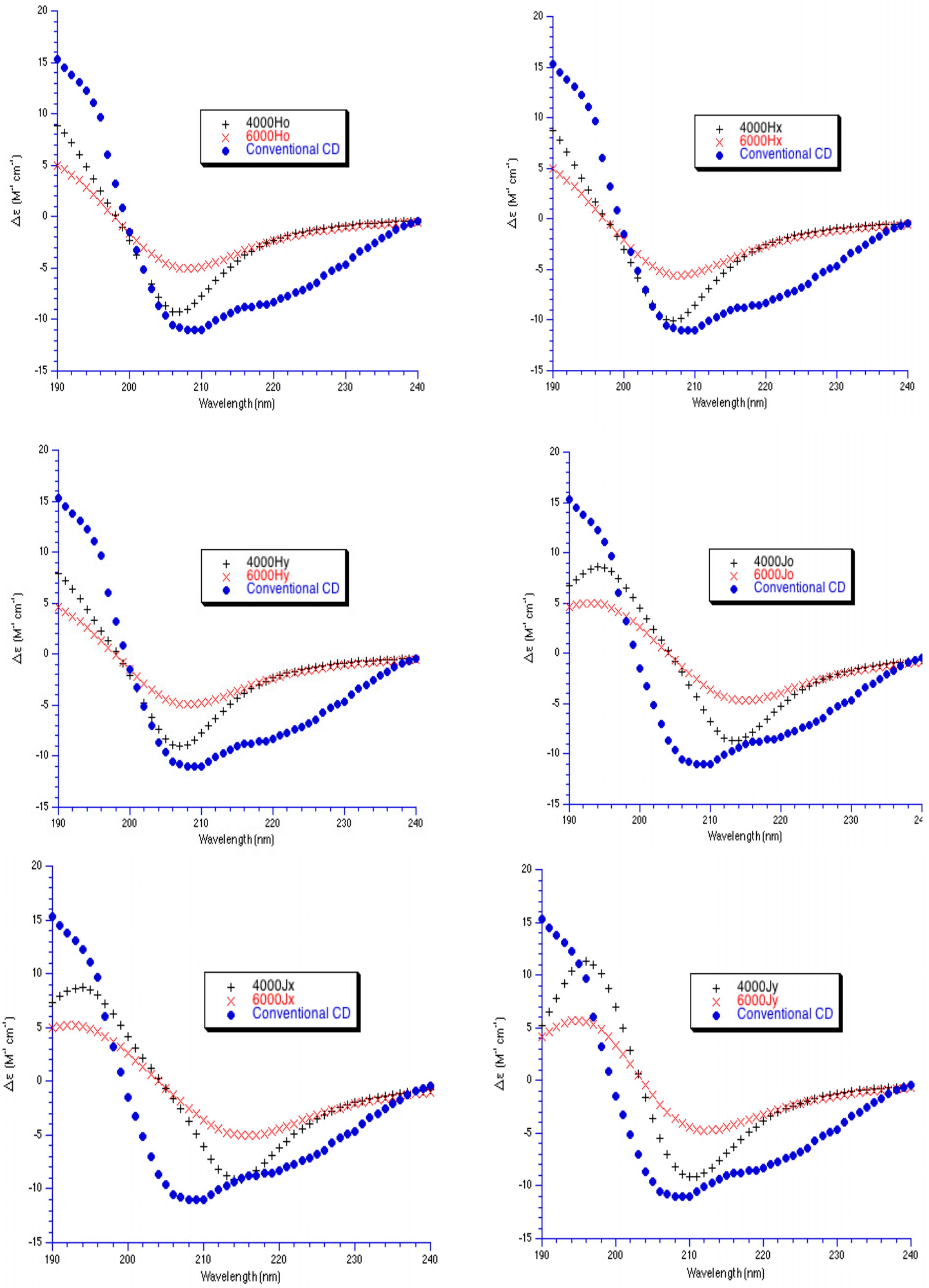


**Figure S54.** *Cont.*

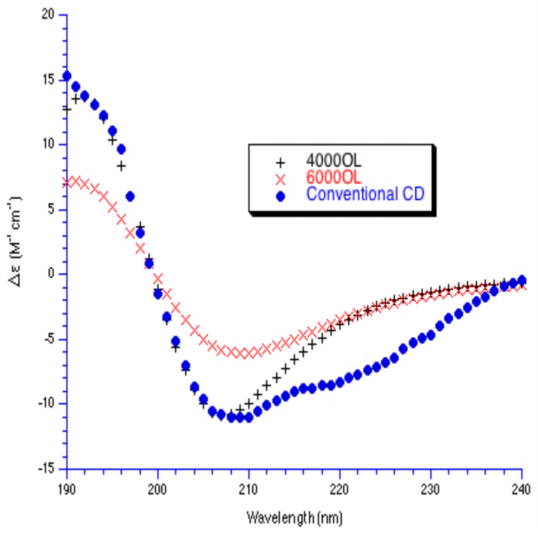


**Figure S54.** Crambin Predicted CD Using CDCALC. The PDB 1AB1 [65] structure was minimized via 5000 conjugate gradients steps with NAMD/CHARMM22. Calculated spectra ignore all $-CH_3$ group hydrogens. Bandwidths are 4000 (+) and 6000 (×) $cm^{-1}$. The blue dots (•) are the experimental conventional CD in 60% ethanol solution [63]. The CATH fold classification [53] is alpha beta/2-layer sandwich.

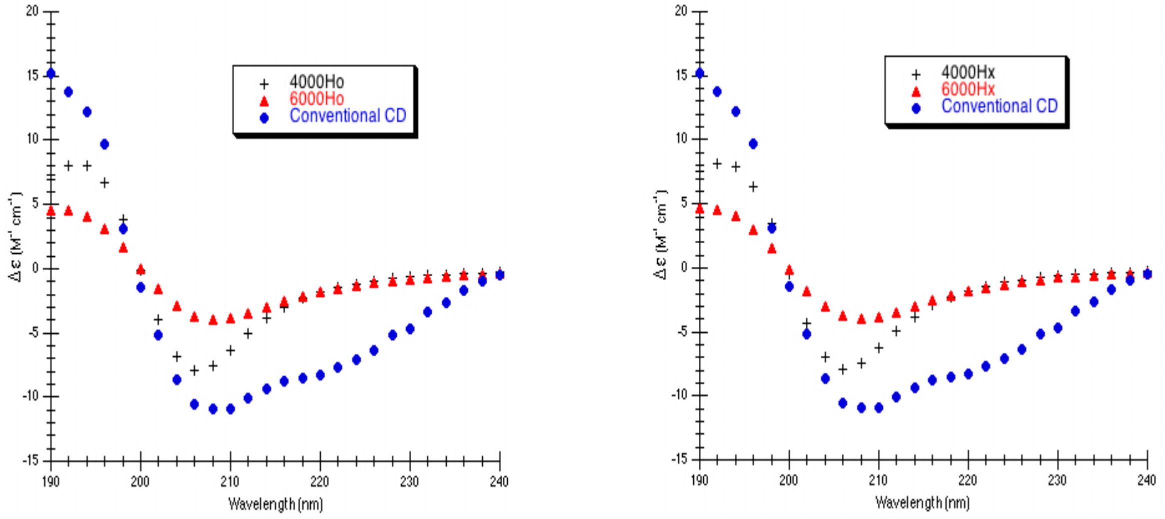


**Figure S55.** *Cont.*

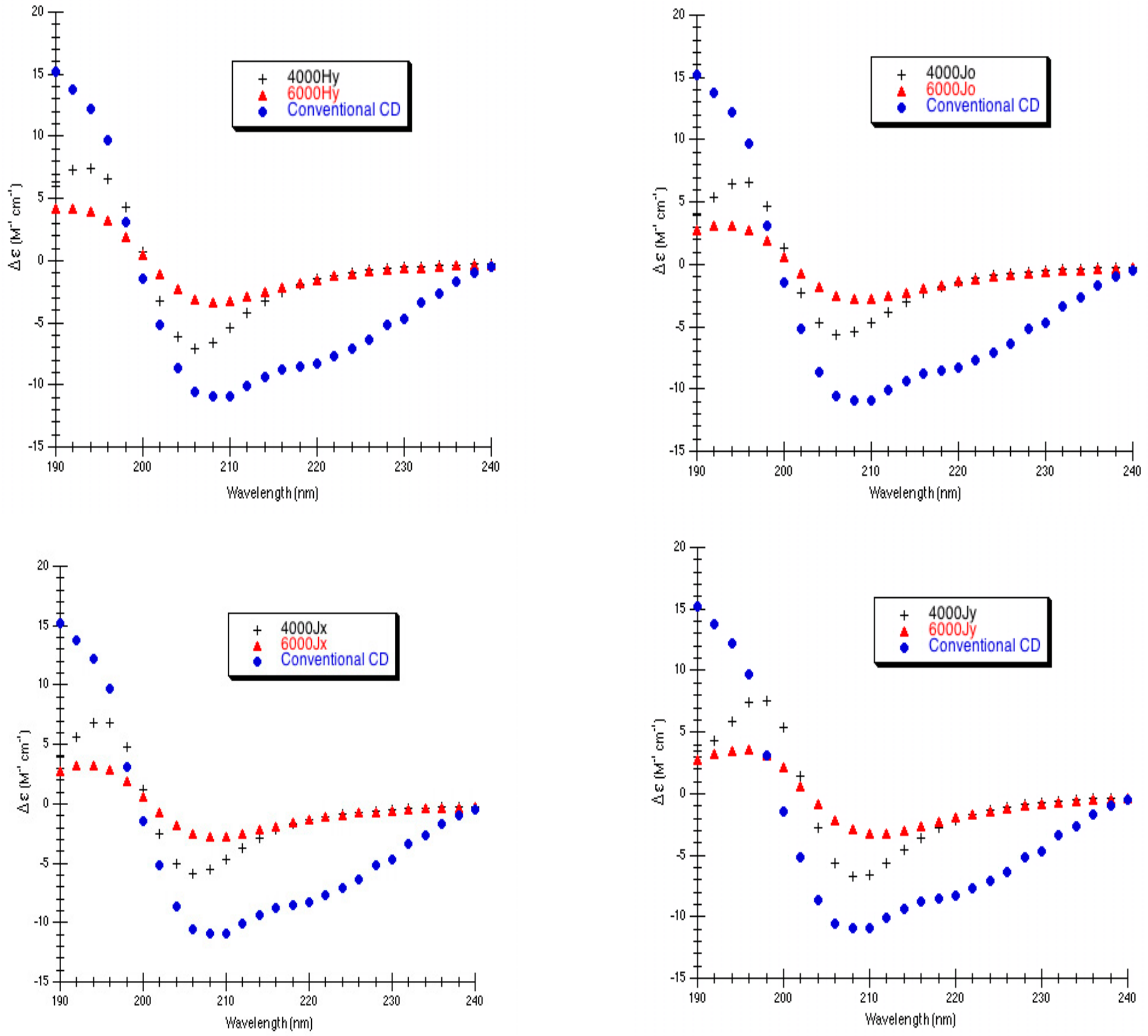


**Figure S55.** Crambin Predicted CD Using CAPPS. The PDB 1AB1 [65] structure was rebuilt ignoring residues 1–2 and 32–34. Bandwidths are 4000 (+) and 6000 (×) $cm^{-1}$. The blue dots (•) are the experimental conventional CD in 60% ethanol solution [63]. The CATH fold classification [53] is alpha-beta/2-layer sandwich.

Insulin: The peptide hormone, human insulin (PDB code 3INC [73]) is a heterodimer (Figure S56) that can form a heterododecamer made of six dimers; *i.e.*, a hexamer overall that is the storage form of insulin. The 3INC dimer has chain C of the dimer with 21 amino acids and chain D with 30 amino acids (Raghavendra, N. Pattabhi, V., Rajan, S. S. in the PDB [73]). 3INC is insulin in the R conformation (α-helical) that is complexed with $Ni^{2+}$ instead of the native $Zn^{2+}$. CATH does not classify the secondary structure of insulin [53], but the PCDDB describes the secondary structure as 46.1% α-helix, 11.8% $3_{10}$-helix, 5.9% β-strand, 2.0% β-bridge, 2.9% bonded turn, 3.9% bend, and 27.5% irregular [44]. The wild-type human insulin for which the SRCD is presented in the PCDDB contains $Zn^{2+}$ [47], and it is known that the CD spectrum is dependent on the presence and kind of metal [123].

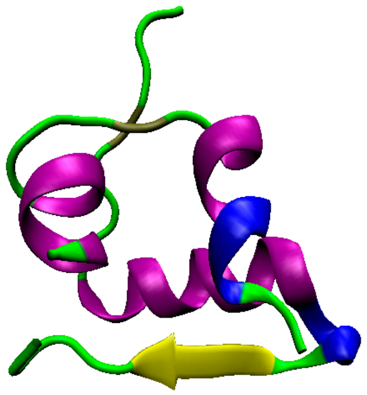

**Figure S56.** Insulin Secondary Structure. PDB code 3INC [73] human insulin showing secondary structure elements of the dimer used in CD calculations. The α-helices (chain C 1–9, 12–17; chain D 8–20) are purple in color, the β-sheet (chain D 24–26) is yellow in color, the $3_{10}$–helices (chain C 18–20; chain C 21–23) are blue in color and the turns and other structures are green in color.

**Table S22.** CD Analysis of Human Insulin (PDB code 3INC). The DInaMo calculations are for the minimized or rebuilt structure using CDCALC or CAPPS. All RMSDs are calculated between 180 and 210 nm.

| CD Method | Peak Wavelength (nm) | Δε ($M^{-1}\cdot cm^{-1}$) | Peak Wavelength (nm) | Δε ($M^{-1}\cdot cm^{-1}$) | RMSD ($M^{-1}\cdot cm^{-1}$) |
|---|---|---|---|---|---|
| [a] SRCD | 192 | 16.75 | 221 | −8.08 | 0.000 |
| [b] 4000 Ho | 189 | 18.25 | 204 | −12.06 | 9.170 |
| [b] 6000 Ho | 188 | 9.89 | 206 | −6.10 | 5.068 |
| [b] 4000 Hx | 189 | 17.87 | 204 | −12.26 | 9.290 |
| [b] 6000 Hx | 188 | 9.91 | 206 | −6.27 | 5.230 |
| [b] 4000 Hy | 188 | 16.45 | 204 | −12.40 | 9.396 |
| [b] 6000 Hy | 187 | 9.34 | 205 | −6.43 | 5.590 |
| [b] 4000 Jo | 195 | 17.07 | 211 | −11.32 | 5.256 |
| [b] 6000 Jo | 194 | 9.28 | 213 | −5.71 | 2.012 |
| [b] 4000 Jx | 195 | 16.17 | 212 | −11.07 | 4.891 |
| [b] 6000 Jx | 194 | 9.04 | 213 | −5.68 | 2.070 |
| [b] 4000 Jy | 195 | 17.98 | 208 | −12.55 | 5.679 |
| [b] 6000 Jy | 195 | 8.59 | 210 | −5.85 | 1.129 |
| [b] 4000 OL | 193 | 26.01 | 206 | −12.11 | 9.930 |
| [b] 6000 OL | 192 | 13.53 | 208 | −5.07 | 4.258 |
| [c] 4000 Ho | 189 | 14.24 | 206 | −10.34 | 7.491 |
| [c] 6000 Ho | 187 | 8.04 | 208 | −5.84 | 4.656 |
| [c] 4000 Hx | 188 | 14.14 | 206 | −10.06 | 7.731 |
| [c] 6000Hx | 187 | 8.02 | 207 | −5.77 | 4.905 |
| [c] 4000 Hy | 187 | 12.98 | 207 | −10.19 | 7.447 |
| [c] 6000 Hy | 186 | 7.73 | 208 | −5.84 | 4.899 |
| [c] 4000 Jo | 194 | 13.45 | 213 | −9.72 | 3.588 |
| [c] 6000 Jo | 193 | 7.56 | 214 | −5.48 | 1.906 |
| [c] 4000 Jx | 194 | 12.85 | 214 | −8.99 | 3.439 |

**Table S22.** *Cont.*

| CD Method | Peak Wavelength (nm) | Δε ($M^{-1}\cdot cm^{-1}$) | Peak Wavelength (nm) | Δε ($M^{-1}\cdot cm^{-1}$) | RMSD ($M^{-1}\cdot cm^{-1}$) |
|---|---|---|---|---|---|
| [c] 6000 Jx | 192 | 7.36 | 215 | −5.21 | 1.995 |
| [c] 4000 Jy | 196 | 14.10 | 210 | −10.86 | 3.650 |
| [c]6000 Jy | 195 | 7.02 | 212 | −5.52 | 0.945 |
| [c] 4000 OL | 192 | 20.80 | 207 | −9.68 | 7.412 |
| [c] 6000 OL | 192 | 11.08 | 210 | −4.68 | 3.253 |
| [d] 4000 Ho | 192 | 19.16 | 204 | −12.08 | 7.716 |
| [d] 6000 Ho | 192 | 9.50 | 206 | −5.51 | 3.418 |
| [d] 4000 Hx | 192 | 19.06 | 204 | −12.38 | 8.036 |
| [d] 6000 Hx | 188 | 9.44 | 206 | −5.67 | 3.754 |
| [d] 4000 Hy | 192 | 19.19 | 204 | −14.17 | 9.018 |
| [d] 6000 Hy | 190 | 9.79 | 206 | −6.88 | 4.438 |
| [d] 4000Jo | 196 | 13.37 | 206 | −6.39 | 2.647 |
| [d] 6000 Jo | 196 | 5.63 | 210 | −2.50 | 1.562 |
| [d] 4000 Jx | 196 | 13.21 | 206 | −6.70 | 2.511 |
| [d] 6000Jx | 196 | 5.56 | 210 | −2.64 | 1.454 |
| [d] 4000Jy | 198 | 16.08 | 208 | −10.34 | 4.612 |
| [d] 6000Jy | 196 | 7.08 | 212 | −4.46 | 1.061 |
| [e] B09:1 | 189 | 9.72 | 208 | −7.25 | 3.639 |
| [f] B09:2 | 189 | 8.93 | 208 | −8.48 | 3.476 |
| [g] B09:3 | 192 | 7.59 | 210 | −4.45 | 2.072 |

[a] SRCD of human insulin from the Protein Circular Dichroism Data Bank (CD0000040000) [44,47]; [b] CDALC using PDB structure 3INC [73] that was minimized via Insight®II/Discover/CVFF. Minimization involved 1000 steps of steepest descents followed by 100 steps of conjugate gradients. All hydrogens are included; [c] CDCALC, 3INC minimized by NAMD/CHARMM22 20,000 conjugate gradient steps. Hs attached to $-CH_3$s are ignored; [d]CAPPS, Rebuilt 3INC secondary structures including all hydrogens. The following residues are ignored: Chain A: 18−21; Chain B: 1–7, 21–23, 24–26; [e] Matrix method using 1TRZ [124] including only the protein backbone transitions [55]; [f] Matrix method using 1 TRZ [124] including protein backbone and charge-transfer transitions [55]; [g] Matrix method using 1 TRZ [124] including protein backbone, charge-transfer and side chain transitions [55]. Grey highlight represents the lowest RMSD for each category.

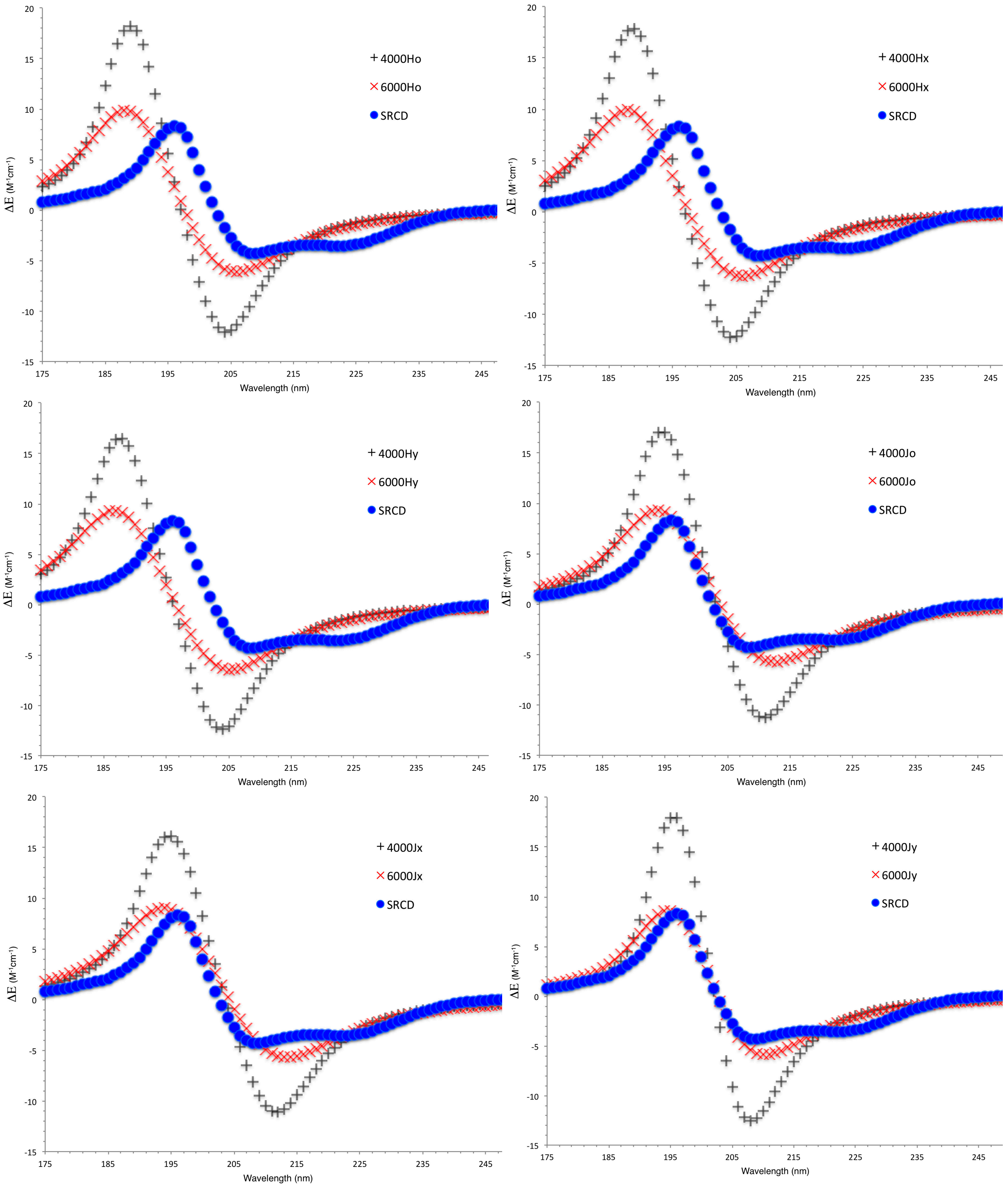


**Figure S57.** *Cont.*

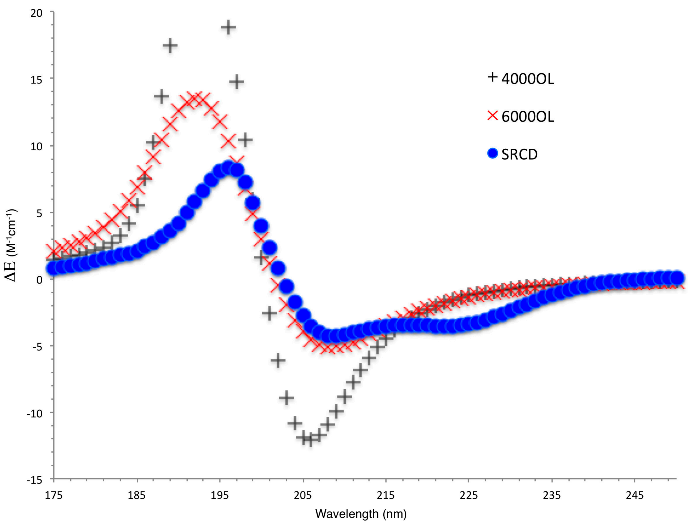


**Figure S57.** Insulin Predicted CD Using CDCALC and Insight®II/Discover/CVFF. The PDB code 3INC [73] chains C and D were minimized with 1000 steps of steepest descents followed by 100 steps of conjugate gradients. Calculated spectra include all hydrogens. Bandwidths are 4000 (+) and 6000 (×) $cm^{-1}$. The blue dots (•) are the experimental SRCD (CD0000040000) [44,47]. Insulin is not classified by the CATH fold classification [53].

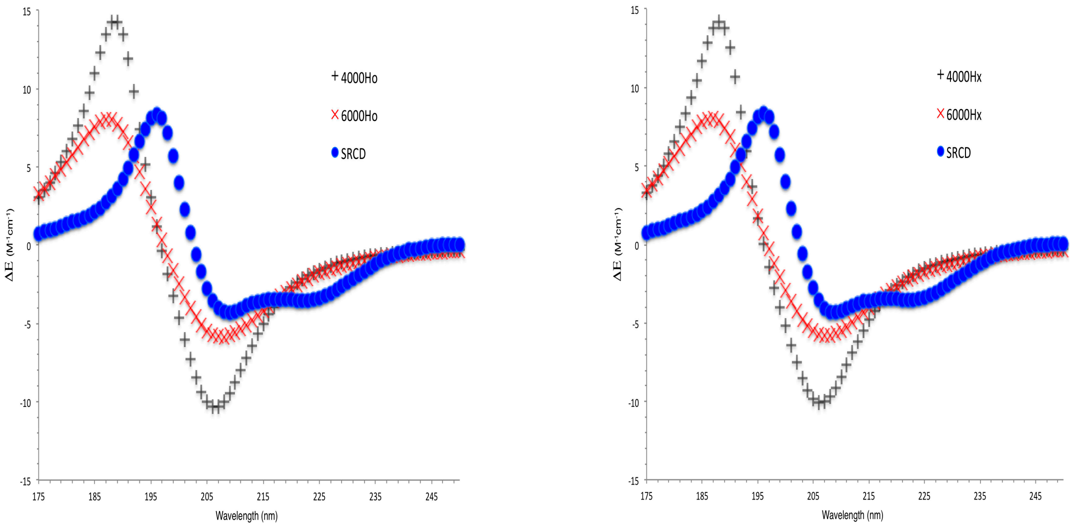


**Figure S58.** *Cont.*

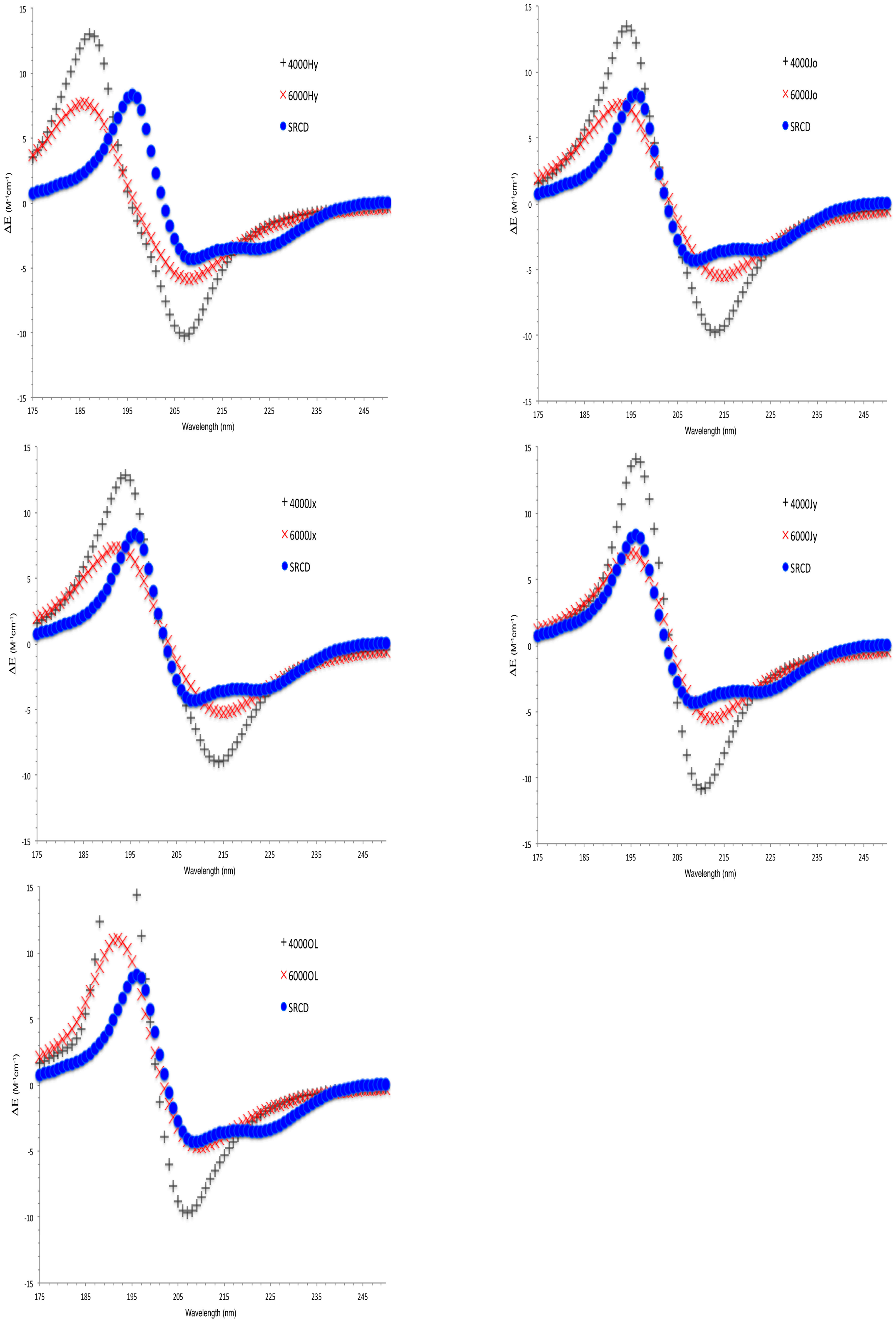


**Figure S58.** Insulin Predicted CD Using CDCALC Minimized via NAMD/CHARMM22. The dimer of chain C and D of PDB code 3INC [73] was minimized with 20,000 conjugate gradient steps. Calculated spectra ignore all $-CH_3$ group hydrogens. Bandwidths are 4000 (+) and 6000 (×) $cm^{-1}$. The blue dots (•) are the experimental SRCD (CD0000040000) [44,47]. Insulin is not classified by the CATH fold classification [53].

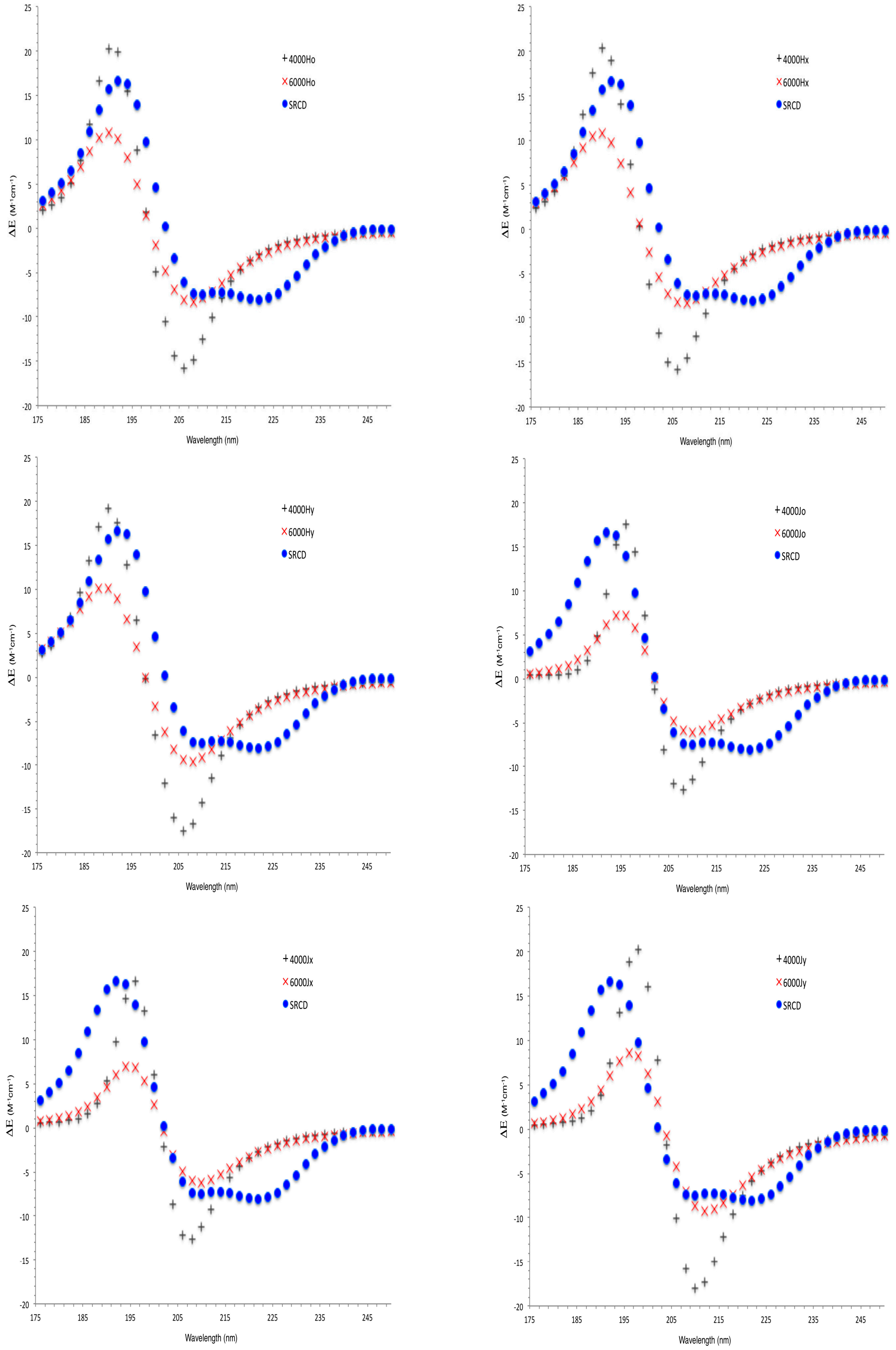


**Figure S59.** Insulin Predicted CD Using CAPPS. Secondary structures of PDB code 3INC [73] dimer are rebuilt including all hydrogens. The following residues are ignored: Chain A: 18–21; Chain B: 1–7, 21–23, 24–26. Bandwidths are 4000 (+) and 6000 (×) $cm^{-1}$. The blue dots (•) are the experimental SRCD (CD0000004000) [44,47]. Insulin is not classified by the CATH fold classification [53].

Bovine Pancreatic Trypsin Inhibitor: Aprotinin (bovine pancreatic trypsin inhibitor (BPTI)) is naturally occurring 58-residue monomer derived from bovine lung (Figure S60) [125]. It's called pancreatic because trypsin is derived from trypsinogen that is produced in the pancreas. Aprotinin inhibits trypsin and related proteolytic enzymes [125]. BPTI is a drug that reduces perioperative bleeding and shows significant reduction in blood loss and transfusion requirements [125]. BPTI folds into stable compact tertiary structures and multiple forms are found in the Protein Data Bank [73]. Herein, 5PTI [76] is chosen because it is recommended by the PCDDB along with the SRCD spectrum of aprotinin [44]. 5PTI is form II of aprotinin showing a $3_{10}$-helix near the N-terminus, an α-helix near the C-terminus, and two β-sheets in-between [76]. CATH describes the secondary structure of 5PTI as a single domain with few secondary structures and an irregular architecture [53]. The PCDDB analyzes the secondary structure to be 13.8% α-helix, 6.9% $3_{10}$-helix, 24.1% β-strand, 1.7% β-bridge, 15.5% bend, and 31.0% irregular [44].

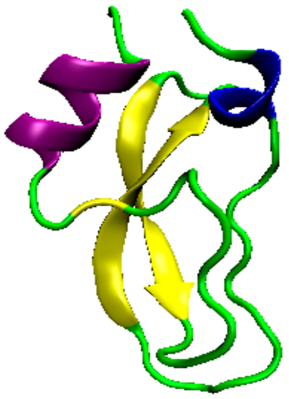

**Figure S60.** Bovine Pancreatic Trypsin Inhibitor Secondary Structure. PDB code 5PTI [76] secondary structure where the blue is the $3_{10}$-helix residues 2–7, the purple is the α-helix residues 47–56, and the yellow tapes are the β-sheets: 18–24 and 29–35. The CATH fold classification [53] is few secondary structures/irregular.

**Table S23.** CD Analysis of Bovine Pancreatic Trypsin Inhibitor. The DInaMo calculations are for the minimized or rebuilt structure using CDCALC or CAPPS. All RMSDs are calculated between 180 and 210 nm.

| CD Method | Peak Wavelength (nm) | Δε ($M^{-1}\cdot cm^{-1}$) | Peak Wavelength (nm) | Δε ($M^{-1}\cdot cm^{-1}$) | M (Root Mean Square Deviation) ($M^{-1}\cdot cm^{-1}$) |
|---|---|---|---|---|---|
| [a] SRCD | 187 | 4.52 | 202 | −7.67 | 0.000 |
| [b] 4000 Ho | 190 | 4.41 | 206 | −4.71 | 2.326 |
| [b] 6000 Ho | 188 | 2.46 | 207 | −2.76 | 3.125 |
| [b] 4000 Hx | 187 | 3.52 | 205 | −5.47 | 1.669 |
| [b] 6000 Hx | 186 | 1.97 | 206 | −3.33 | 2.777 |
| [b] 4000 Hy | 188 | 3.68 | 206 | −4.55 | 2.149 |
| [b] 6000 Hy | 186 | 2.13 | 207 | −2.79 | 3.068 |
| [b] 4000 Jo | 195 | 4.15 | 212 | −4.43 | 4.954 |
| [b] 6000 Jo | 193 | 2.31 | 213 | −2.58 | 4.532 |
| [b] 4000 Jx | 193 | 3.21 | 212 | −4.93 | 4.521 |

**Table S23.** *Cont.*

| CD Method | Peak Wavelength (nm) | Δε ($M^{-1}·cm^{-1}$) | Peak Wavelength (nm) | Δε ($M^{-1}·cm^{-1}$) | M (Root Mean Square Deviation) ($M^{-1}·cm^{-1}$) |
|---|---|---|---|---|---|
| [b] 6000 Jx | 192 | 1.82 | 214 | −2.99 | 4.238 |
| [b] 4000 Jy | 194 | 6.46 | 210 | −5.13 | 4.851 |
| [b] 6000 Jy | 192 | 3.48 | 211 | −2.77 | 4.351 |
| [b] 4000 OL | 190 | 7.18 | 206 | −6.25 | 2.971 |
| [b] 6000 OL | 189 | 3.86 | 207 | −3.42 | 3.056 |
| [c] 4000 Ho | 194 | 3.61 | 206 | −4.18 | 3.724 |
| [c] 6000 Ho | 192 | 1.95 | 208 | −2.09 | 3.913 |
| [c] 4000 Hx | 192 | 3.06 | 206 | −3.90 | 3.667 |
| [c] 6000 Hx | 190 | 1.78 | 208 | −1.95 | 3.925 |
| [c] 4000 Hy | 194 | 3.02 | 206 | −4.16 | 3.634 |
| [c] 6000 Hy | 188 | 1.83 | 208 | −2.07 | 3.864 |
| [c] 4000 Jo | 196 | 2.41 | 206 | −3.35 | 4.287 |
| [c] 6000 Jo | 194 | 0.87 | 208 | −1.69 | 4.269 |
| [c] 4000 Jx | 196 | 2.34 | 206 | −3.03 | 4.123 |
| [c] 6000 Jx | 194 | 0.98 | 208 | −1.65 | 4.206 |
| [c] 4000 Jy | 184 | 1.11 | 210 | −4.44 | 4.687 |
| [c] 6000 Jy | 196 | 1.14 | 210 | −2.24 | 4.352 |
| [d] RH04:1 | 193 | 5.64 | 210 | −2.56 | 3.932 |
| [e] RH04:2 | 190 | 8.84 | 207 | −7.25 | 2.773 |
| [f] RH04:3 | 187 | 6.72 | 205 | −6.48 | 1.629 |
| [g] SMPZGW:1 | 192 | 9.65 | 216 | −4.05 | 7.100 |
| [h] SMPZGW:2 | 189 | 8.07 | 208 | −4.89 | 4.221 |
| [i] SMPZGW:3 | 191 | 10.47 | 210 | −4.45 | 5.940 |

[a] SRCD from the PCDDB code CD0000007000 [44,47]; [b] CDCALC using PDB structure 5PTI minimized via NAMD/CHARMM22 and 5000 conjugate gradient steps. The hydrogens on all −$CH_3$ groups are ignored; [c] CAPPS results using PDB structure 5PTI that contained rebuilt secondary structures including hydrogens; the following residues were ignored: 1, 45, 46, 57, and 58; [d] Matrix method on PDB code 5PTI [76] including *ab initio* protein backbone parameters only [68]; [e] Matrix method on PDB code 5PTI [76] including *ab initio* protein backbone and semi-empirical side chain parameters [68]; [f] Matrix method on PDB code 5PTI [76] including *ab initio* protein backbone and *ab initio* side chain parameters [68]; [g] Matrix method on PDB code 4PTI (form I of BPTI) including CNDO/S backbone, tyrosine, phenylalanine and disulfide transitions [126]. [h] Matrix method on PDB code 5PTI (form II of BPTI) including CNDO/S backbone, tyrosine, phenylalanine and disulfide transitions [126]; [i] Matrix method on PDB code 6PTI (from III of BPTI) including CNDO/S backbone, tyrosine, phenylalanine and disulfide transitions [126]. Grey highlight represents the lowest RMSD for each category.

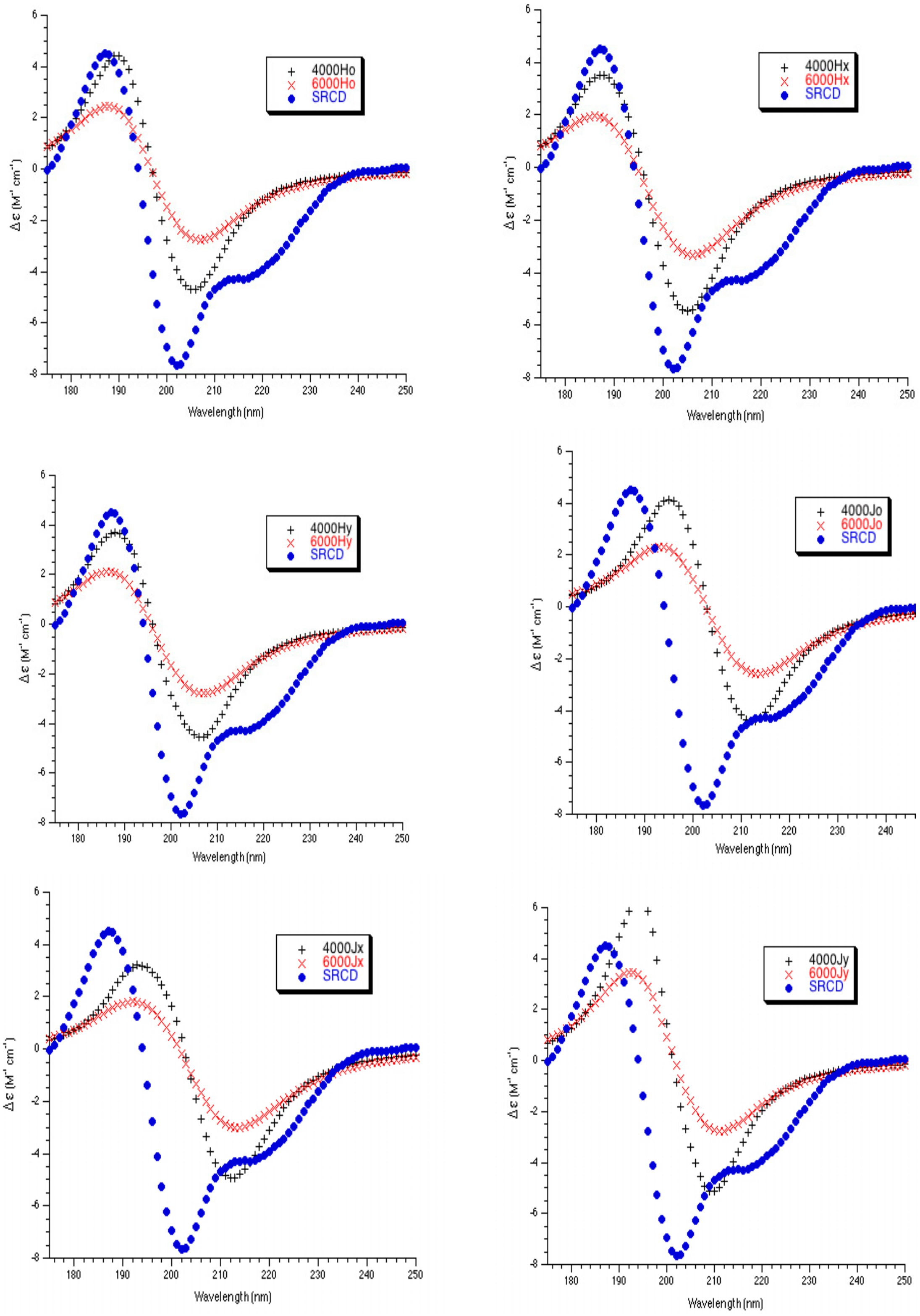


**Figure S61.** *Cont.*

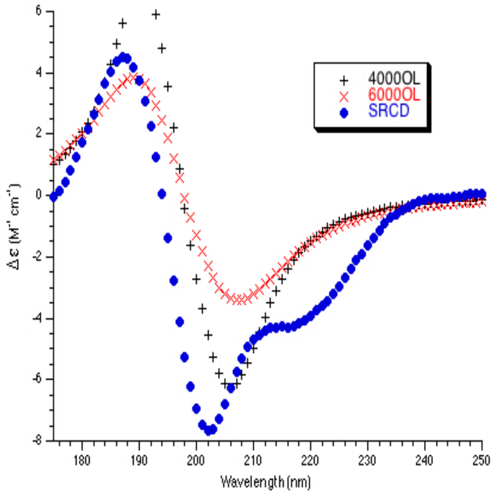


**Figure S61.** Bovine Pancreatic Trypsin Inhibitor Predicted CD Using CDCALC. The PDB code 5PTI [76] was minimized via NAMD/CHARMM22 and 5000 conjugate gradient steps. The hydrogens on all −CH3 groups are ignored. Bandwidths are 4000 (+) and 6000 (×) $cm^{-1}$. The blue dots (•) are the experimental SRCD (CD000007000) [44,47]. The CATH fold classification [53] is few secondary structures/irregular.

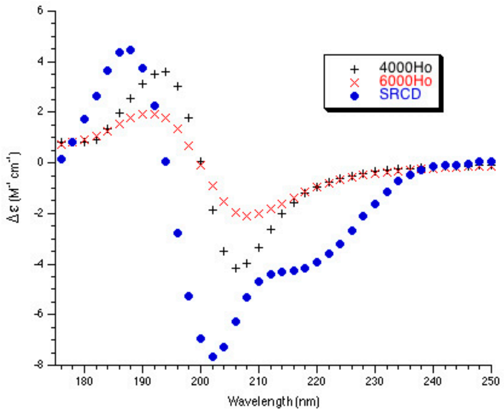


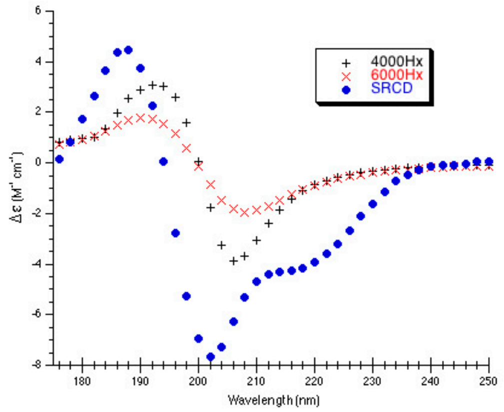


**Figure S62.** *Cont.*

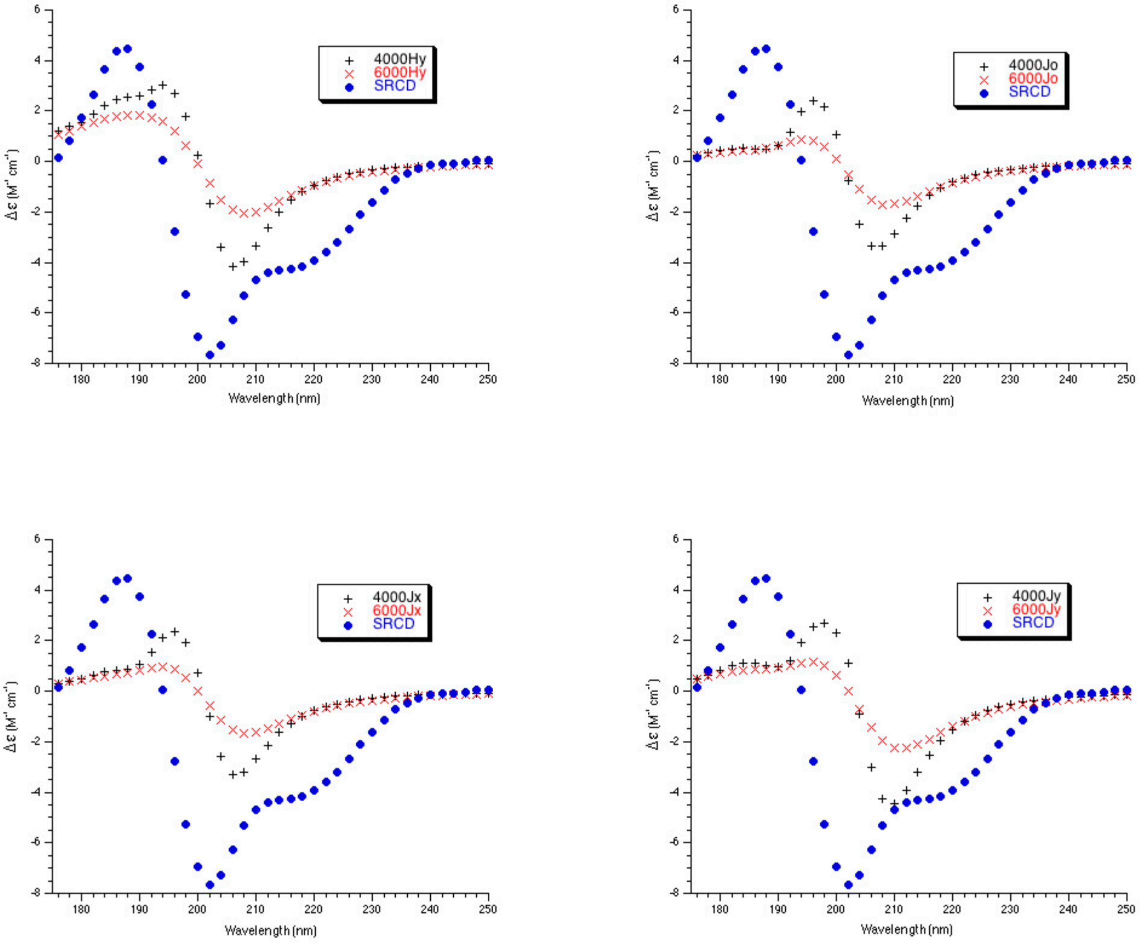


**Figure S62.** Bovine Pancreatic Trypsin Inhibitor Predicted CD Using CAPPS. The PDB code 5PTI [76] rebuilt secondary structures including hydrogens; the following residues are ignored: 1, 45, 46, 57, and 58. Bandwidths are 4000 (+) and 6000 (×) $cm^{-1}$. The blue dots (•) are the experimental SRCD (CD0000007000) [44,47]. The CATH fold classification [53] is few secondary structures/irregular.

**Table S24.** CD Analysis of Light-Harvesting Protein Complex II. The DInaMo calculations are for the minimized or rebuilt structure using CDCALC or CAPPS. All RMSDs are calculated between 180 nm and 210 nm.

| CD Method | Peak Wavelength (nm) | Δε ($M^{-1}{\cdot}cm^{-1}$) | Peak Wavelength (nm) | Δε ($M^{-1}{\cdot}cm^{-1}$) | RMSD ($M^{-1}{\cdot}cm^{-1}$) |
|---|---|---|---|---|---|
| [a] SRCD | 191 | 18.12 | 210 | −6.97 | 0.000 |
| [b] 4000Ho | 189 | 18.53 | 207 | −13.71 | 6.675 |
| [b] 6000Ho | 189 | 9.16 | 208 | −7.78 | 6.828 |
| [b] 4000Hx | 189 | 18.84 | 207 | −14.96 | 7.352 |
| [b] 6000Hx | 189 | 9.36 | 208 | −8.63 | 7.082 |
| [b] 4000Hy | 189 | 16.27 | 208 | −13.13 | 6.643 |
| [b] 6000Hy | 189 | 8.26 | 209 | −7.60 | 7.221 |

**Table S24.** *Cont.*

| CD Method | Peak Wavelength (nm) | Δε ($M^{-1}·cm^{-1}$) | Peak Wavelength (nm) | Δε ($M^{-1}·cm^{-1}$) | RMSD ($M^{-1}·cm^{-1}$) |
|---|---|---|---|---|---|
| [b] 4000 Jo | 195 | 17.46 | 214 | −12.87 | 6.439 |
| [b] 6000 Jo | 195 | 8.58 | 215 | −7.30 | 6.870 |
| [b] 4000 Jx | 195 | 16.89 | 215 | −13.53 | 6.004 |
| [b] 6000 Jx | 195 | 8.50 | 216 | −7.82 | 6.659 |
| [b] 4000 Jy | 197 | 22.95 | 214 | −12.94 | 10.390 |
| [b] 6000 Jy | 197 | 10.40 | 214 | −6.86 | 8.155 |
| [b] 4000 OL | 192 | 29.02 | 209 | −16.42 | 8.013 |
| [b] 6000 OL | 192 | 13.81 | 211 | −8.90 | 4.503 |
| [c] 4000 Ho | 190 | 23.91 | 208 | −20.50 | 9.104 |
| [c] 6000 Ho | 190 | 11.98 | 208 | −11.90 | 6.349 |
| [c] 4000 Hx | 190 | 23.60 | 206 | −21.18 | 9.834 |
| [c] 6000 Hx | 188 | 11.93 | 208 | −11.84 | 6.865 |
| [c] 4000 Hy | 190 | 23.90 | 208 | −23.52 | 10.537 |
| [c] 6000 Hy | 188 | 12.08 | 208 | −13.70 | 7.108 |
| [c] 4000 Jo | 196 | 19.27 | 208 | −17.30 | 8.307 |
| [c] 6000 Jo | 194 | 7.98 | 210 | −9.46 | 7.267 |
| [c] 4000 Jx | 196 | 18.40 | 208 | −17.81 | 5.182 |
| [c] 6000 Jx | 194 | 7.73 | 210 | −9.71 | 7.264 |
| [c] 4000 Jy | 196 | 23.14 | 212 | −24.74 | 10.278 |
| [c] 6000 Jy | 196 | 9.98 | 214 | −13.83 | 7.054 |

[a] SRCD from the Protein Circular Dichroism Data Bank CD0000114000 [44,59]; [b] CDCALC, PDB code 1NKZ [67] A/B dimer NAMD/CHARMM22 minimized for 5000 steps. Hs attached to $-CH_3$s are ignored; [c] CAPPS, Rebuilt PDB code 1NKZ [67] secondary structures only. The following residues were ignored, all in chain A: 2-4, 10. All hydrogens included. Grey highlight represents the lowest RMSD for each category.

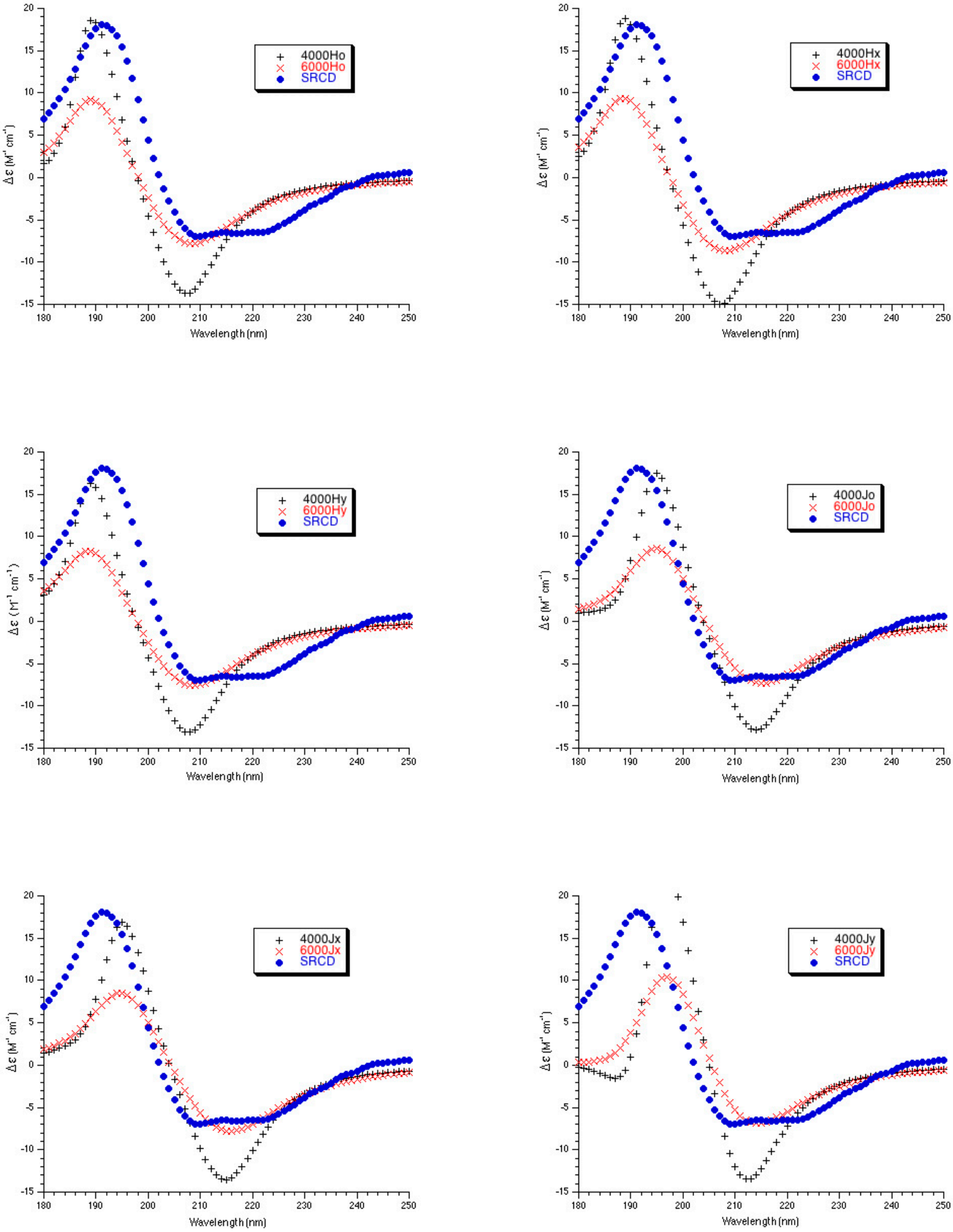


**Figure S63.** *Cont.*

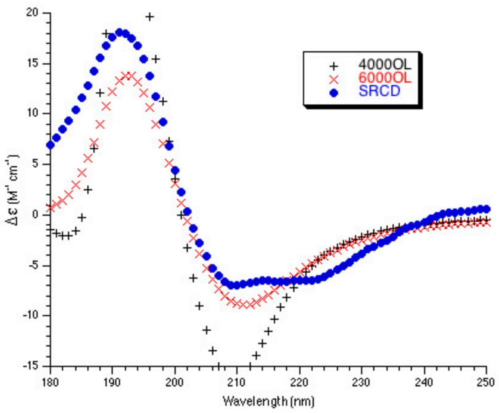


**Figure S63.** Light−Harvesting Protein Complex II CDCALC Predicted CD. The PDB 1NKZ [67] A/B dimer was minimized with 5,000 conjugate gradient steps using NAMD/CHARMM22. Calculated spectra ignore all $-CH_3$ group hydrogens. Bandwidths are 4000 (+) and 6000 (×) $cm^{-1}$. The blue dots (•) are the experimental SRCD (CD0000114000) [44,59]. The CATH fold classification [53] is a combination of few secondary structures/irregular for chain A and mainly alpha/up-down bundle for chain B. Note: the complete hexameric form of the protein was not treated and neither were the any of the ligands (bacteriochlorophyll A, benzamidine, β-octylgucoside, rhodopin glucoside).

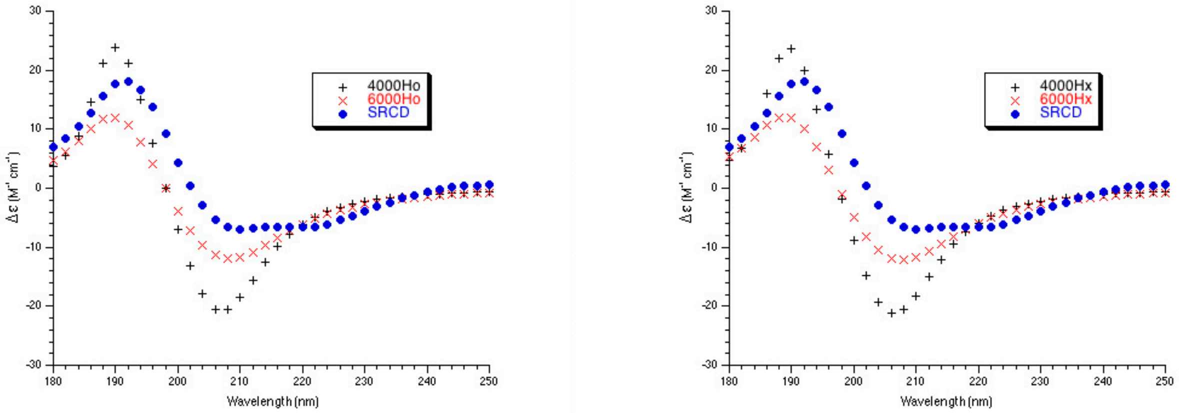


**Figure S64.** *Cont.*

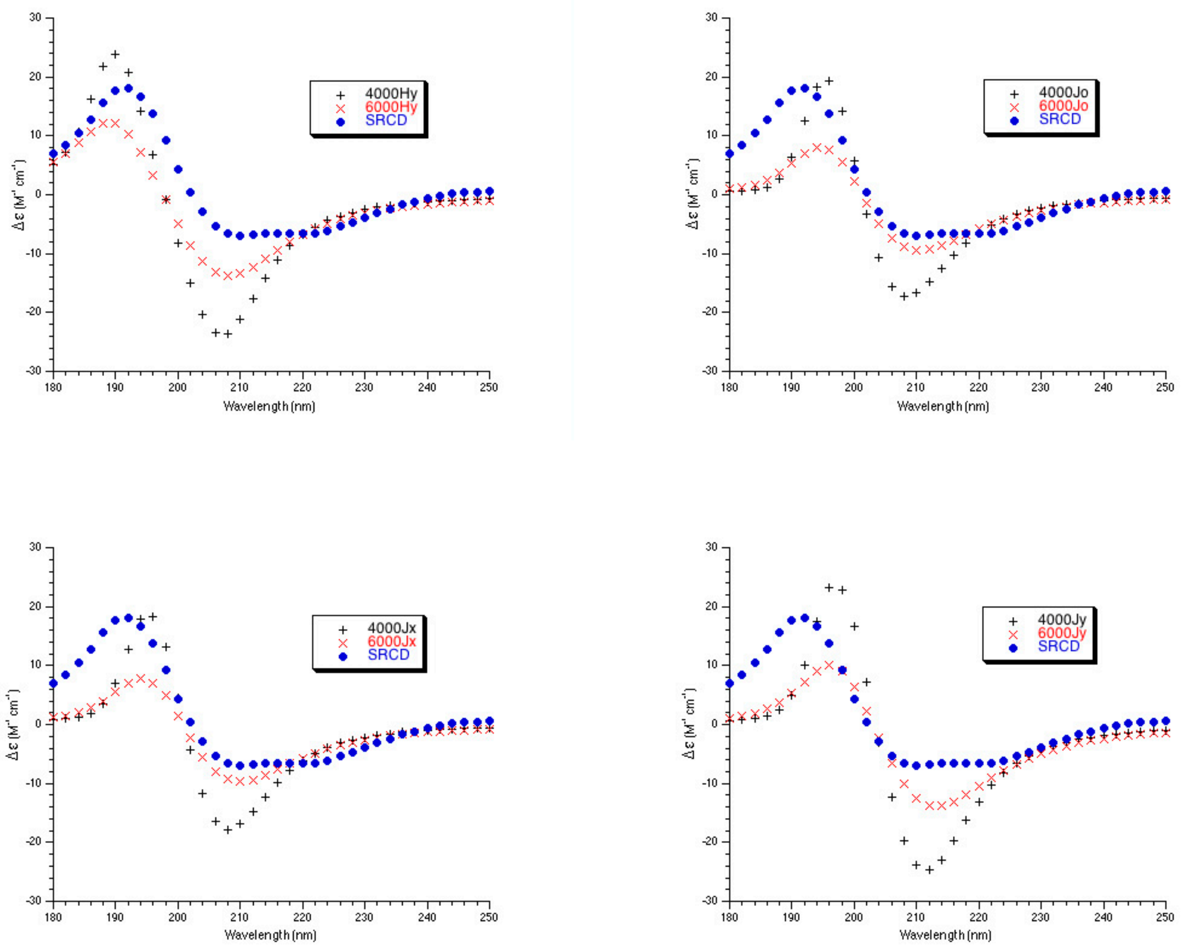


**Figure S64.** Light Harvesting Protein Complex II Predicted CD Using CAPPS. The PDB 1NKZ [67] dimer structure was rebuilt ignoring residues A2-4 and A10. The complete hexameric form of the protein was not treated and neither were the any of the ligands (bacteriochlorophyll A, benzamidine, β-octylgucoside, rhodopin glucoside). Bandwidths are 4000 (+) and 6000 (×) cm$^{-1}$. The blue dots (•) are the experimental SRCD (CD0000144000) [44,59]. The CATH fold classification [53] is a combination of few secondary structures/irregular for chain A and mainly alpha/up−down bundle for chain B.
